# Supplementary figures and images for: Assessing the evolution of research topics in a biological field using plant science as an example
Source: PLoS Biol. 2024 May 23;22(5):e3002612. doi: 10.1371/journal.pbio.3002612 (PMC11115244; doi:10.1371/journal.pbio.3002612)

Figure S1

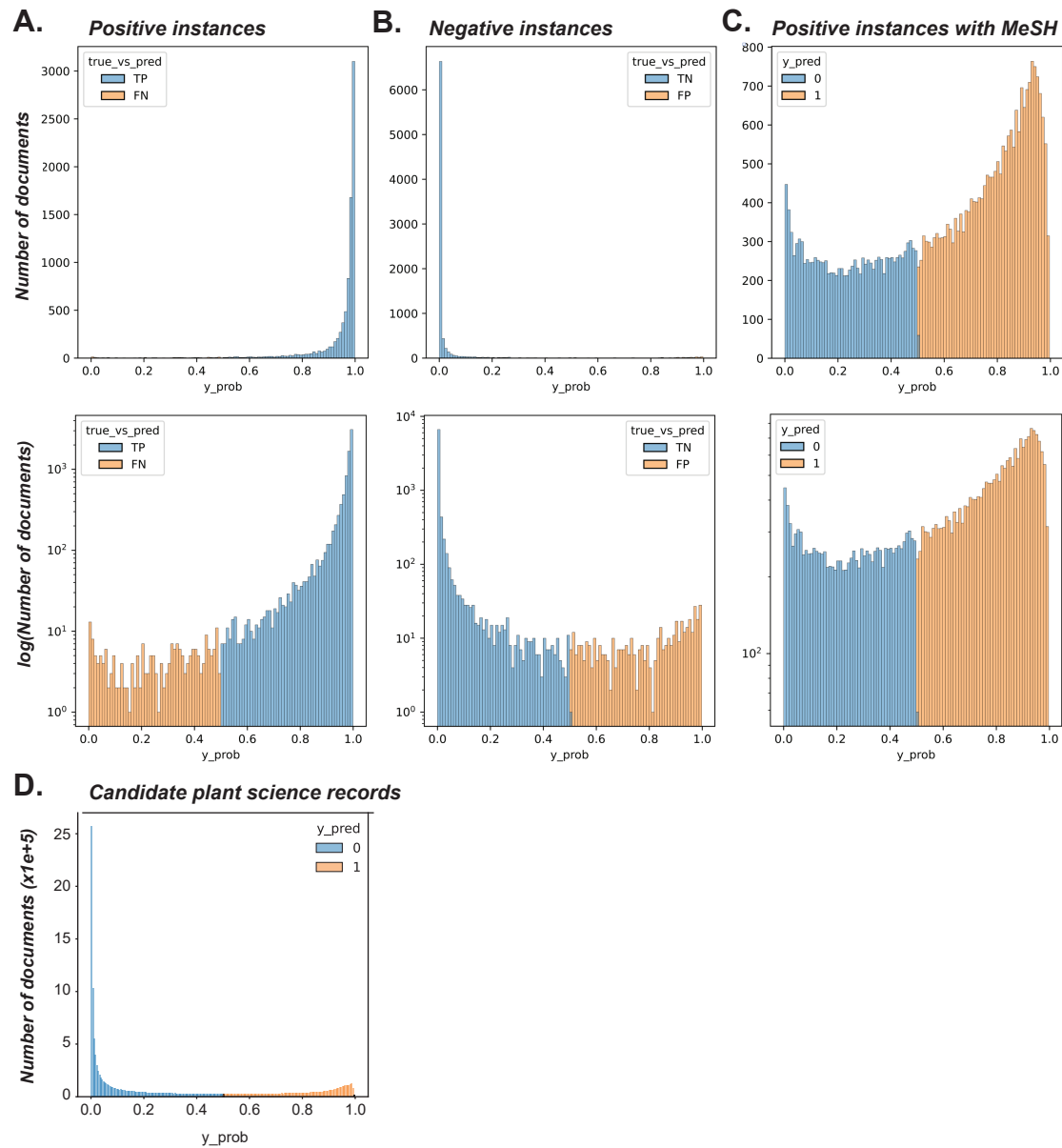

Supplement: S1 Fig — (A–C) Distributions of prediction probabilities (y_prob) of (A) positive instances (plant science records), (B) negative instances (non-plant science records), and (C) positive instances with the Medical Subject Heading “Plants” (ID = D010944). The data are color coded in blue and orange if they are correctly and incorrectly predicted, respectively. The lower subfigures contain log10-transformed x axes for the same distributions as the top subfigure for better visualization of incorrect predictions. (D) Prediction probability distribution for candidate plant science records. Prediction probabilities plotted here are available in S13 Data. (PDF) [file pbio.3002612.s001.pdf]

Figure S2

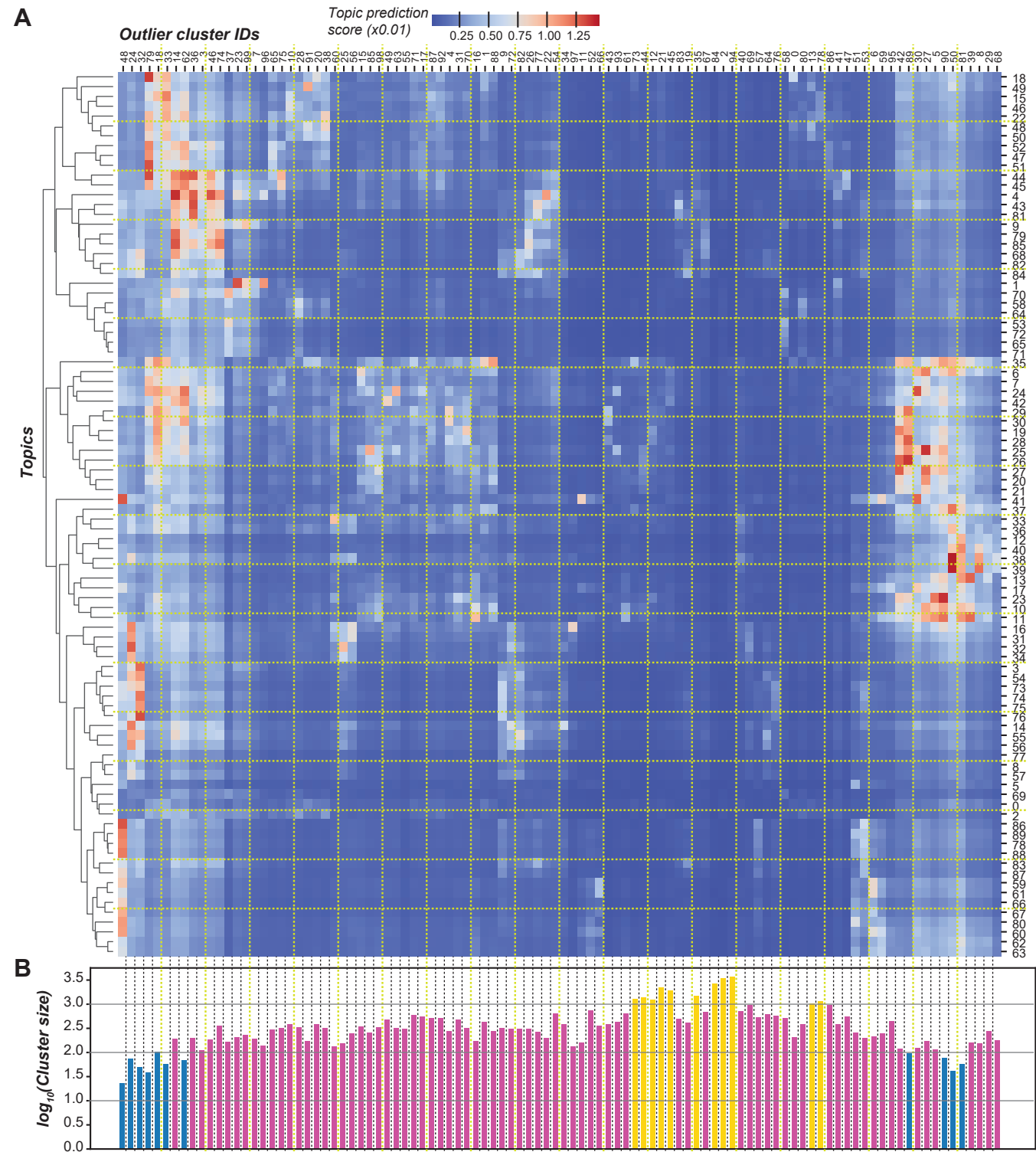

Supplement: S2 Fig — (A) Heatmap demonstrating that some outlier clusters tend to have high prediction scores for multiple topics. Each cell shows the average prediction score of a topic for records in an outlier cluster. (B) Size of outlier clusters. (PDF) [file pbio.3002612.s002.pdf]

**Figure S4**

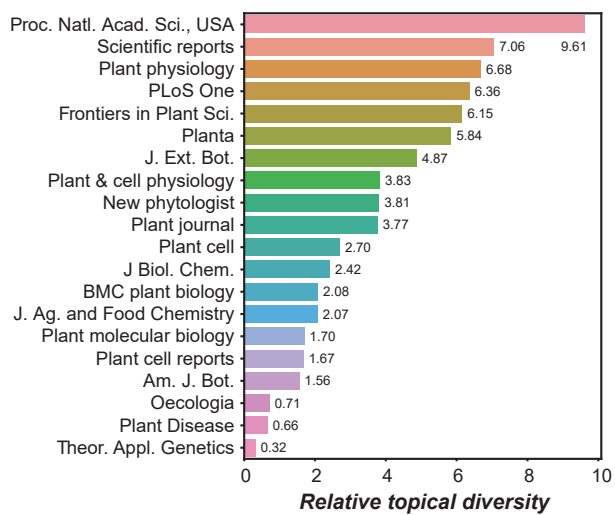

Supplement: S4 Fig — The 20 journals with the most plant science records are shown. The journal names were taken from the journal list in PubMed (https://www.nlm.nih.gov/bsd/serfile_addedinfo.html). (PDF) [file pbio.3002612.s004.pdf]

Figure S5

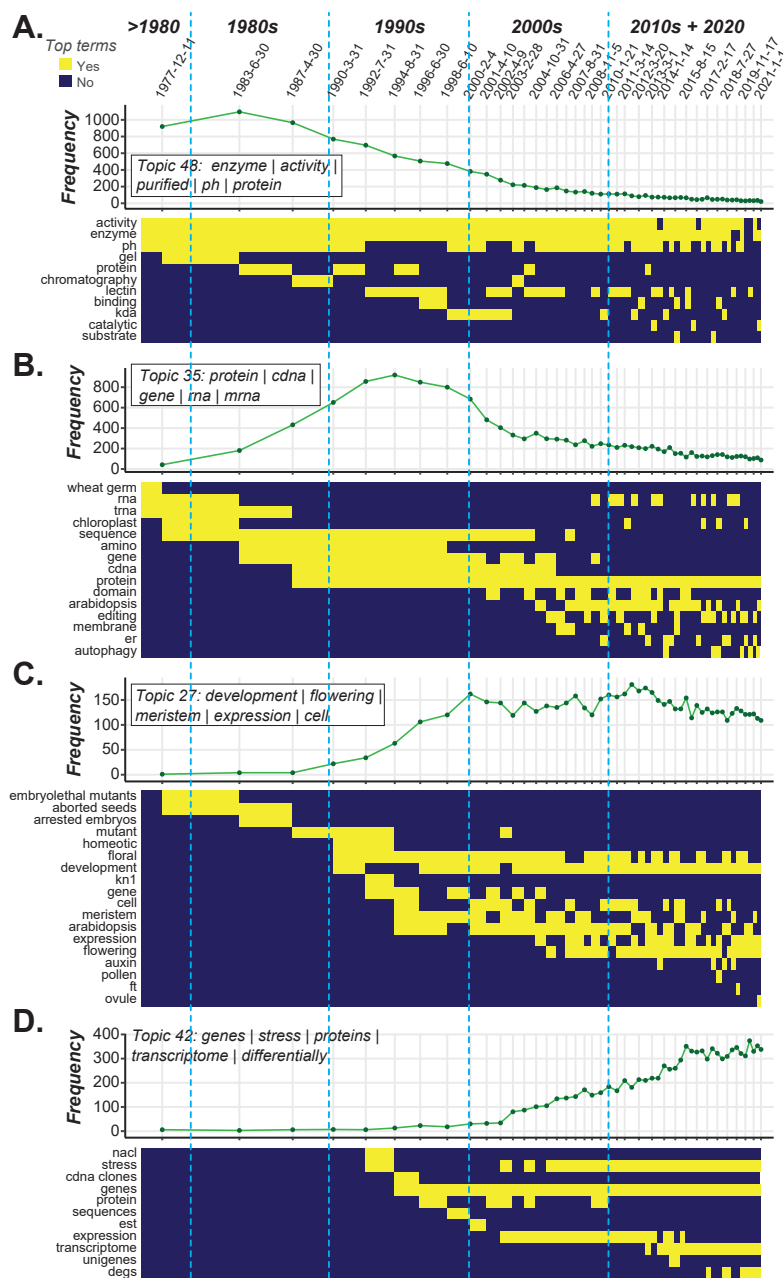

Supplement: S5 Fig — (A-D) Different patterns of topical frequency distributions for example topics (A) 48, (B) 35, (C) 27, and (D) 42. For each topic, the top graph shows the frequency of topical records in each time bin, which are the same as those in Fig 3 (green line), and the end date for each bin is indicated. The heatmap below each line plot depicts whether a term is among the top terms in a time bin (yellow) or not (blue). Blue dotted lines delineate different decades (see S5 Data for the original frequencies, S6 Data for the LOWESS fitted frequencies and the top terms for different topics/time bins). (PDF) [file pbio.3002612.s005.pdf]

Figure S6

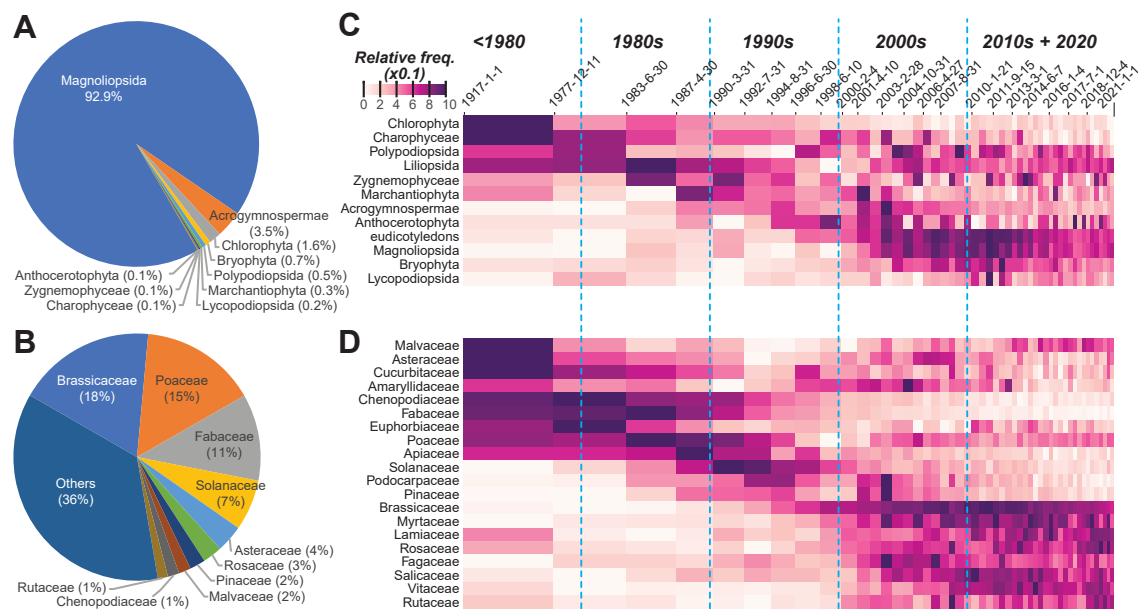

Supplement: S6 Fig — (A, B) Percentage of records mentioning specific taxa at the (A) major lineage and (B) family levels. (C, D) The prevalence of taxon mentions over time at the (C) major lineage and (E) family levels. The data used for plotting are available in S9 Data. (PDF) [file pbio.3002612.s006.pdf]

Figure S7

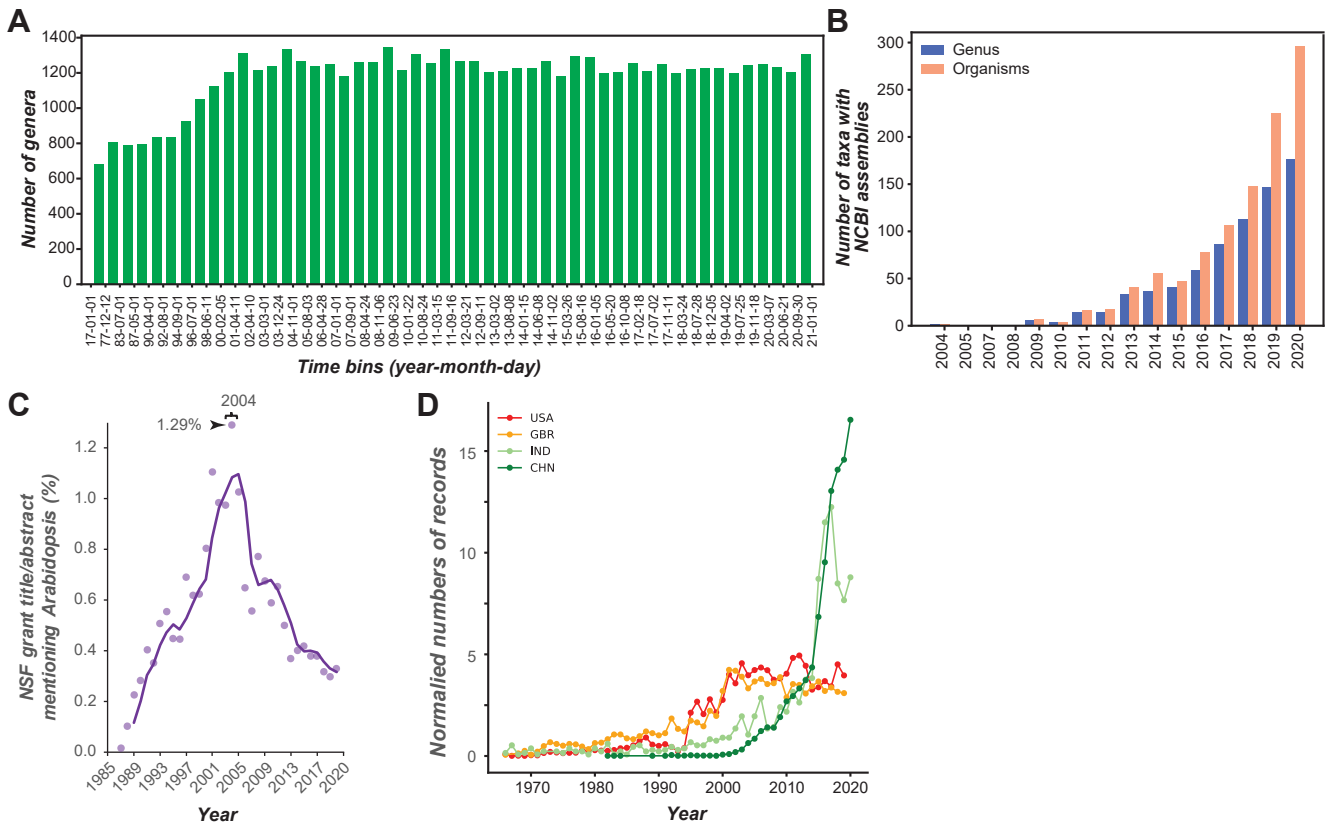

Supplement: S7 Fig — (A) Number of genera being mentioned in plant science records during different time bins (the date indicates the end date of that bin, exclusive). (B) Numbers of genera (blue) and organisms (salmon) with draft genomes available from National Center of Biotechnology Information in different years. (C) Percentage of US National Science Foundation (NSF) grants mentioning the genus Arabidopsis over time with peak percentage and year indicated. The data for (A–C) are in S9 Data. (D) Number of plant science records in the top 17 plant science journals from the USA (red), Great Britain (GBR) (orange), India (IND) (light green), and China (CHN) (dark green) normalized against the total numbers of publications of each country over time in these 17 journals. The data used for plotting can be found in S11 Data. (PDF) [file pbio.3002612.s007.pdf]

Figure S8

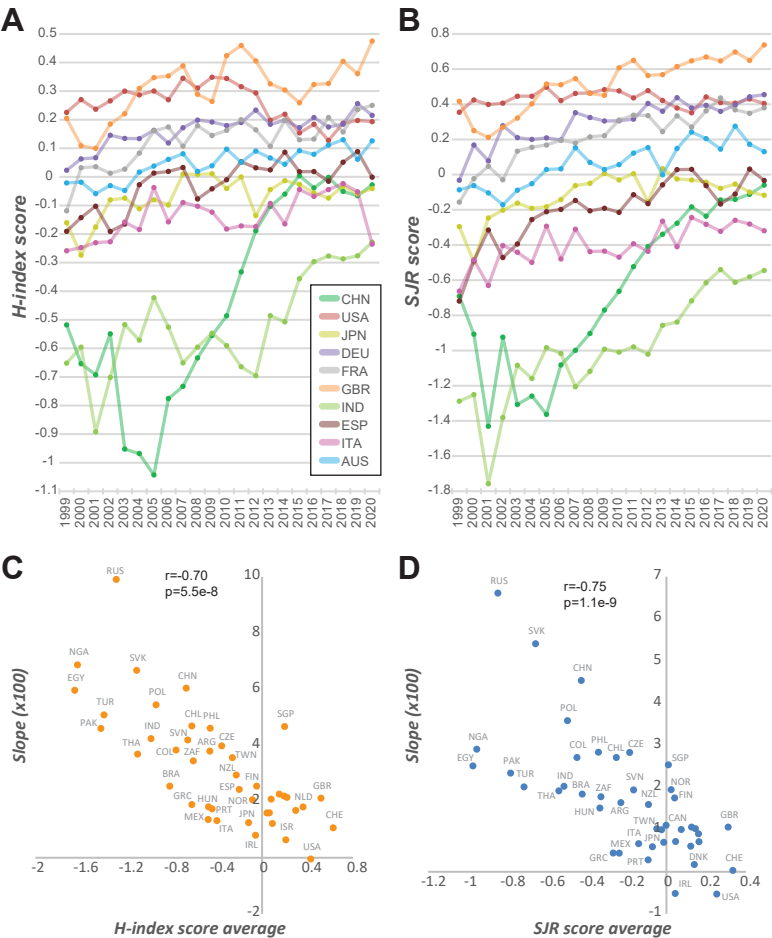

Supplement: S8 Fig — (A, B) Difference in 2 impact metrics from 1999 to 2020 for the 10 countries with the highest number of plant science records. (A) H-index. (B) SCImago Journal Rank (SJR). (C, D) Plots show the relationships between the impact metrics (H-index in (C), SJR in (D)) averaged from 1999 to 2020 and the slopes of linear fits with years as the predictive variable and impact metric as the response variable for different countries (A3 country codes shown). The countries with >400 records and with <10% missing impact values are included. The data used for plotting can be found in S11 Data. (PDF) [file pbio.3002612.s008.pdf]

Figure S9

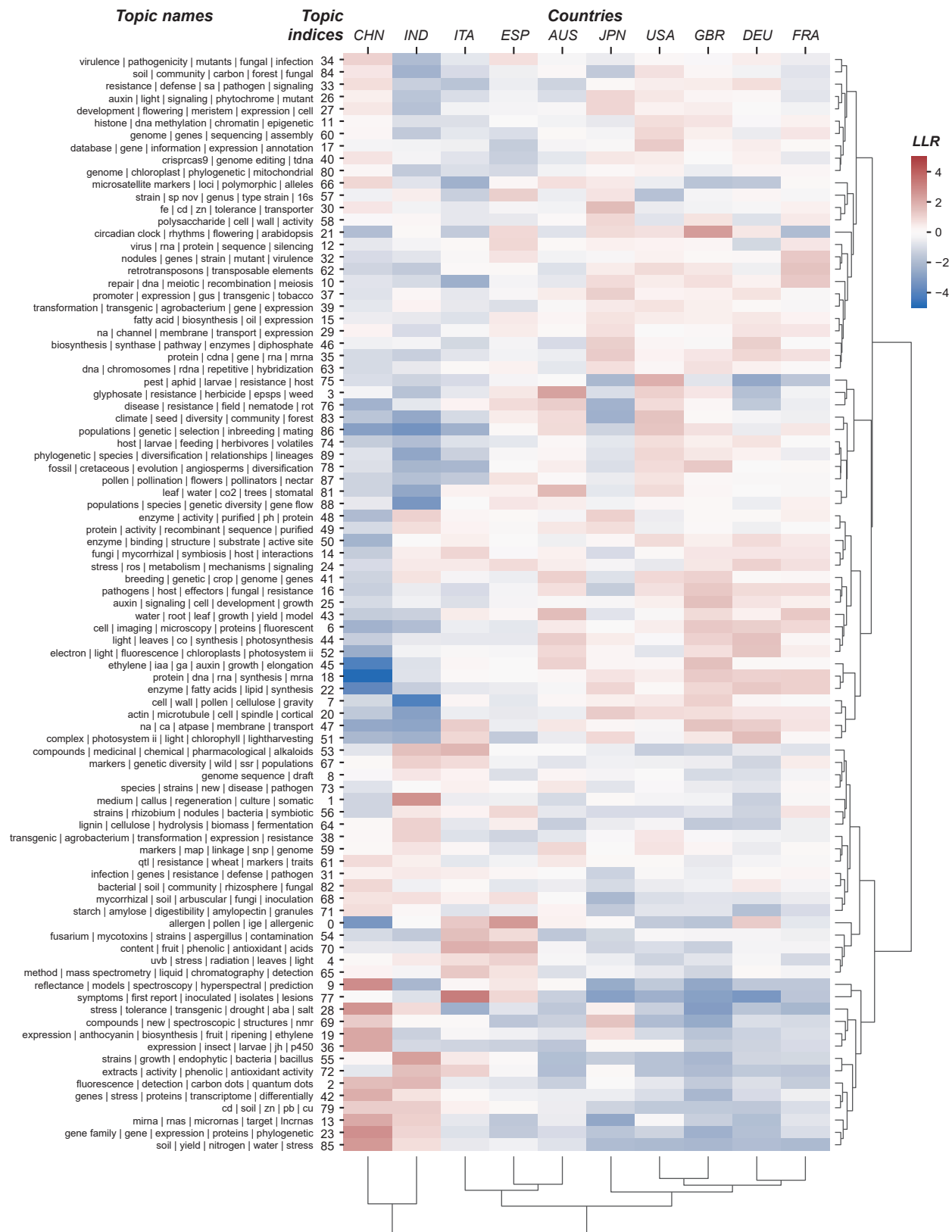

Supplement: S9 Fig — Enrichment scores (LLR, log likelihood ratio) of topics for each of the top 10 countries. Red: overrepresentation, blue: underrepresentation. The data for plotting can be found in S12 Data. (PDF) [file pbio.3002612.s009.pdf]

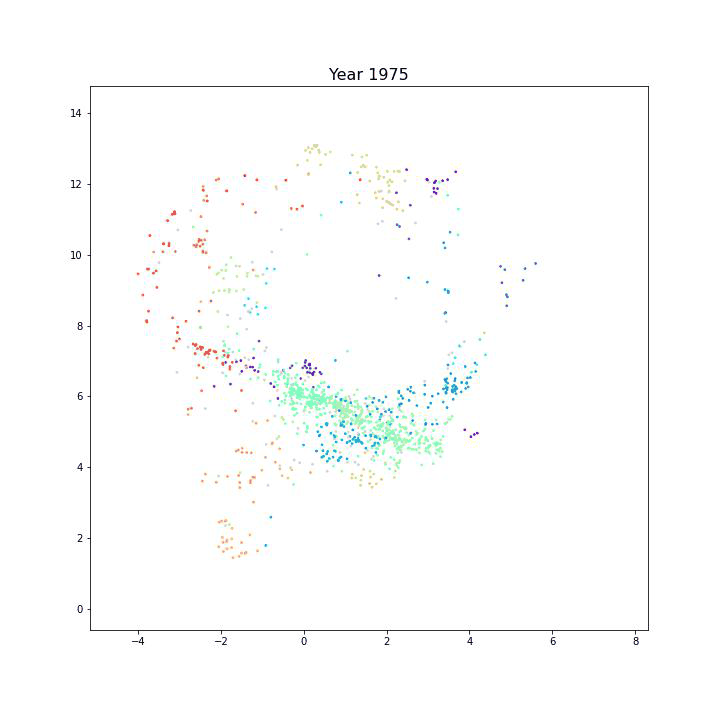

Supplement: S5 Data — The 2D embedding generated with UMAP was used to plot document relationships for each year. The plots from 1975 to 2020 were compiled into an animation. (GIF) [file pbio.3002612.s014.gif]
