# Supplementary material for: Assessing the evolution of research topics in a biological field using plant science as an example
Source: PLoS Biol. 2024 May 23;22(5):e3002612. doi: 10.1371/journal.pbio.3002612 (PMC11115244; doi:10.1371/journal.pbio.3002612)
Supplement: S3 Fig — (A) Heatmap showing cosine similarities between topic pairs. Top-left: hierarchical clustering of the cosine similarity matrix using the Ward algorithm. The branches are colored to indicate groups of related topics. (B) Topic labels and names. The topic ordering was based on hierarchical clustering of topics. Colored rectangles: neighboring topics with >0.5 cosine similarities. (PDF) [file pbio.3002612.s003.pdf]

Figure S3

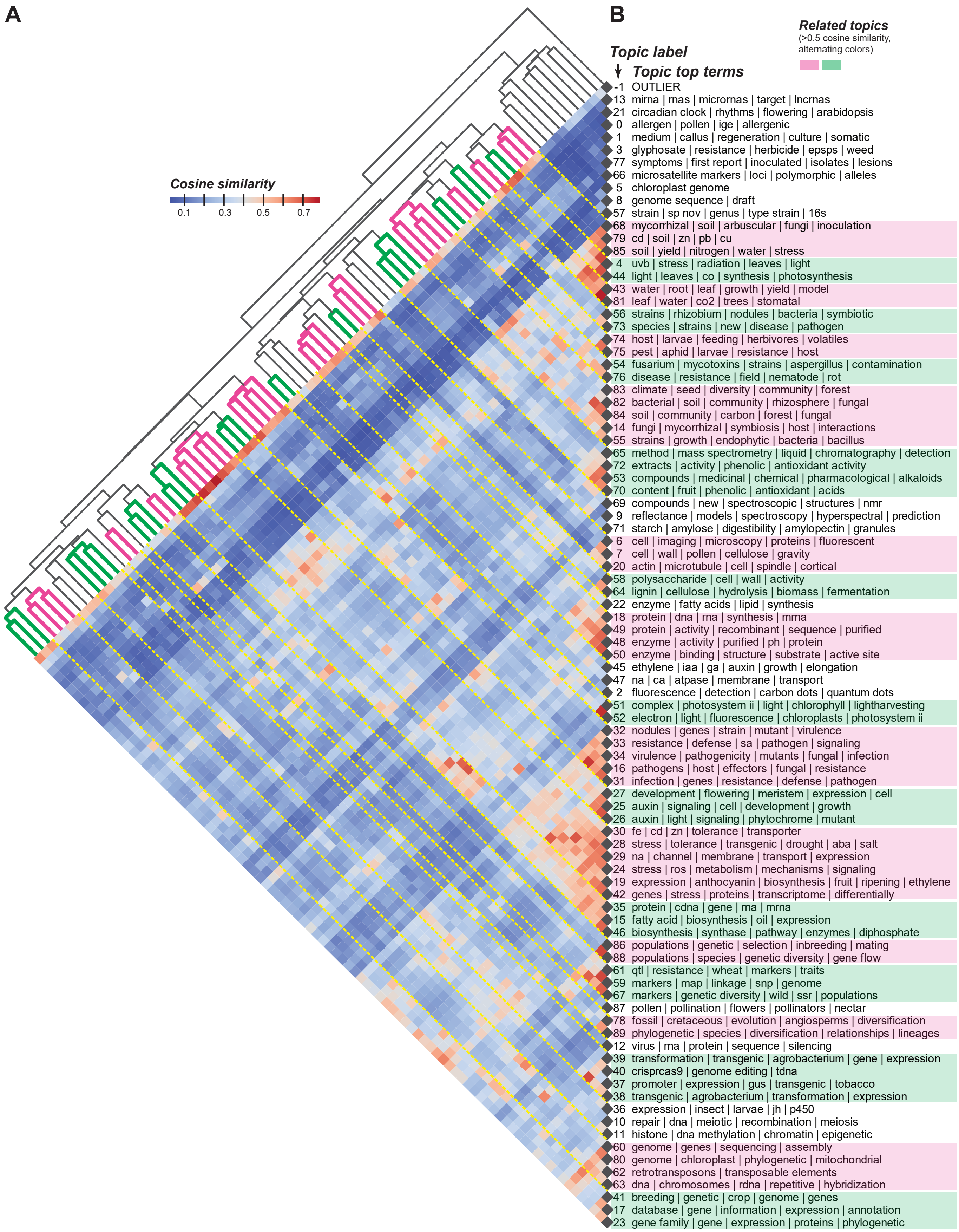
A
B
Cosine similarity
0.1
0.3
0.5
0.7
Related topics
(>0.5 cosine similarity,
alternating colors)
Topic label
Topic top terms
-1 OUTLIER
13 mirna | rnas | micrornas | target | lncrnas
21 circadian clock | rhythms | flowering | arabidopsis
0 allergen | pollen | ige | allergenic
1 medium | callus | regeneration | culture | somatic
3 glyphosate | resistance | herbicide | epsps | weed
77 symptoms | first report | inoculated | isolates | lesions
66 microsatellite markers | loci | polymorphic | alleles
5 chloroplast genome
8 genome sequence | draft
57 strain | sp nov | genus | type strain | 16s
68 mycorrhizal | soil | arbuscular | fungi | inoculation
79 cd | soil | zn | pb | cu
85 soil | yield | nitrogen | water | stress
4 uvb | stress | radiation | leaves | light
44 light | leaves | co | synthesis | photosynthesis
43 water | root | leaf | growth | yield | model
81 leaf | water | co2 | trees | stomatal
56 strains | rhizobium | nodules | bacteria | symbiotic
73 species | strains | new | disease | pathogen
74 host | larvae | feeding | herbivores | volatiles
75 pest | aphid | larvae | resistance | host
54 fusarium | mycotoxins | strains | aspergillus | contamination
76 disease | resistance | field | nematode | rot
83 climate | seed | diversity | community | forest
82 bacterial | soil | community | rhizosphere | fungal
84 soil | community | carbon | forest | fungal
14 fungi | mycorrhizal | symbiosis | host | interactions
55 strains | growth | endophytic | bacteria | bacillus
65 method | mass spectrometry | liquid | chromatography | detection
72 extracts | activity | phenolic | antioxidant activity
53 compounds | medicinal | chemical | pharmacological | alkaloids
70 content | fruit | phenolic | antioxidant | acids
69 compounds | new | spectroscopic | structures | nmr
9 reflectance | models | spectroscopy | hyperspectral | prediction
71 starch | amylose | digestibility | amylopectin | granules
6 cell | imaging | microscopy | proteins | fluorescent
7 cell | wall | pollen | cellulose | gravity
20 actin | microtubule | cell | spindle | cortical
58 polysaccharide | cell | wall | activity
64 lignin | cellulose | hydrolysis | biomass | fermentation
22 enzyme | fatty acids | lipid | synthesis
18 protein | dna | rna | synthesis | mrna
49 protein | activity | recombinant | sequence | purified
48 enzyme | activity | purified | ph | protein
50 enzyme | binding | structure | substrate | active site
45 ethylene | iaa | ga | auxin | growth | elongation
47 na | ca | atpase | membrane | transport
2 fluorescence | detection | carbon dots | quantum dots
51 complex | photosystem ii | light | chlorophyll | lightharvesting
52 electron | light | fluorescence | chloroplasts | photosystem ii
32 nodules | genes | strain | mutant | virulence
33 resistance | defense | sa | pathogen | signaling
34 virulence | pathogenicity | mutants | fungal | infection
16 pathogens | host | effectors | fungal | resistance
31 infection | genes | resistance | defense | pathogen
27 development | flowering | meristem | expression | cell
25 auxin | signaling | cell | development | growth
26 auxin | light | signaling | phytochrome | mutant
30 fe | cd | zn | tolerance | transporter
28 stress | tolerance | transgenic | drought | aba | salt
29 na | channel | membrane | transport | expression
24 stress | ros | metabolism | mechanisms | signaling
19 expression | anthocyanin | biosynthesis | fruit | ripening | ethylene
42 genes | stress | proteins | transcriptome | differentially
35 protein | cdna | gene | rna | mrna
15 fatty acid | biosynthesis | oil | expression
46 biosynthesis | synthase | pathway | enzymes | diphosphate
86 populations | genetic | selection | inbreeding | mating
88 populations | species | genetic diversity | gene flow
61 qtl | resistance | wheat | markers | traits
59 markers | map | linkage | snp | genome
67 markers | genetic diversity | wild | ssr | populations
87 pollen | pollination | flowers | pollinators | nectar
78 fossil | cretaceous | evolution | angiosperms | diversification
89 phylogenetic | species | diversification | relationships | lineages
12 virus | rna | protein | sequence | silencing
39 transformation | transgenic | agrobacterium | gene | expression
40 crisprcas9 | genome editing | tdna
37 promoter | expression | gus | transgenic | tobacco
38 transgenic | agrobacterium | transformation | expression
36 expression | insect | larvae | jh | p450
10 repair | dna | meiotic | recombination | meiosis
11 histone | dna methylation | chromatin | epigenetic
60 genome | genes | sequencing | assembly
80 genome | chloroplast | phylogenetic | mitochondrial
62 retrotransposons | transposable elements
63 dna | chromosomes | rdna | repetitive | hybridization
41 breeding | genetic | crop | genome | genes
17 database | gene | information | expression | annotation
23 gene family | gene | expression | proteins | phylogenetic
