# Supplementary material for: Assessing the evolution of research topics in a biological field using plant science as an example
Source: PLoS Biol. 2024 May 23;22(5):e3002612. doi: 10.1371/journal.pbio.3002612 (PMC11115244; doi:10.1371/journal.pbio.3002612)
Supplement: S4 Data — For a topic T, records in the UMAP graph are colored red and records not in T are colored gray. (PDF) [file pbio.3002612.s013.pdf]

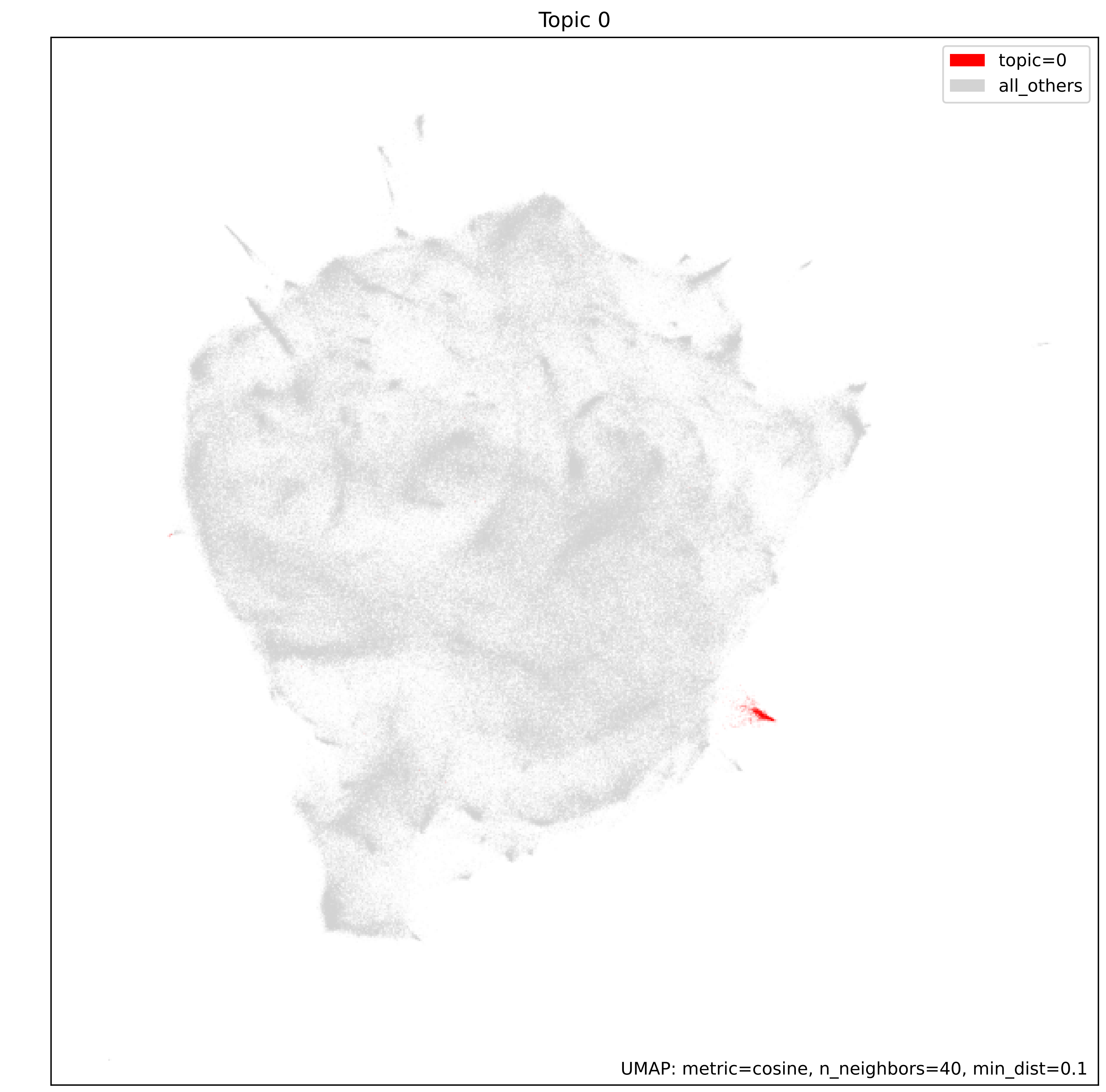
Topic 0
topic=0
all_others
UMAP: metric=cosine, n_neighbors=40, min_dist=0.1

Topic 1

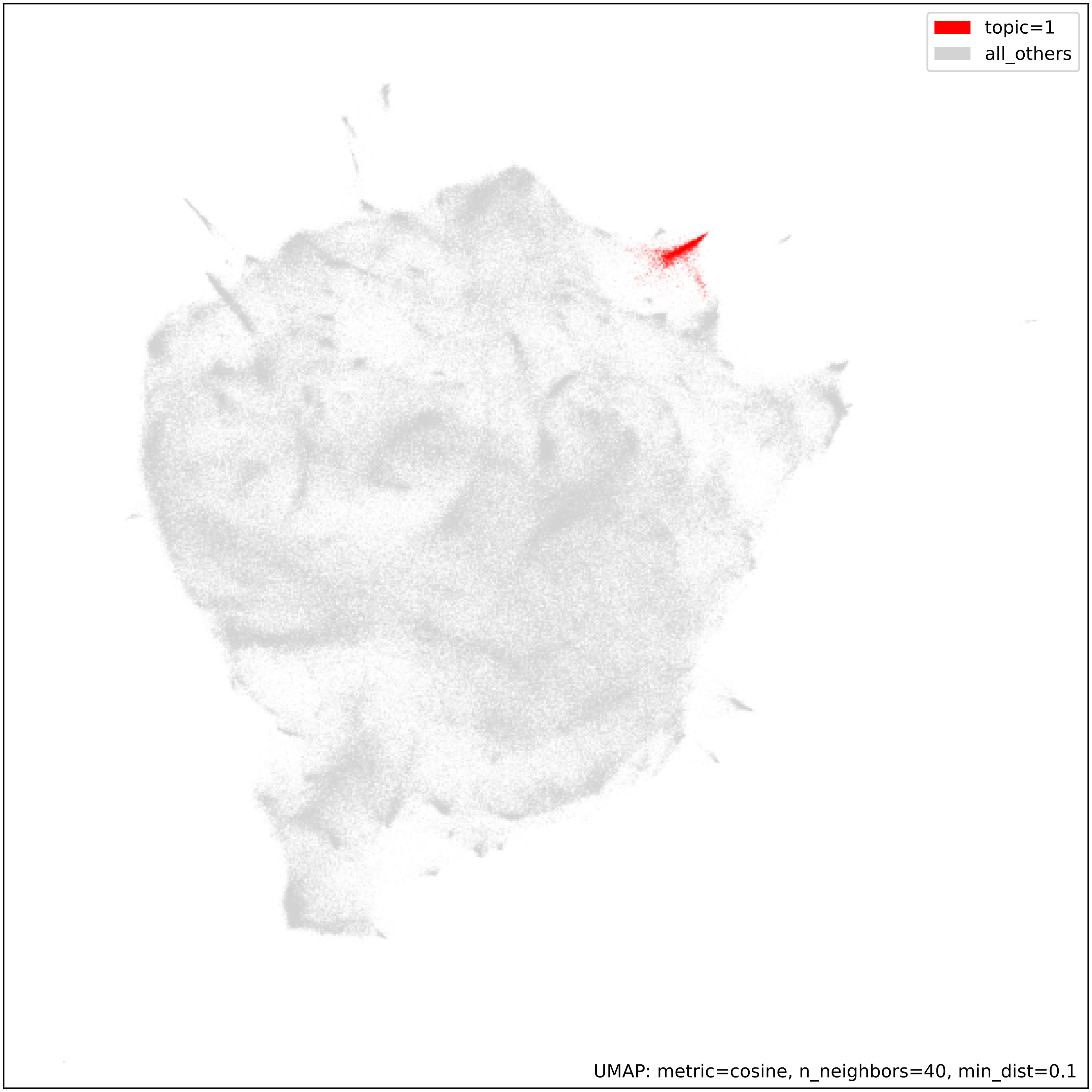
topic=1
all_others
UMAP: metric=cosine, n_neighbors=40, min_dist=0.1

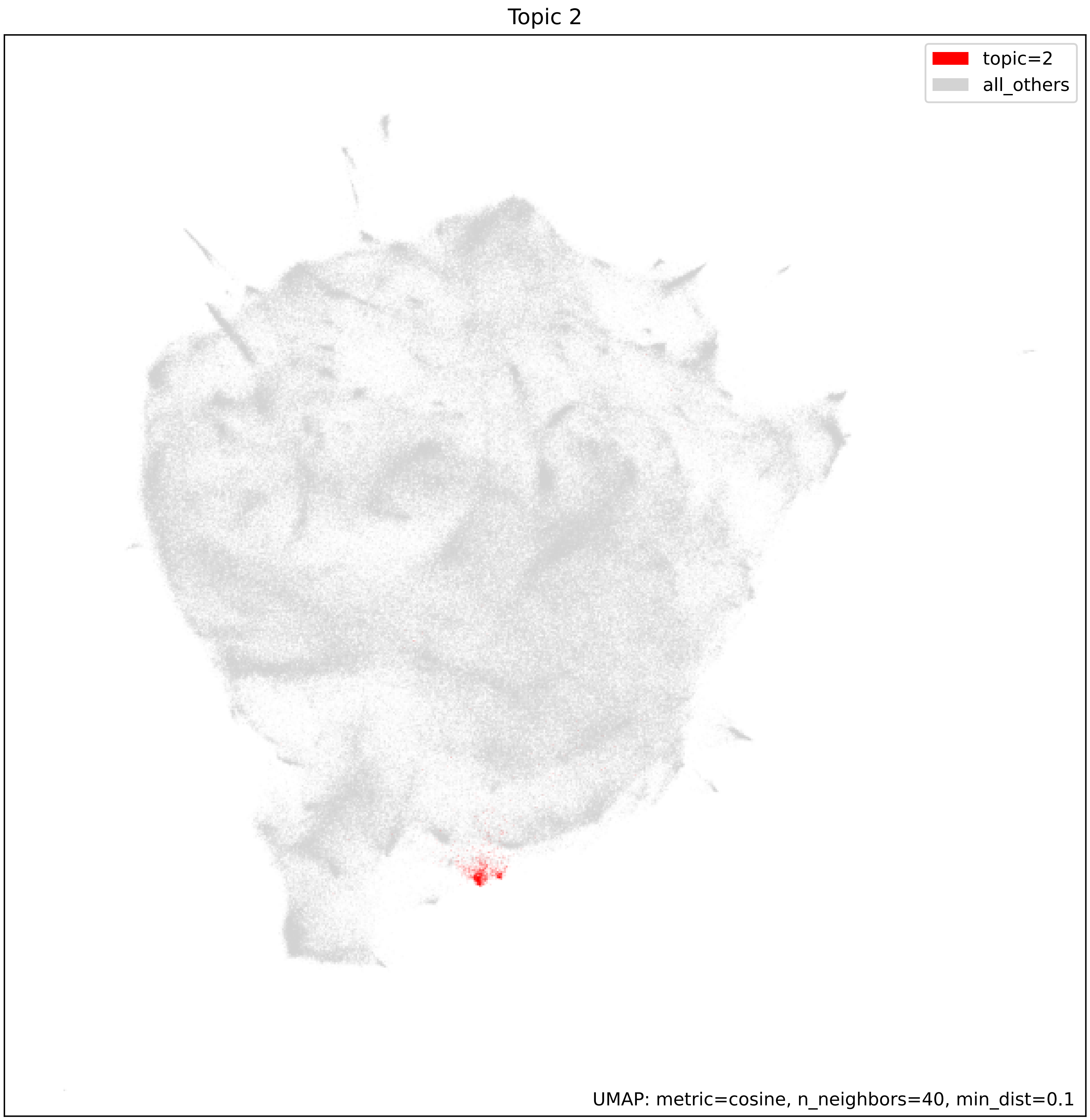
Topic 2
topic=2
all_others
UMAP: metric=cosine, n_neighbors=40, min_dist=0.1

Topic 3

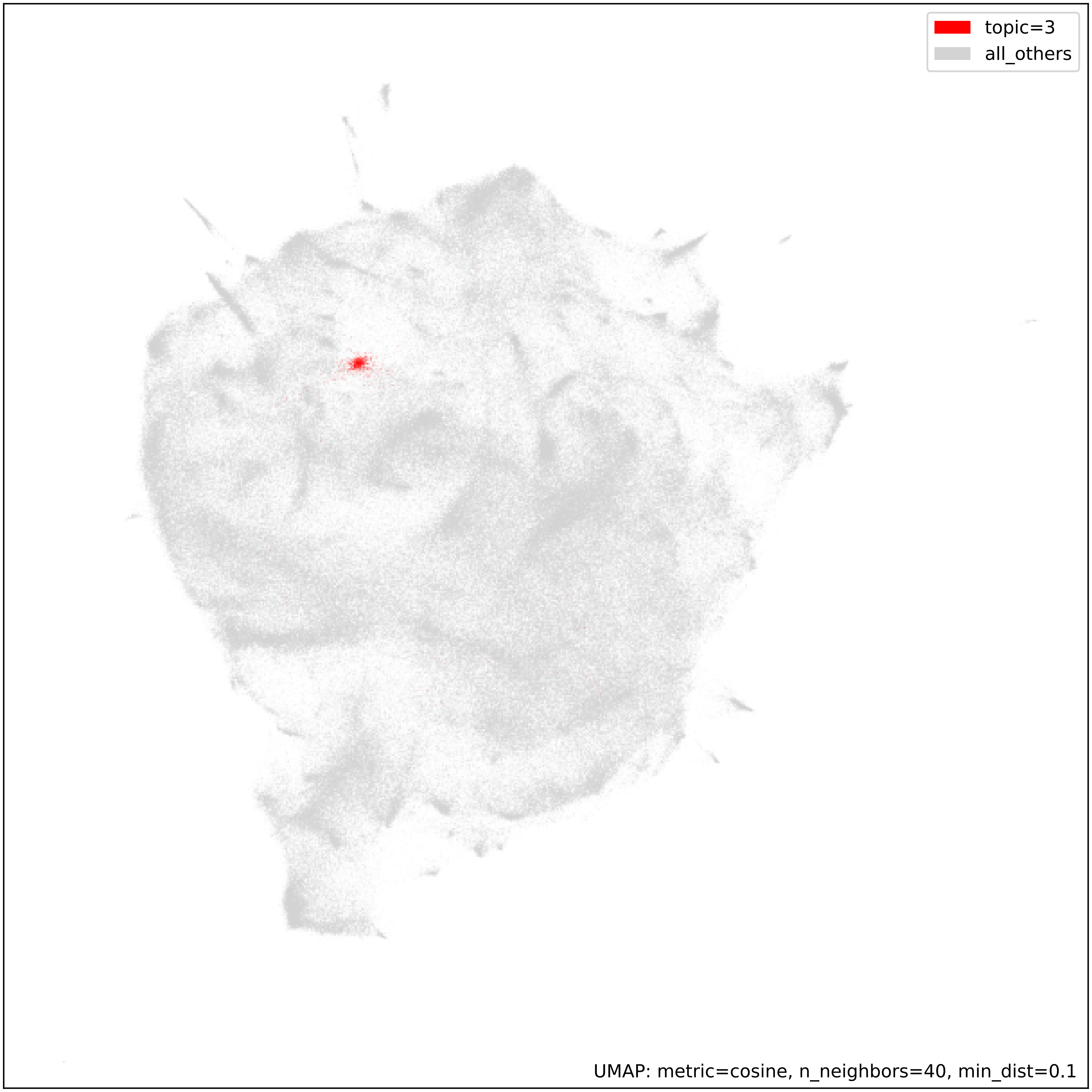
topic=3
all_others
UMAP: metric=cosine, n_neighbors=40, min_dist=0.1

Topic 4

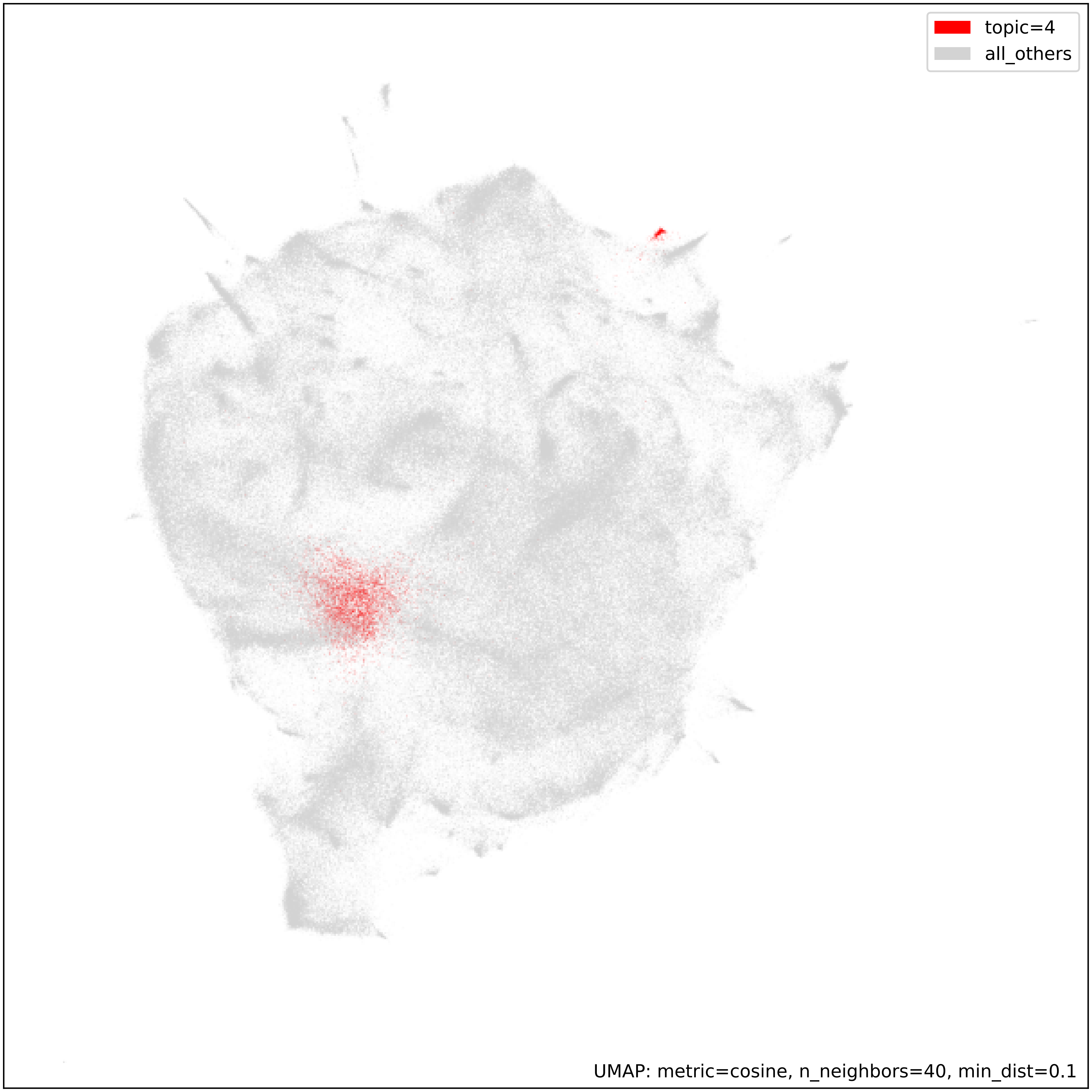

topic=4
all_others
UMAP: metric=cosine, n_neighbors=40, min_dist=0.1

Topic 5

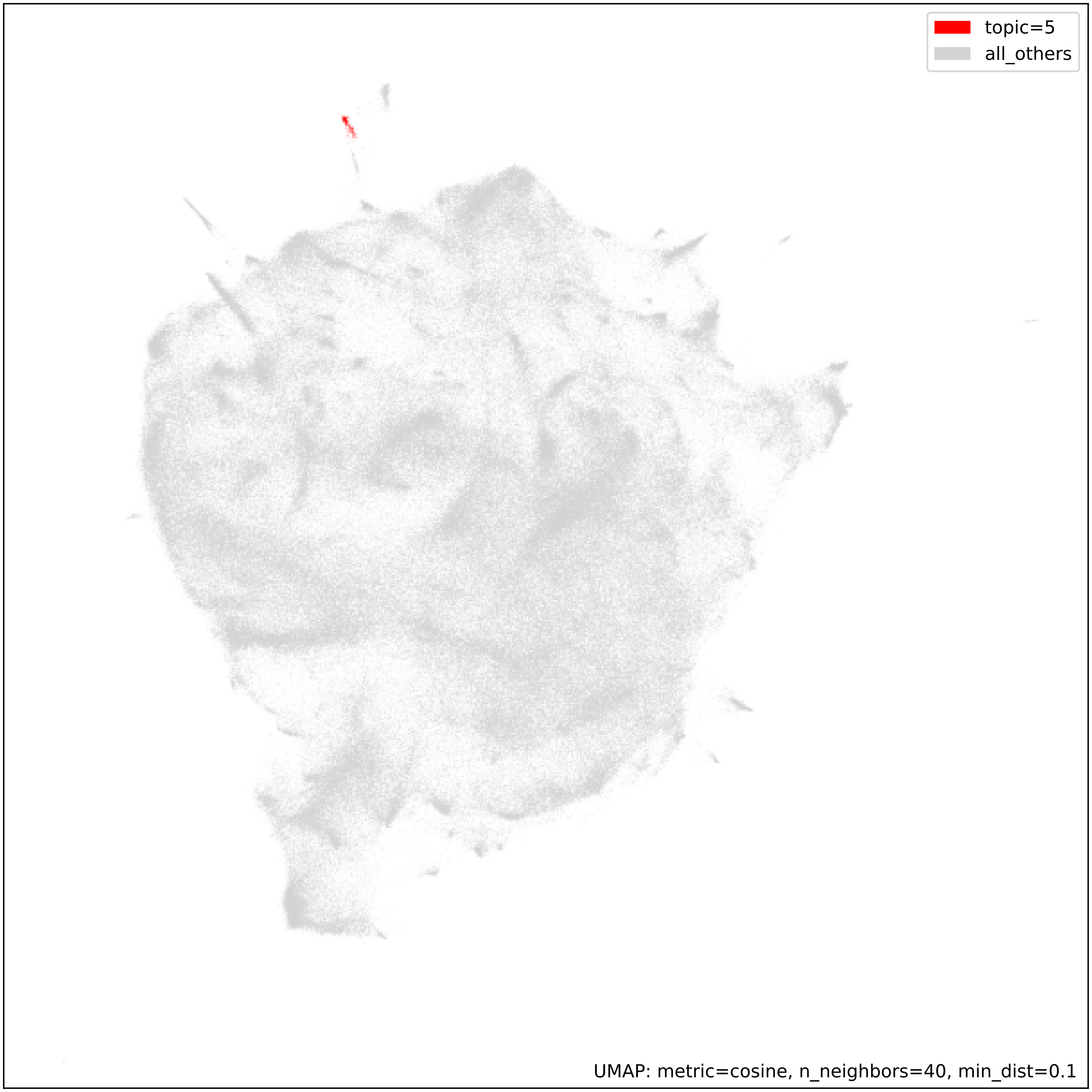
topic=5
all_others
UMAP: metric=cosine, n_neighbors=40, min_dist=0.1

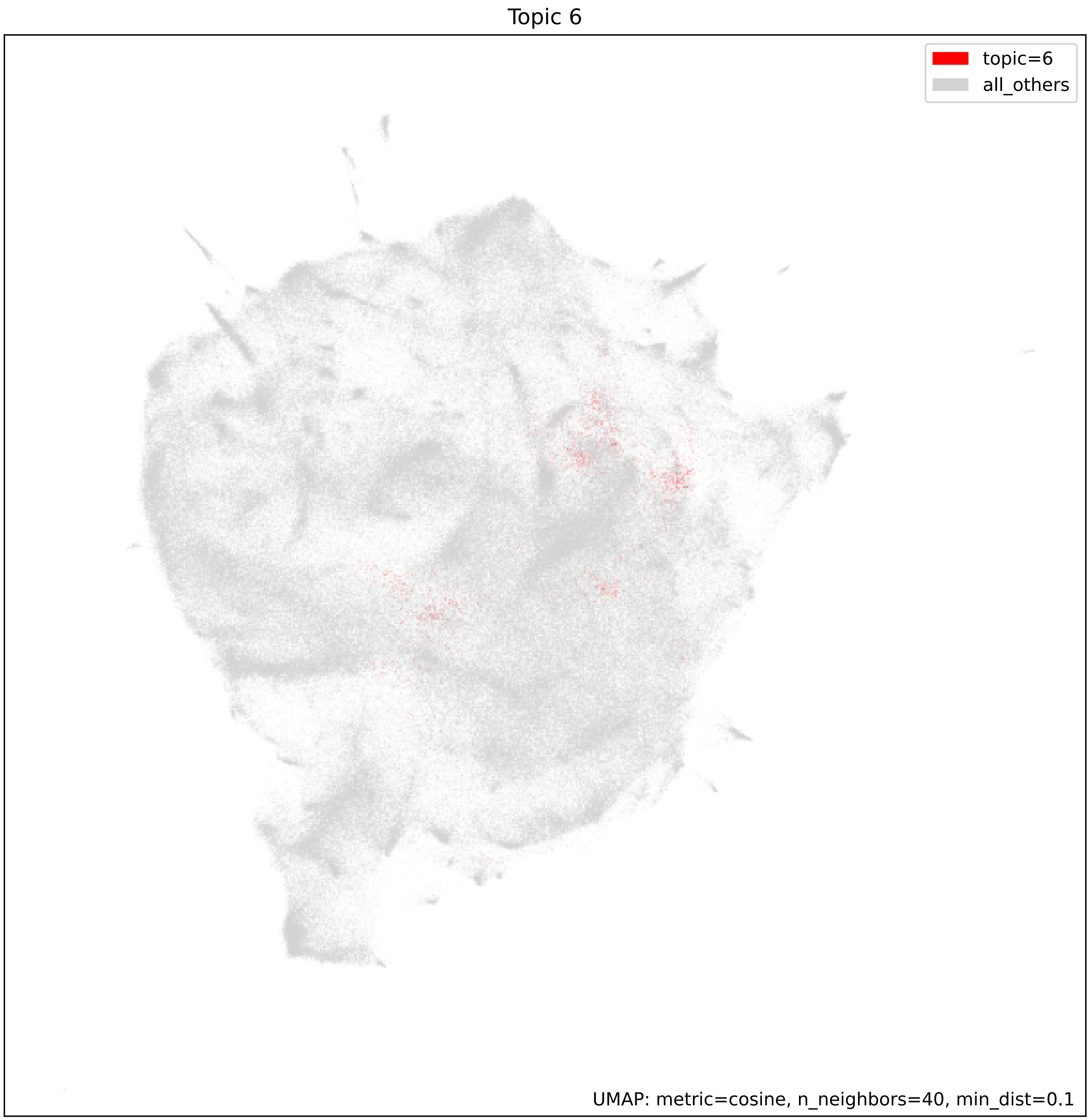
Topic 6
topic=6
all_others
UMAP: metric=cosine, n_neighbors=40, min_dist=0.1

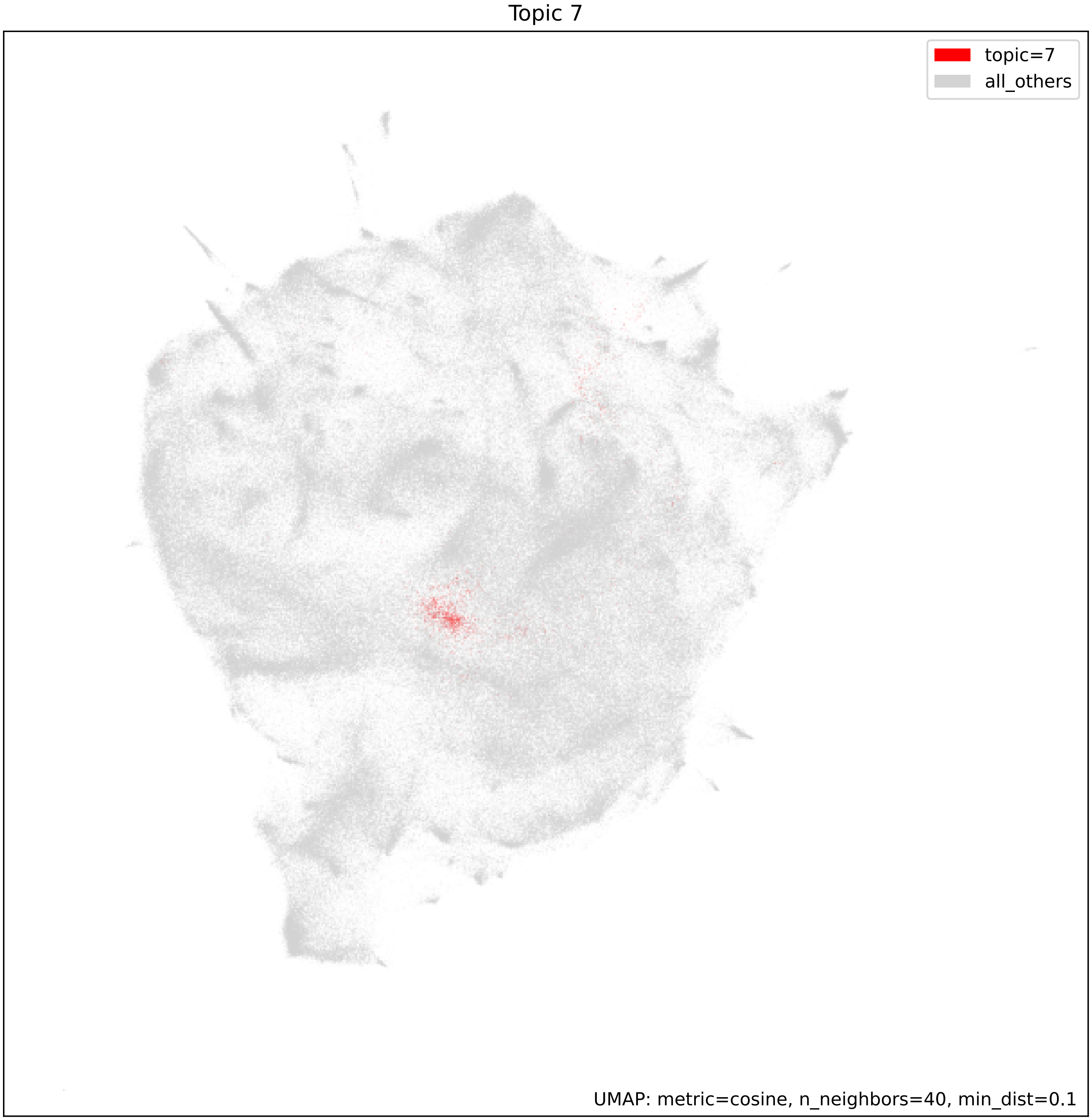
Topic 7
topic=7
all_others
UMAP: metric=cosine, n_neighbors=40, min_dist=0.1

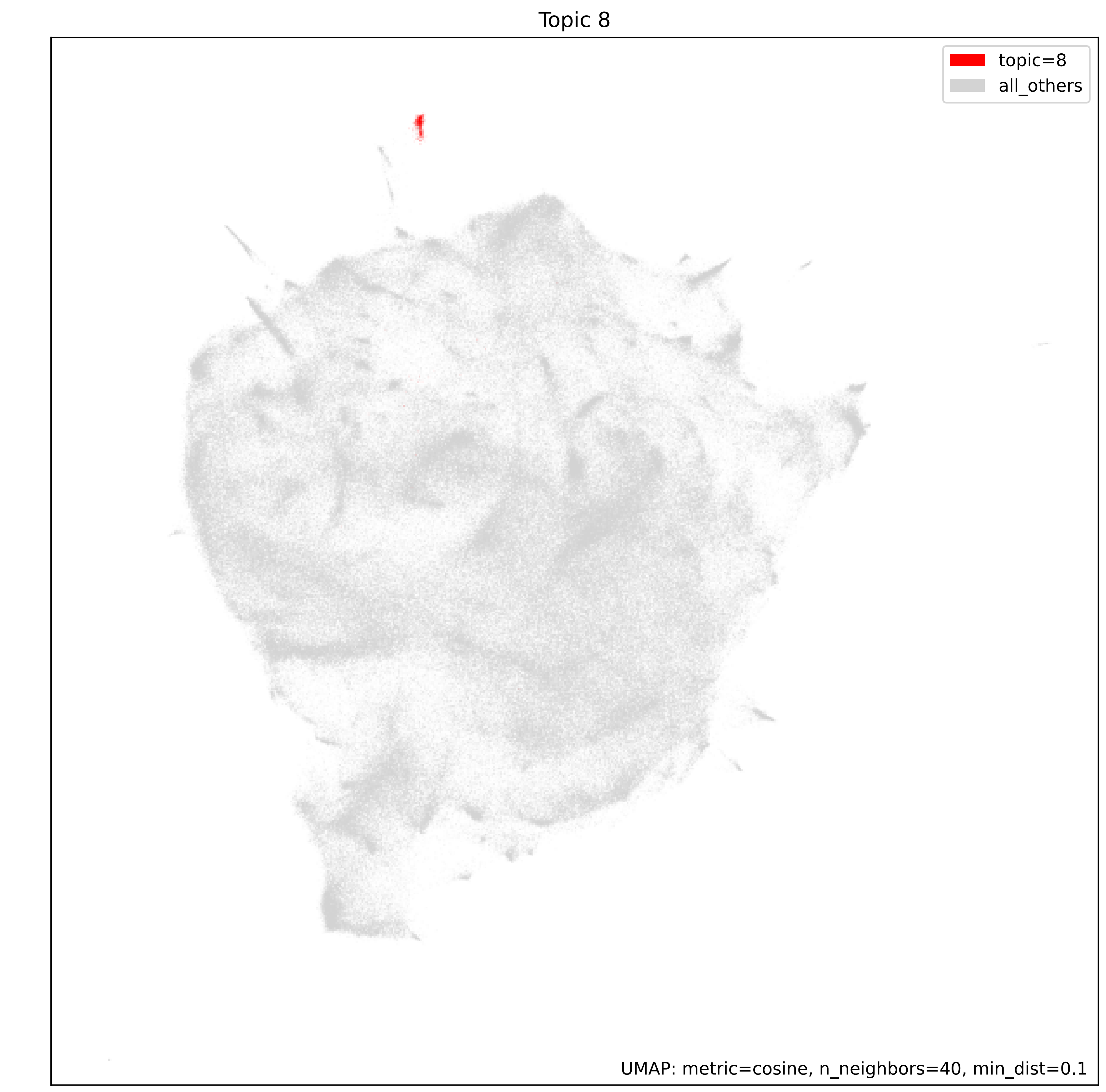

Topic 8
topic=8
all_others
UMAP: metric=cosine, n_neighbors=40, min_dist=0.1

Topic 9

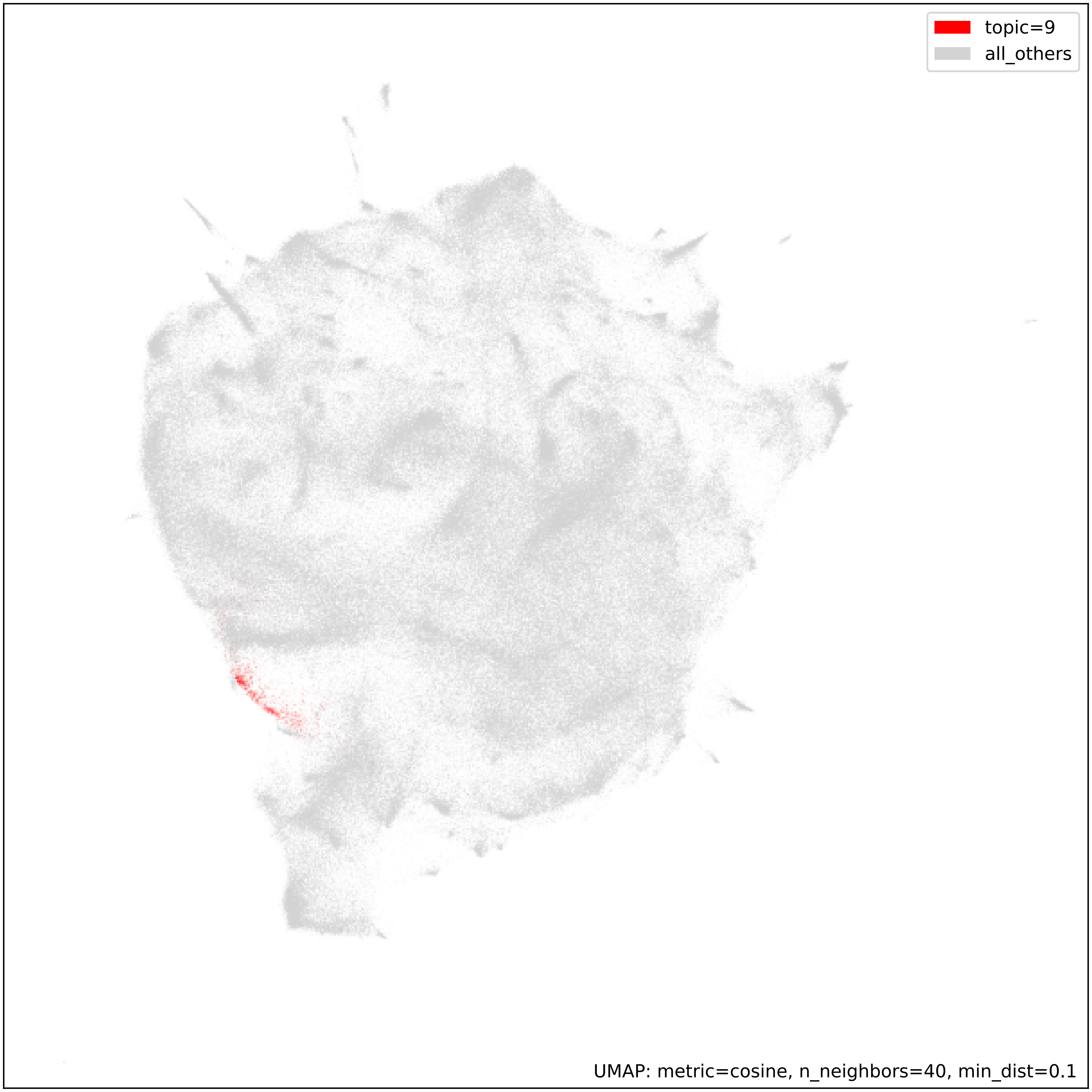
topic=9
all_others
UMAP: metric=cosine, n_neighbors=40, min_dist=0.1

Topic 10

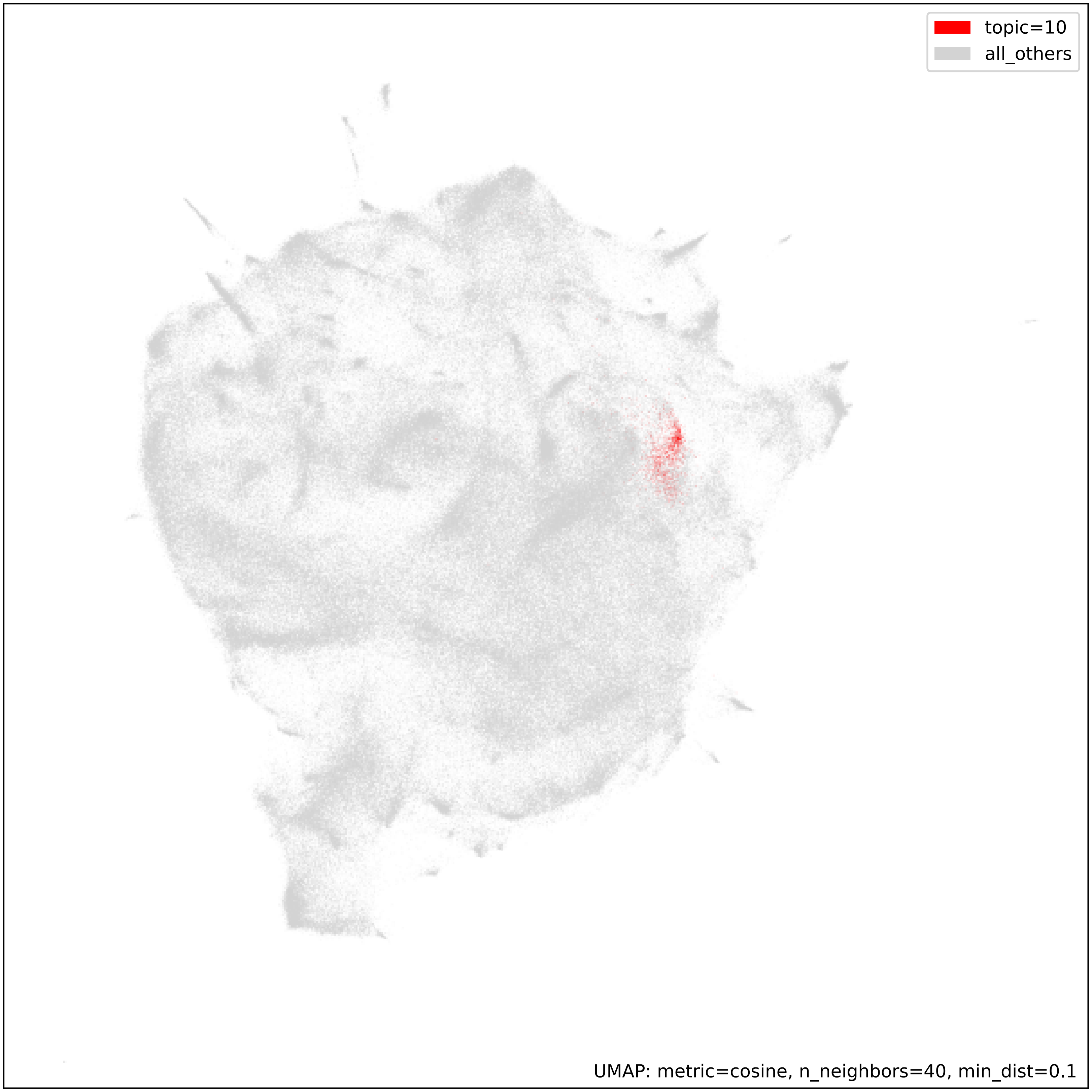
topic=10
all_others
UMAP: metric=cosine, n_neighbors=40, min_dist=0.1

Topic 11

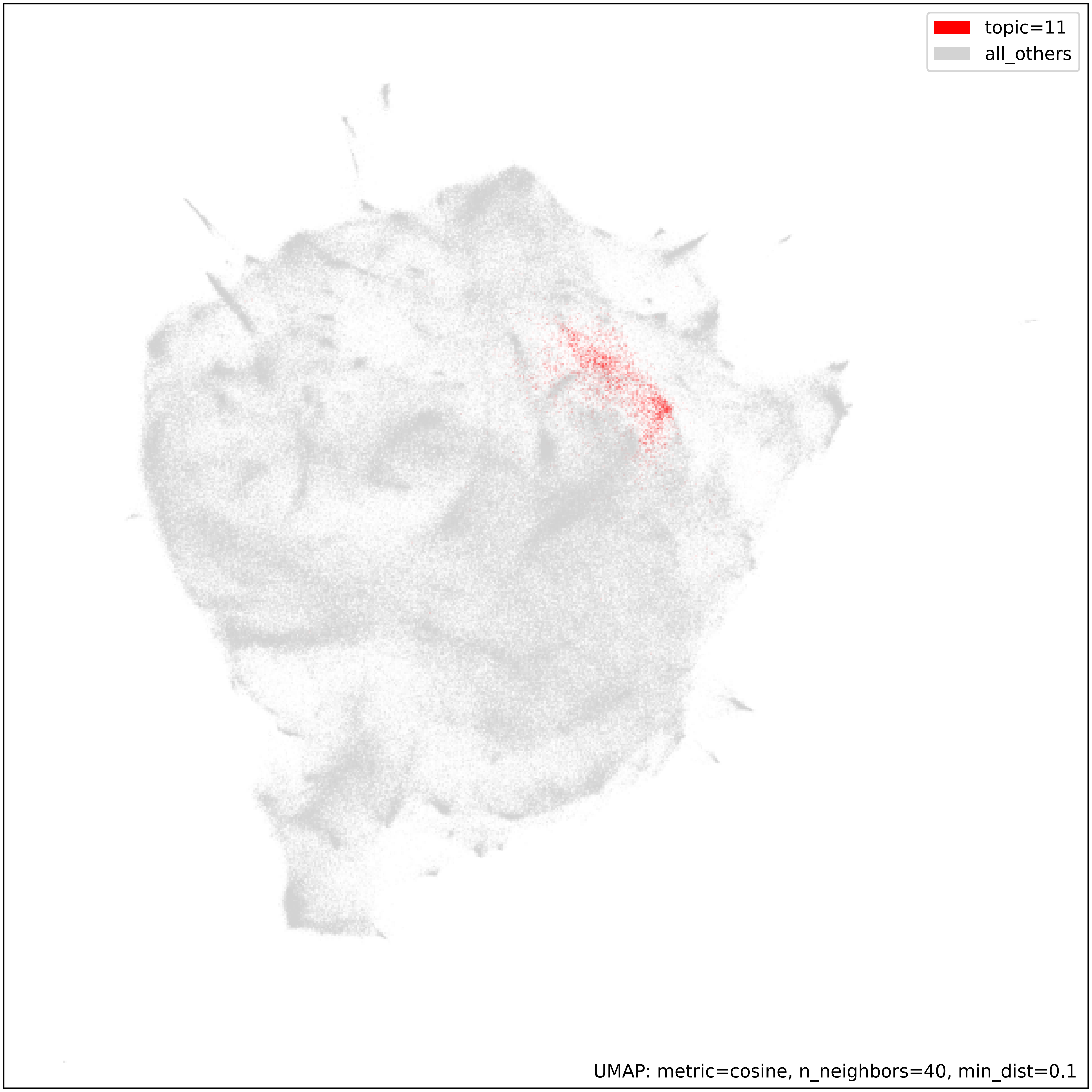

topic=11
all_others
UMAP: metric=cosine, n_neighbors=40, min_dist=0.1

Topic 12

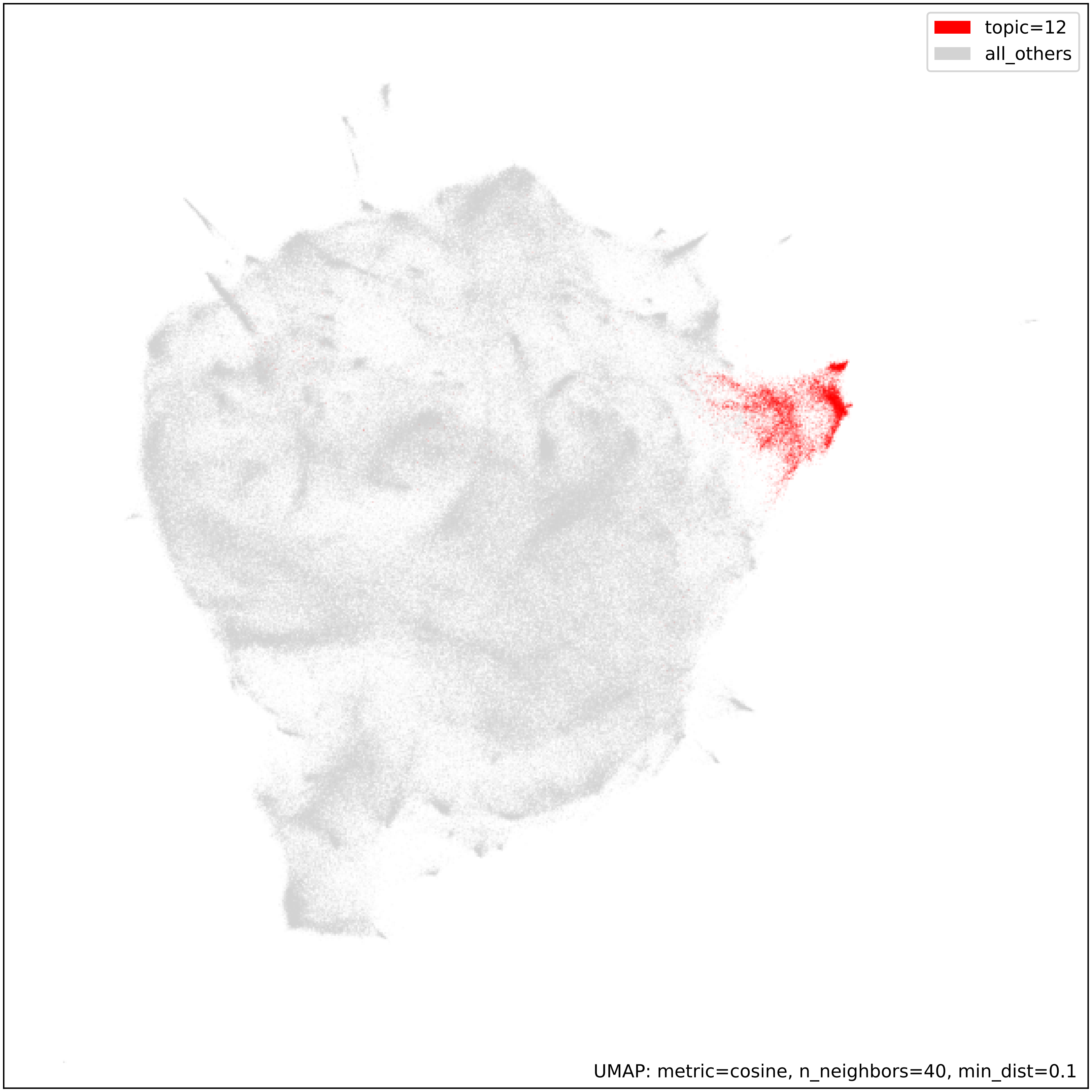
topic=12
all_others
UMAP: metric=cosine, n_neighbors=40, min_dist=0.1

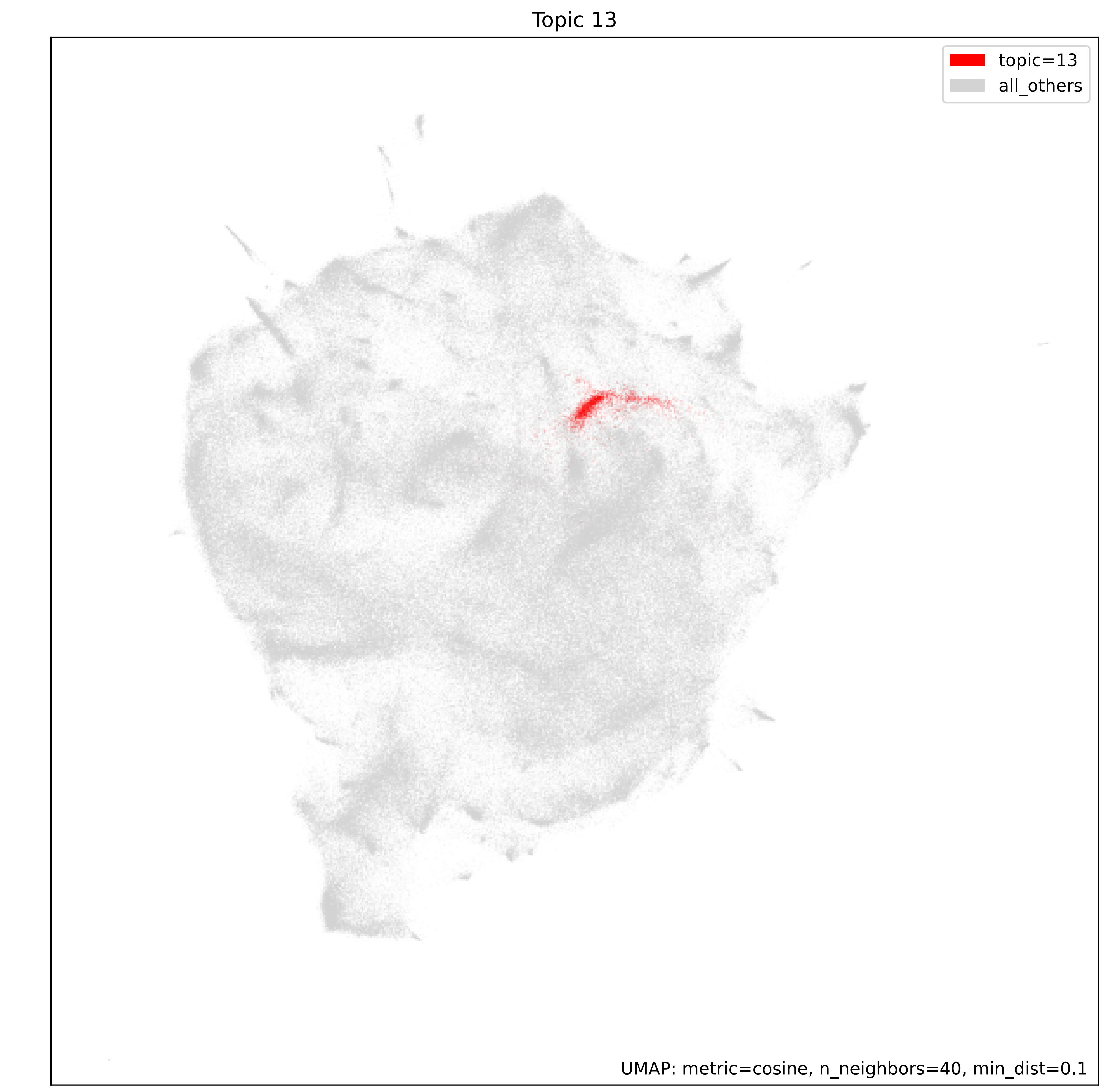
Topic 13
topic=13
all_others
UMAP: metric=cosine, n_neighbors=40, min_dist=0.1

Topic 14

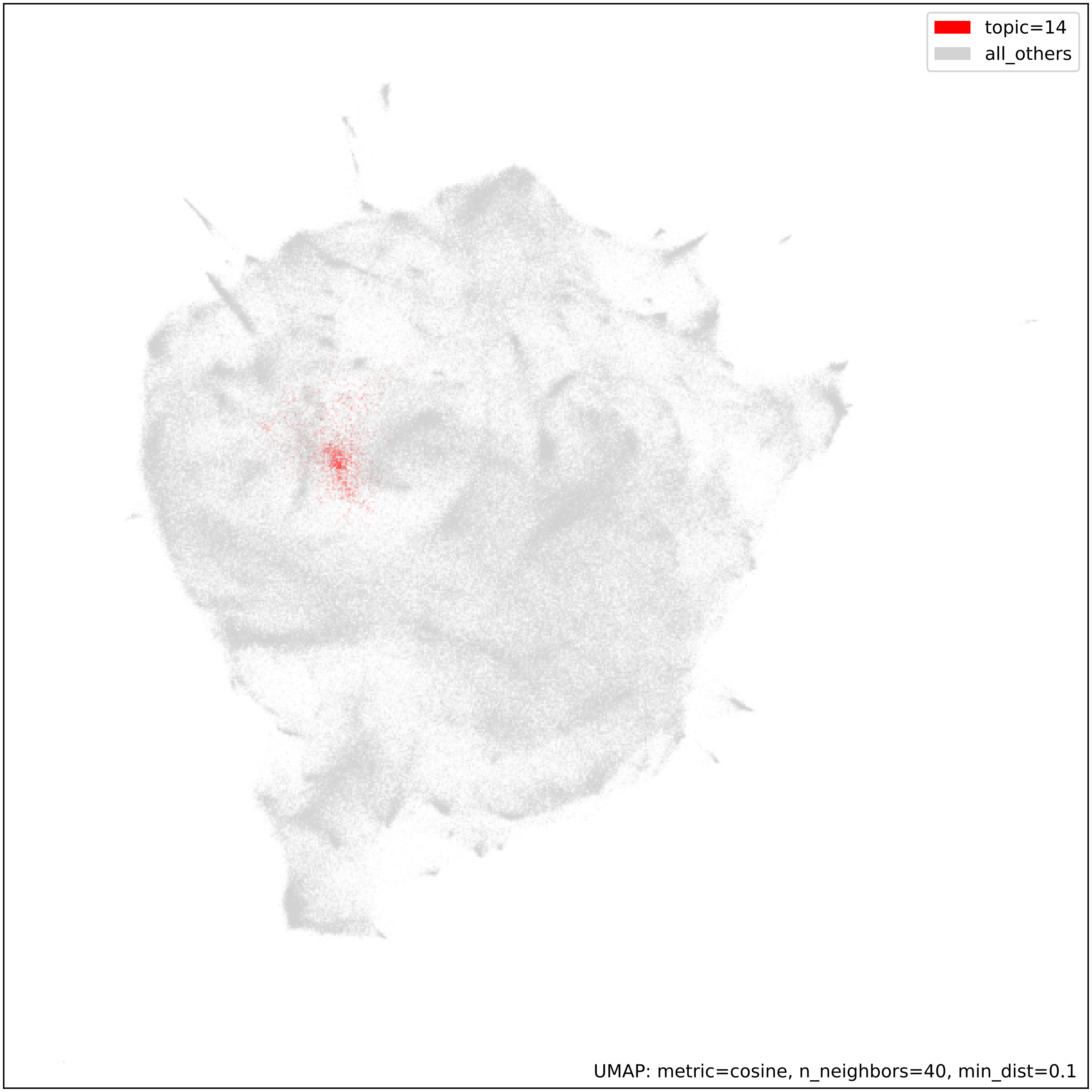
topic=14
all_others
UMAP: metric=cosine, n_neighbors=40, min_dist=0.1

Topic 15

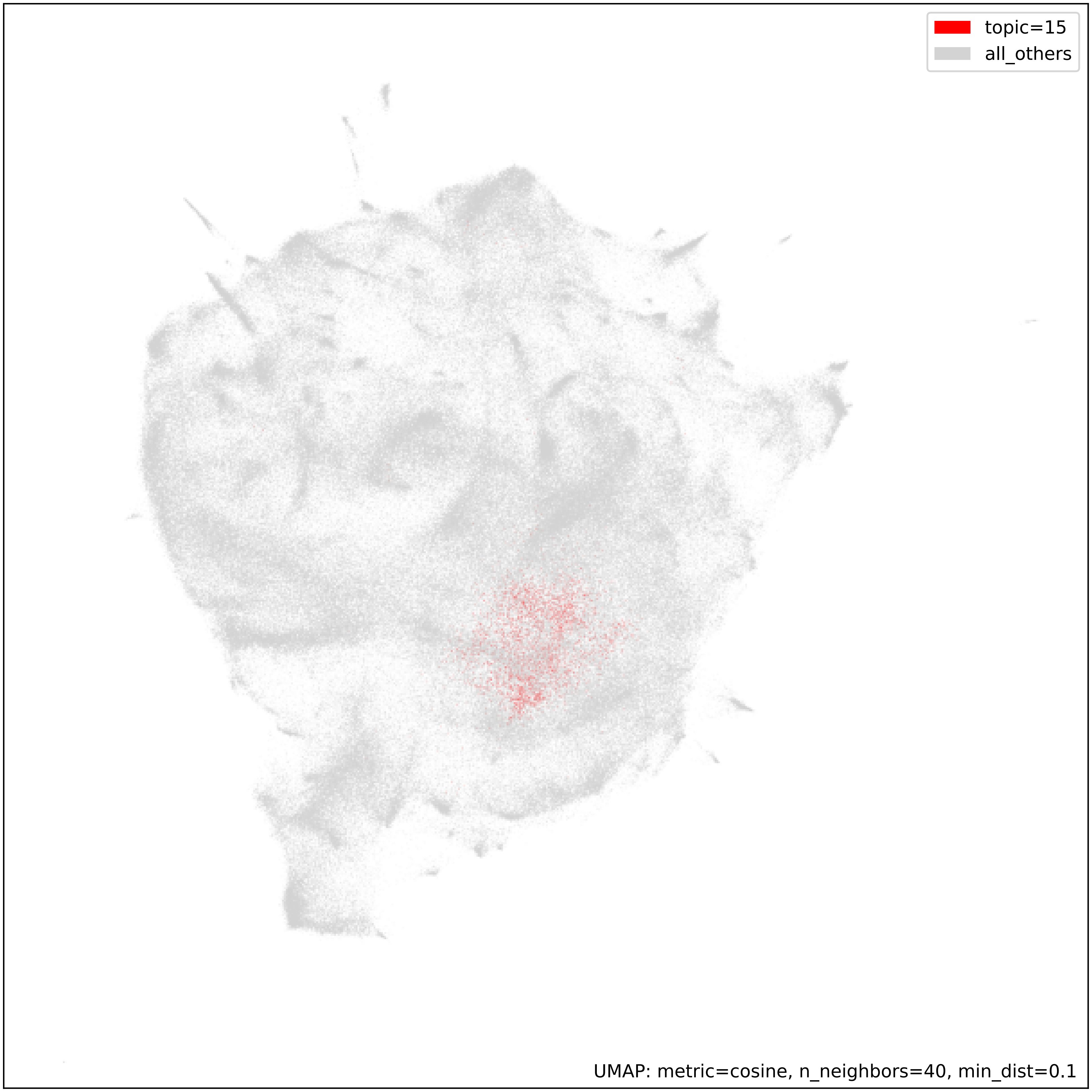

topic=15
all_others
UMAP: metric=cosine, n_neighbors=40, min_dist=0.1

Topic 16

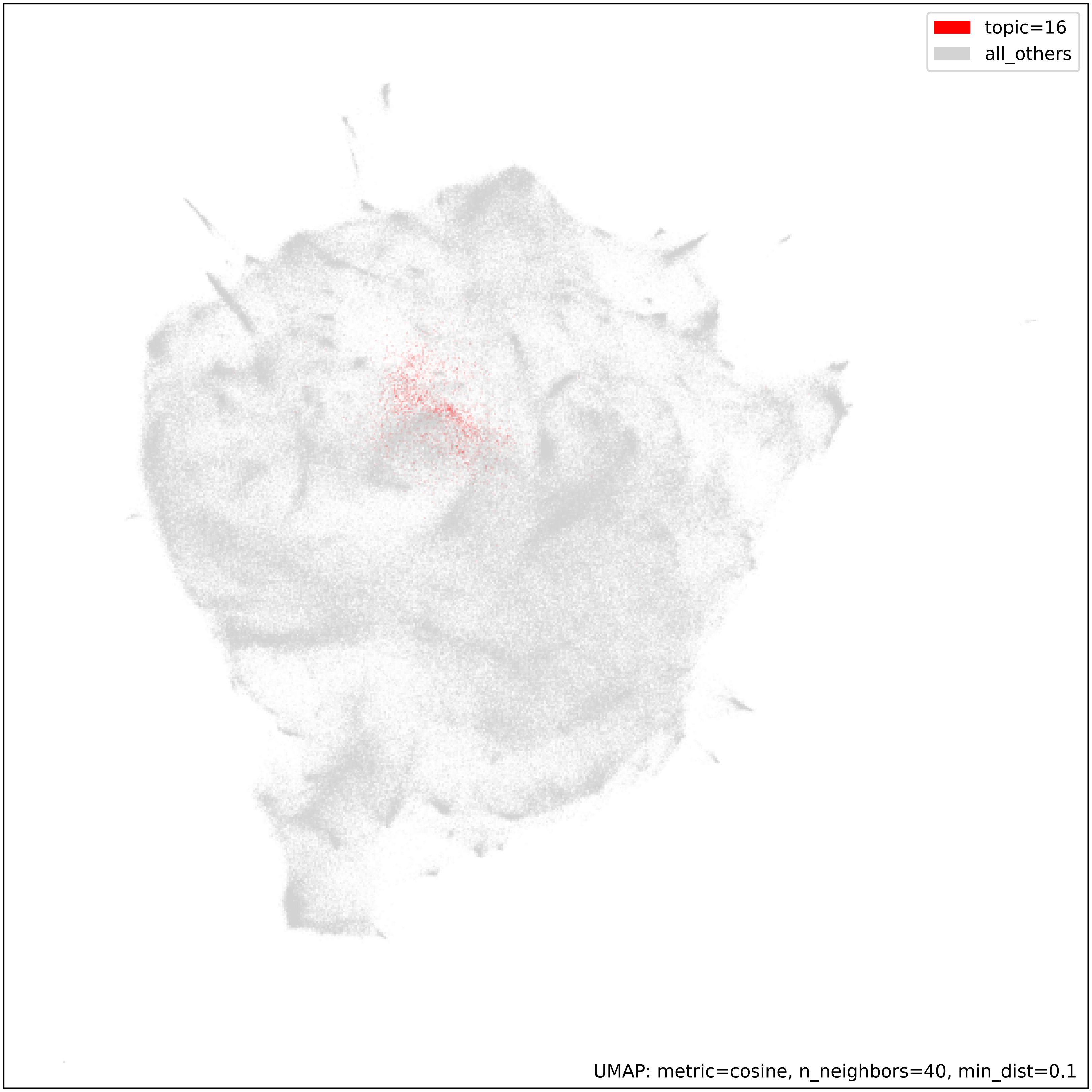

topic=16
all_others
UMAP: metric=cosine, n_neighbors=40, min_dist=0.1

Topic 17

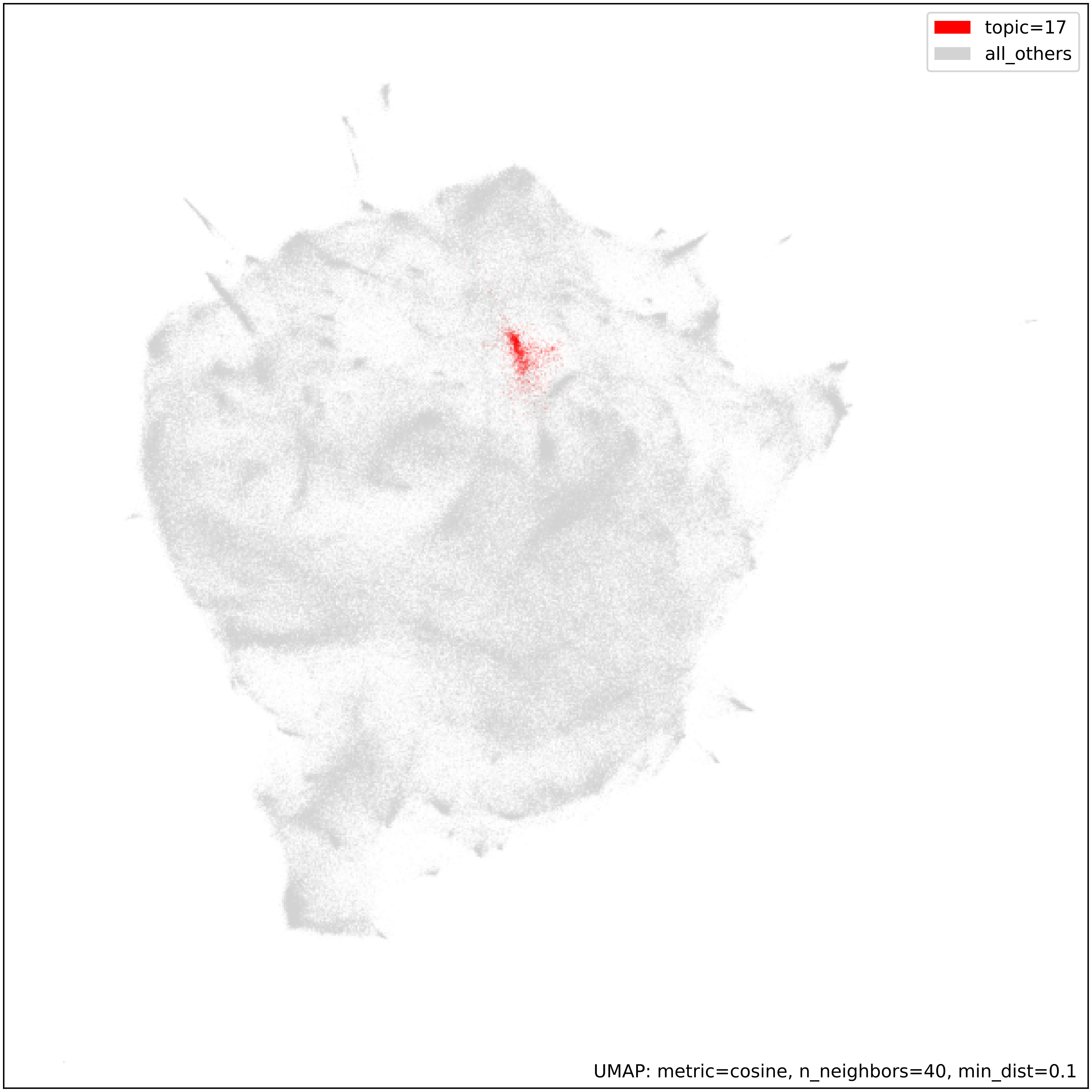

topic=17
all_others
UMAP: metric=cosine, n_neighbors=40, min_dist=0.1

Topic 18

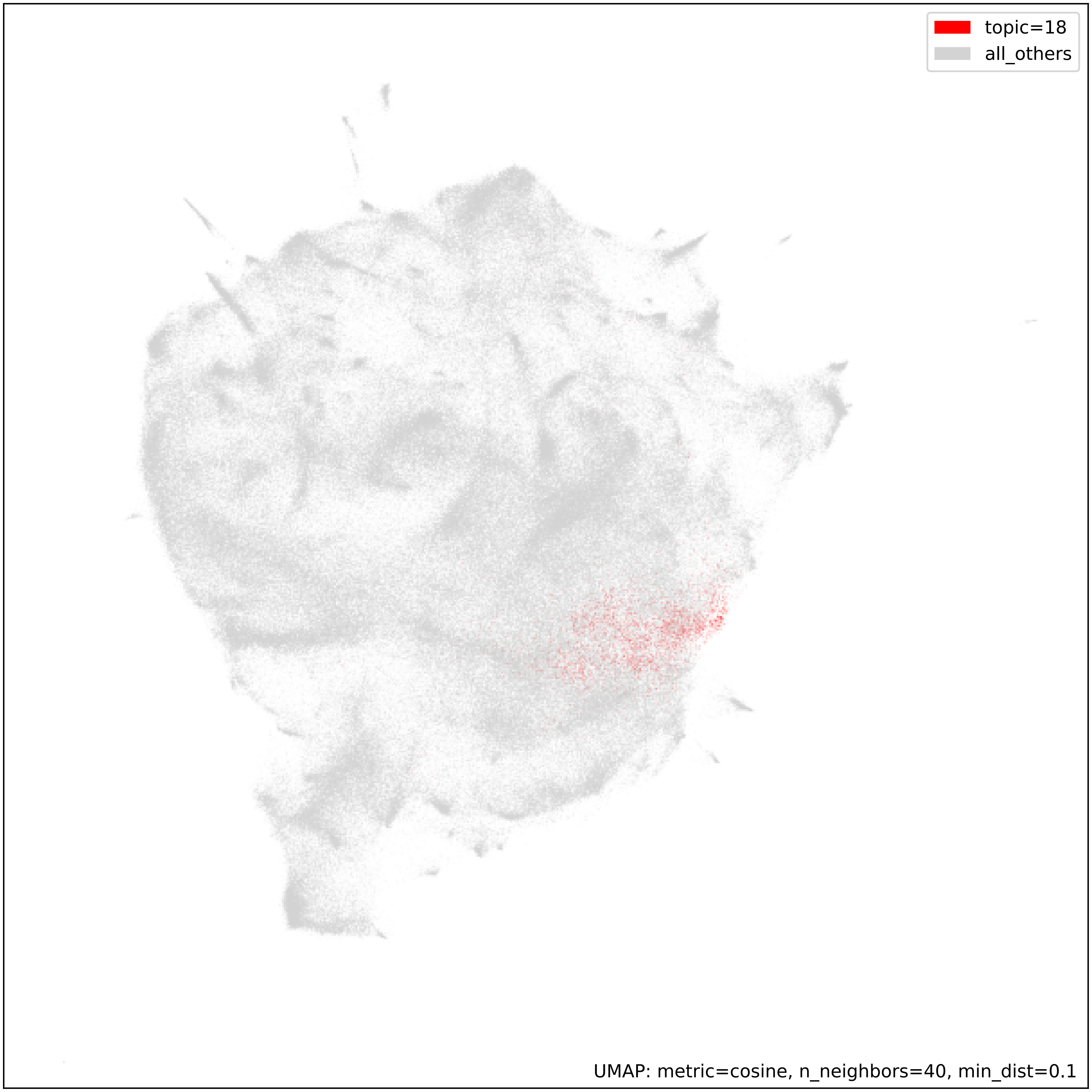

topic=18
all_others
UMAP: metric=cosine, n_neighbors=40, min_dist=0.1

Topic 19

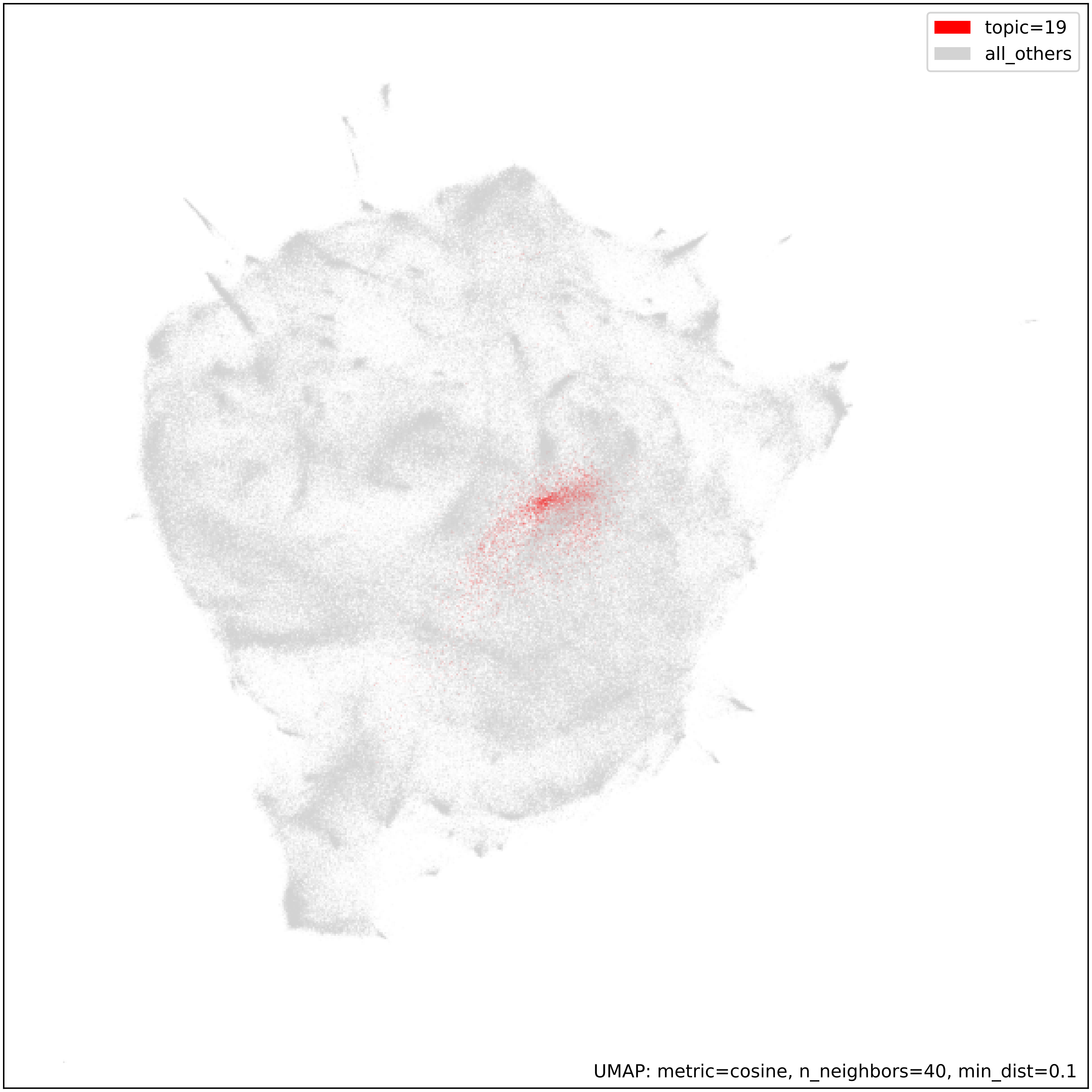
topic=19
all_others
UMAP: metric=cosine, n_neighbors=40, min_dist=0.1

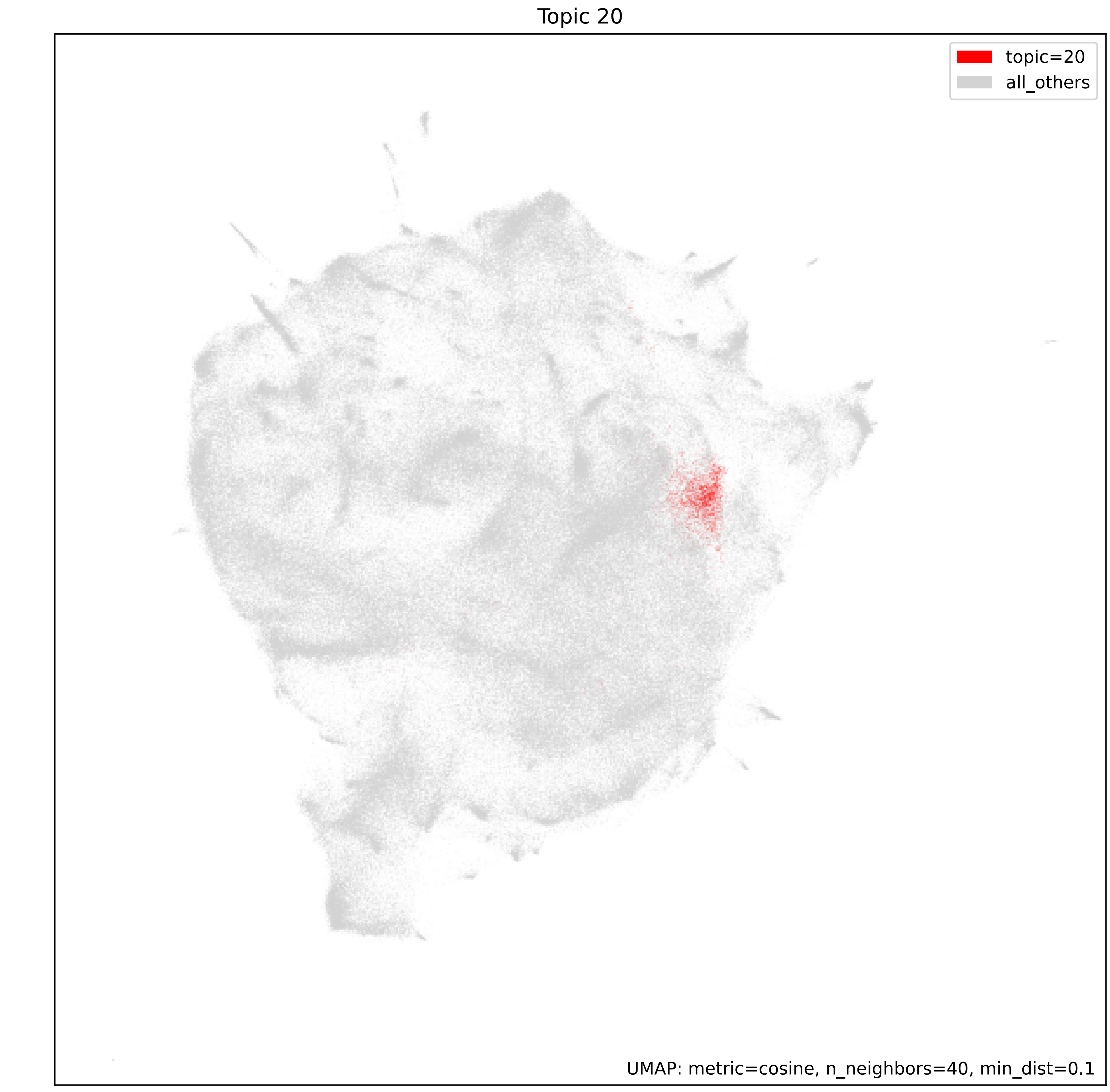
Topic 20
topic=20
all_others
UMAP: metric=cosine, n_neighbors=40, min_dist=0.1

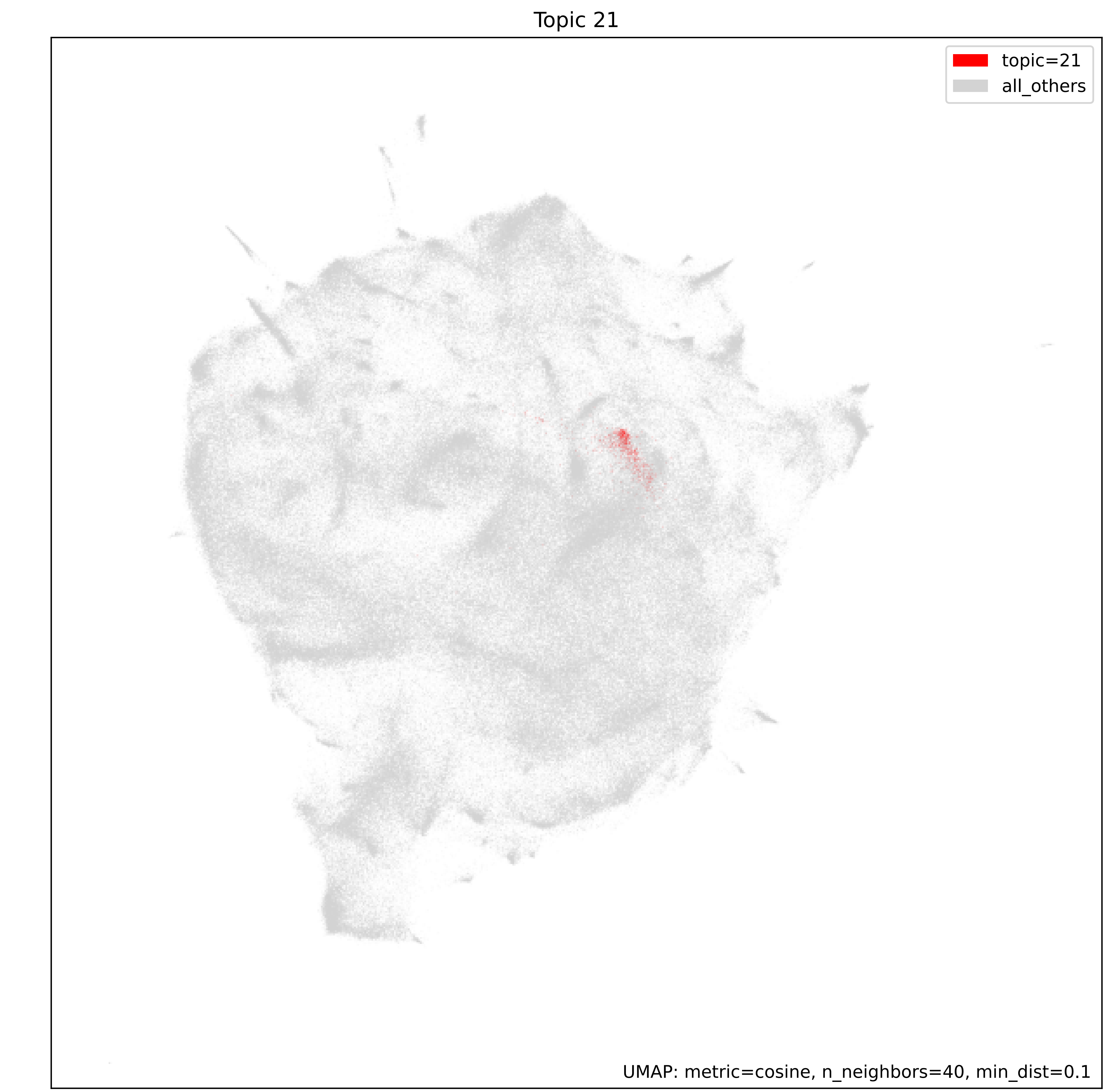
Topic 21
topic=21
all_others
UMAP: metric=cosine, n_neighbors=40, min_dist=0.1

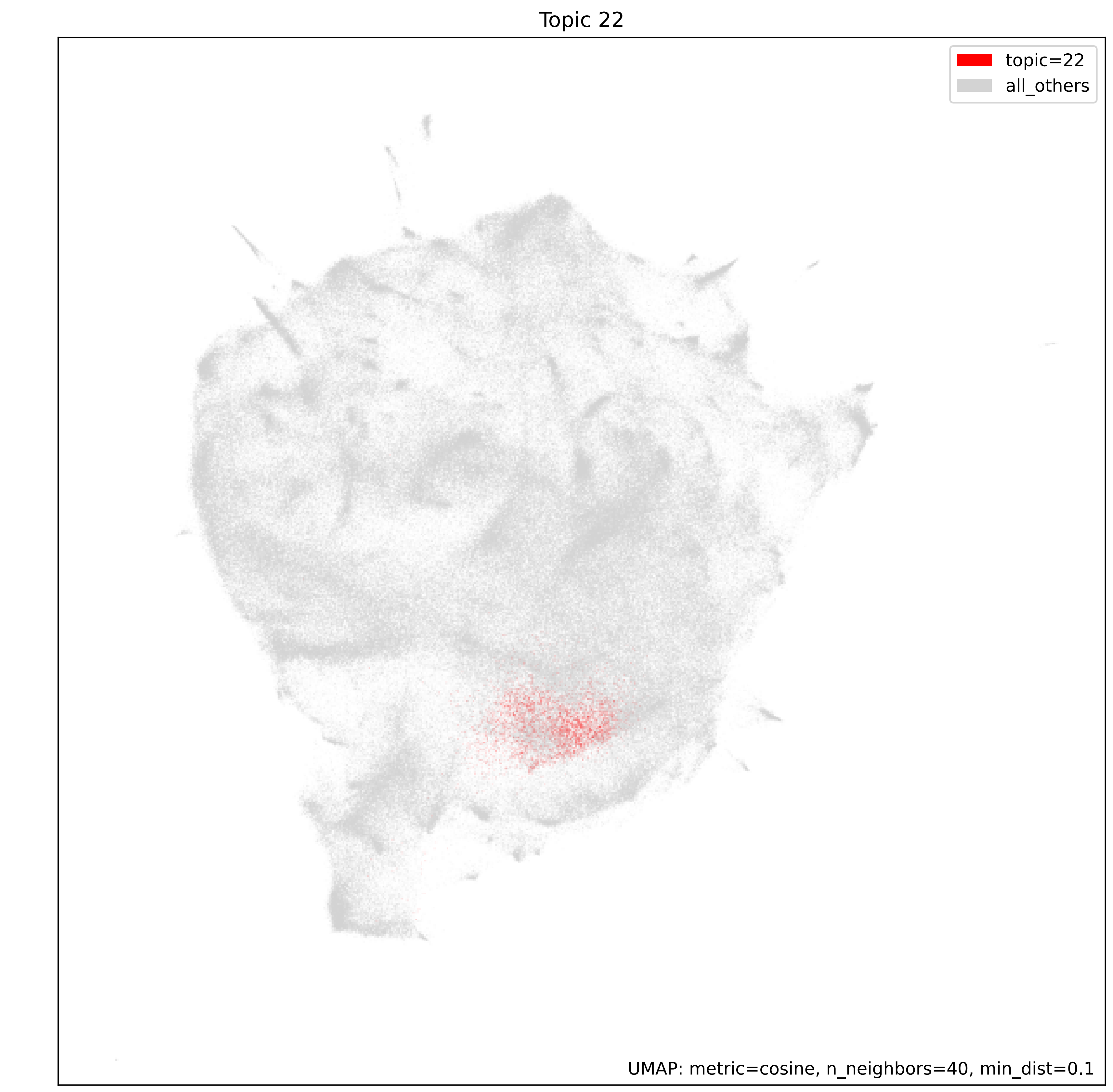
Topic 22
topic=22
all_others
UMAP: metric=cosine, n_neighbors=40, min_dist=0.1

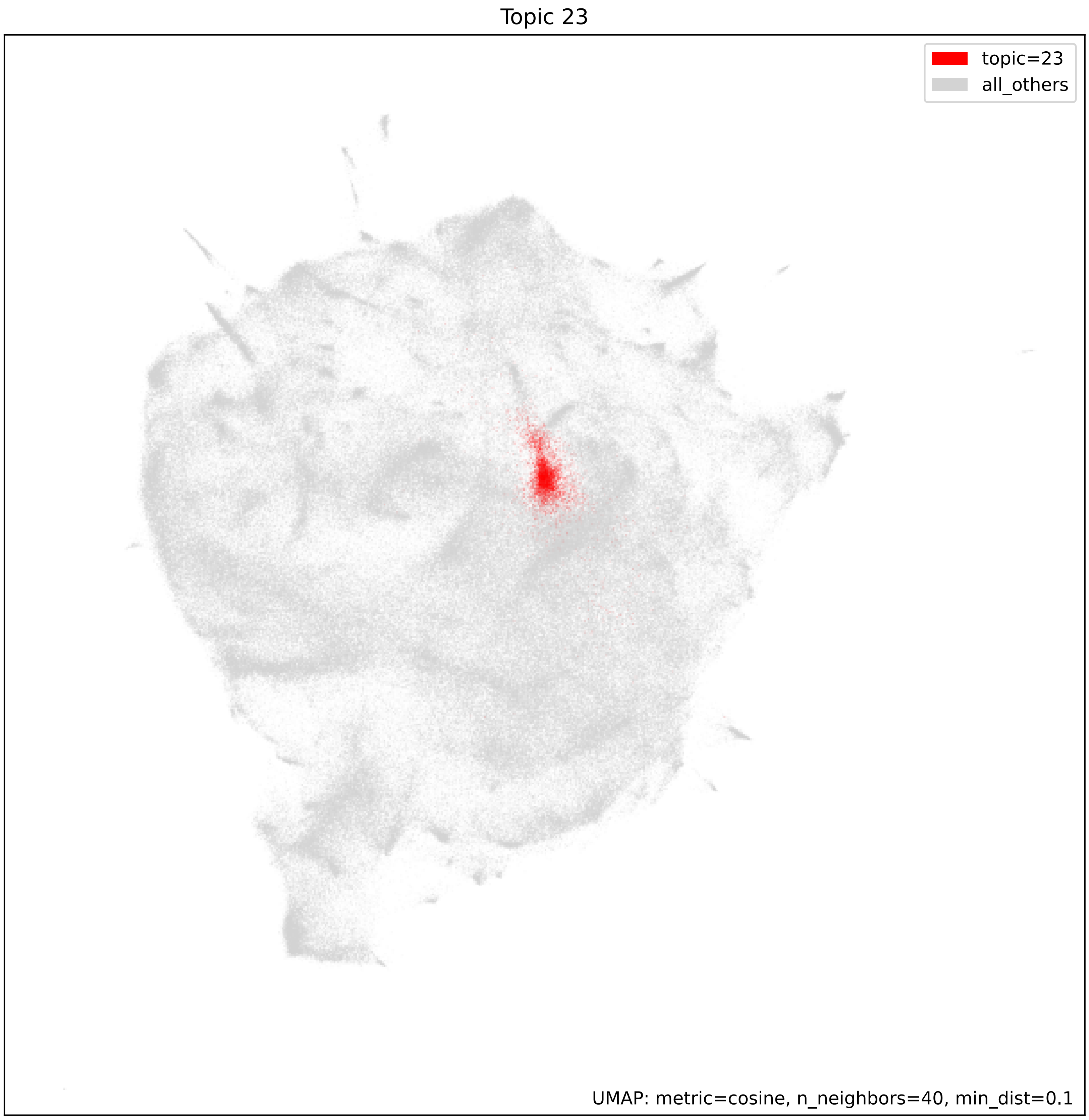
Topic 23
topic=23
all_others
UMAP: metric=cosine, n_neighbors=40, min_dist=0.1

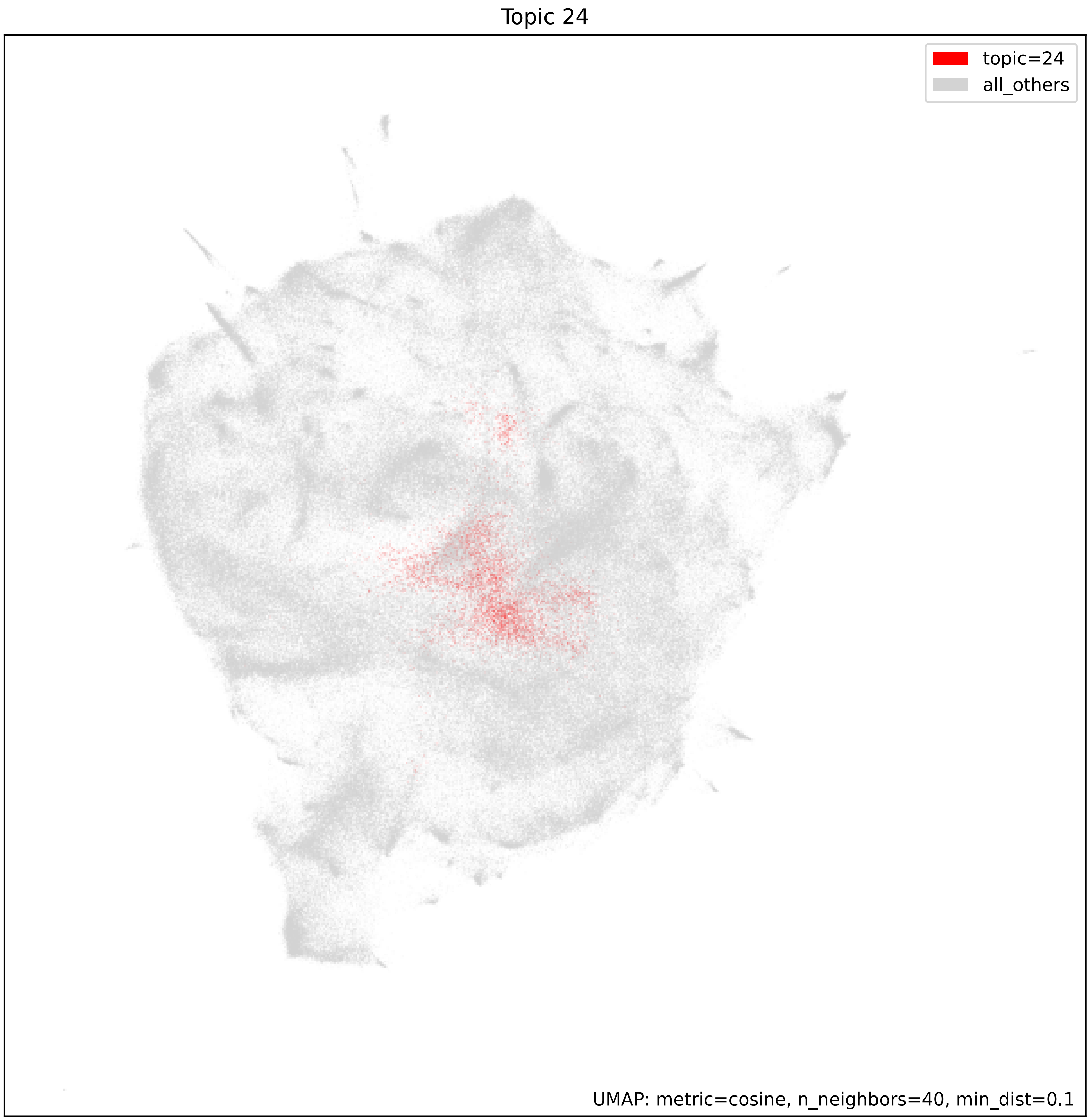
Topic 24
topic=24
all_others
UMAP: metric=cosine, n_neighbors=40, min_dist=0.1

Topic 25

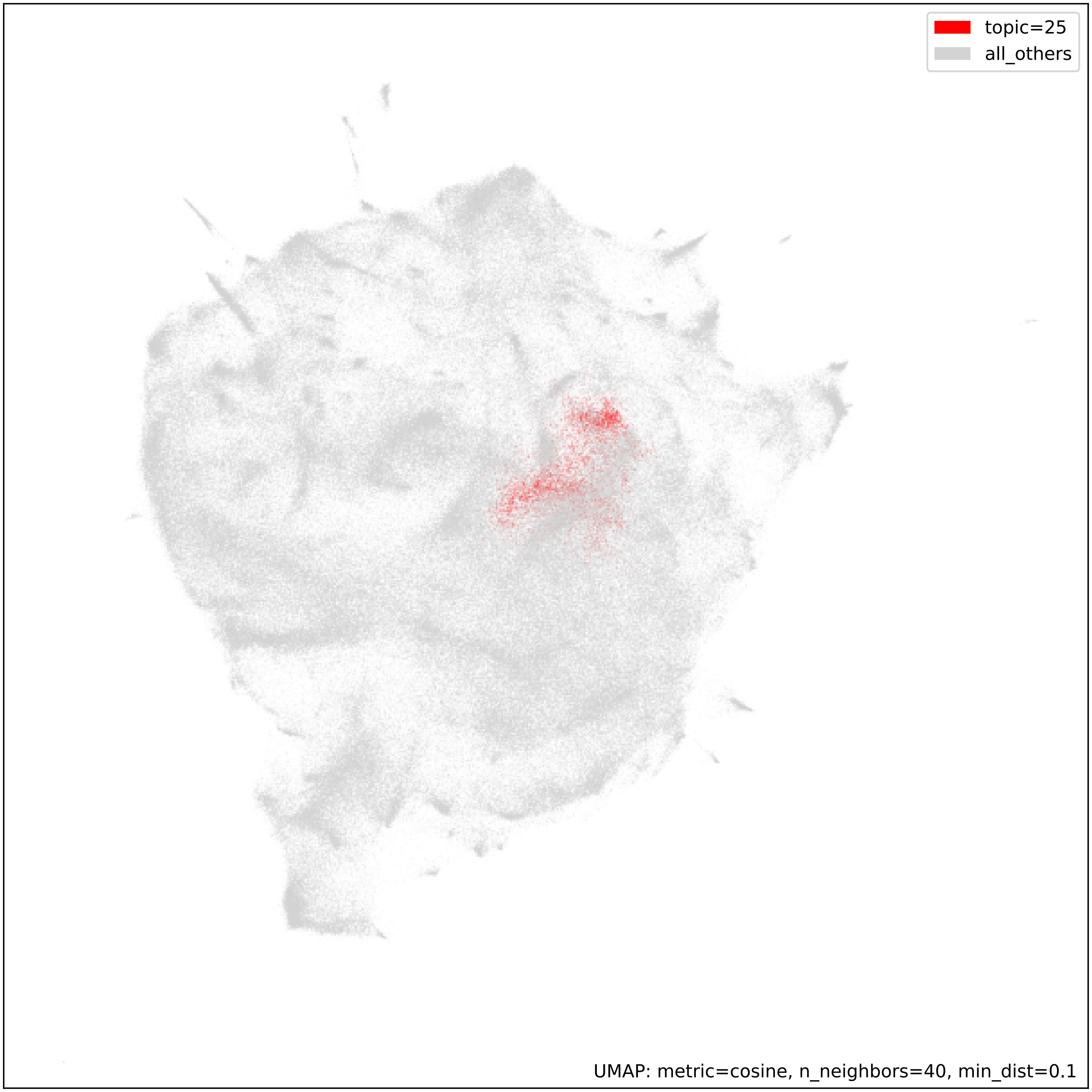
topic=25
all_others
UMAP: metric=cosine, n_neighbors=40, min_dist=0.1

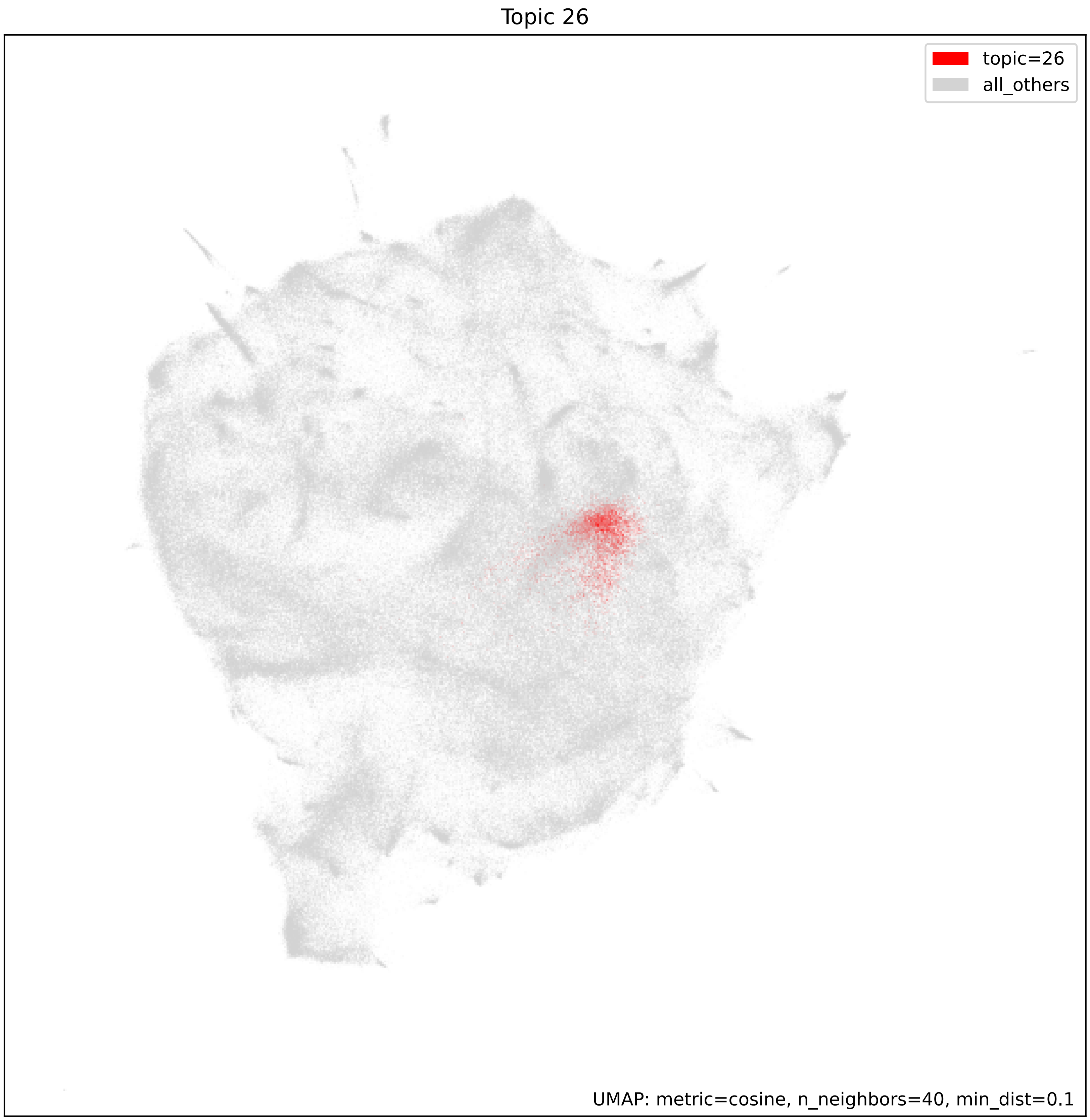
Topic 26
topic=26
all_others
UMAP: metric=cosine, n_neighbors=40, min_dist=0.1

Topic 27

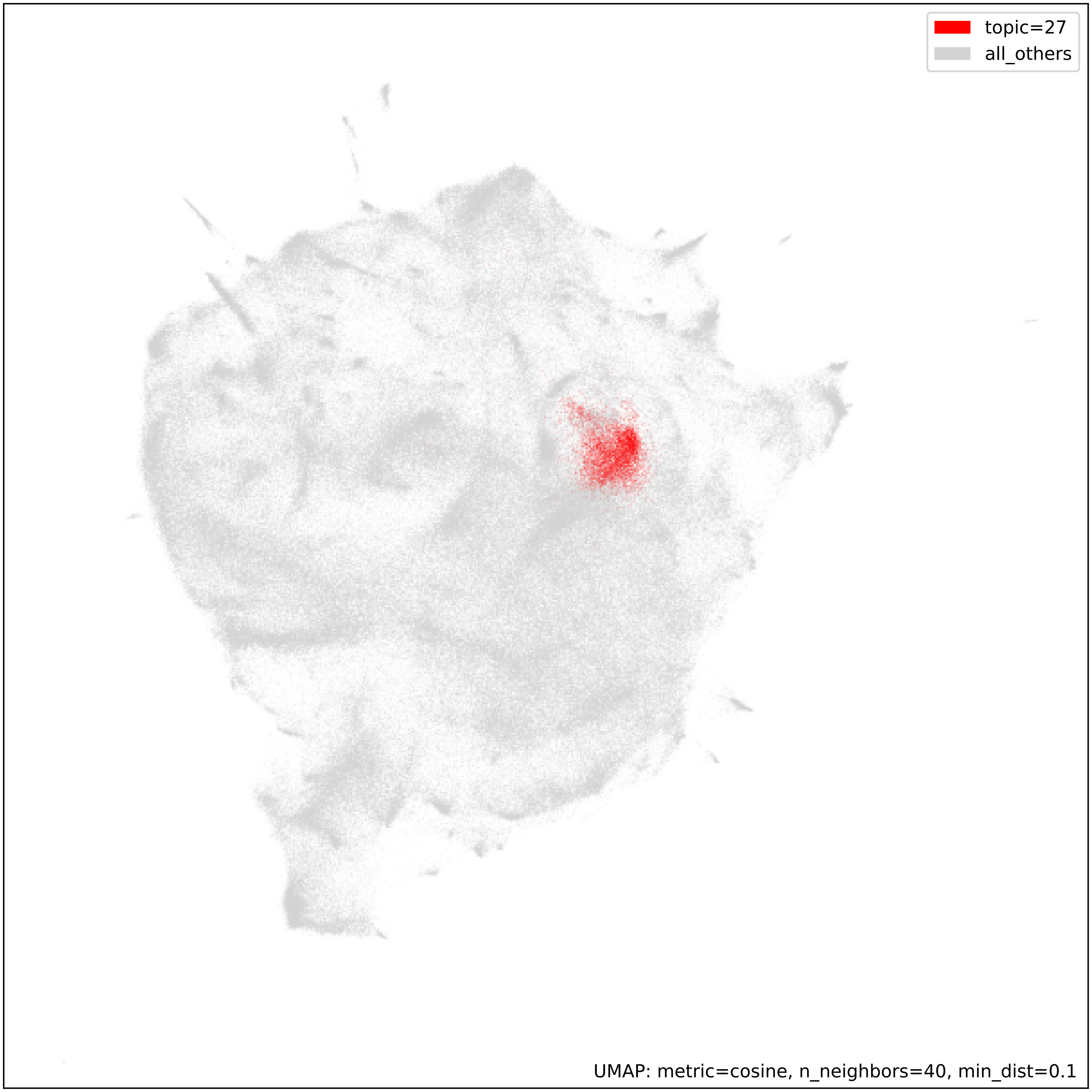

topic=27
all_others
UMAP: metric=cosine, n_neighbors=40, min_dist=0.1

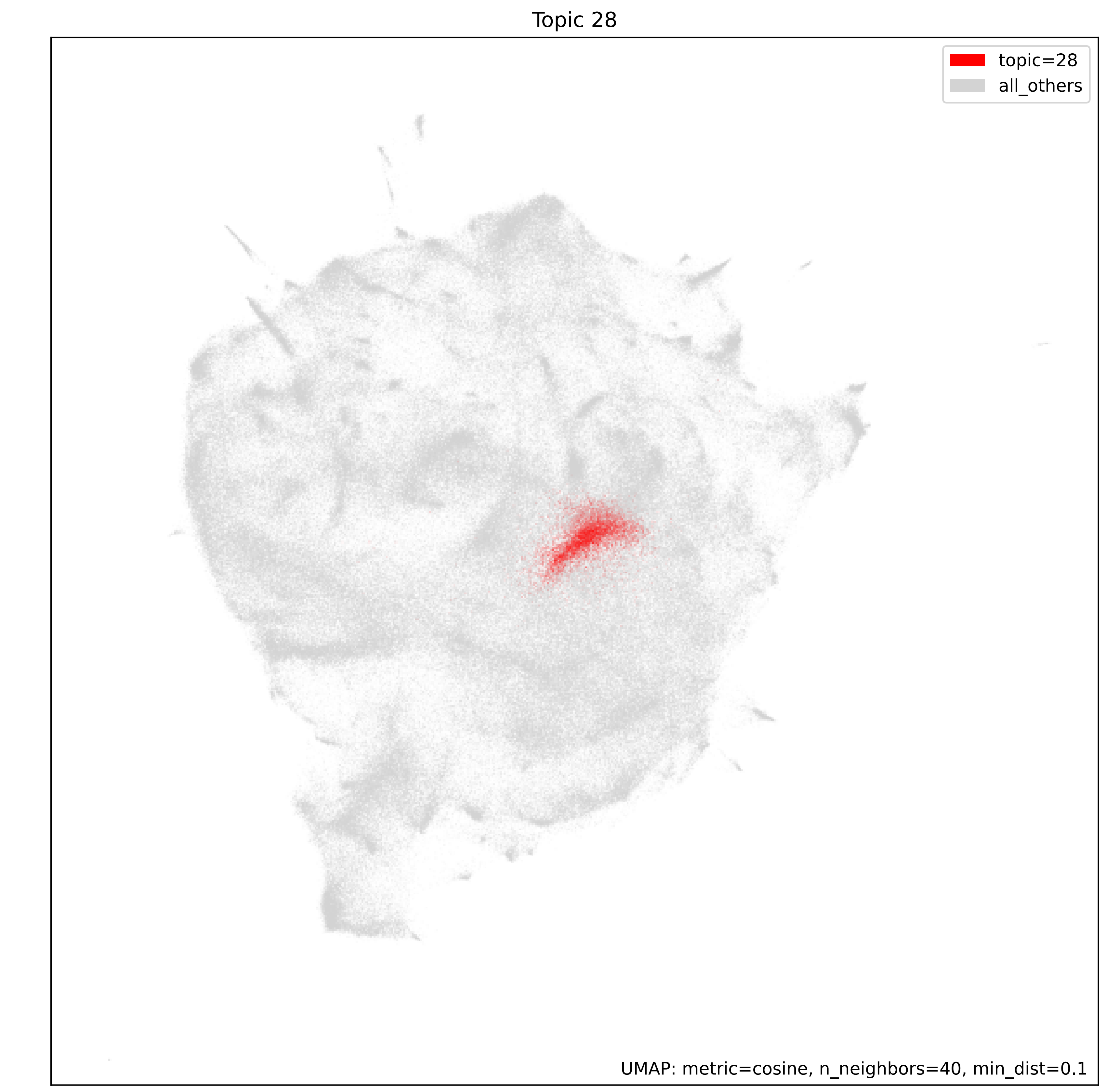

Topic 28
topic=28
all_others
UMAP: metric=cosine, n_neighbors=40, min_dist=0.1

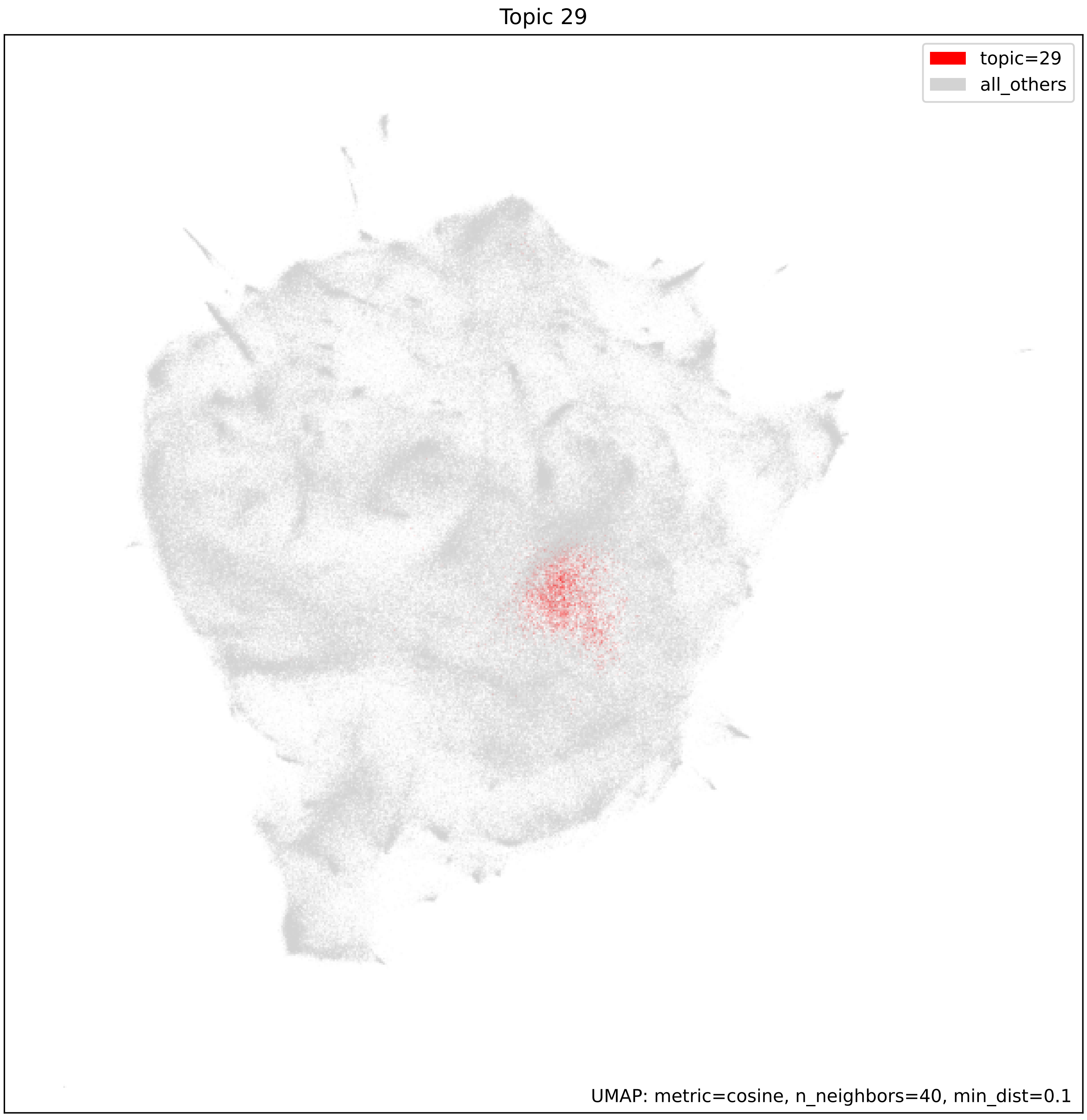

Topic 29
topic=29
all_others
UMAP: metric=cosine, n_neighbors=40, min_dist=0.1

Topic 30

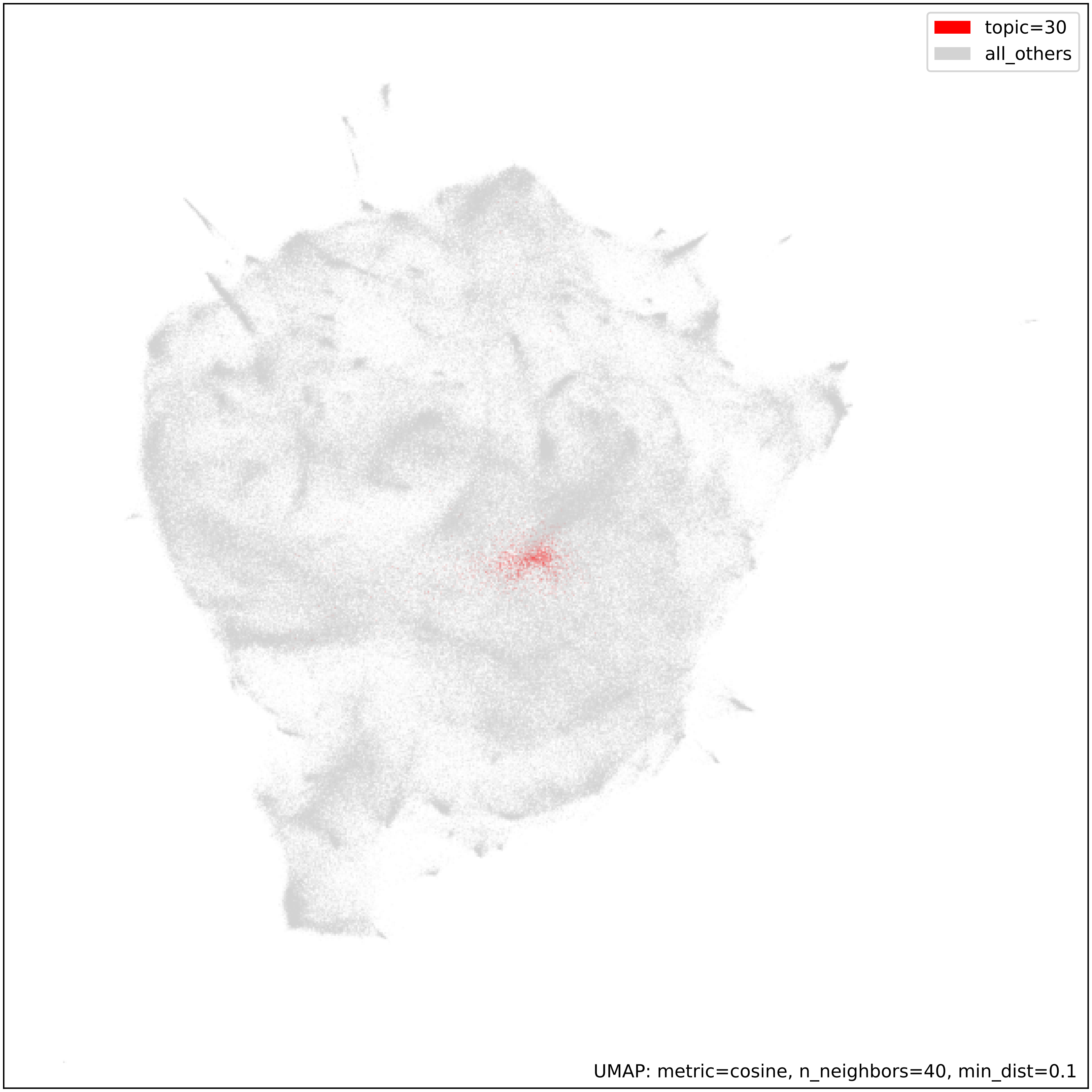
topic=30
all_others
UMAP: metric=cosine, n_neighbors=40, min_dist=0.1

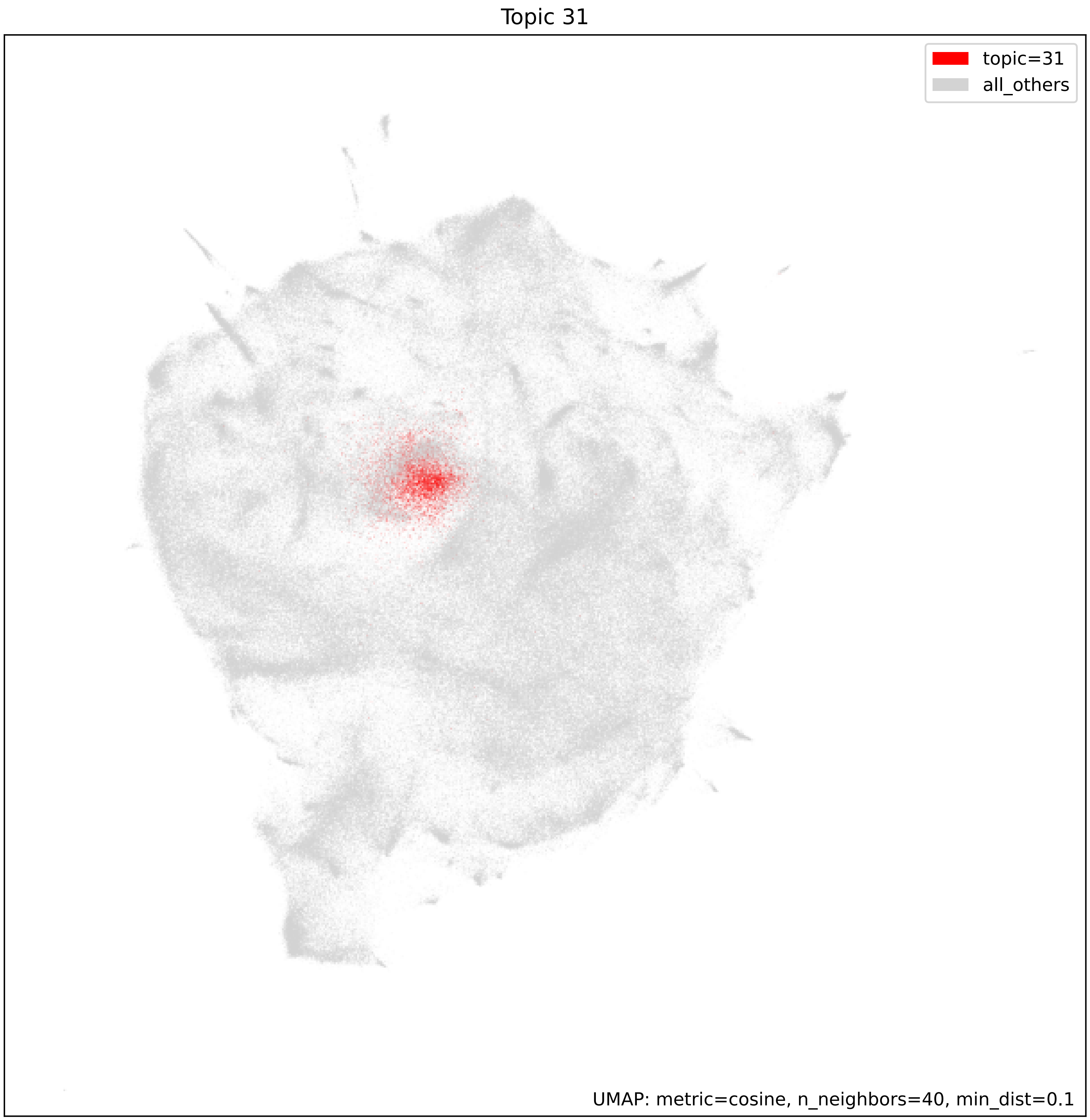
Topic 31
topic=31
all_others
UMAP: metric=cosine, n_neighbors=40, min_dist=0.1

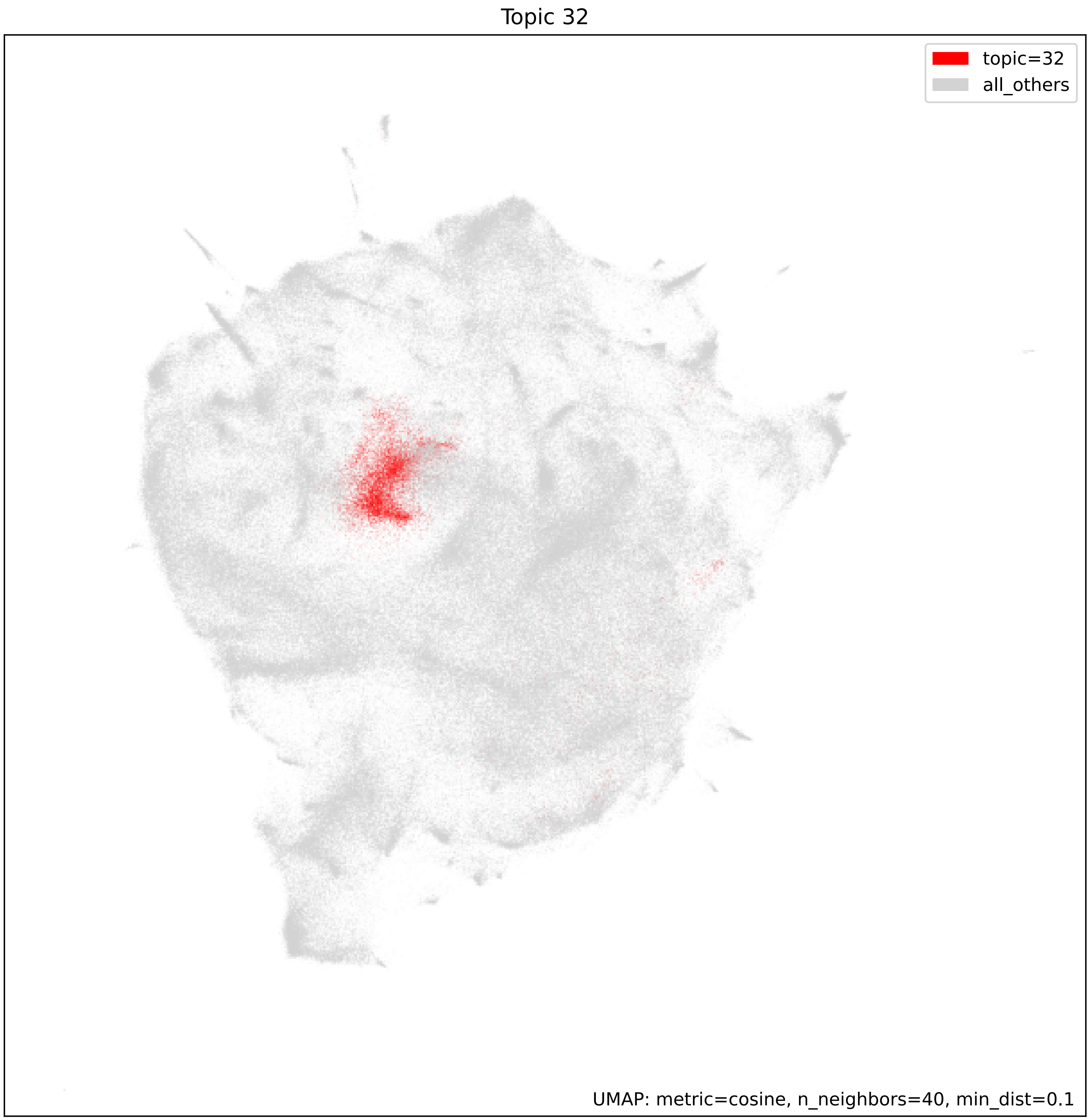
Topic 32
topic=32
all_others
UMAP: metric=cosine, n_neighbors=40, min_dist=0.1

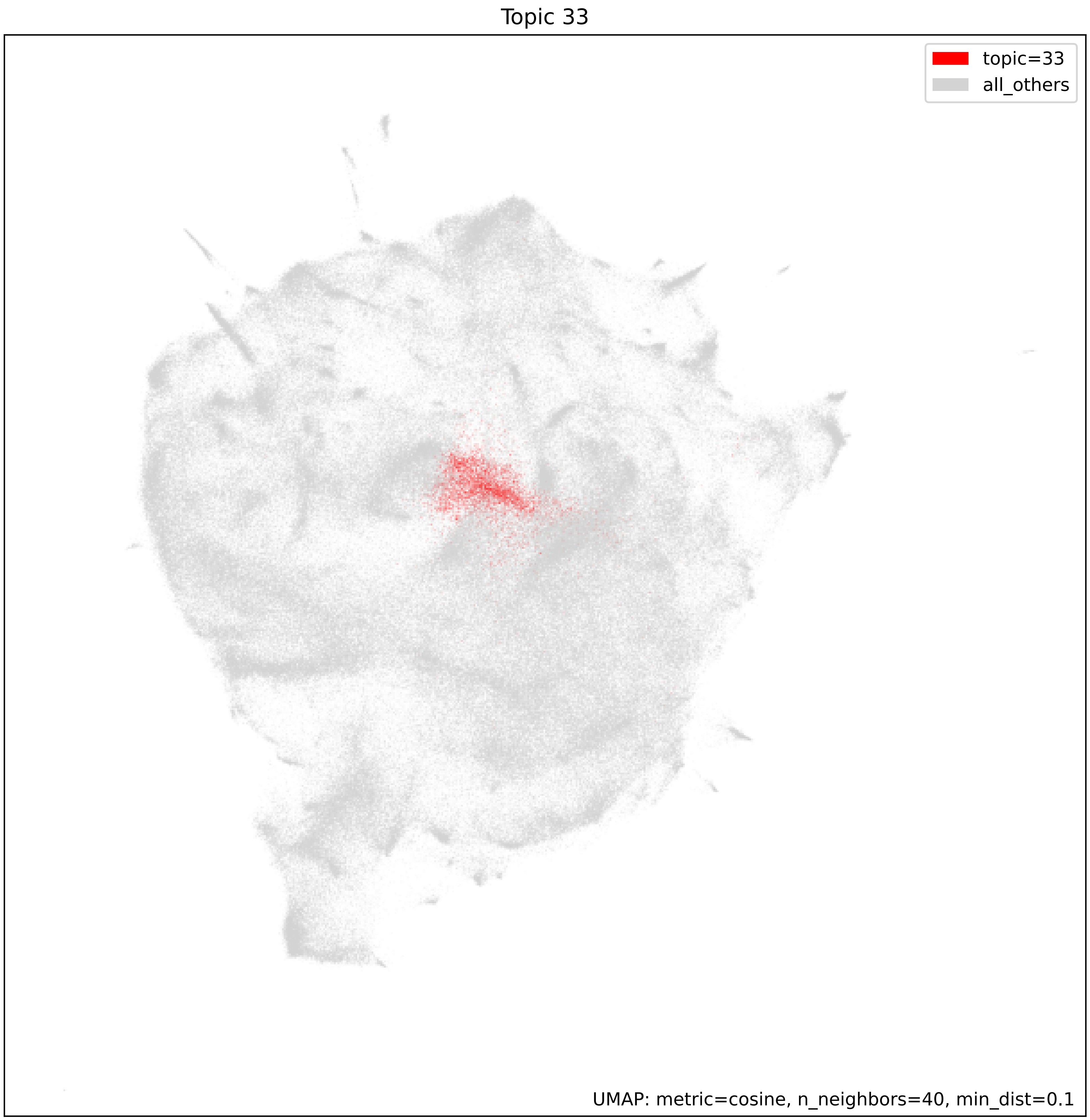

Topic 34

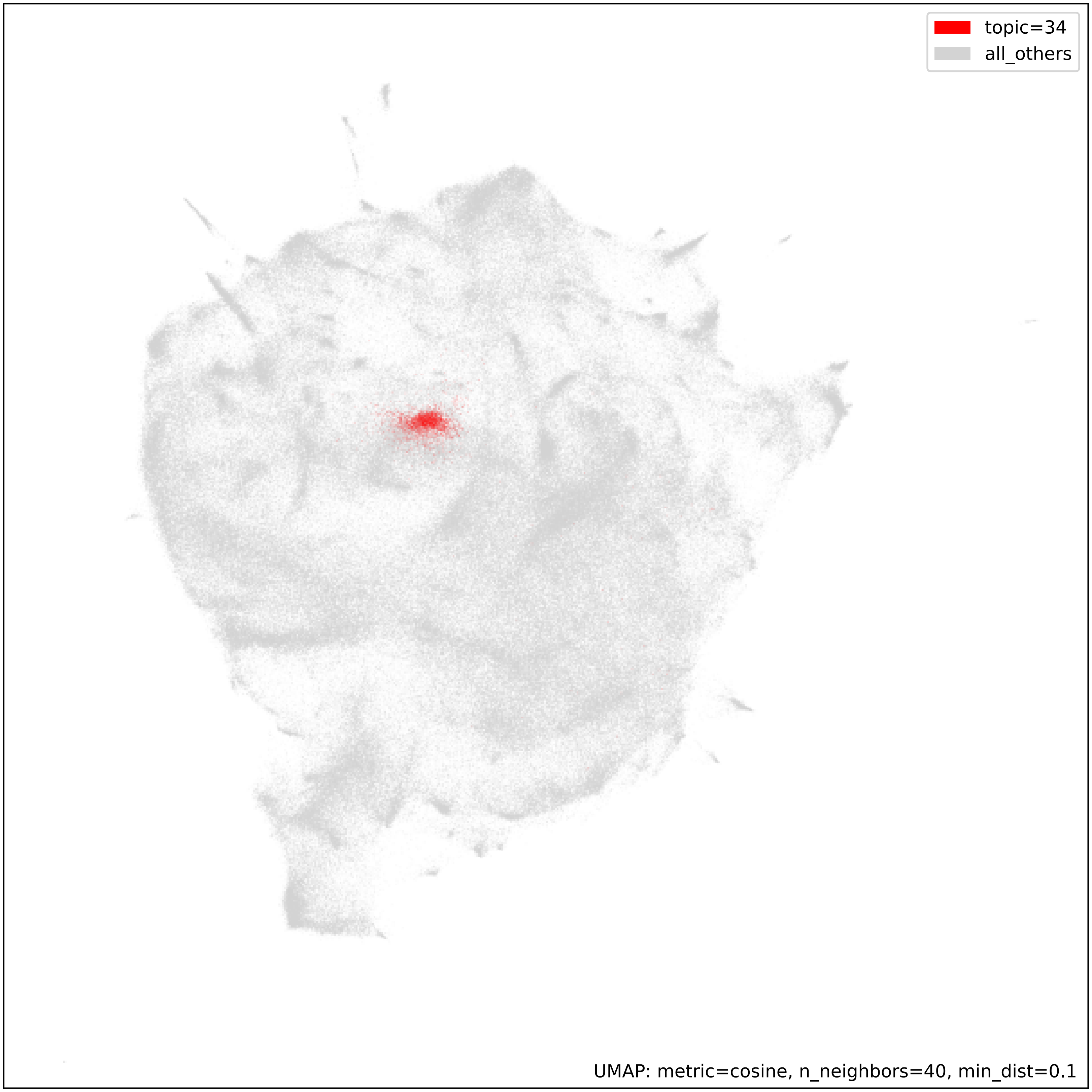
topic=34
all_others
UMAP: metric=cosine, n_neighbors=40, min_dist=0.1

Topic 35

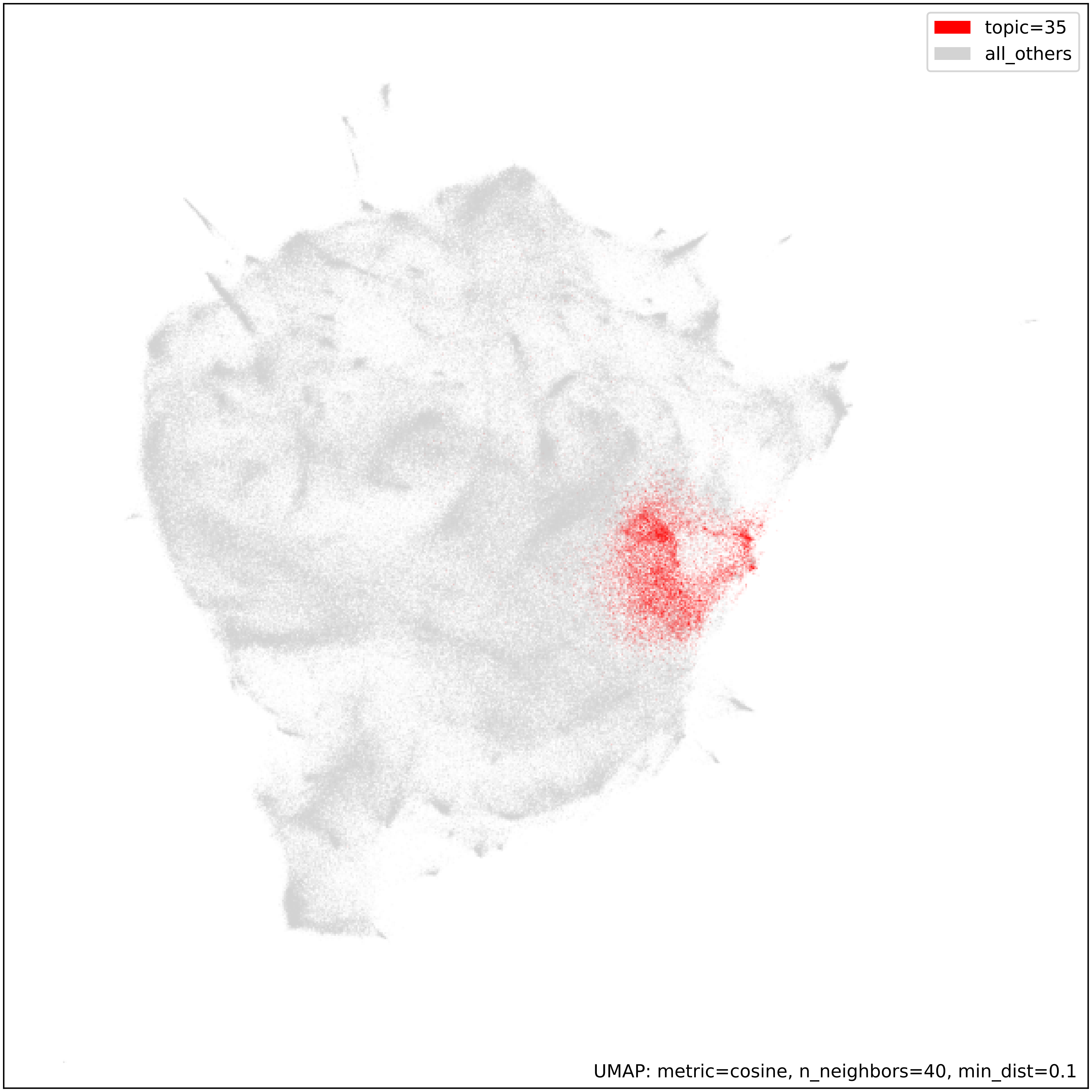
topic=35
all_others
UMAP: metric=cosine, n_neighbors=40, min_dist=0.1

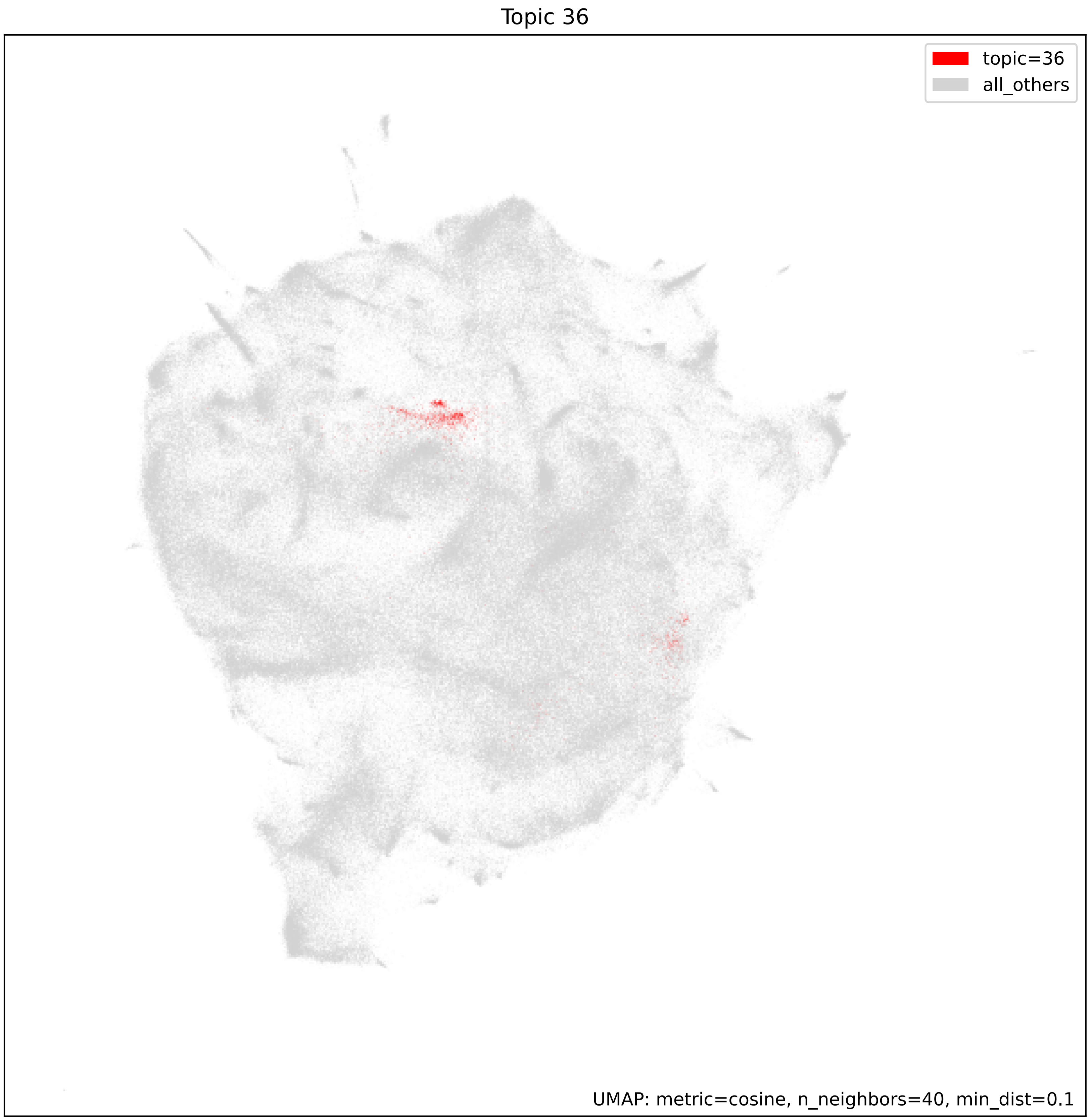
Topic 36
topic=36
all_others
UMAP: metric=cosine, n_neighbors=40, min_dist=0.1

Topic 37

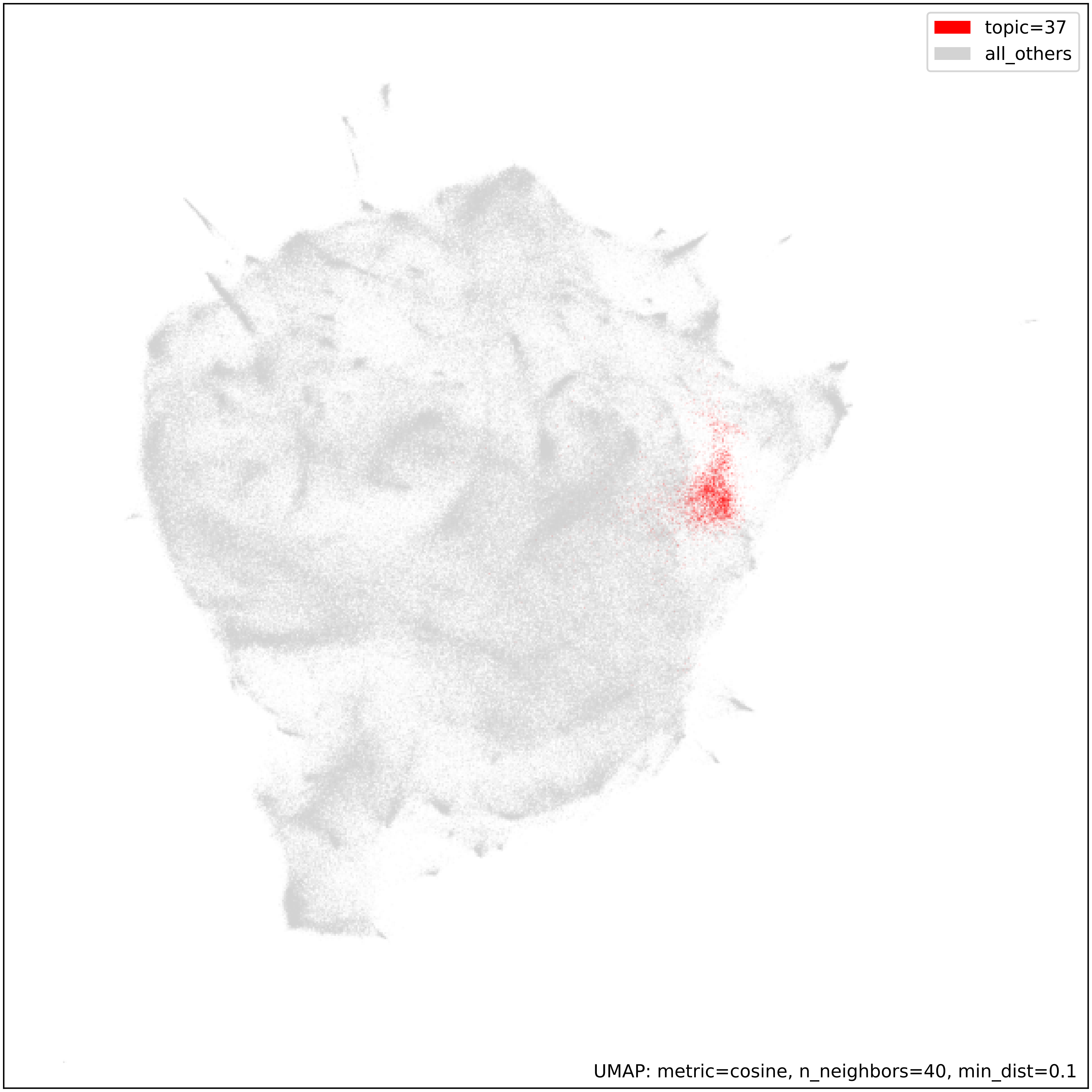
topic=37
all_others
UMAP: metric=cosine, n_neighbors=40, min_dist=0.1

Topic 38

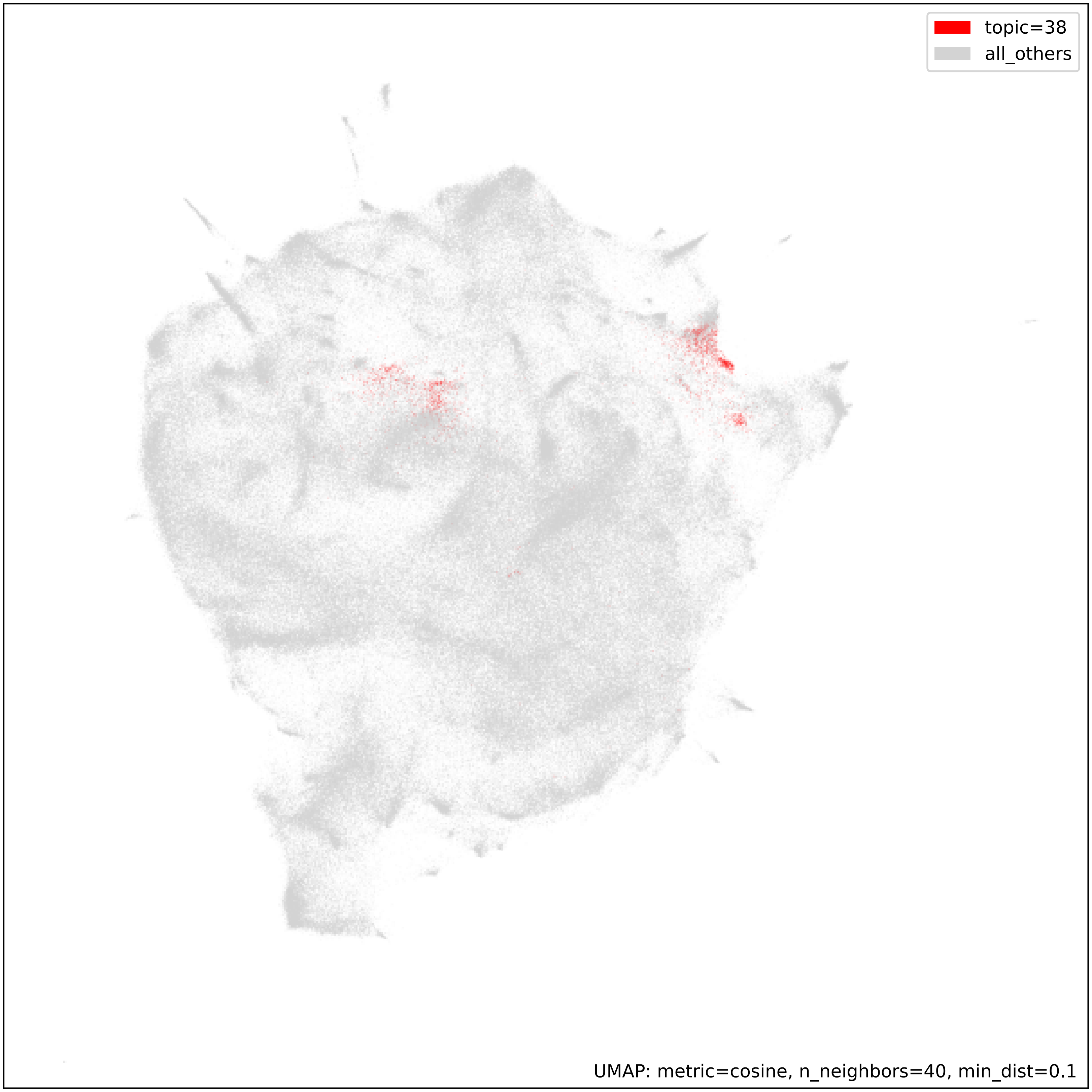
topic=38
all_others
UMAP: metric=cosine, n_neighbors=40, min_dist=0.1

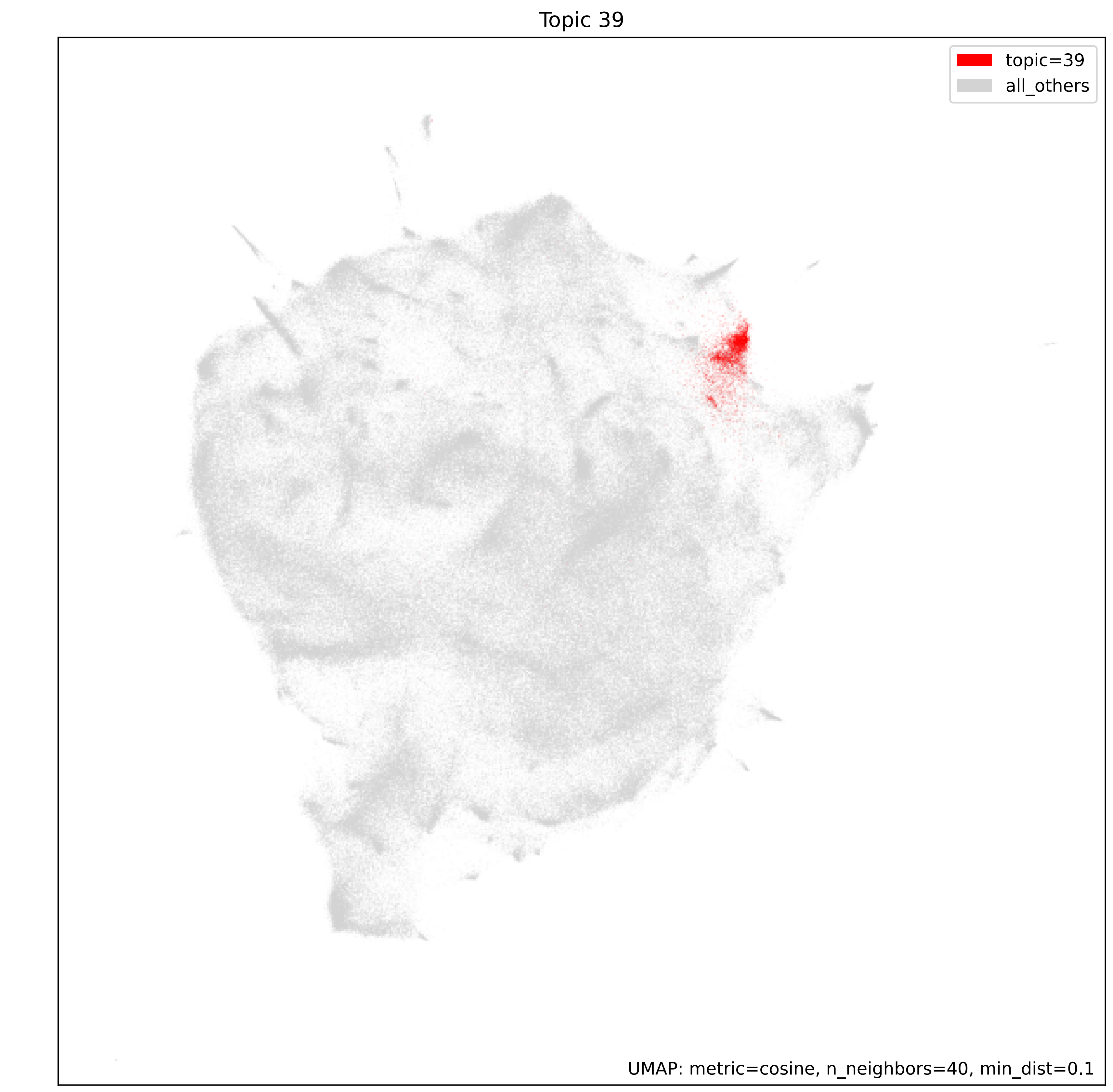
Topic 39
topic=39
all_others
UMAP: metric=cosine, n_neighbors=40, min_dist=0.1

Topic 40

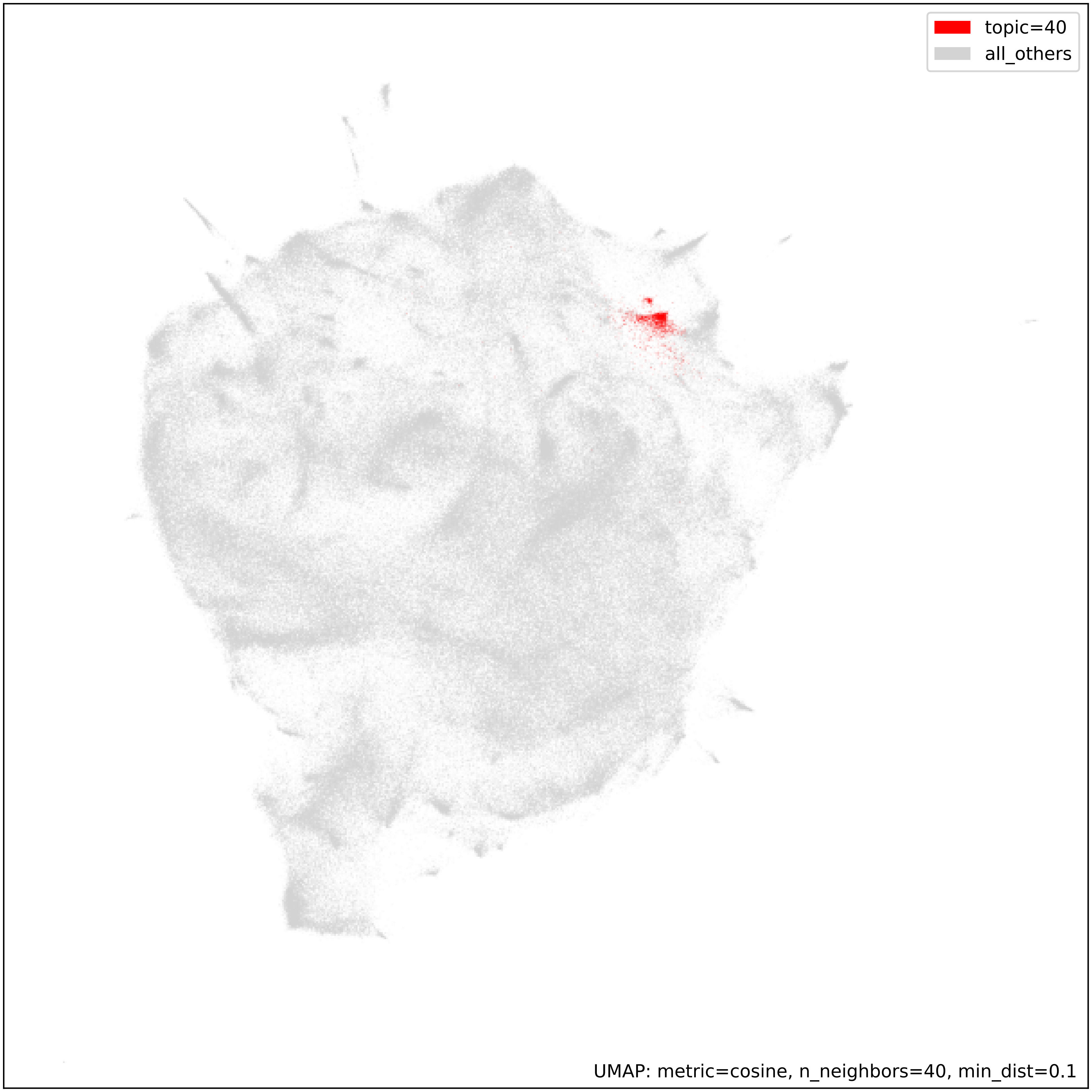
topic=40
all_others
UMAP: metric=cosine, n_neighbors=40, min_dist=0.1

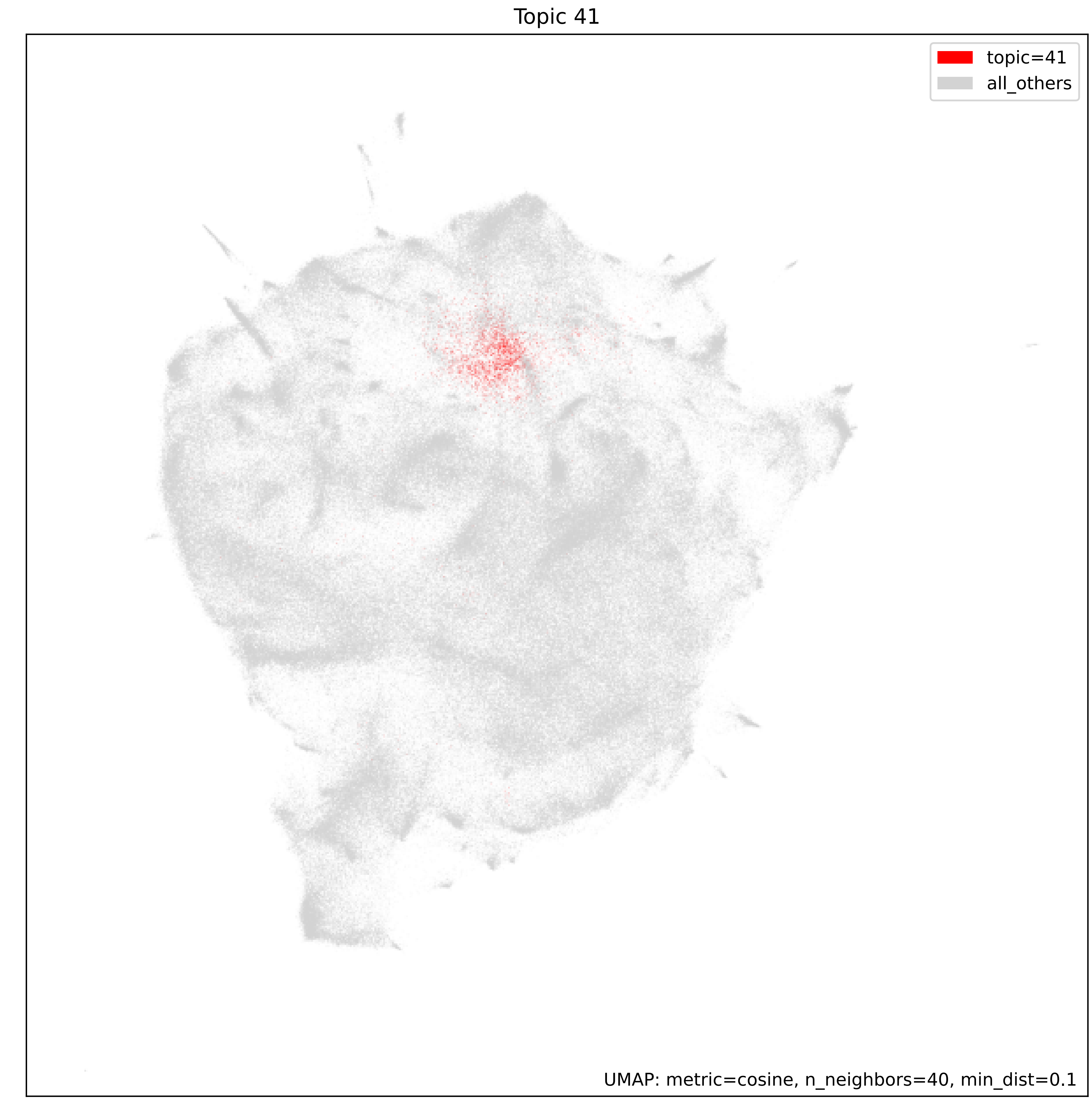
Topic 41
topic=41
all_others
UMAP: metric=cosine, n_neighbors=40, min_dist=0.1

Topic 42

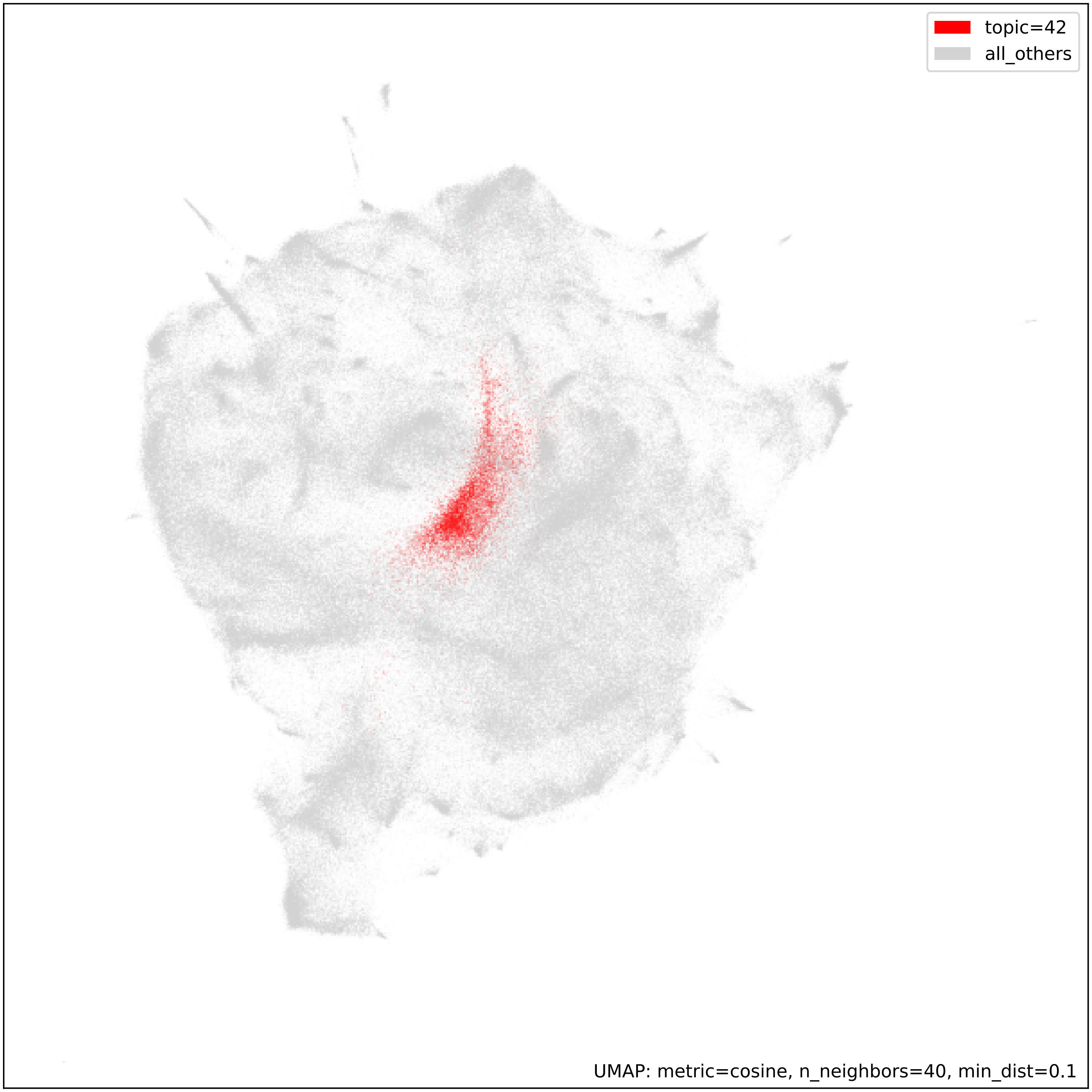
topic=42
all_others
UMAP: metric=cosine, n_neighbors=40, min_dist=0.1

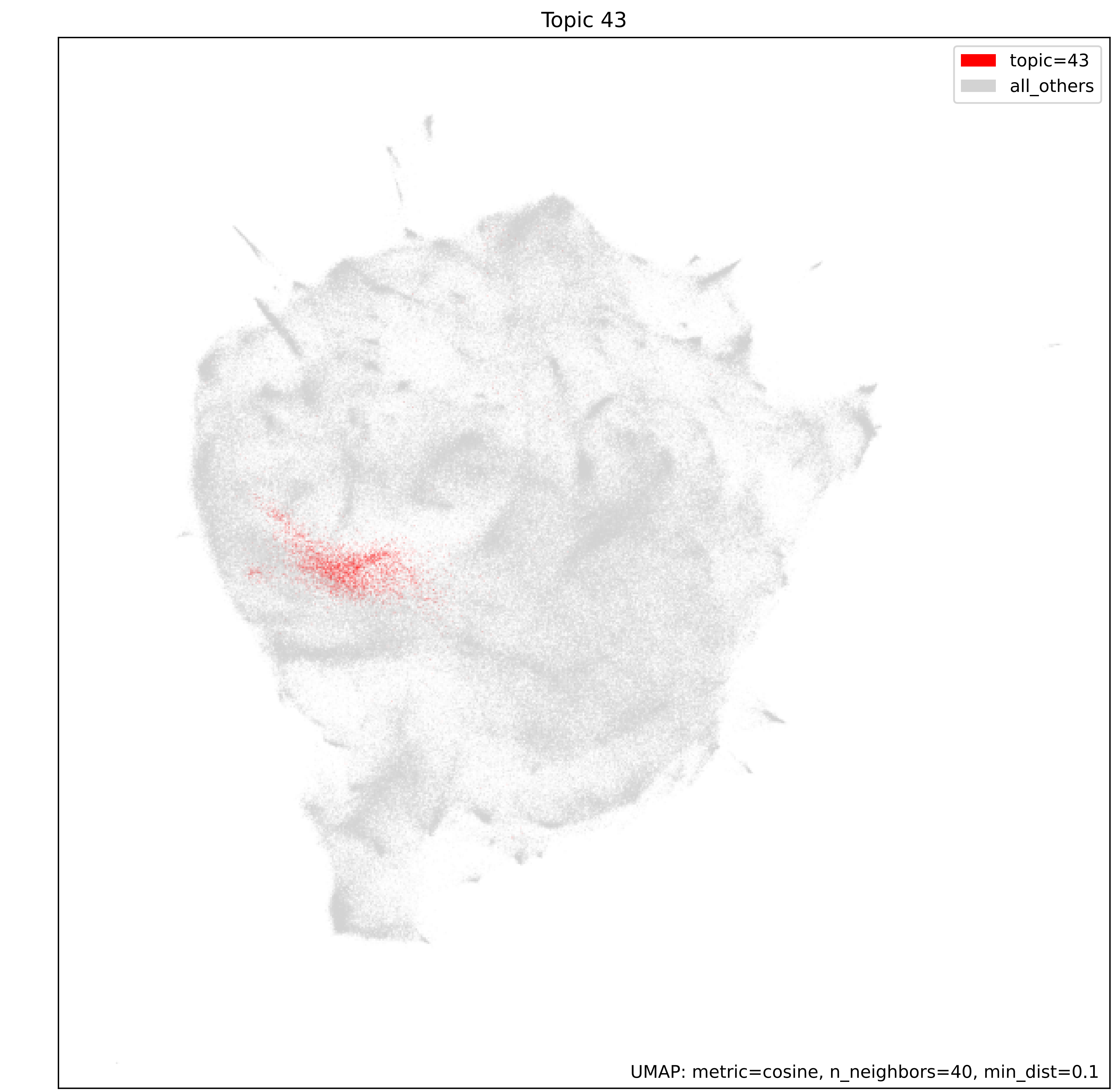
Topic 43
topic=43
all_others
UMAP: metric=cosine, n_neighbors=40, min_dist=0.1

Topic 44

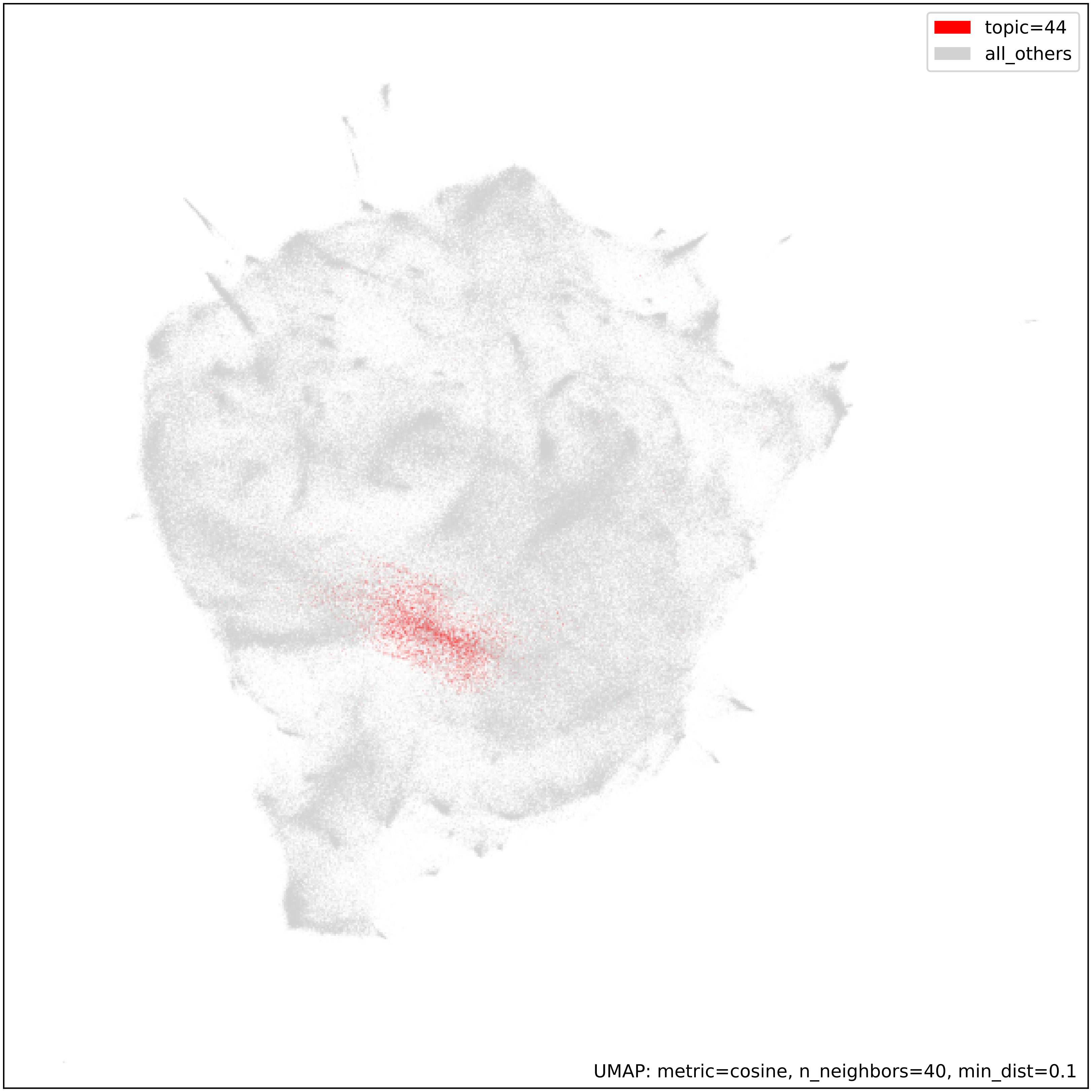

topic=44
all_others
UMAP: metric=cosine, n_neighbors=40, min_dist=0.1

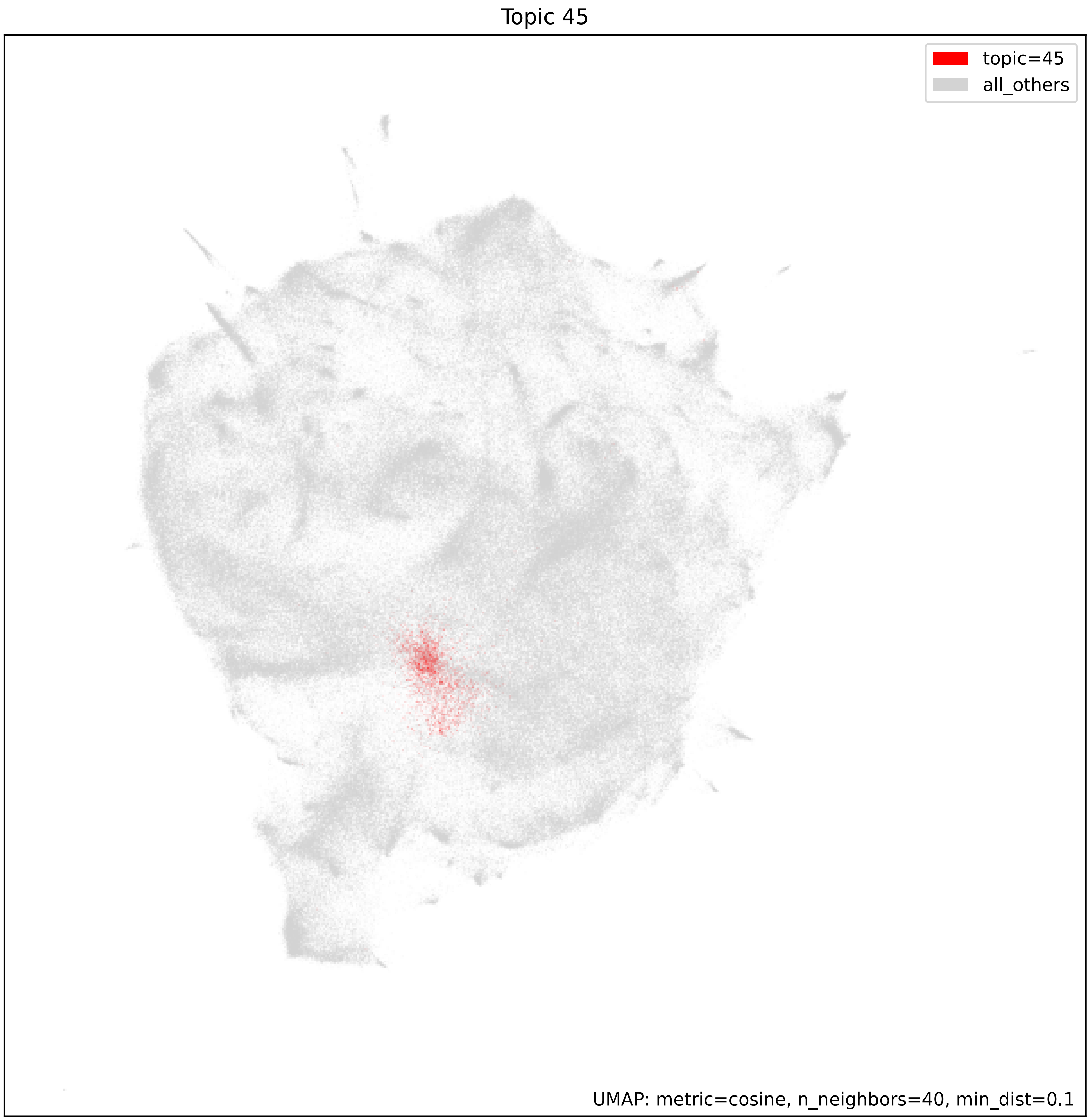
Topic 45
topic=45
all_others
UMAP: metric=cosine, n_neighbors=40, min_dist=0.1

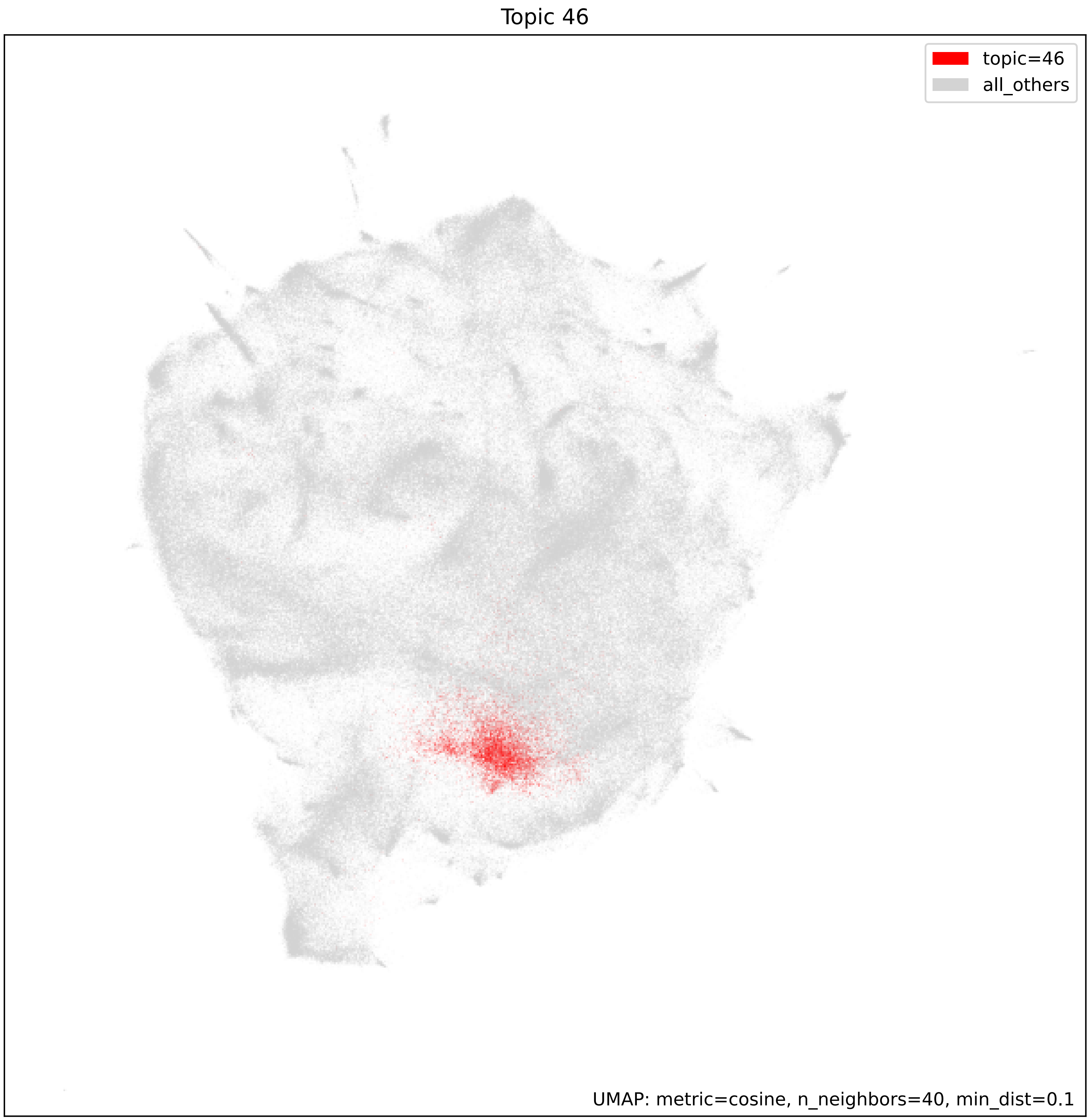
Topic 46
topic=46
all_others
UMAP: metric=cosine, n_neighbors=40, min_dist=0.1

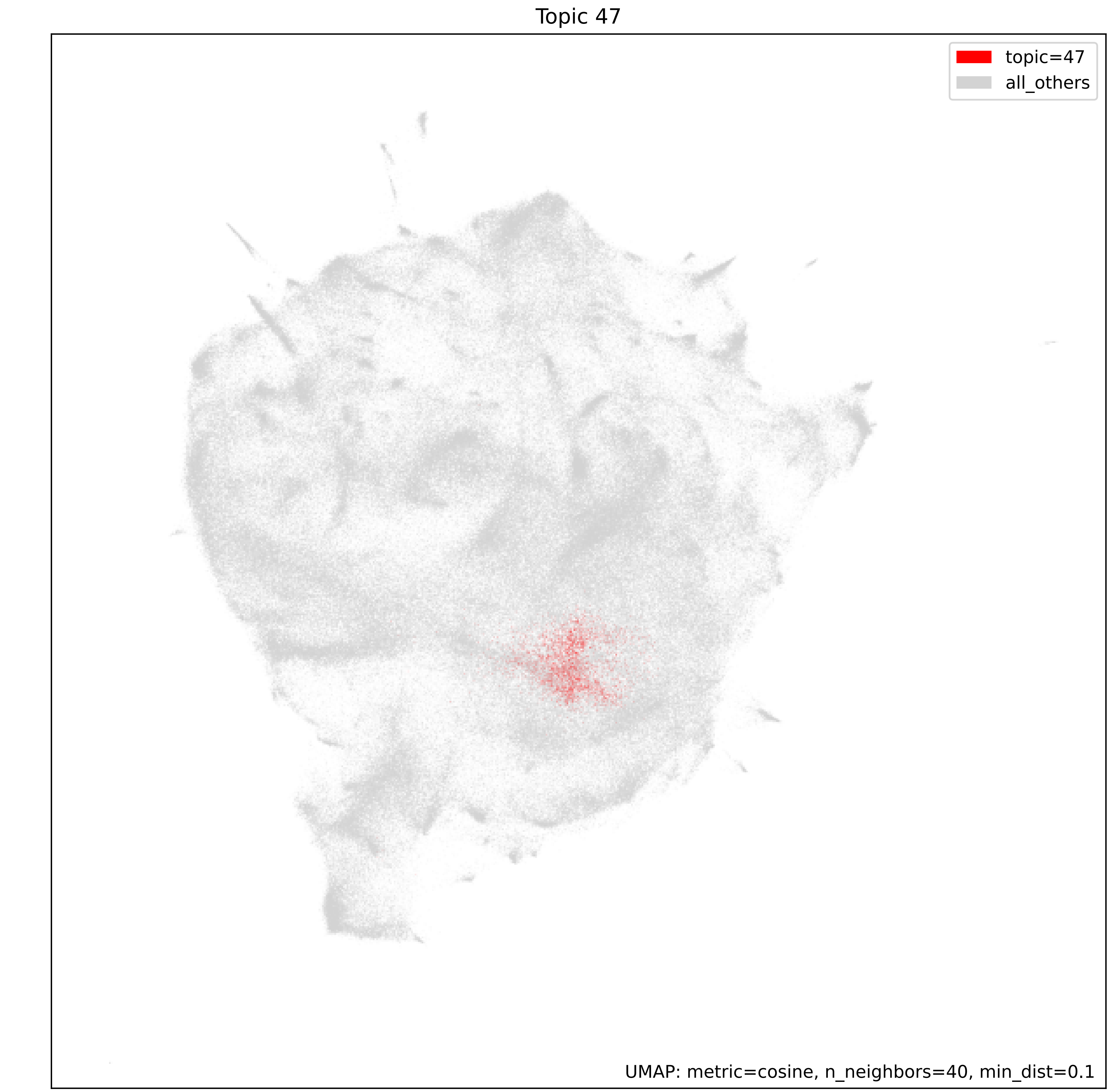
Topic 47
topic=47
all_others
UMAP: metric=cosine, n_neighbors=40, min_dist=0.1

Topic 48

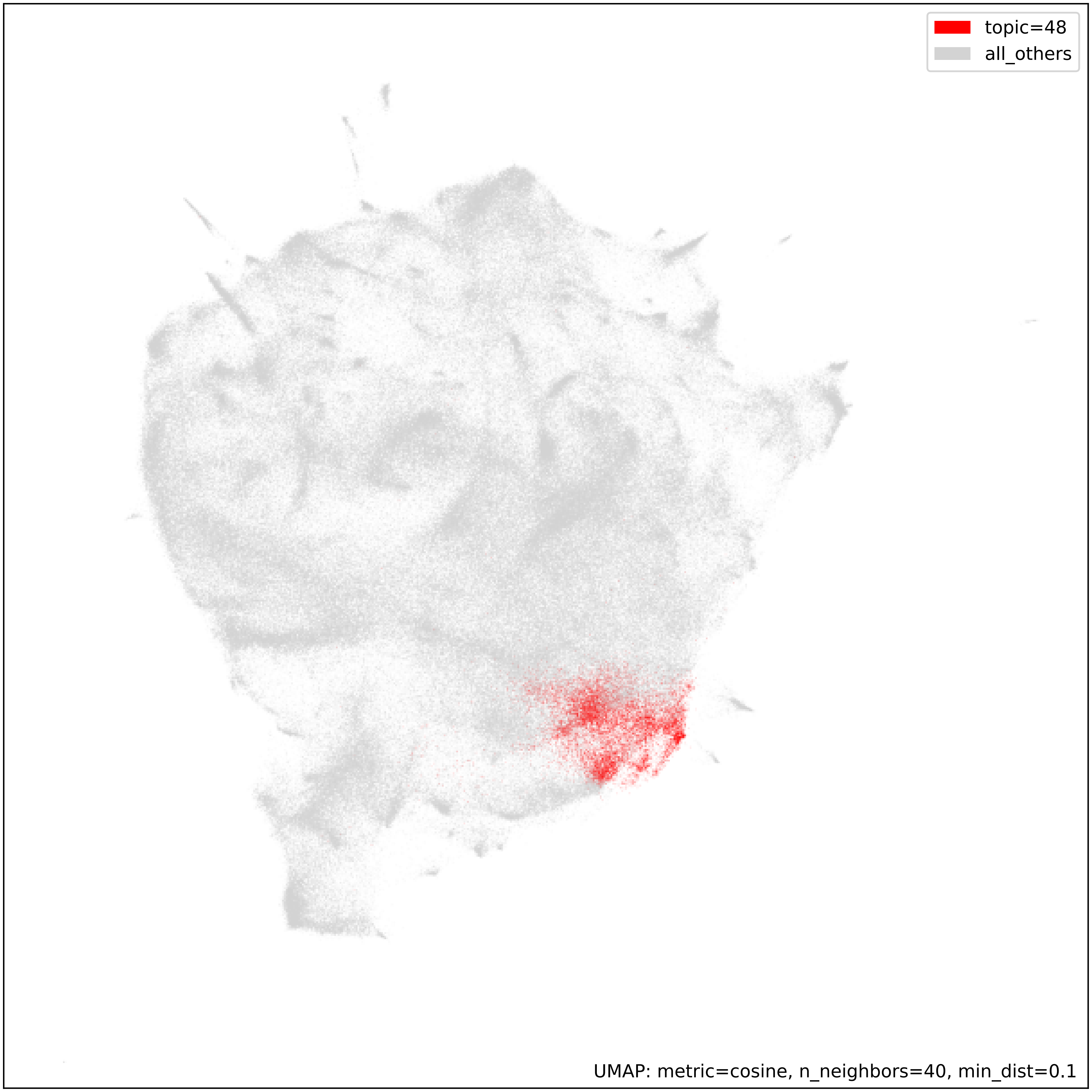
topic=48
all_others
UMAP: metric=cosine, n_neighbors=40, min_dist=0.1

Topic 49

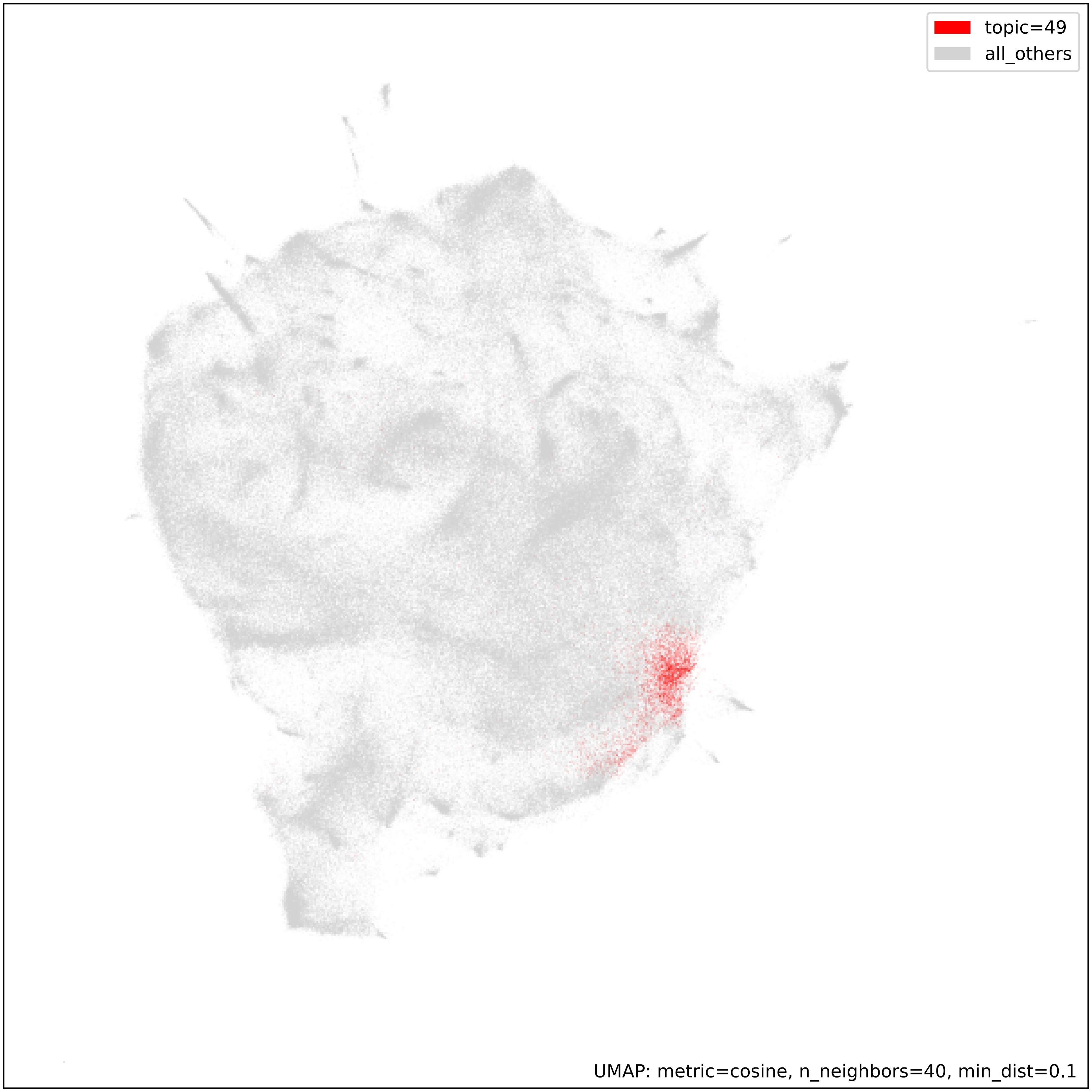

topic=49
all_others
UMAP: metric=cosine, n_neighbors=40, min_dist=0.1

Topic 50

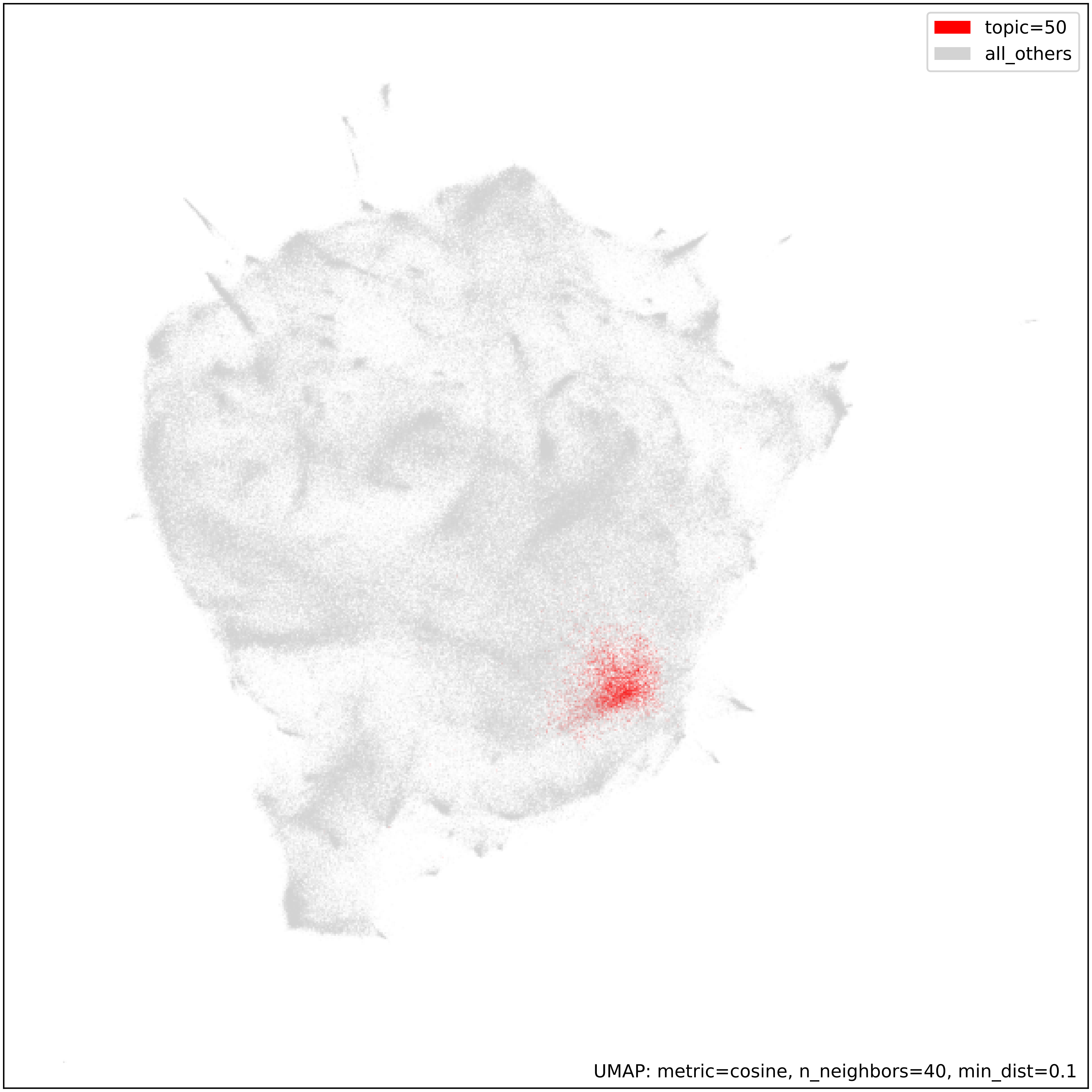
topic=50
all_others
UMAP: metric=cosine, n_neighbors=40, min_dist=0.1

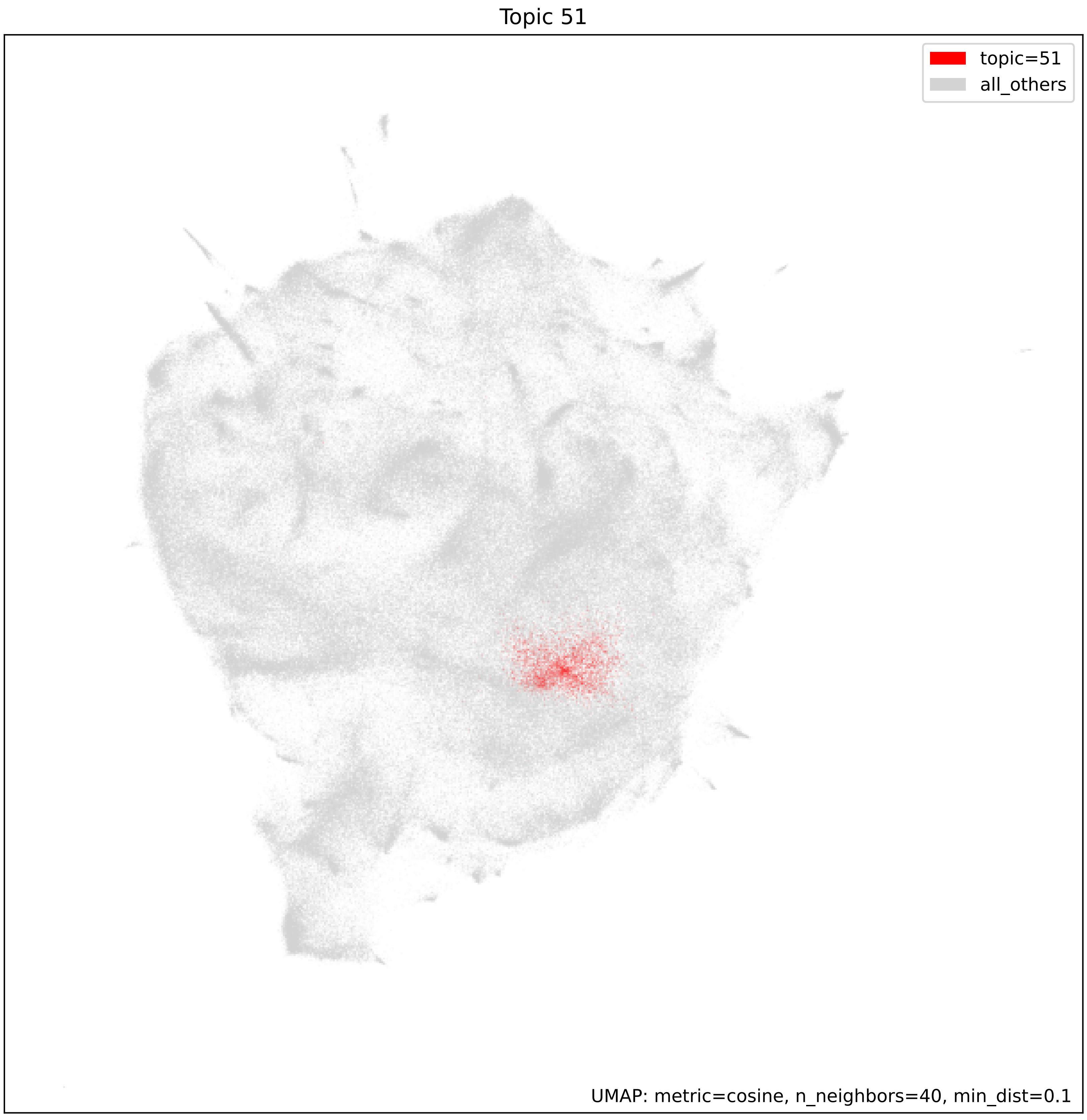
Topic 51
topic=51
all_others
UMAP: metric=cosine, n_neighbors=40, min_dist=0.1

Topic 52

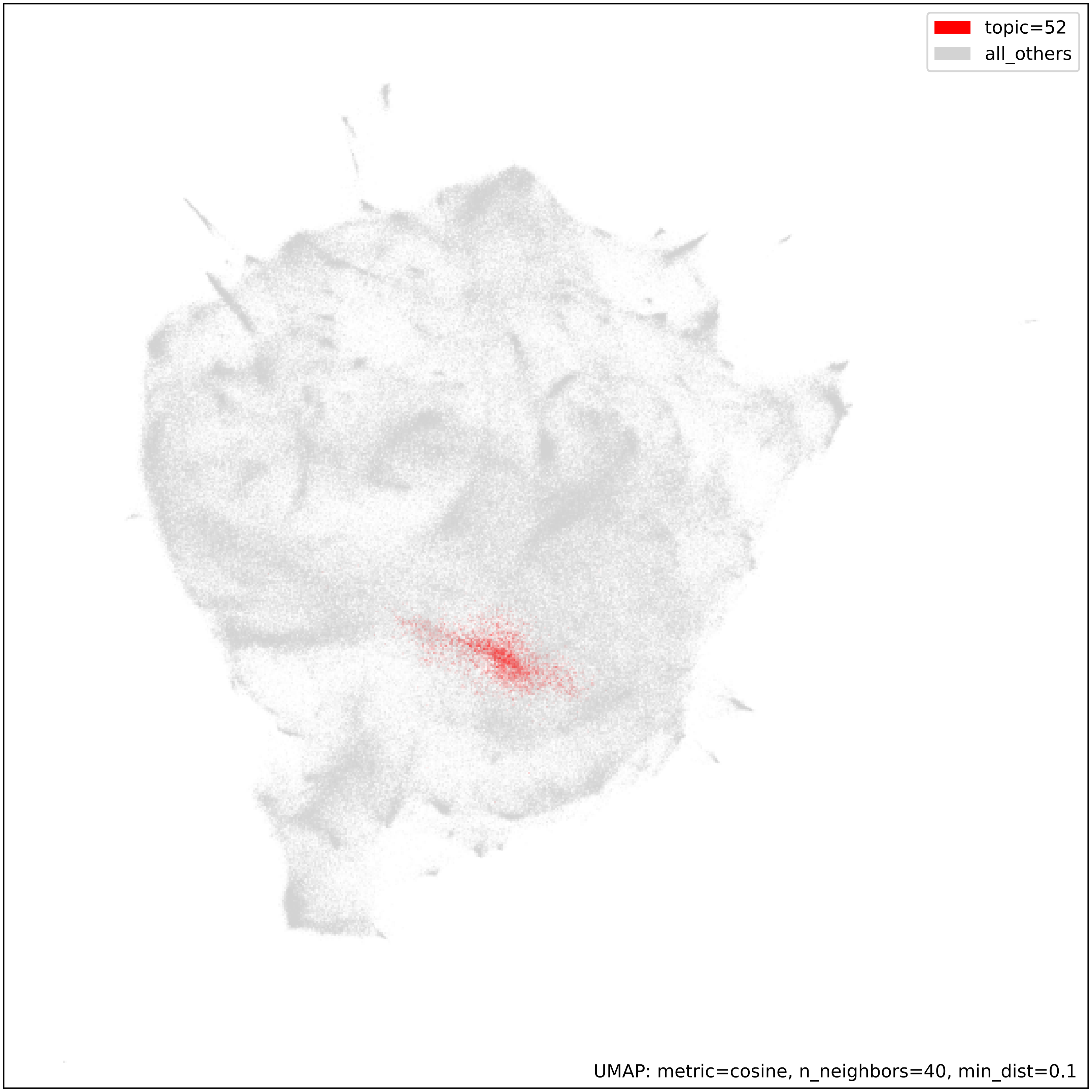
topic=52
all_others
UMAP: metric=cosine, n_neighbors=40, min_dist=0.1

Topic 53

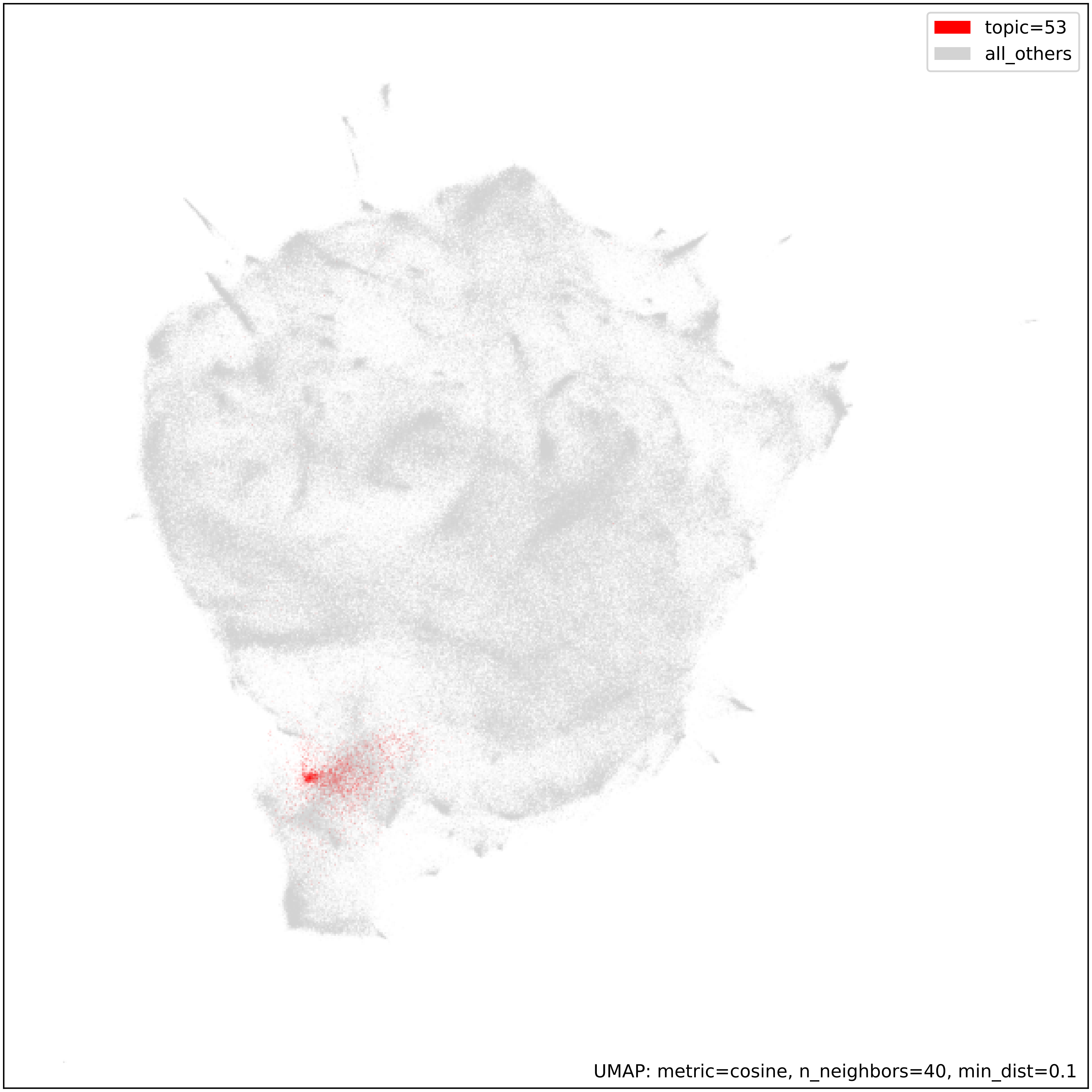

topic=53
all_others
UMAP: metric=cosine, n_neighbors=40, min_dist=0.1

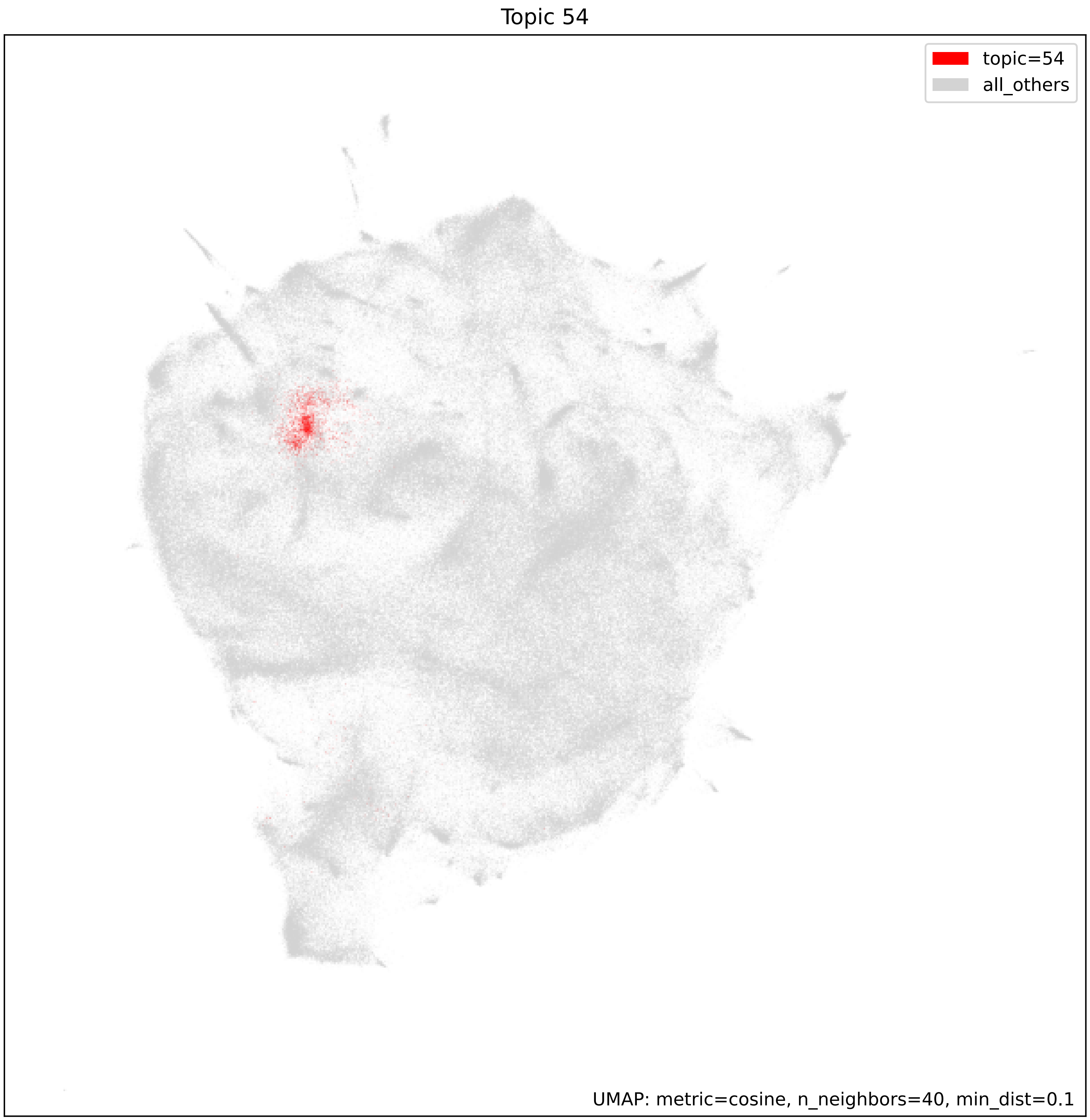
Topic 54
topic=54
all_others
UMAP: metric=cosine, n_neighbors=40, min_dist=0.1

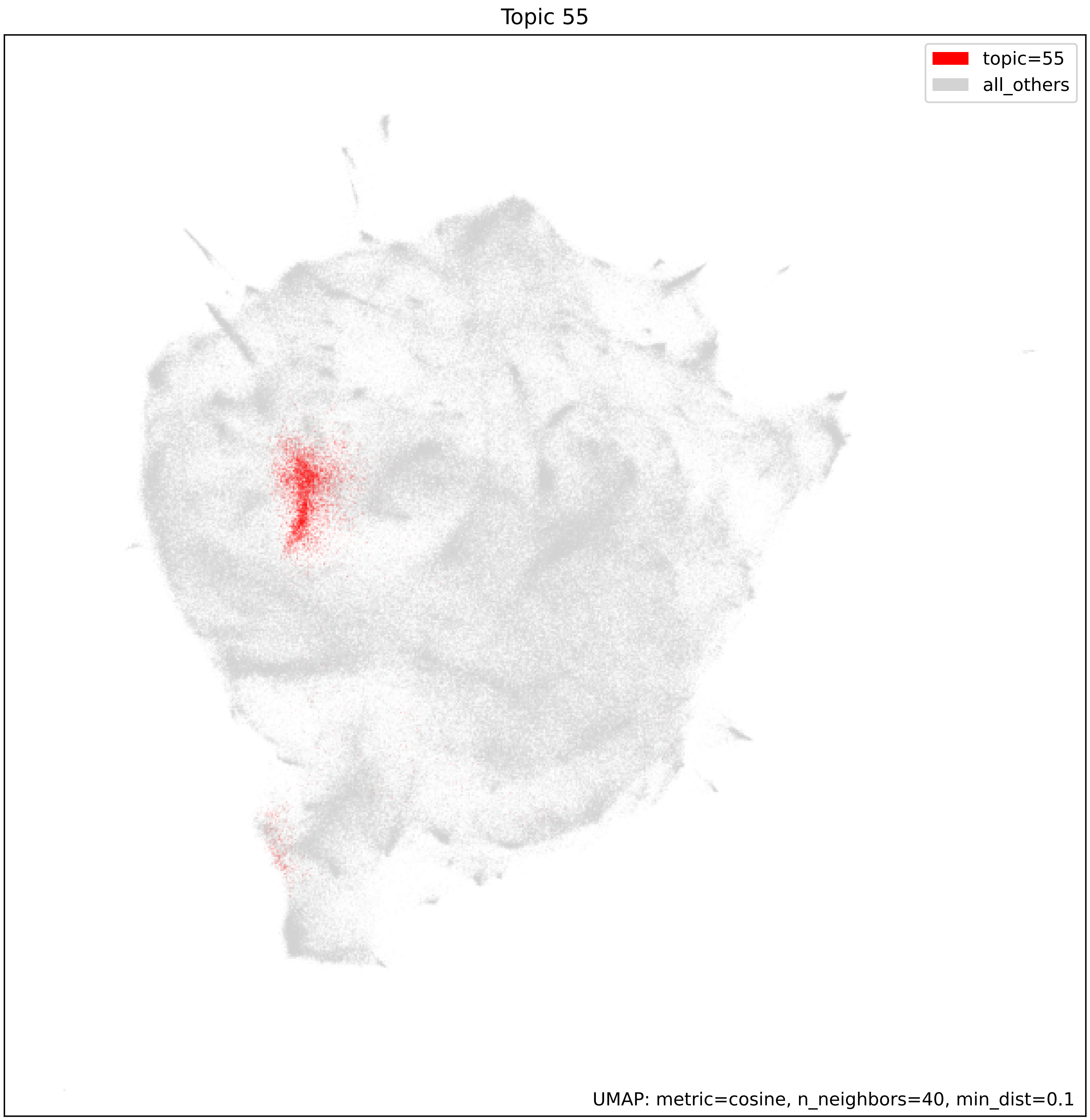
Topic 55
topic=55
all_others
UMAP: metric=cosine, n_neighbors=40, min_dist=0.1

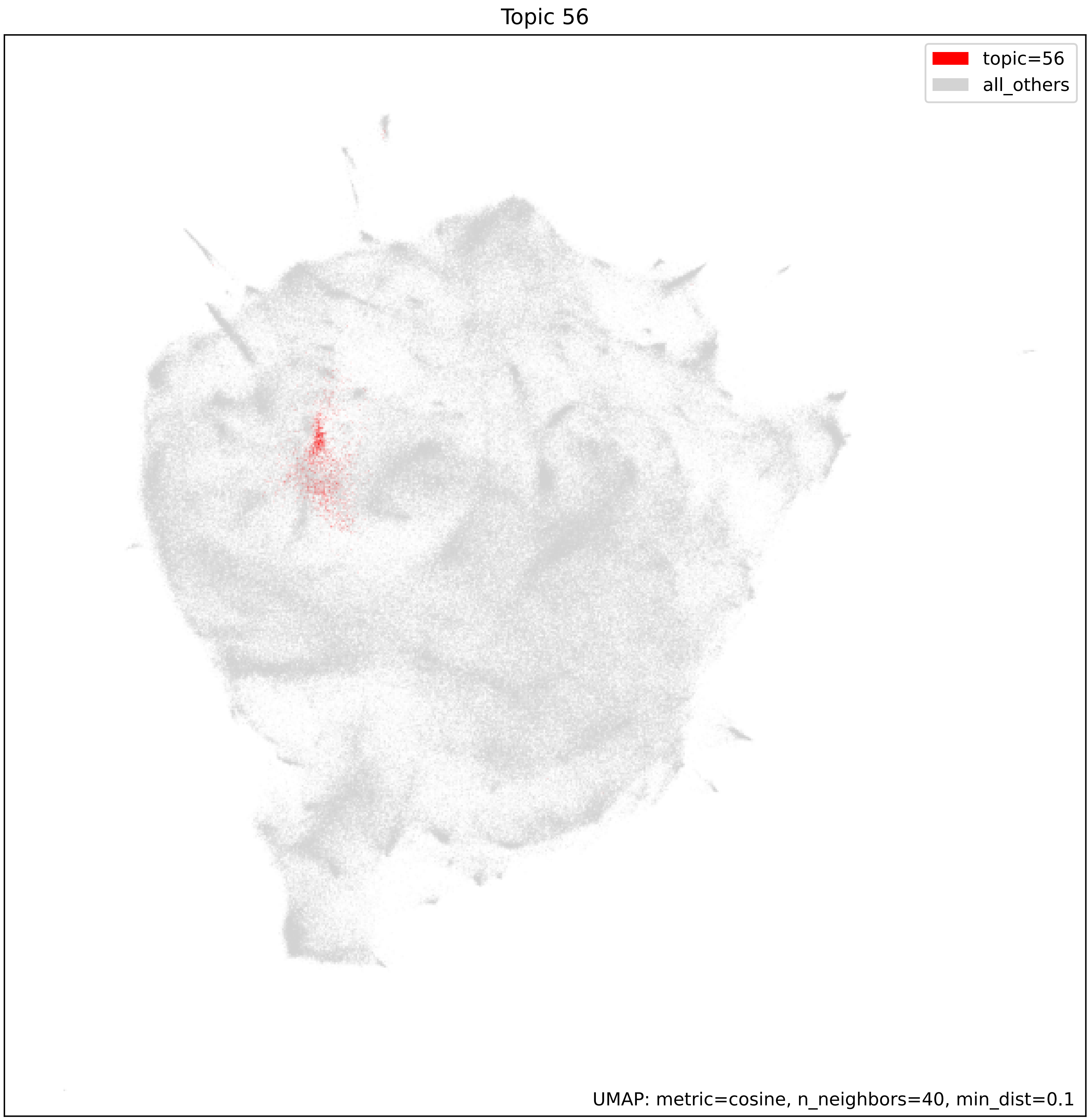
Topic 56
topic=56
all_others
UMAP: metric=cosine, n_neighbors=40, min_dist=0.1

Topic 57

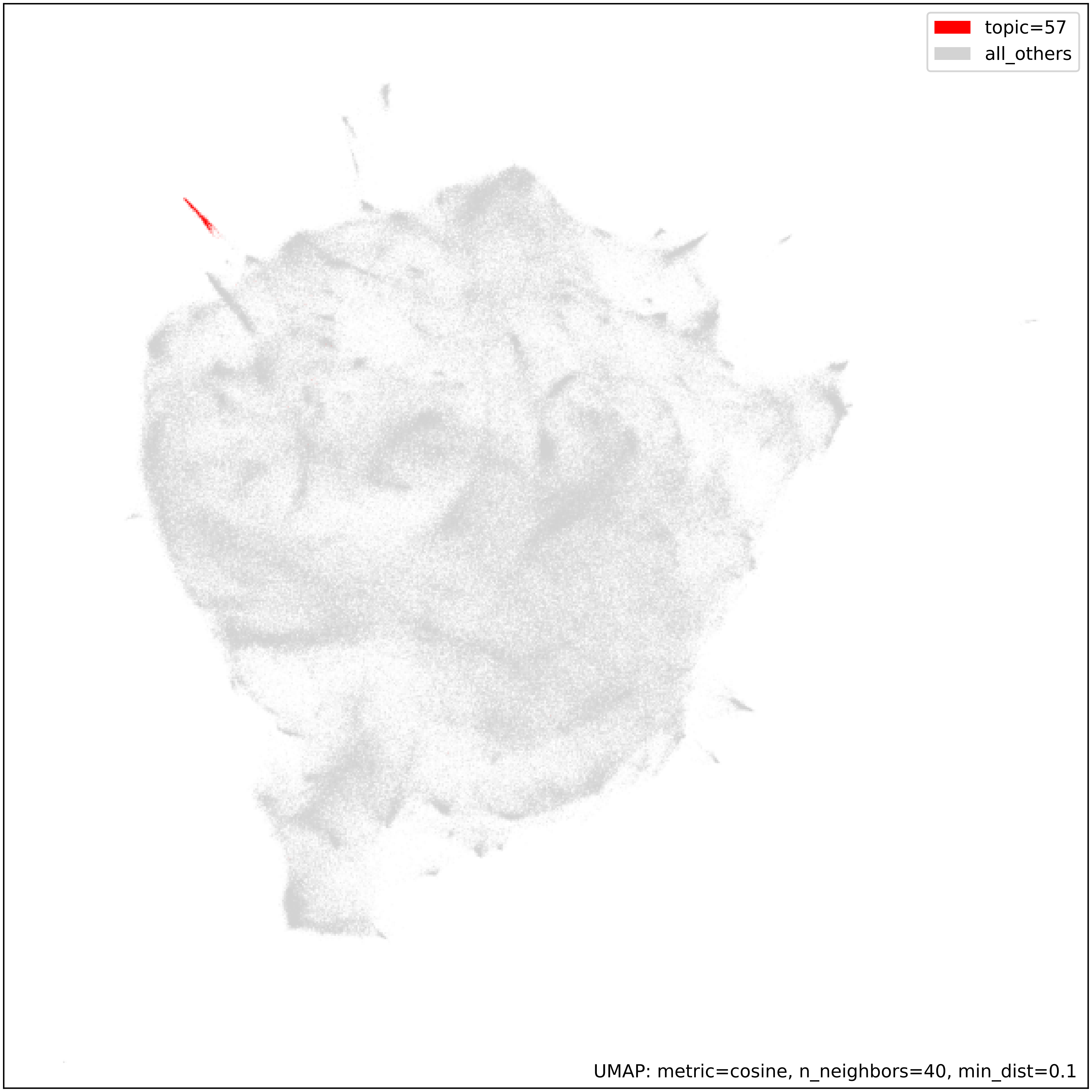
topic=57
all_others
UMAP: metric=cosine, n_neighbors=40, min_dist=0.1

Topic 58

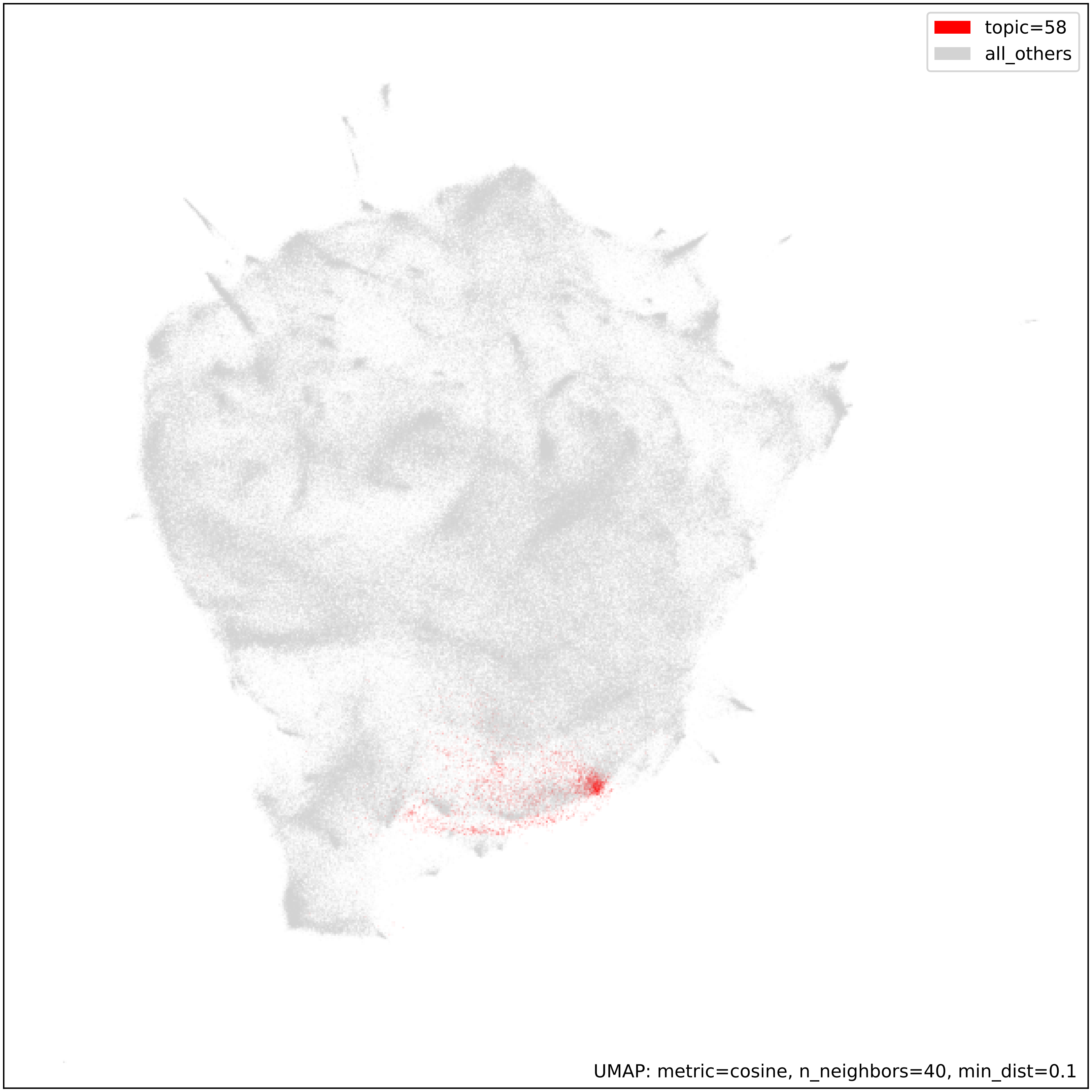
topic=58
all_others
UMAP: metric=cosine, n_neighbors=40, min_dist=0.1

Topic 59

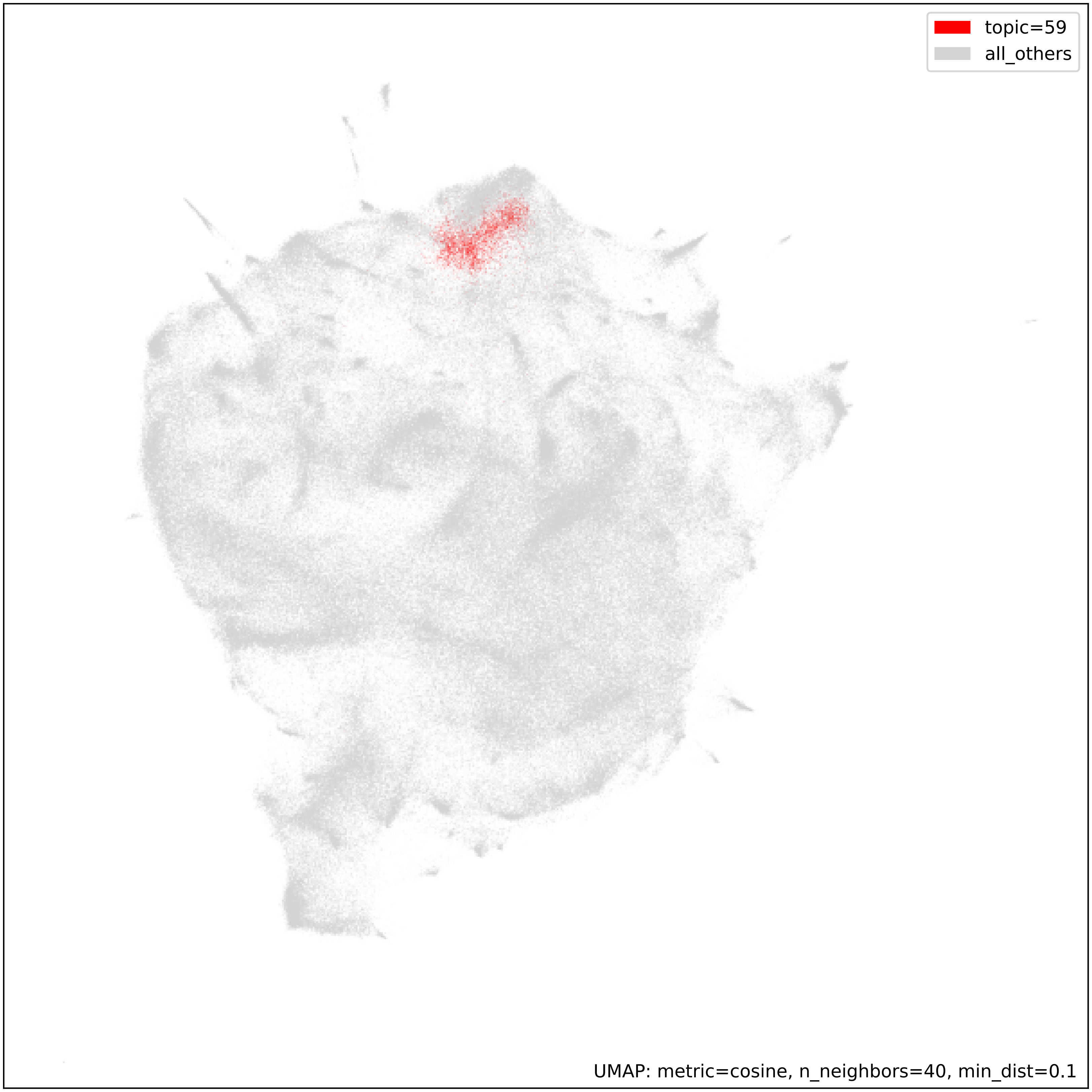

topic=59
all_others
UMAP: metric=cosine, n_neighbors=40, min_dist=0.1

Topic 60

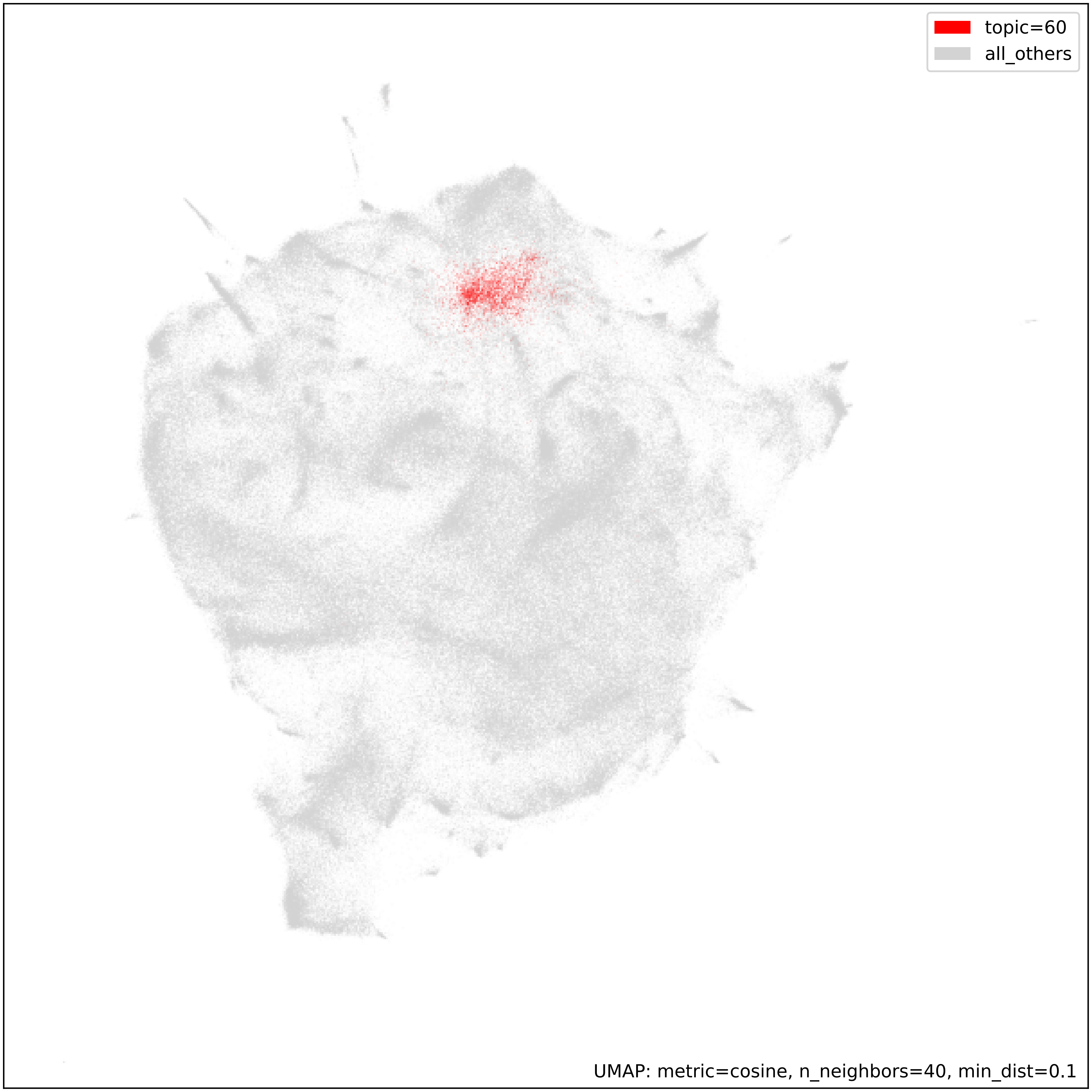

topic=60
all_others
UMAP: metric=cosine, n_neighbors=40, min_dist=0.1

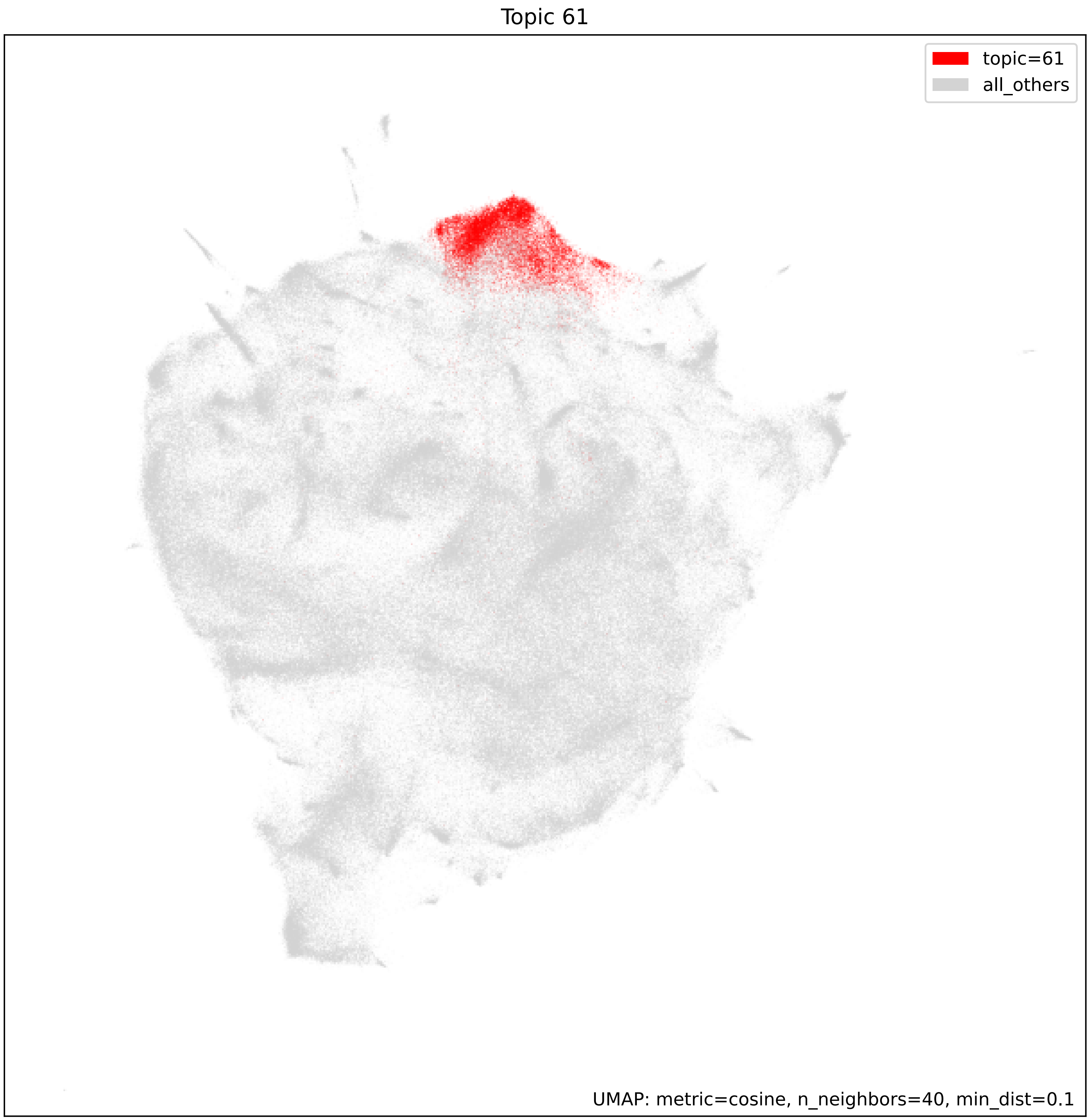
Topic 61
topic=61
all_others
UMAP: metric=cosine, n_neighbors=40, min_dist=0.1

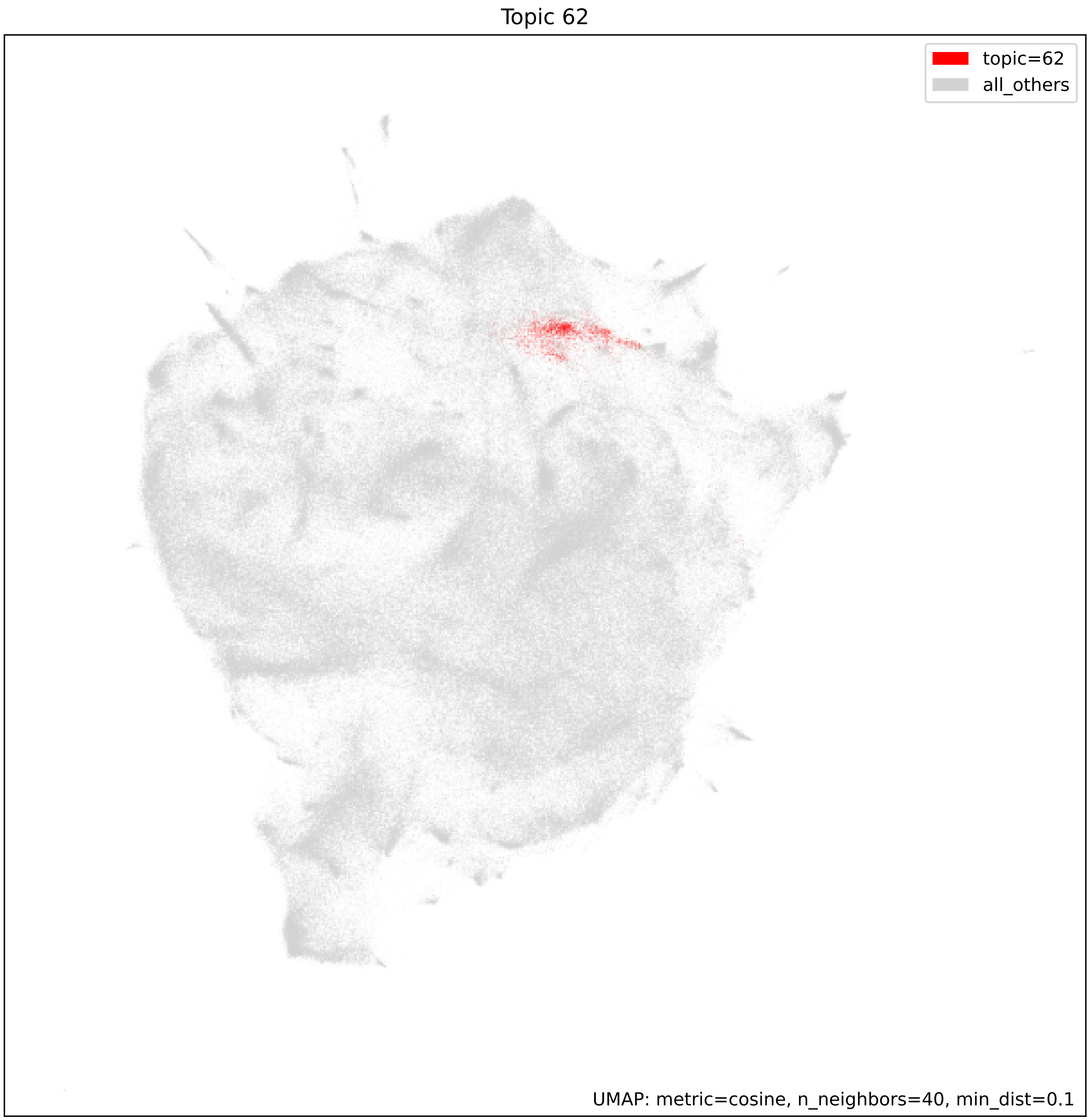
Topic 62
topic=62
all_others
UMAP: metric=cosine, n_neighbors=40, min_dist=0.1

Topic 63

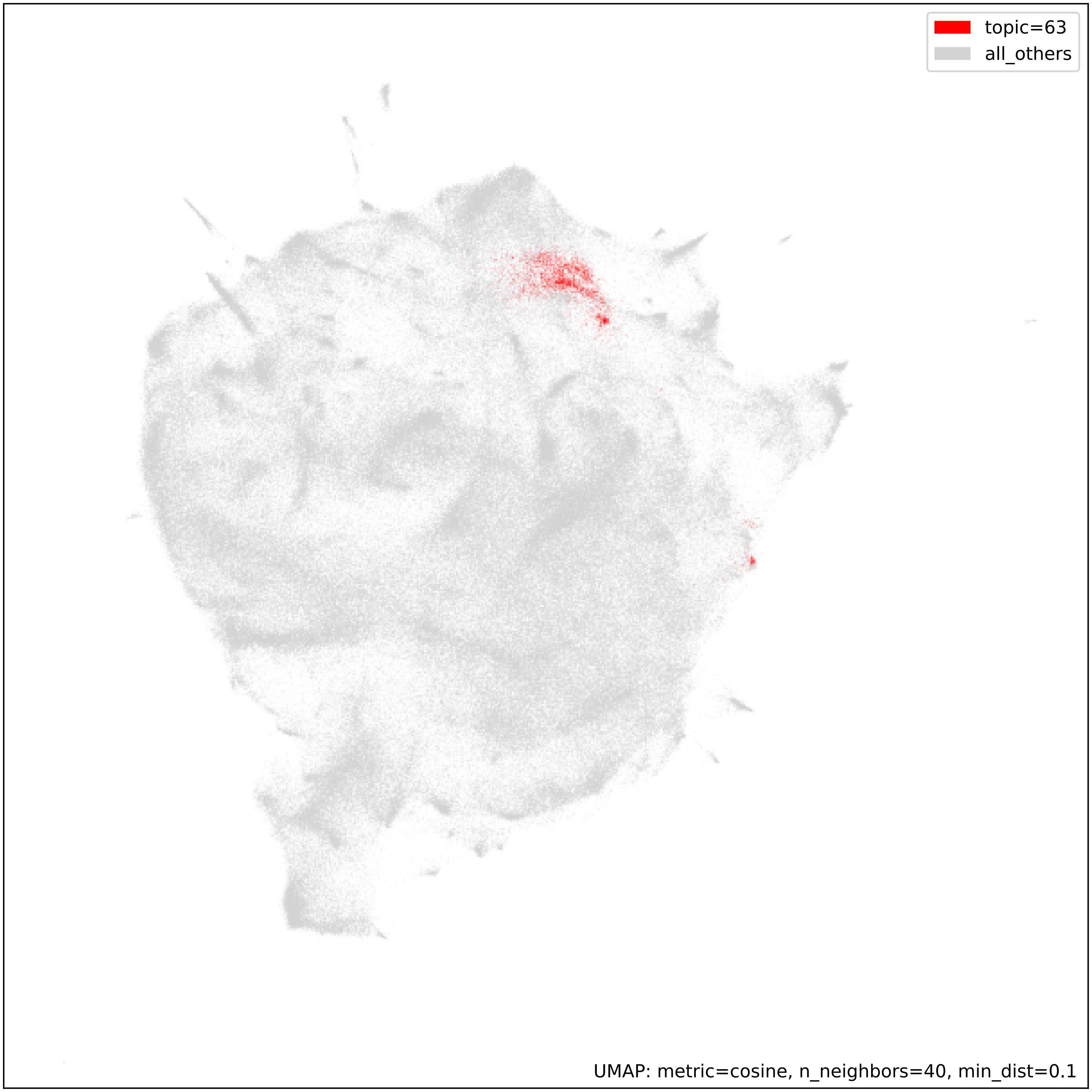

topic=63
all_others
UMAP: metric=cosine, n_neighbors=40, min_dist=0.1

Topic 64

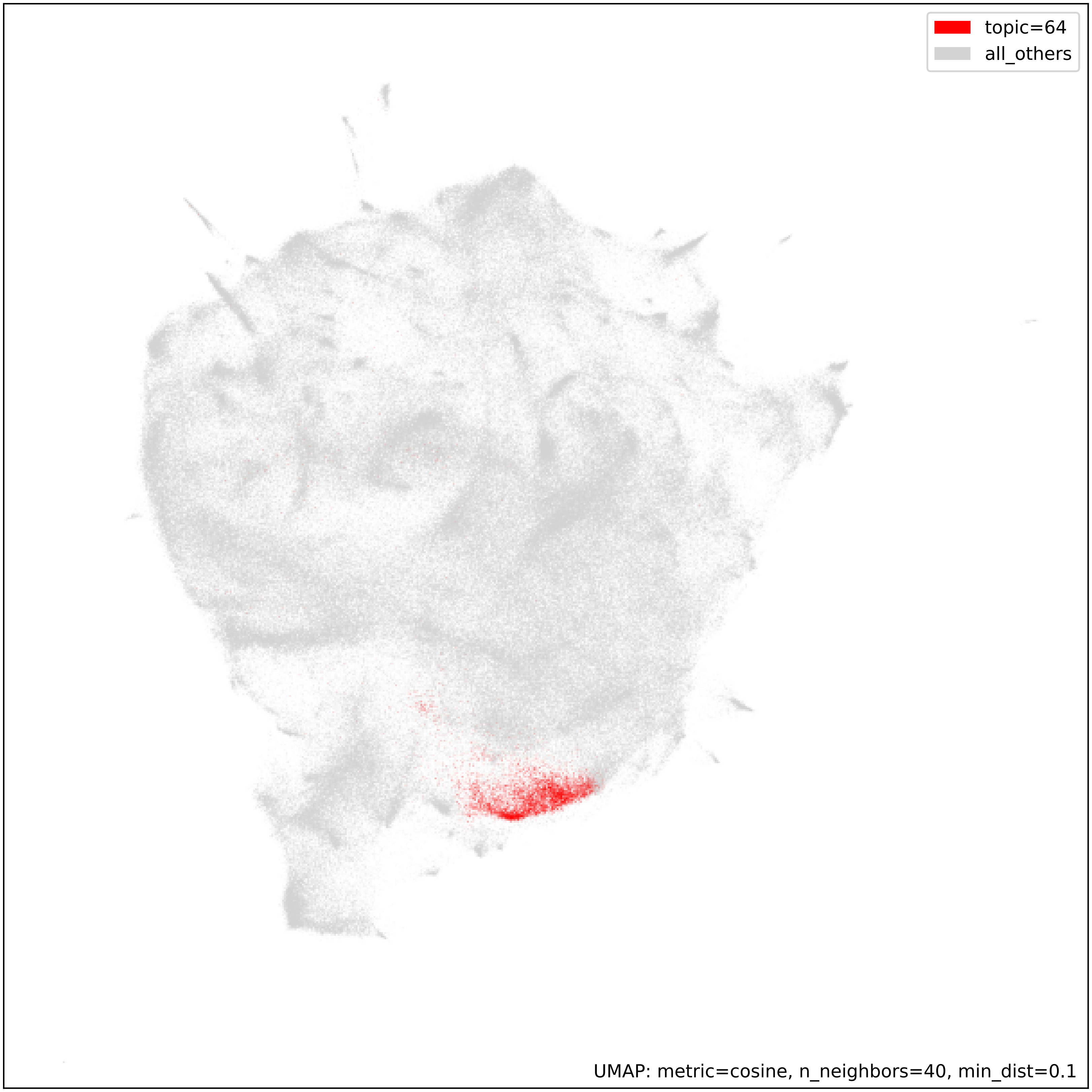
topic=64
all_others
UMAP: metric=cosine, n_neighbors=40, min_dist=0.1

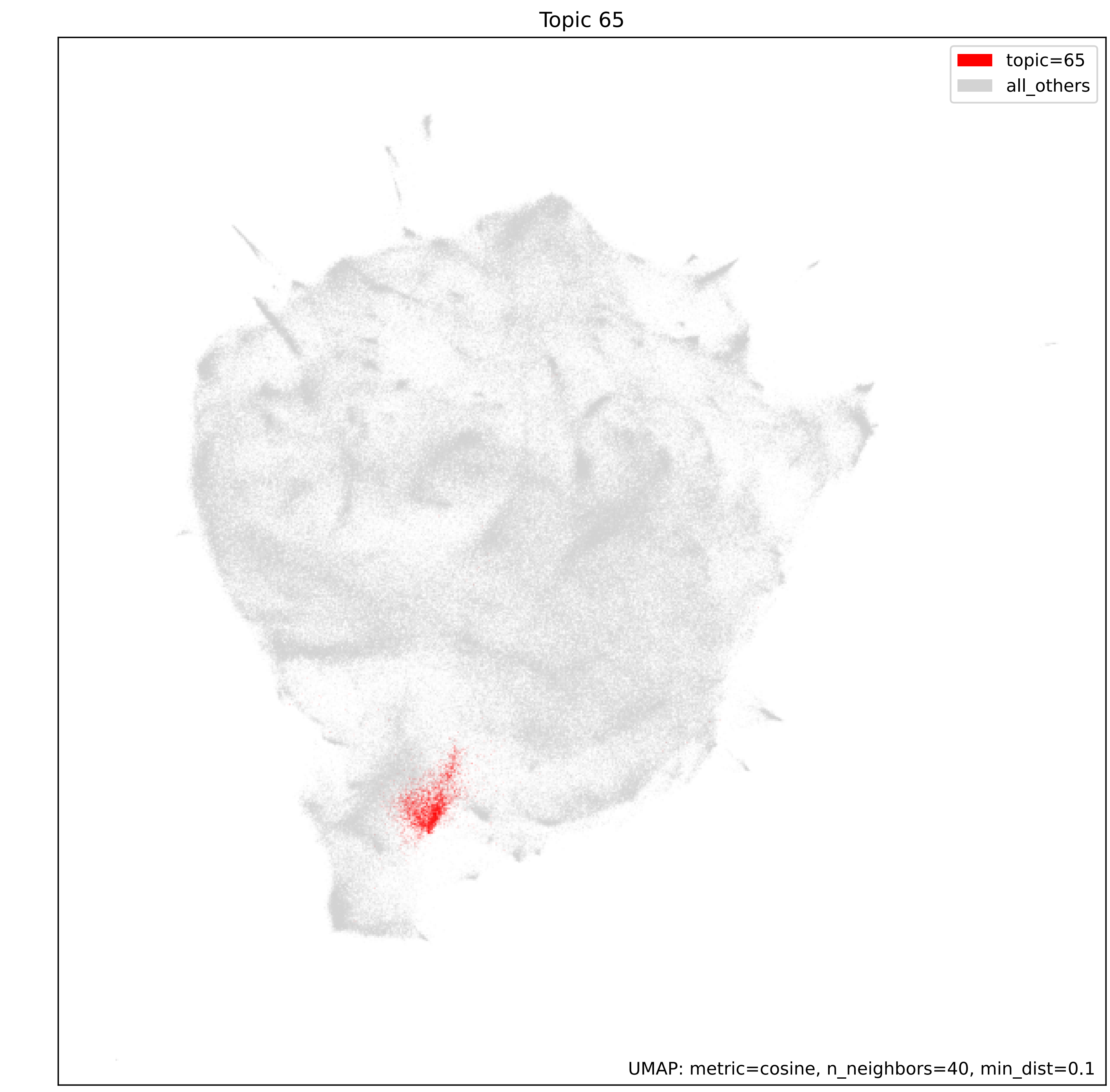
Topic 65
topic=65
all_others
UMAP: metric=cosine, n_neighbors=40, min_dist=0.1

Topic 66

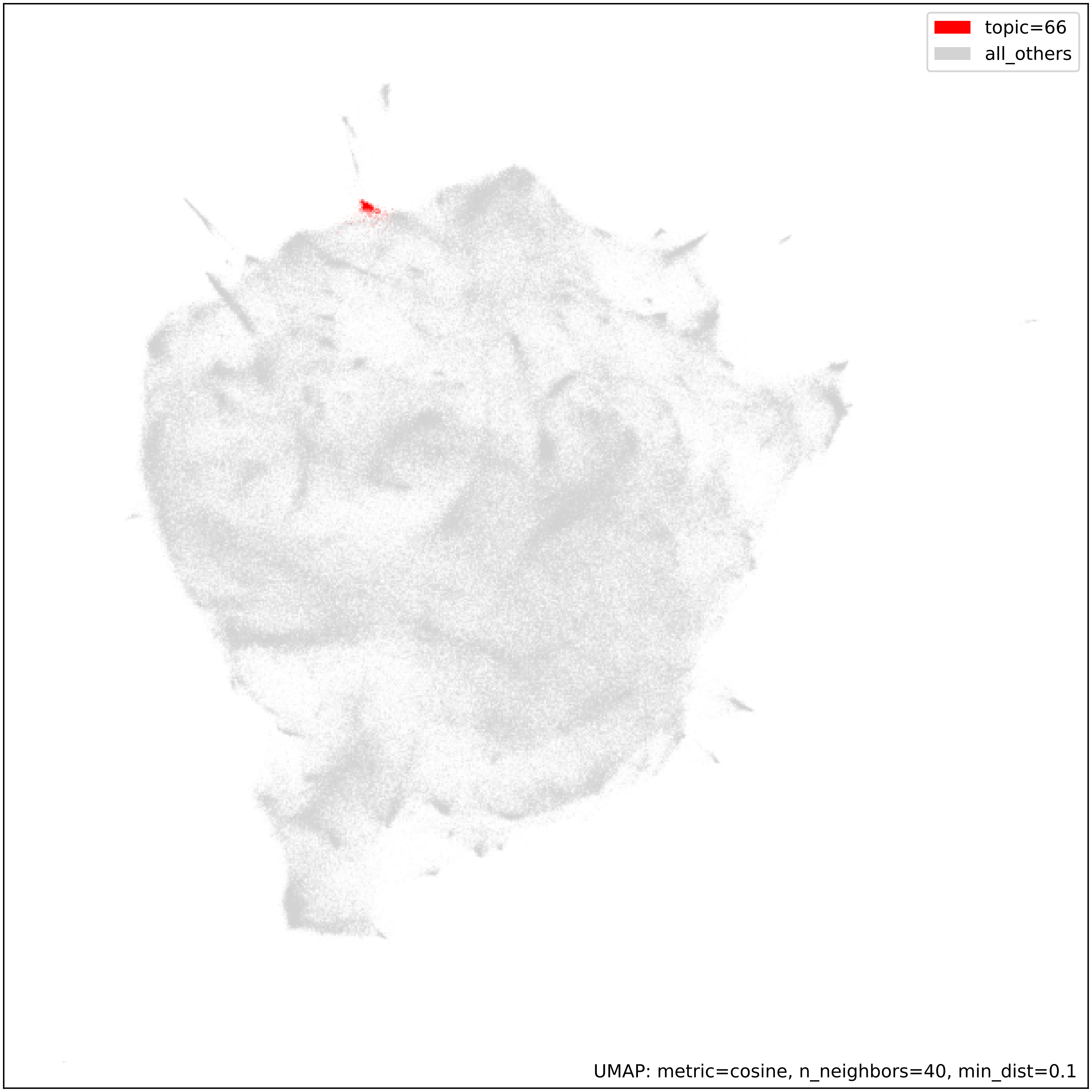
topic=66
all_others
UMAP: metric=cosine, n_neighbors=40, min_dist=0.1

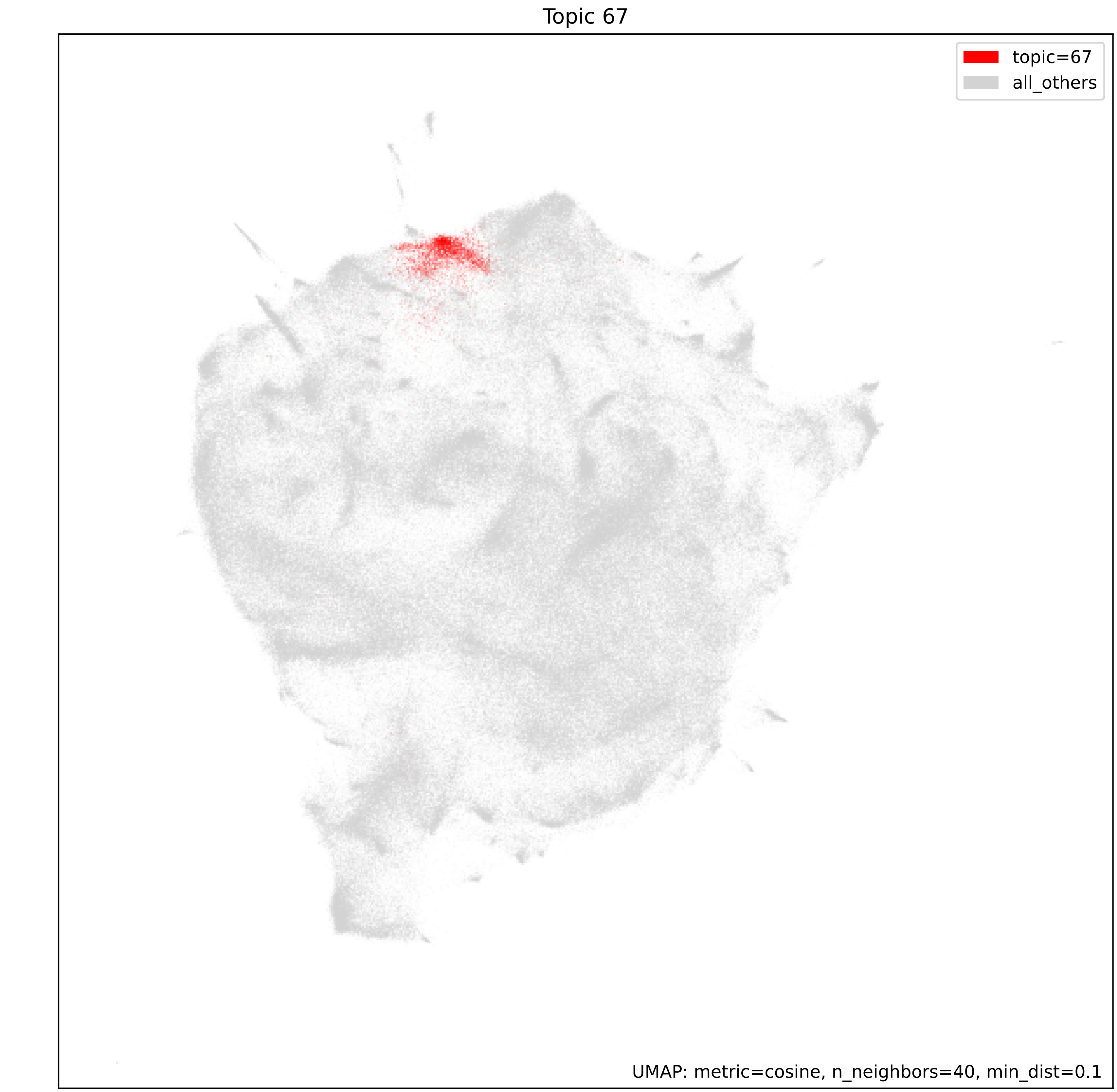
Topic 67
topic=67
all_others
UMAP: metric=cosine, n_neighbors=40, min_dist=0.1

Topic 68

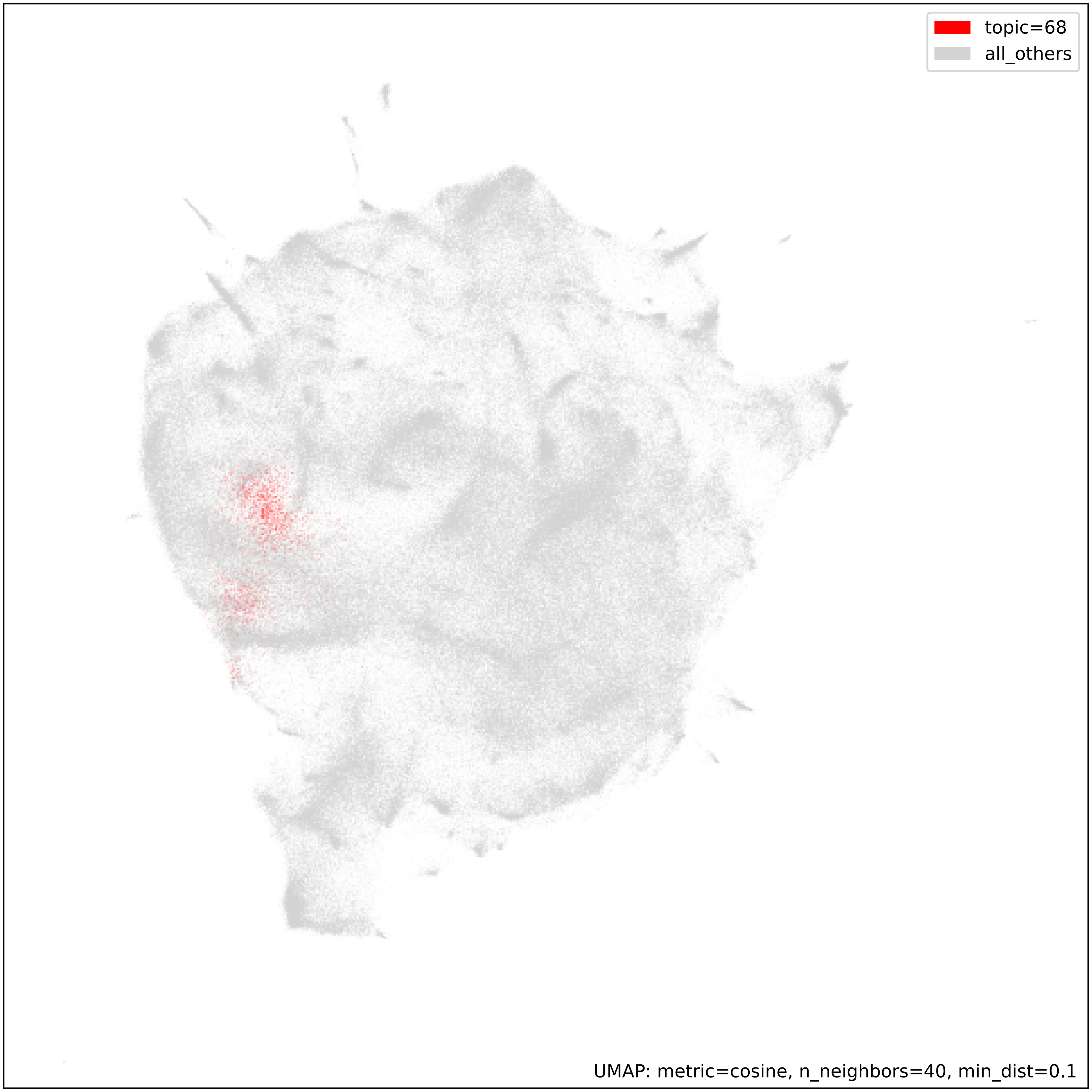

topic=68
all_others
UMAP: metric=cosine, n_neighbors=40, min_dist=0.1

Topic 69

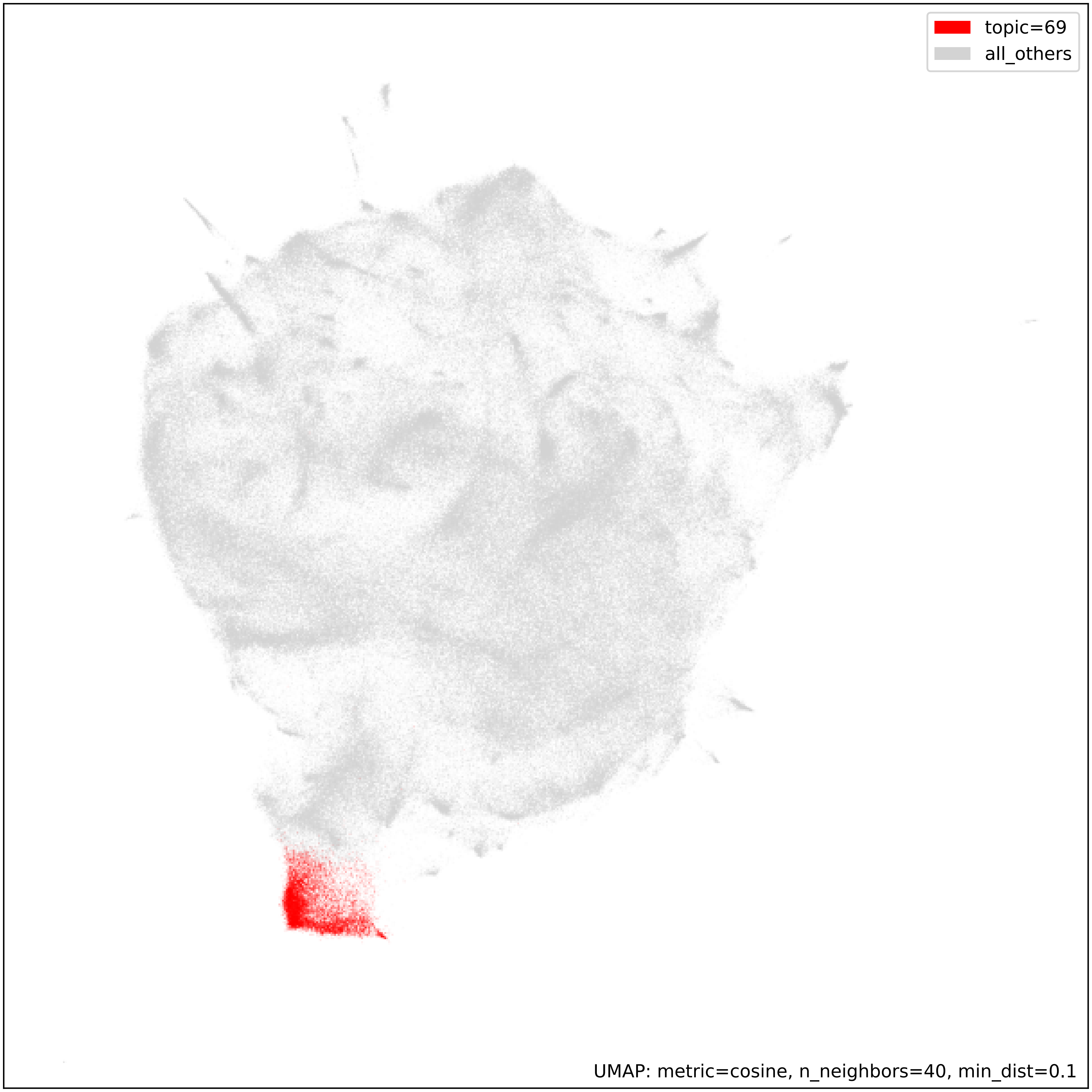

topic=69
all_others
UMAP: metric=cosine, n_neighbors=40, min_dist=0.1

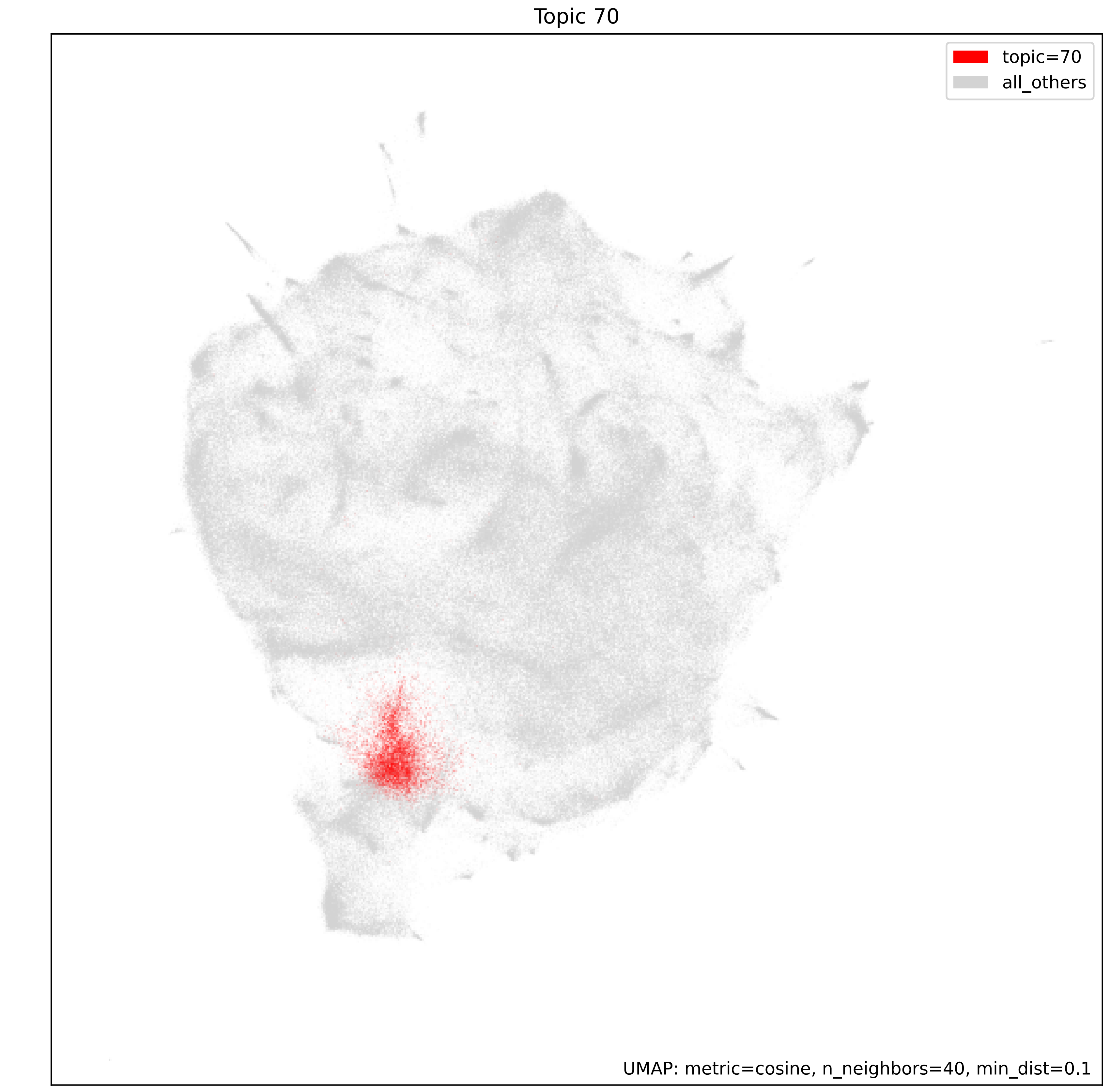

Topic 70
topic=70
all_others
UMAP: metric=cosine, n_neighbors=40, min_dist=0.1

Topic 71

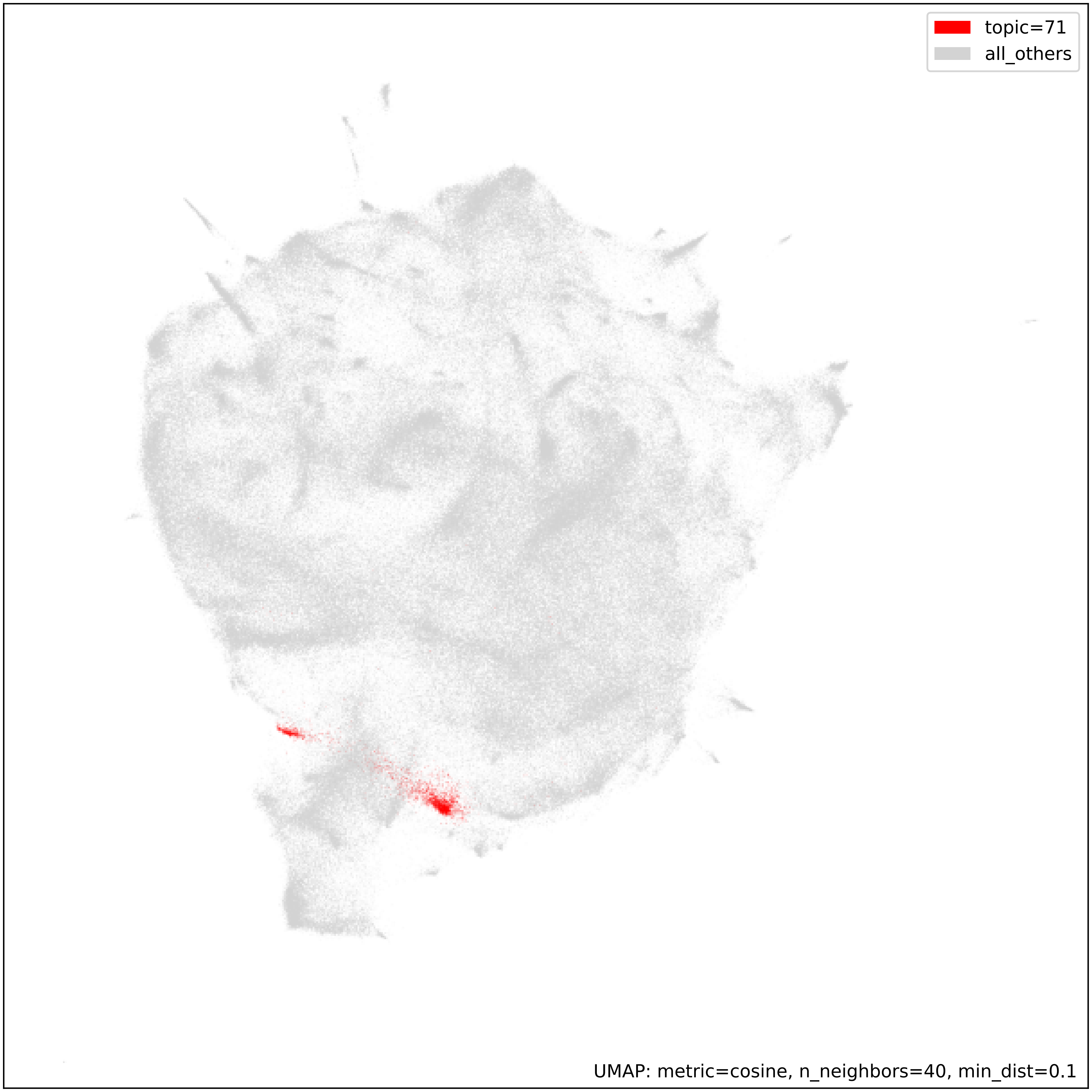
topic=71
all_others
UMAP: metric=cosine, n_neighbors=40, min_dist=0.1

Topic 72

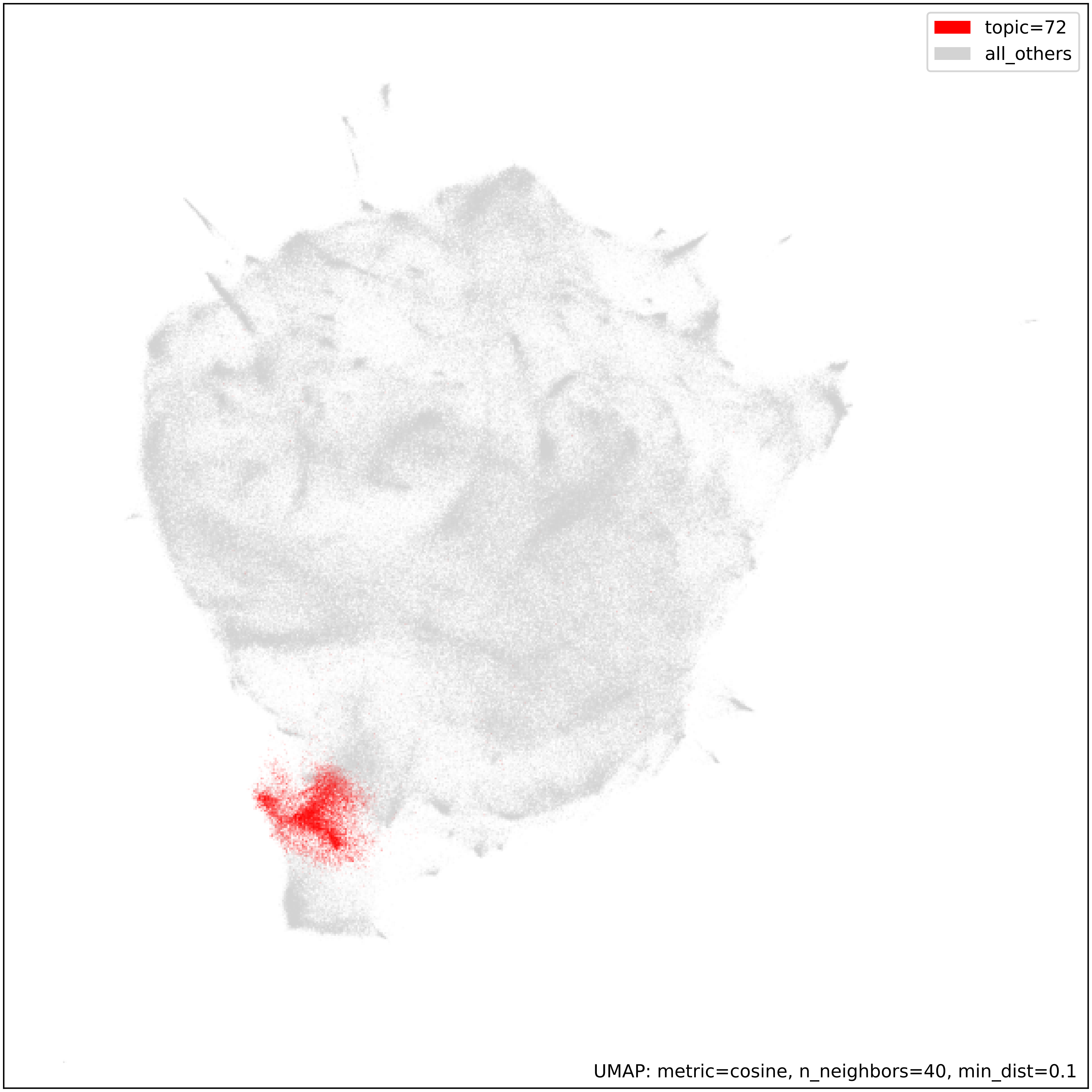
topic=72
all_others
UMAP: metric=cosine, n_neighbors=40, min_dist=0.1

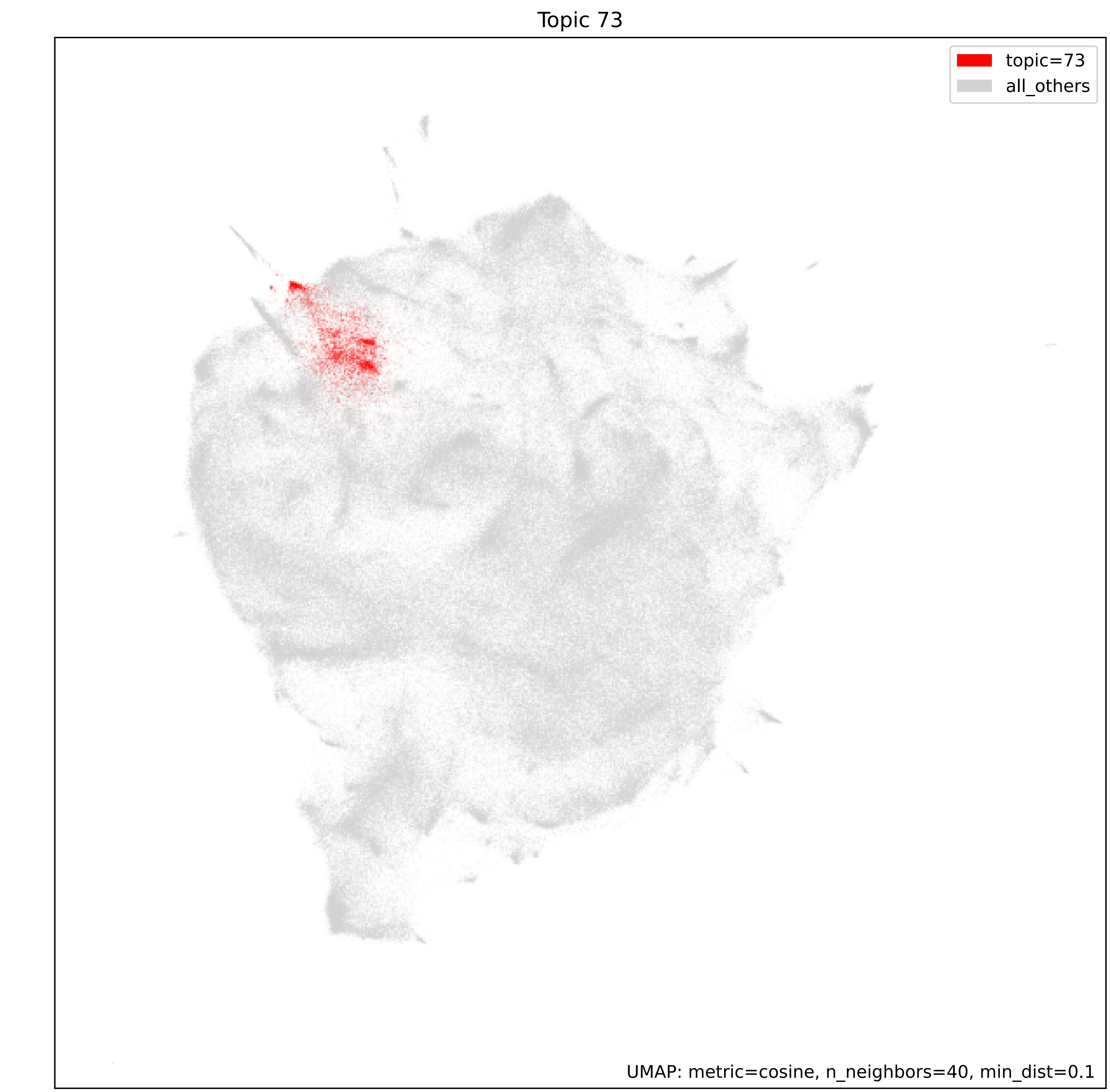

Topic 73
topic=73
all_others
UMAP: metric=cosine, n_neighbors=40, min_dist=0.1

Topic 74

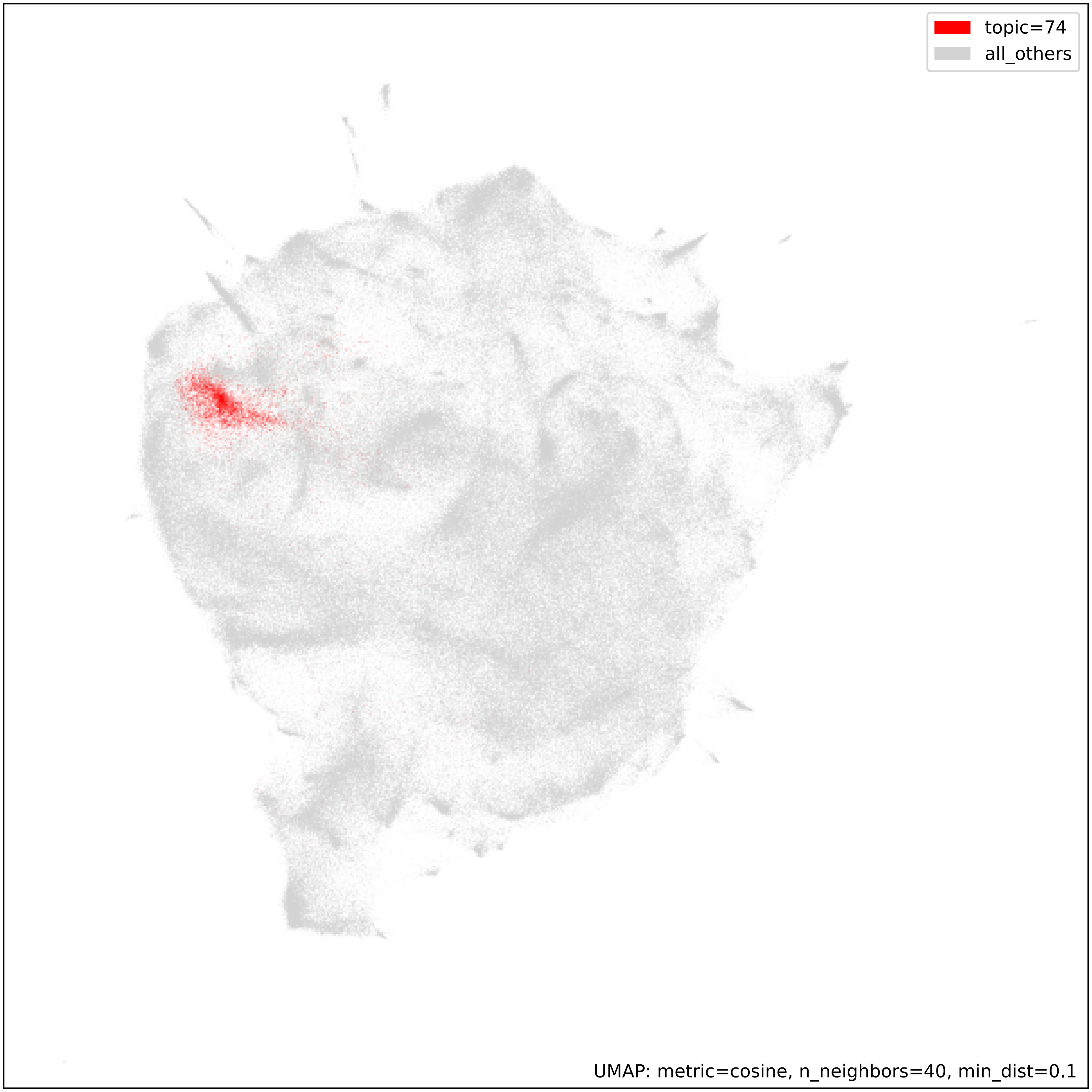

topic=74
all_others
UMAP: metric=cosine, n_neighbors=40, min_dist=0.1

Topic 75

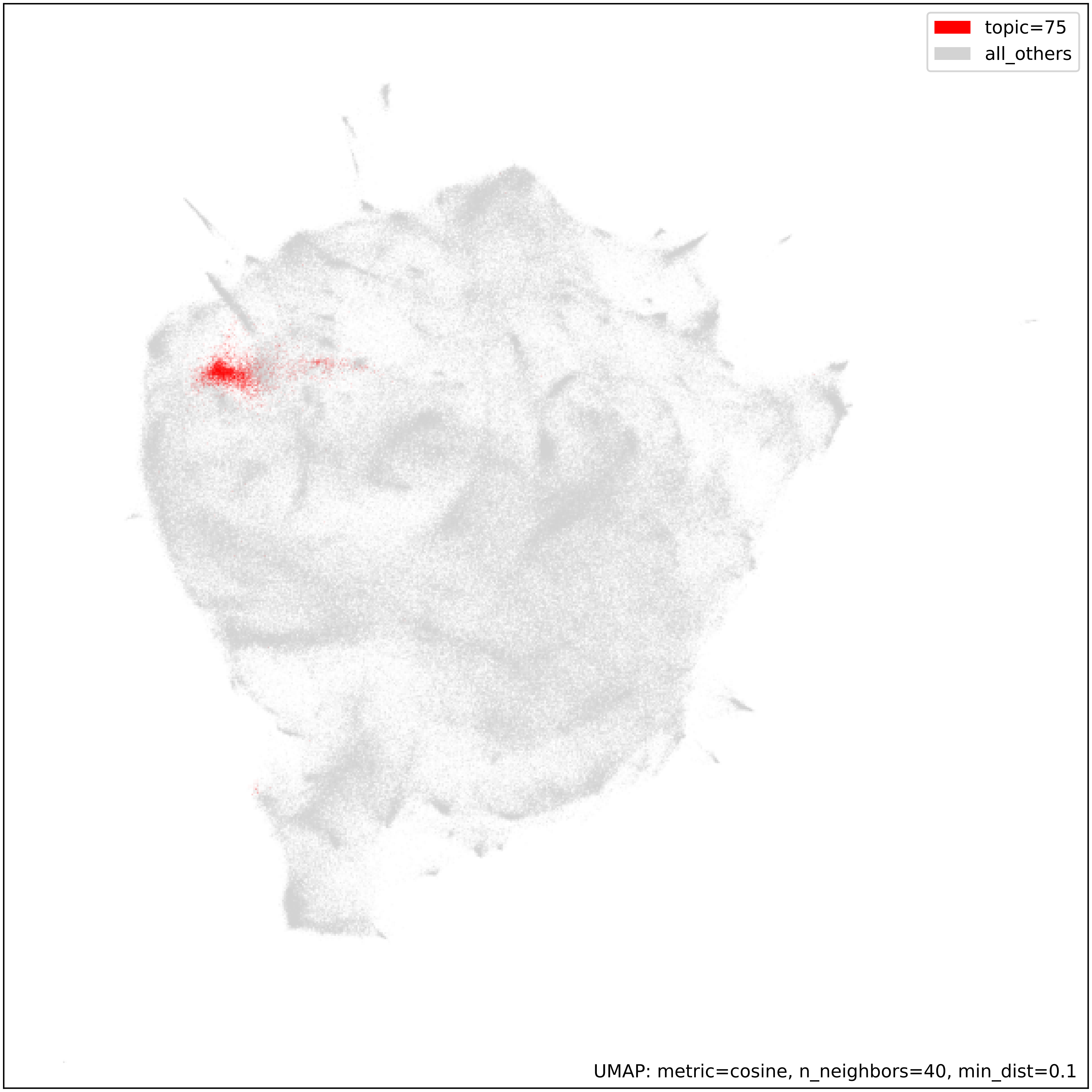
topic=75
all_others
UMAP: metric=cosine, n_neighbors=40, min_dist=0.1

Topic 76

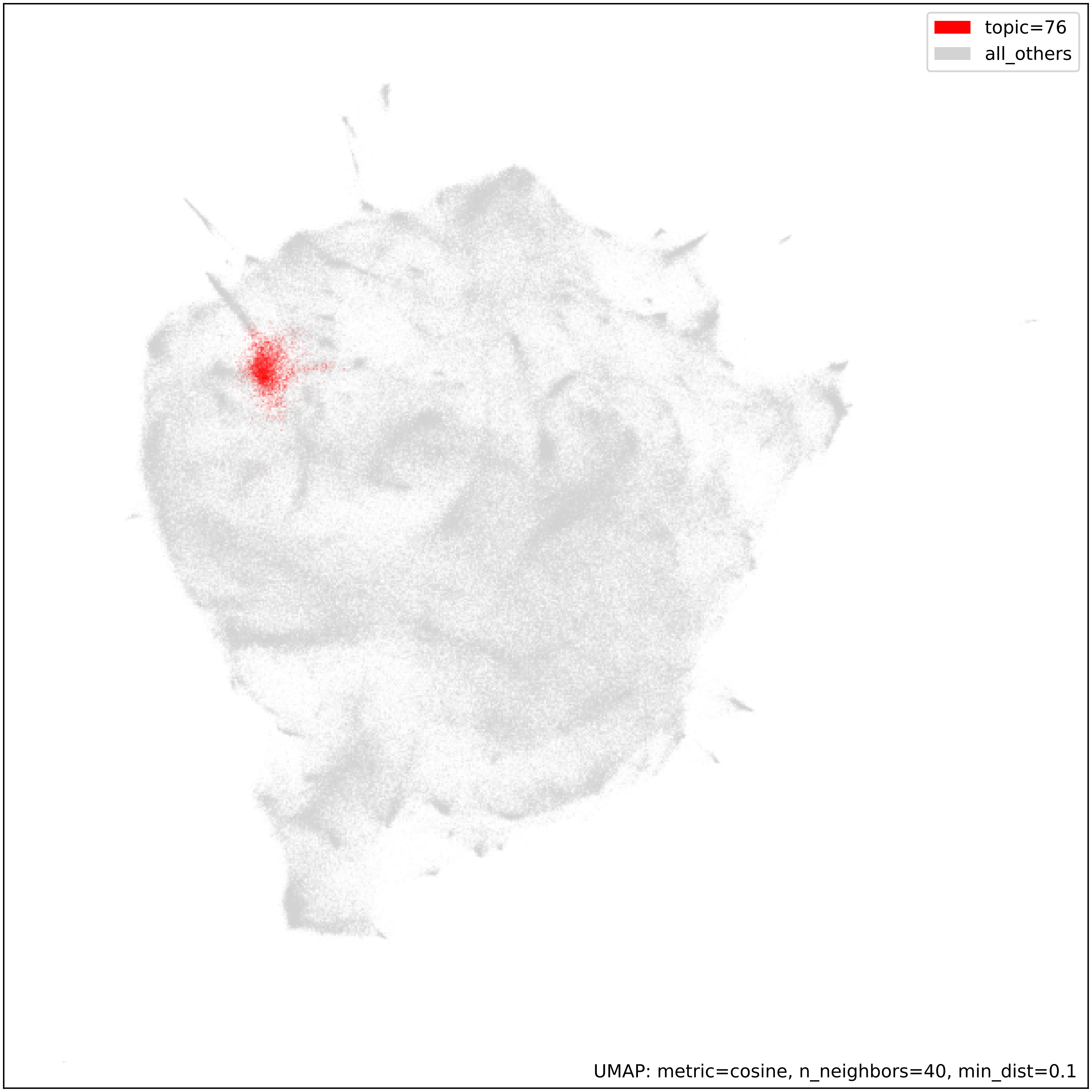
topic=76
all_others
UMAP: metric=cosine, n_neighbors=40, min_dist=0.1

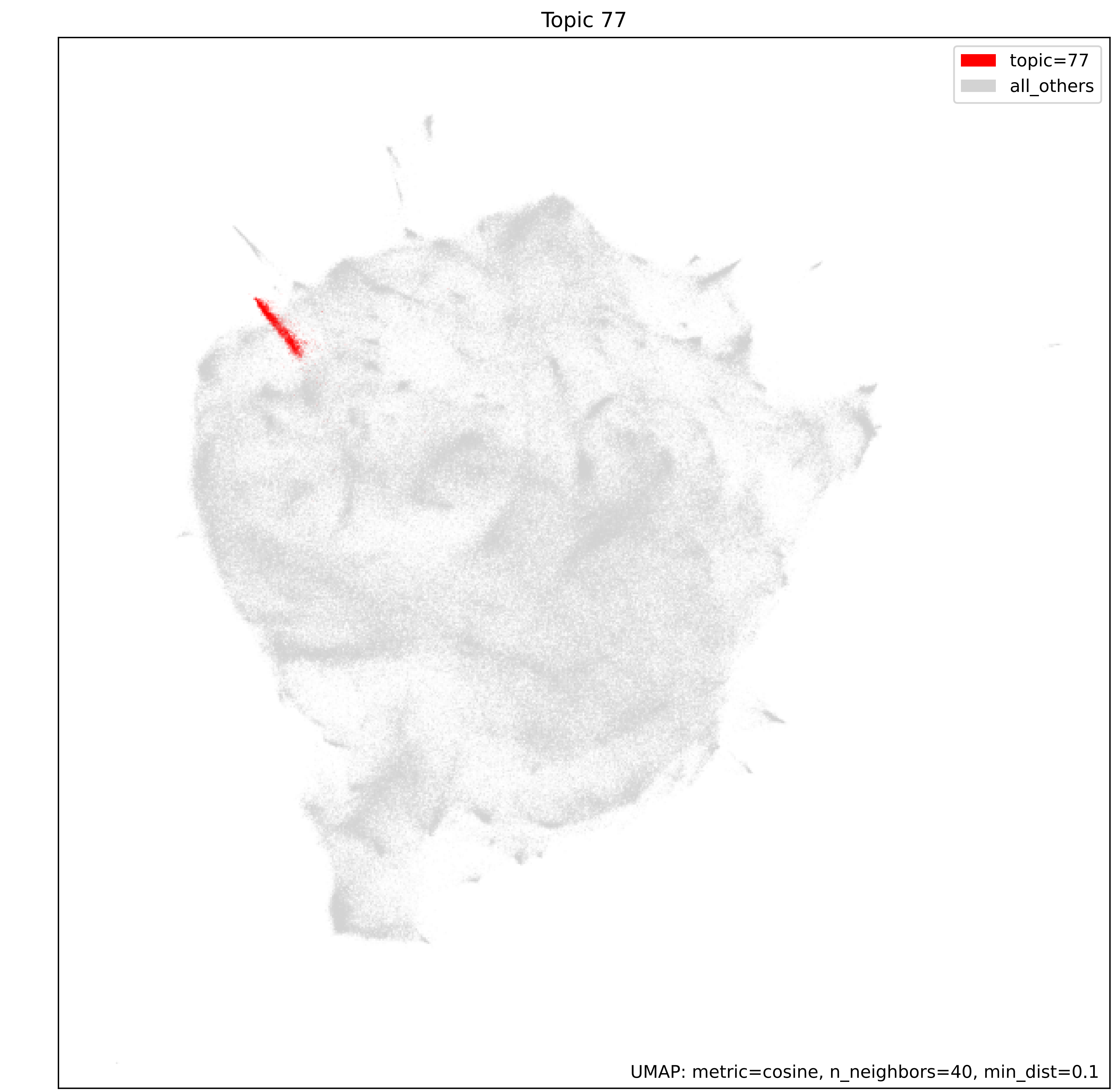
Topic 77
topic=77
all_others
UMAP: metric=cosine, n_neighbors=40, min_dist=0.1

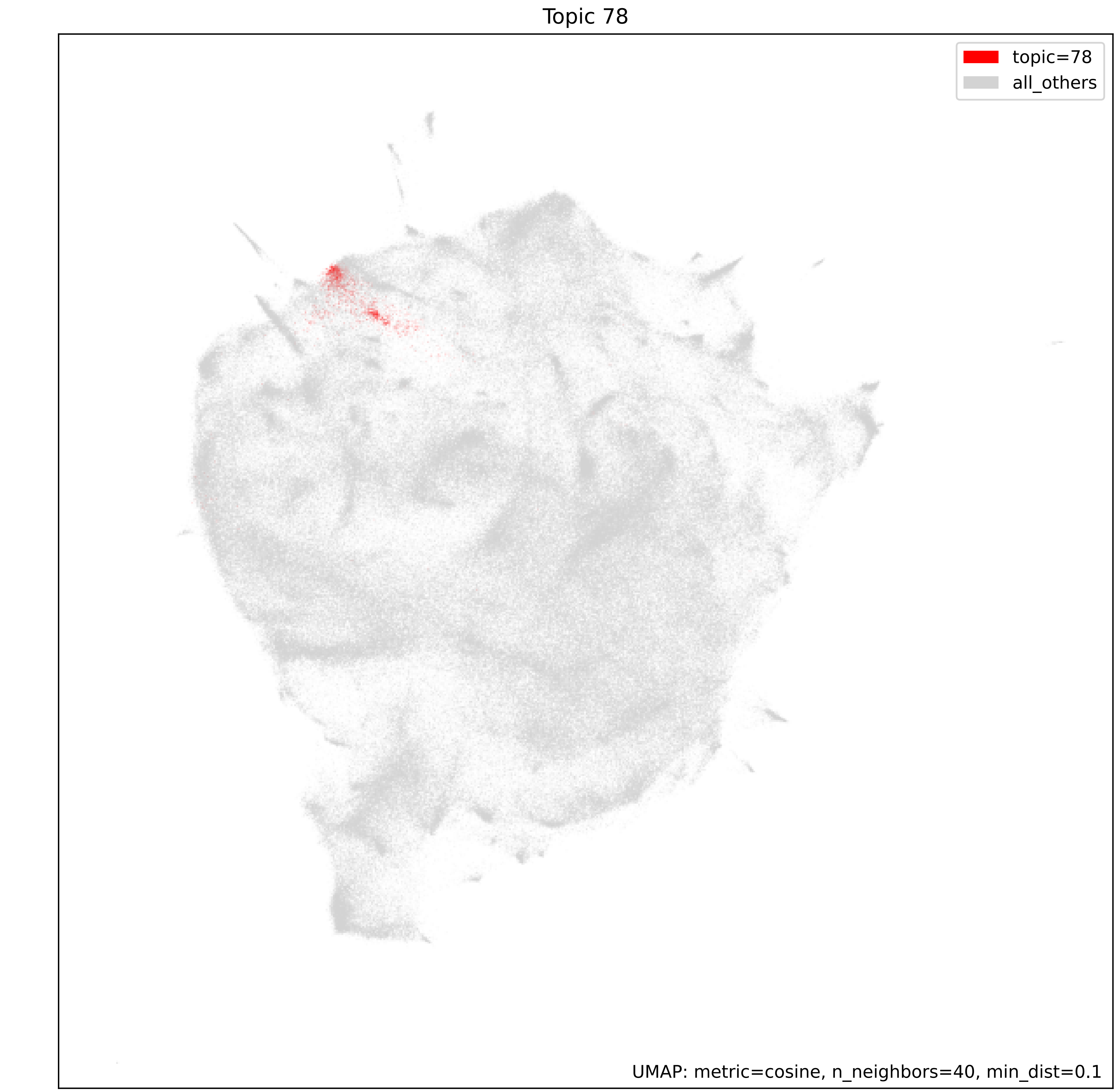
Topic 78
topic=78
all_others
UMAP: metric=cosine, n_neighbors=40, min_dist=0.1

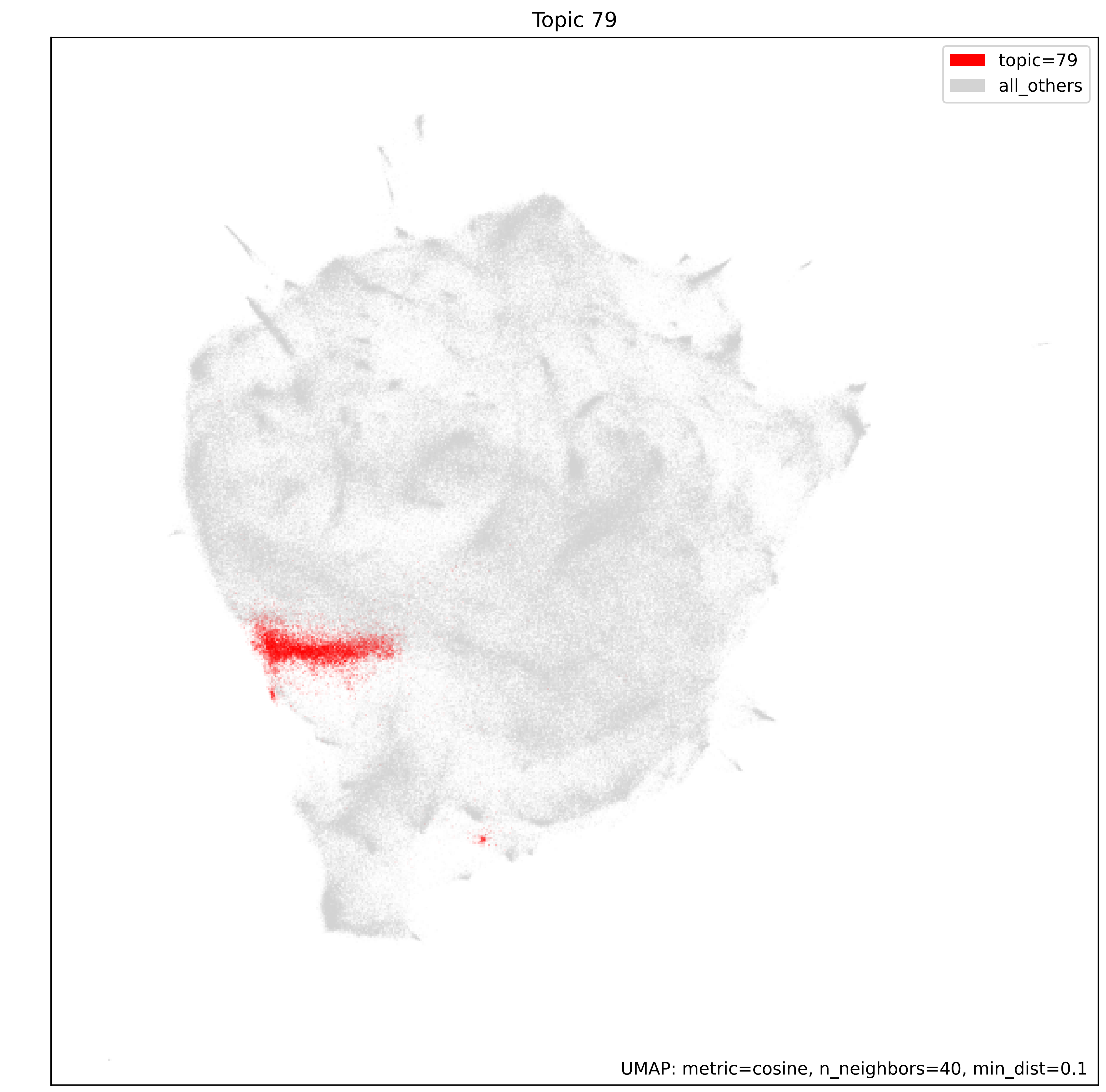
Topic 79
topic=79
all_others
UMAP: metric=cosine, n_neighbors=40, min_dist=0.1

Topic 80

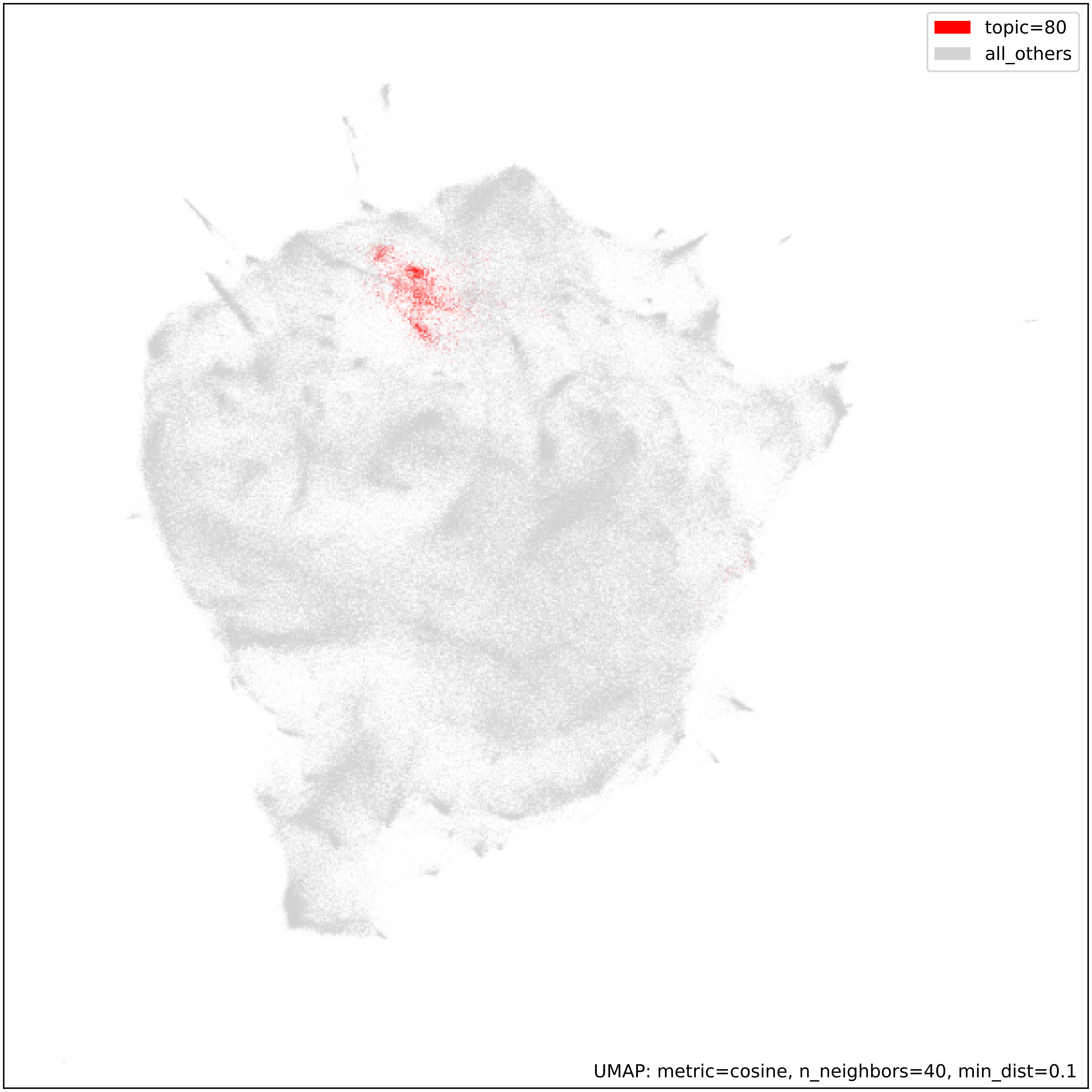
topic=80
all_others
UMAP: metric=cosine, n_neighbors=40, min_dist=0.1

Topic 81

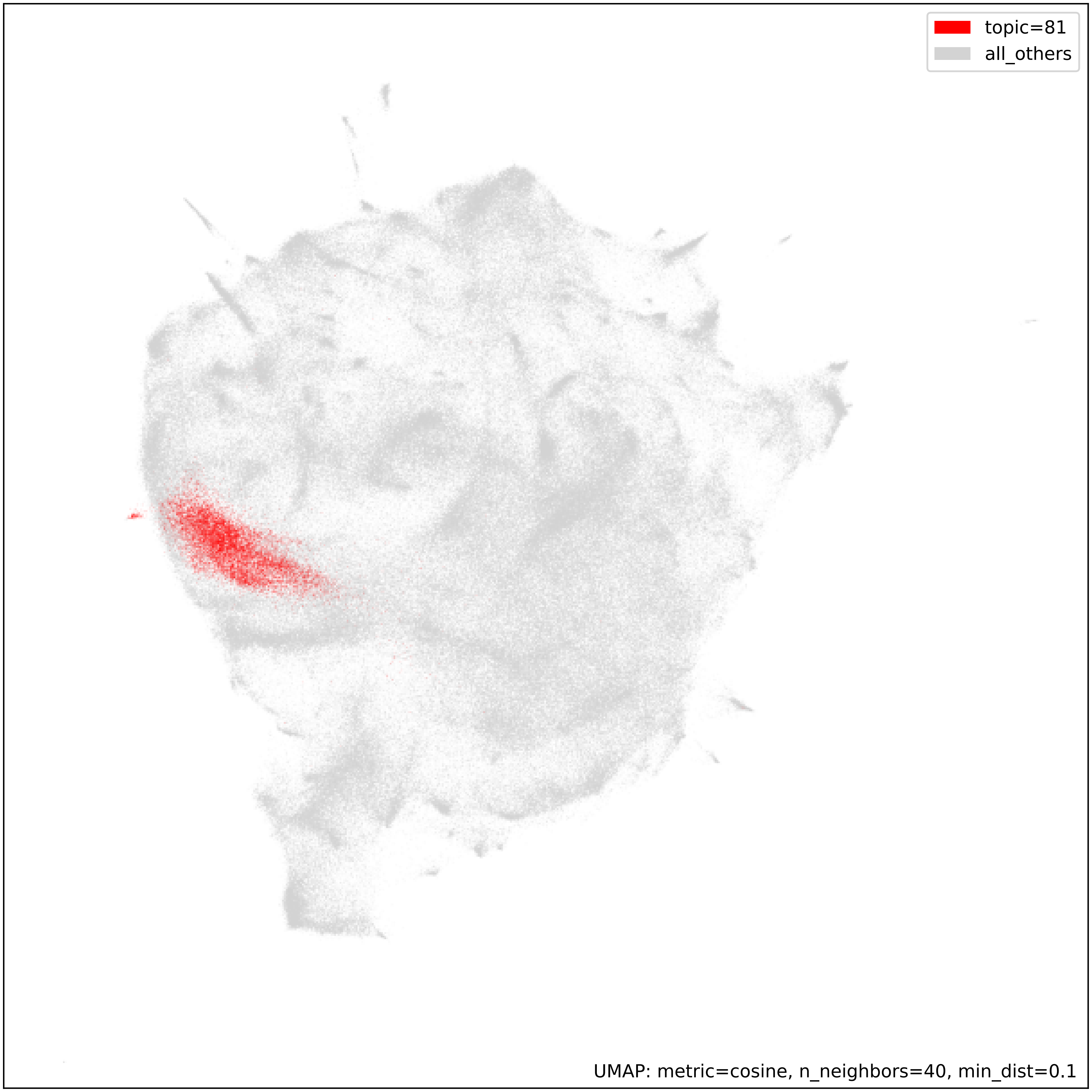
topic=81
all_others
UMAP: metric=cosine, n_neighbors=40, min_dist=0.1

Topic 82

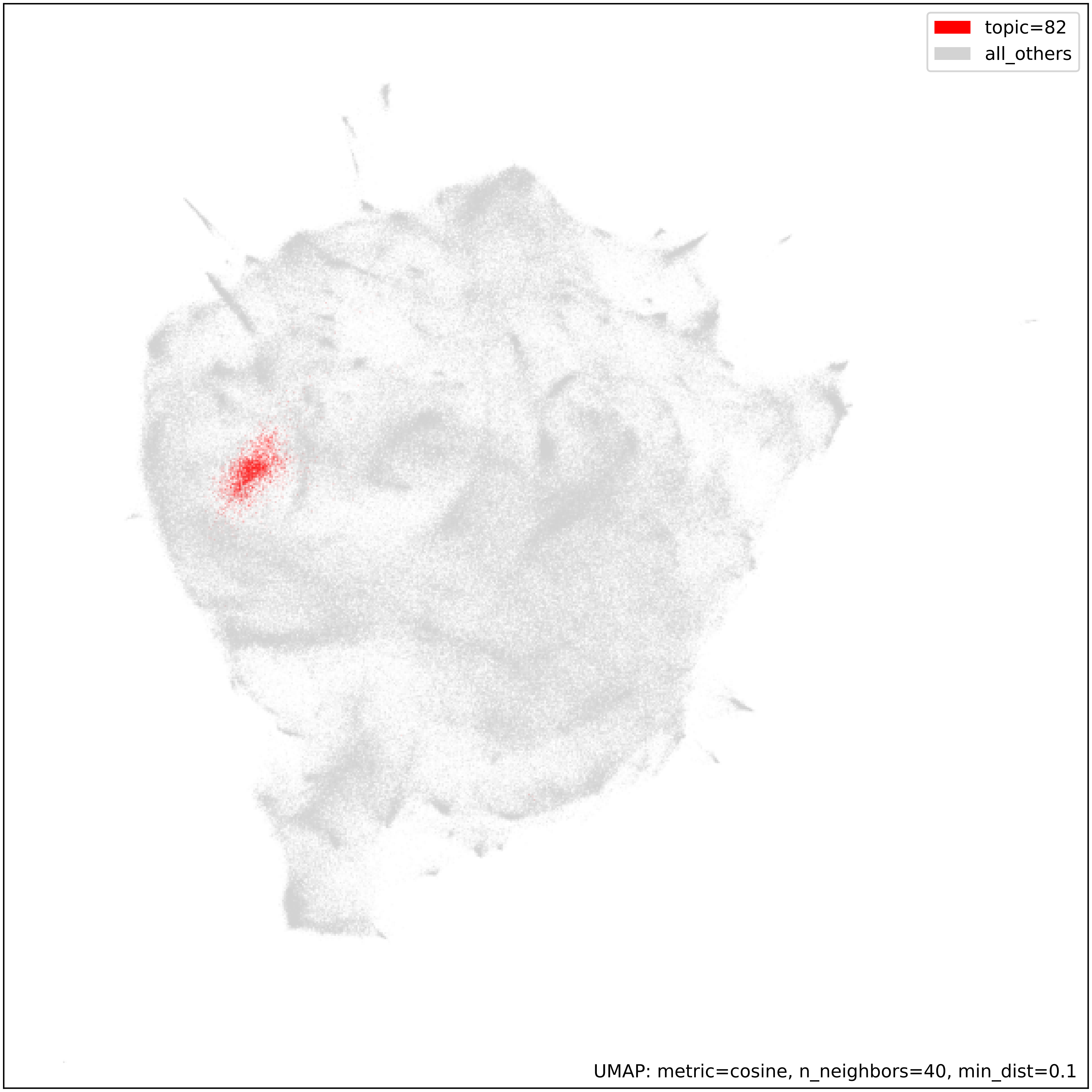
topic=82
all_others
UMAP: metric=cosine, n_neighbors=40, min_dist=0.1

Topic 83

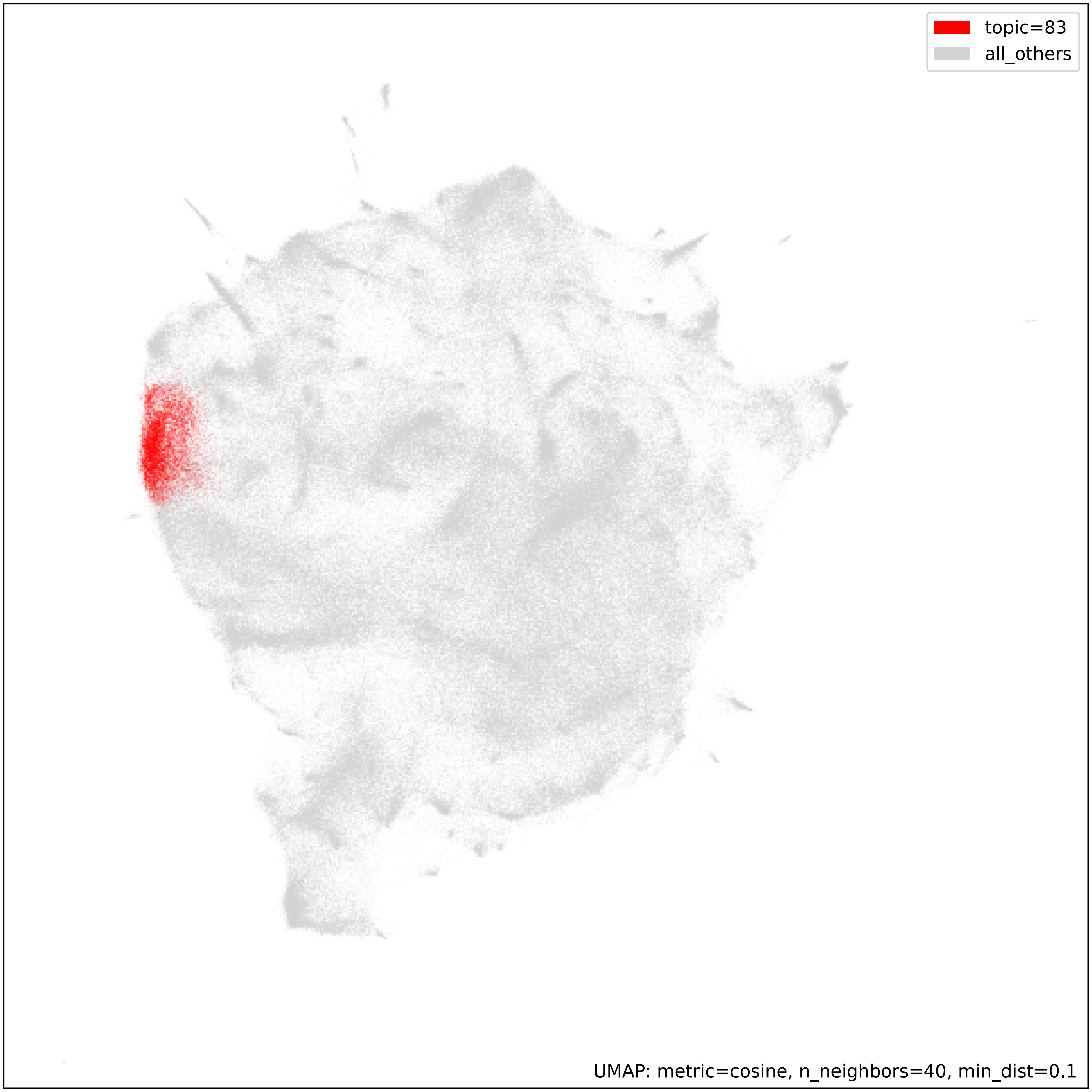
topic=83
all_others
UMAP: metric=cosine, n_neighbors=40, min_dist=0.1

Topic 84

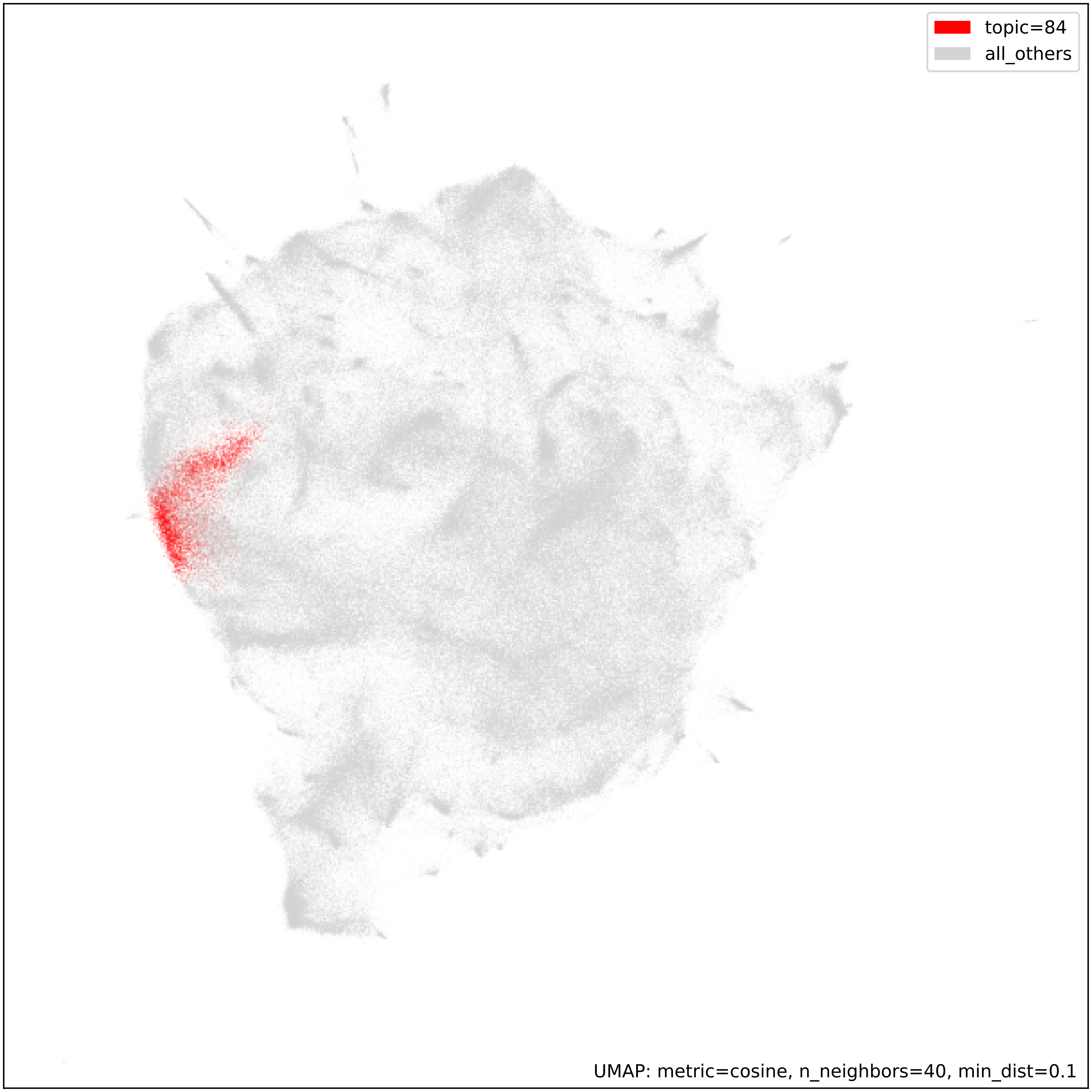
topic=84
all_others
UMAP: metric=cosine, n_neighbors=40, min_dist=0.1

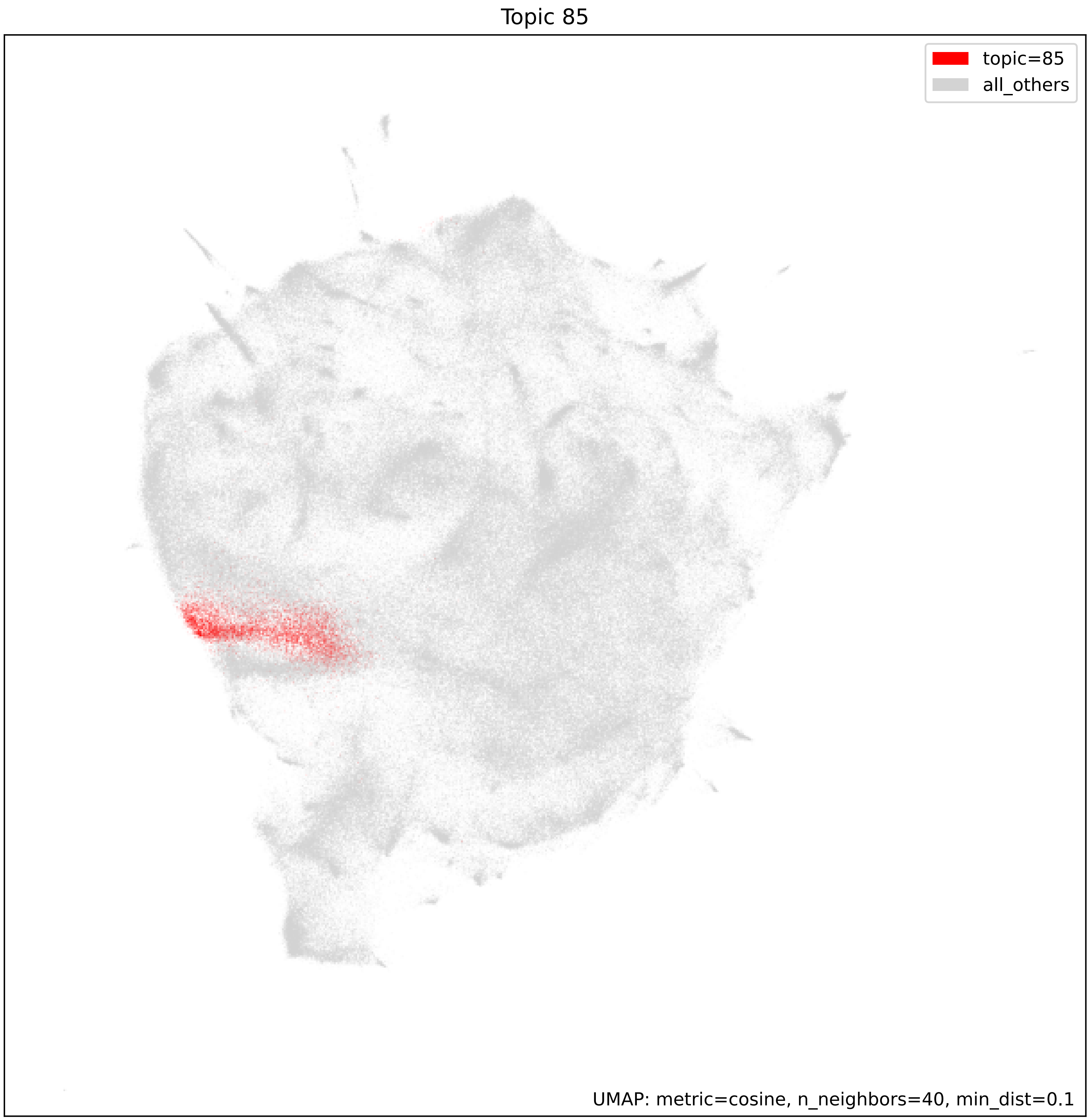
Topic 85
topic=85
all_others
UMAP: metric=cosine, n_neighbors=40, min_dist=0.1

Topic 86

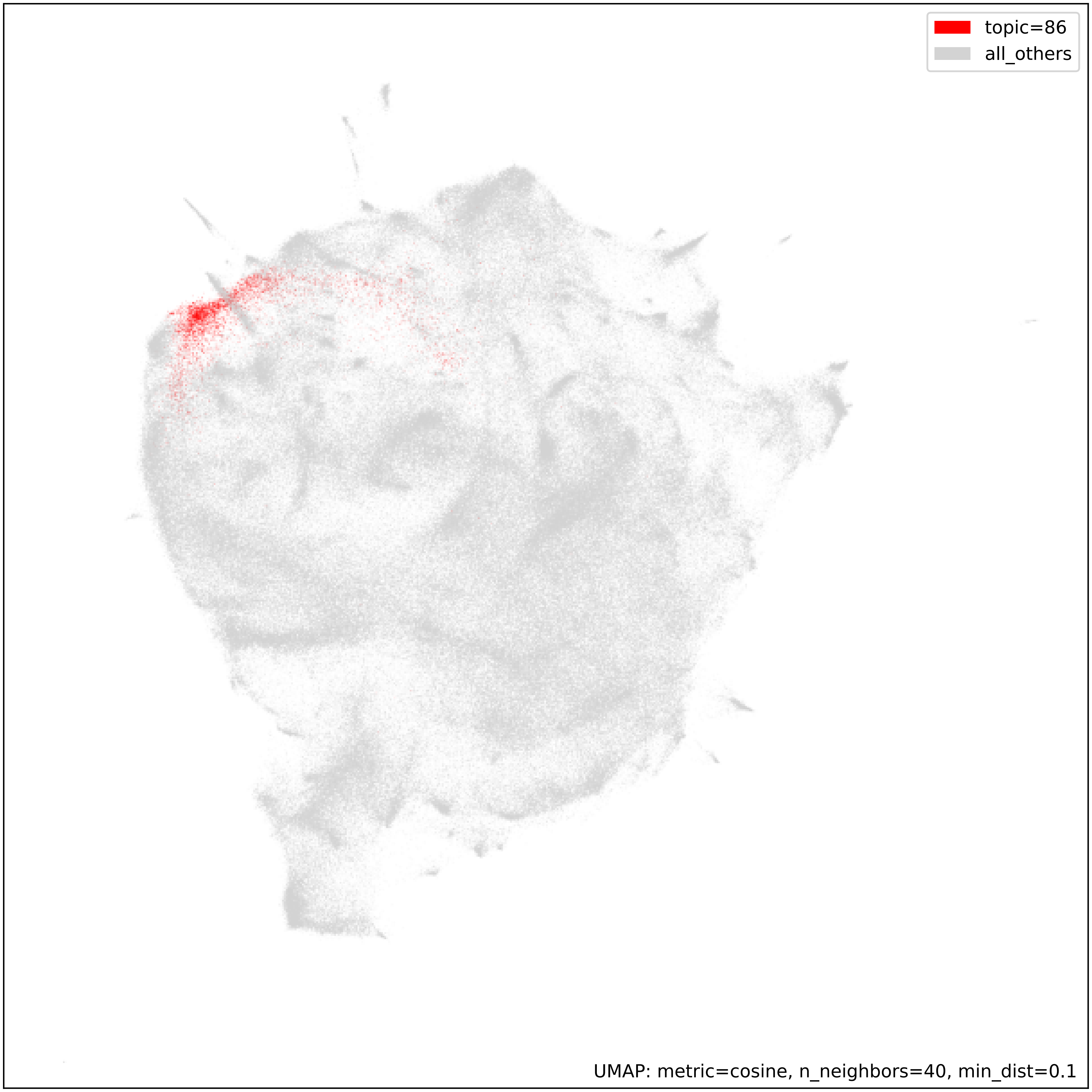
topic=86
all_others
UMAP: metric=cosine, n_neighbors=40, min_dist=0.1

Topic 87

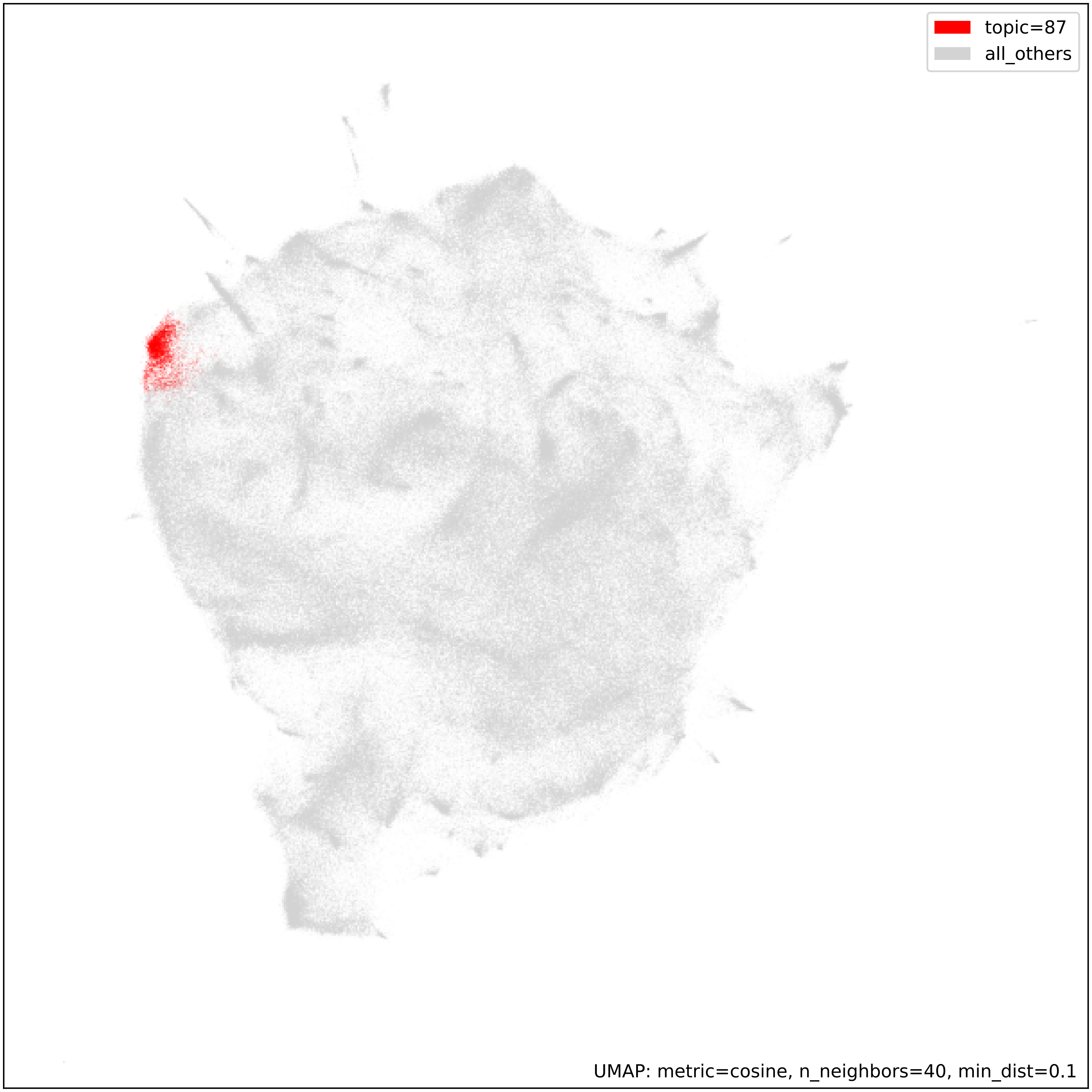
topic=87
all_others
UMAP: metric=cosine, n_neighbors=40, min_dist=0.1

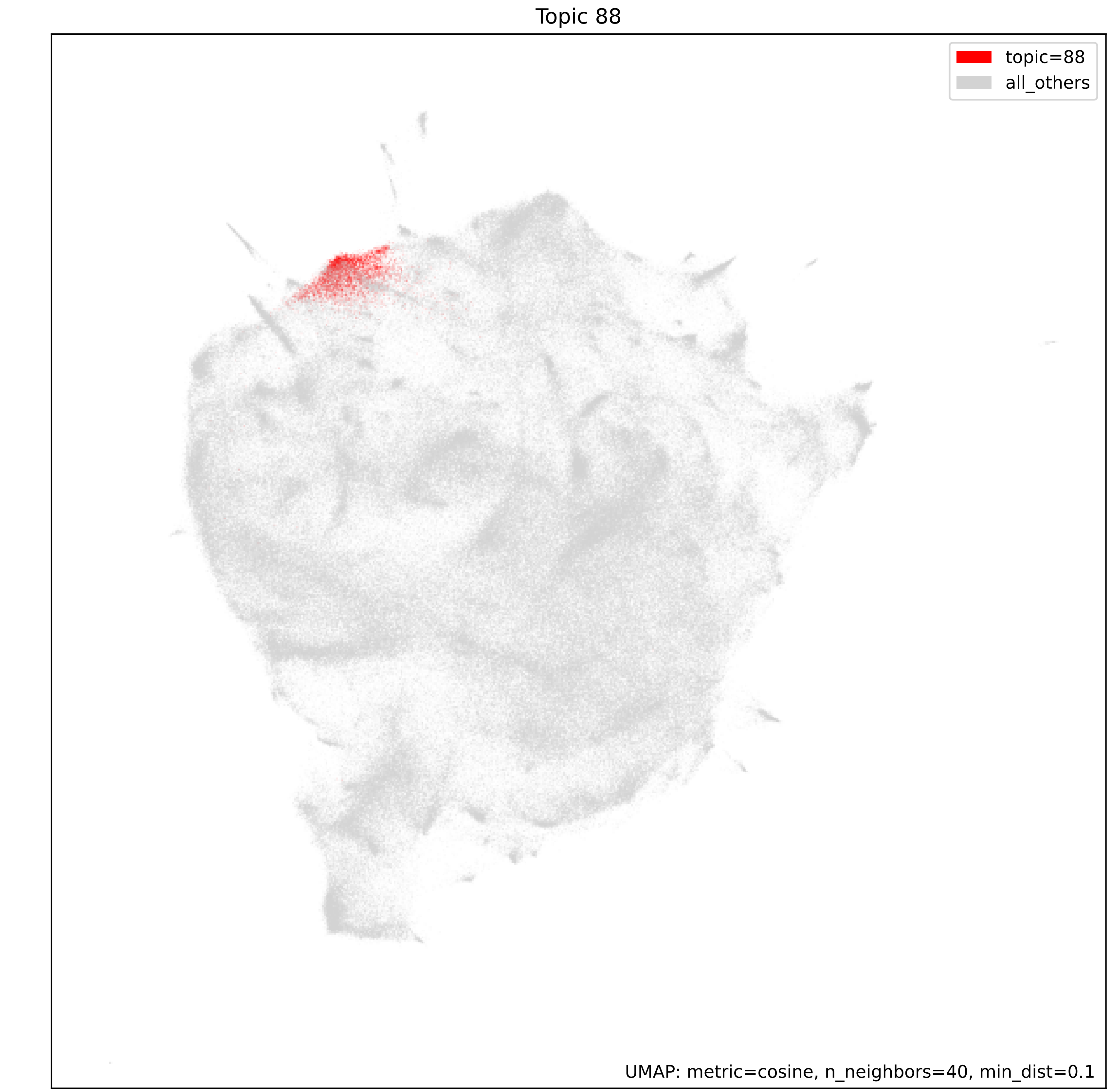
Topic 88
topic=88
all_others
UMAP: metric=cosine, n_neighbors=40, min_dist=0.1

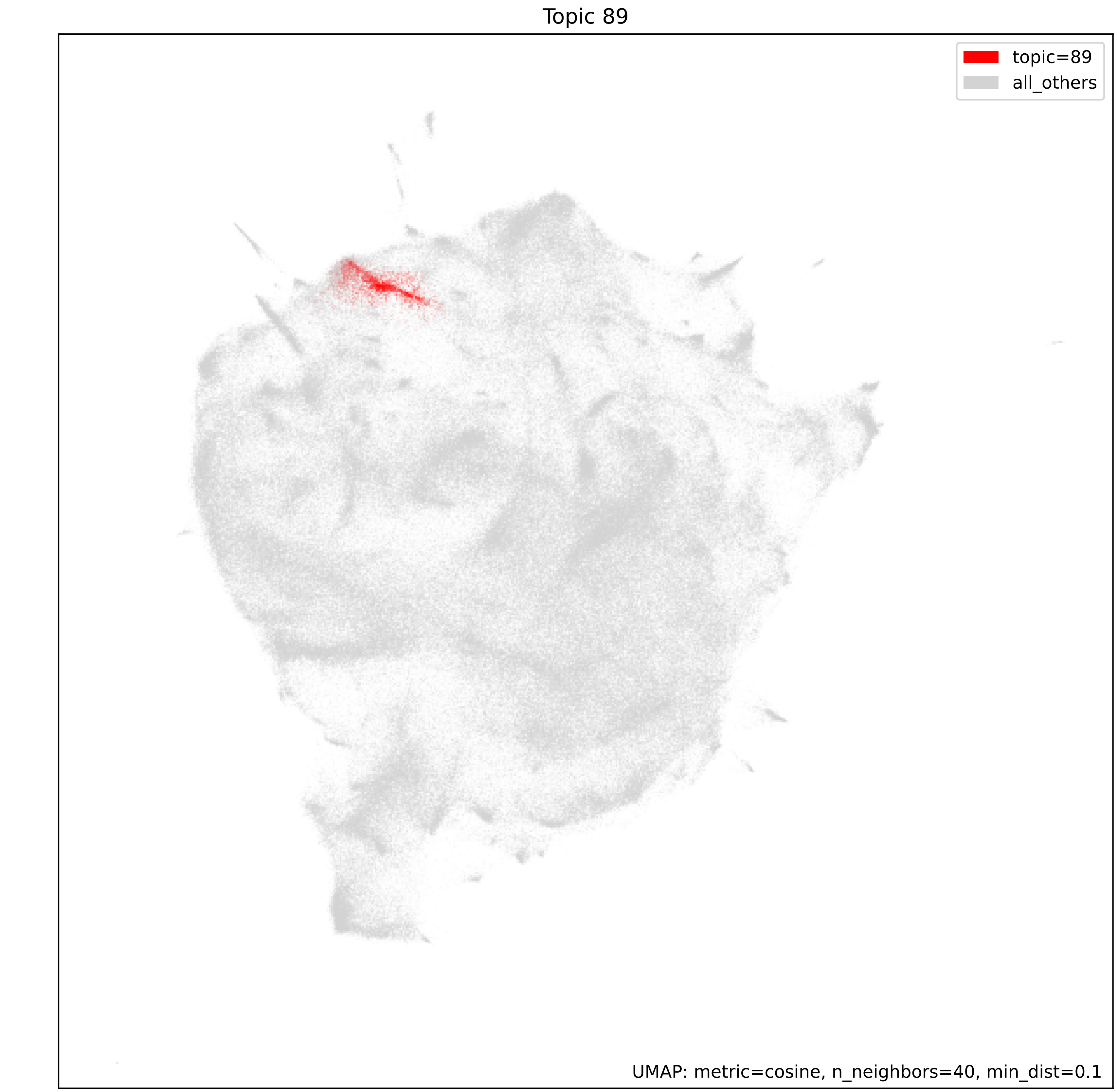
Topic 89
topic=89
all_others
UMAP: metric=cosine, n_neighbors=40, min_dist=0.1
