# Supplementary material for: Assessing the evolution of research topics in a biological field using plant science as an example
Source: PLoS Biol. 2024 May 23;22(5):e3002612. doi: 10.1371/journal.pbio.3002612 (PMC11115244; doi:10.1371/journal.pbio.3002612)
Supplement: S7 Data — There are 90 scatter plots, one for each topic, where the x axis is time, and the y axis is the document frequency (blue dots). The LOWESS fit is shown as orange points connected with a green line. The category a topic belongs to and its order in Fig 3 are labeled on the top left corner. The data used for plotting are in S6 Data. (PDF) [file pbio.3002612.s016.pdf]

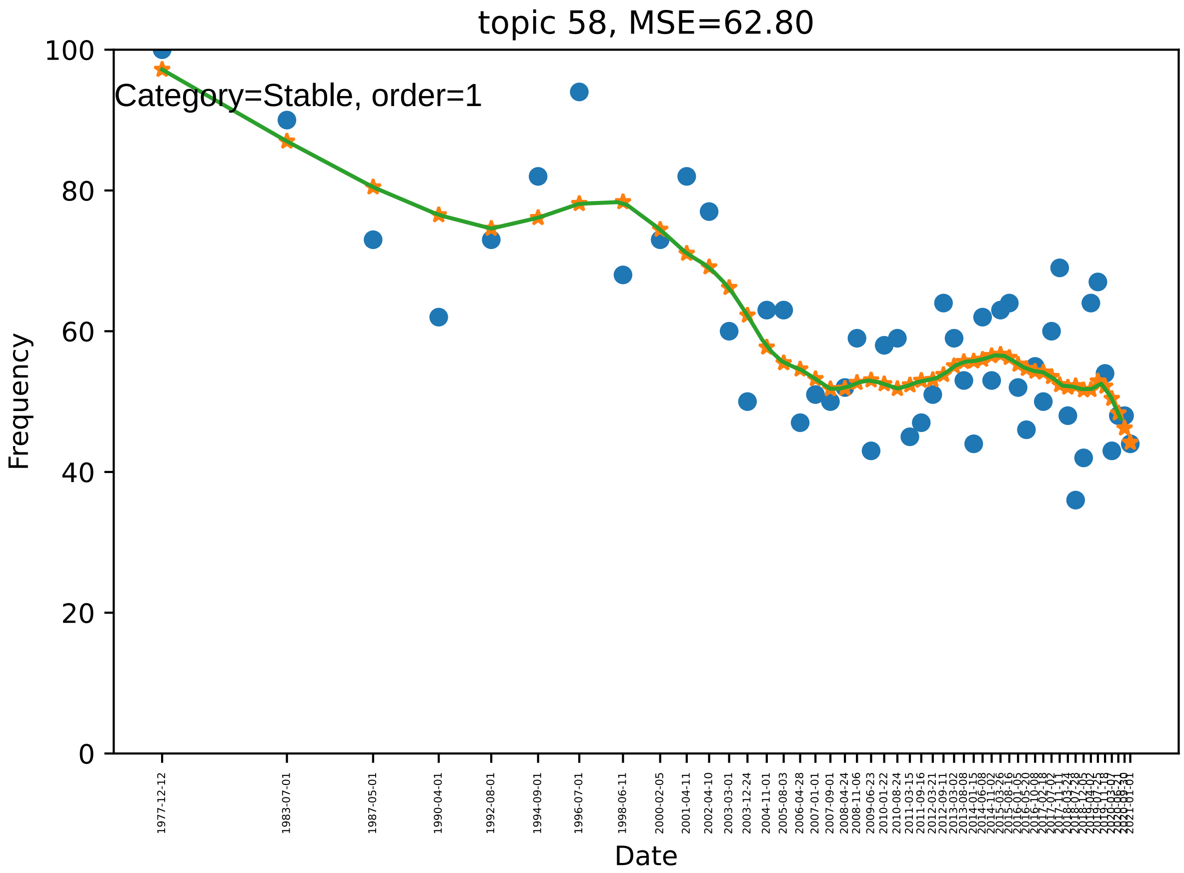

topic 58, MSE=62.80
Category=Stable, order=1
Frequency
Date
0
20
40
60
80
100
1977-12-12
1983-07-01
1987-05-01
1990-04-01
1992-08-01
1994-09-01
1996-07-01
1998-06-11
2000-02-05
2001-04-11
2002-04-10
2003-03-01
2003-12-24
2004-11-01
2005-08-03
2006-04-28
2007-01-01
2007-09-01
2008-04-24
2008-11-06
2009-06-23
2010-01-22
2010-08-24
2011-03-15
2011-09-16
2012-03-21
2012-09-11
2013-03-02
2013-08-08
2014-01-15
2014-06-08
2014-11-02
2015-03-26
2015-08-16
2016-01-05
2016-05-20
2016-10-08
2017-02-18
2017-07-02

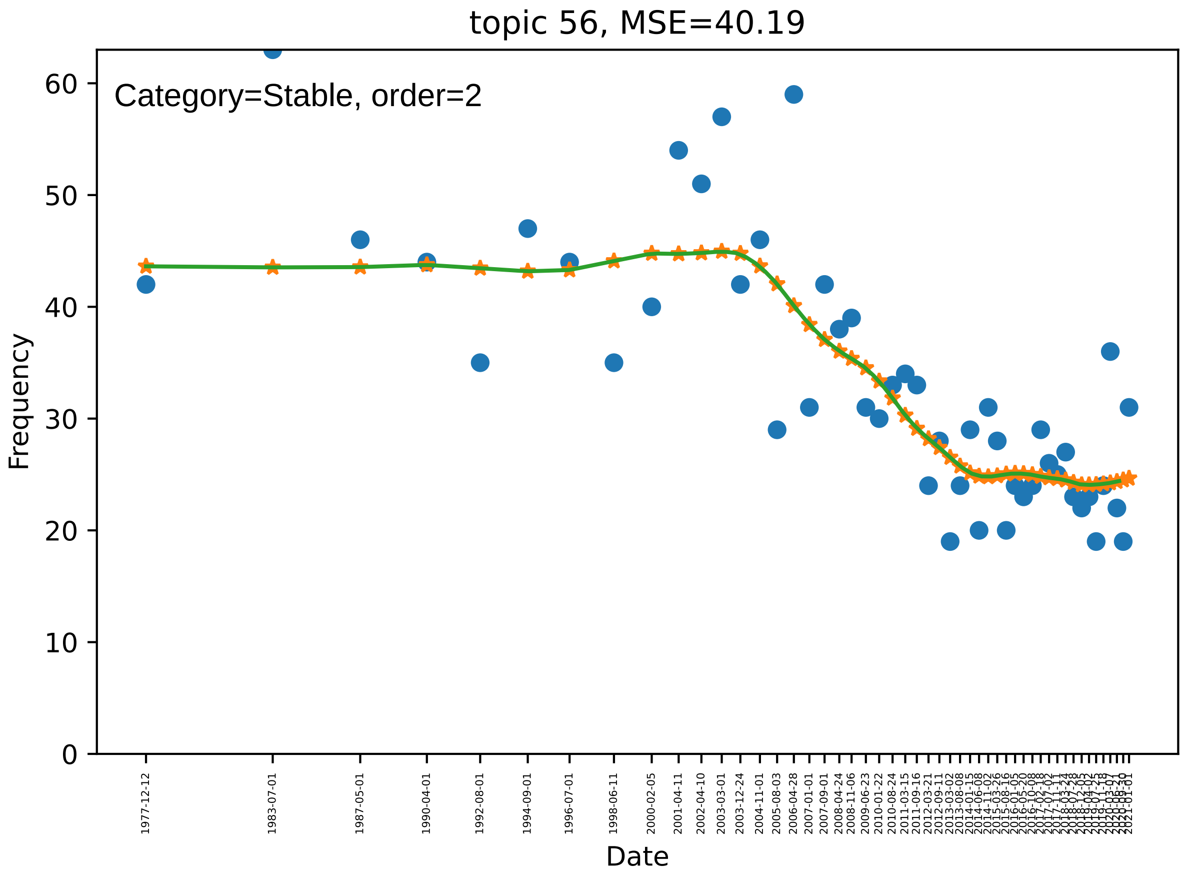
topic 56, MSE=40.19
Category=Stable, order=2
Frequency
0
10
20
30
40
50
60
Date
1977-12-12
1983-07-01
1987-05-01
1990-04-01
1992-08-01
1994-09-01
1996-07-01
1998-06-11
2000-02-05
2001-04-11
2002-04-10
2003-03-01
2003-12-24
2004-11-01
2005-08-03
2006-04-28
2007-01-01
2007-09-01
2008-04-24
2008-11-06
2009-06-23
2010-01-22
2010-08-24
2011-03-15
2011-09-16
2012-03-21
2012-09-11
2013-03-02
2013-08-08

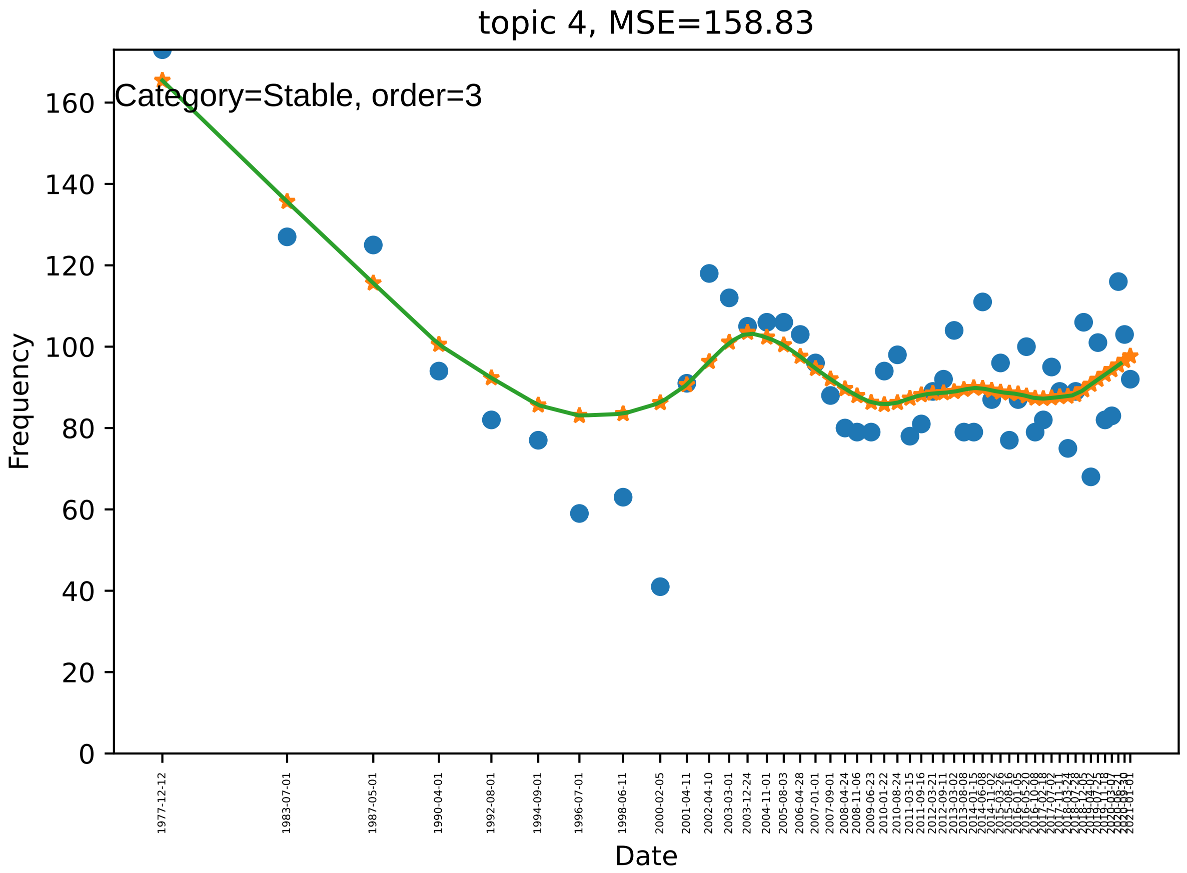

topic 4, MSE=158.83
Category=Stable, order=3
Frequency
Date
0
20
40
60
80
100
120
140
160
1977-12-12
1983-07-01
1987-05-01
1990-04-01
1992-08-01
1994-09-01
1996-07-01
1998-06-11
2000-02-05
2001-04-11
2002-04-10
2003-03-01
2003-12-24
2004-11-01
2005-08-03
2006-04-28
2007-01-01
2007-09-01
2008-04-24
2008-11-06
2009-06-23
2010-01-22
2010-08-24
2011-03-15
2011-09-16
2012-03-21
2012-09-11
2013-03-02
2013-08-08
2014-01-15
2014-06-08
2014-11-02
2015-03-26
2015-08-16
2016-01-05
2016-05-20
2016-10-08
2017-02-18
2017-07-02
2017-11-11
2021-01-01

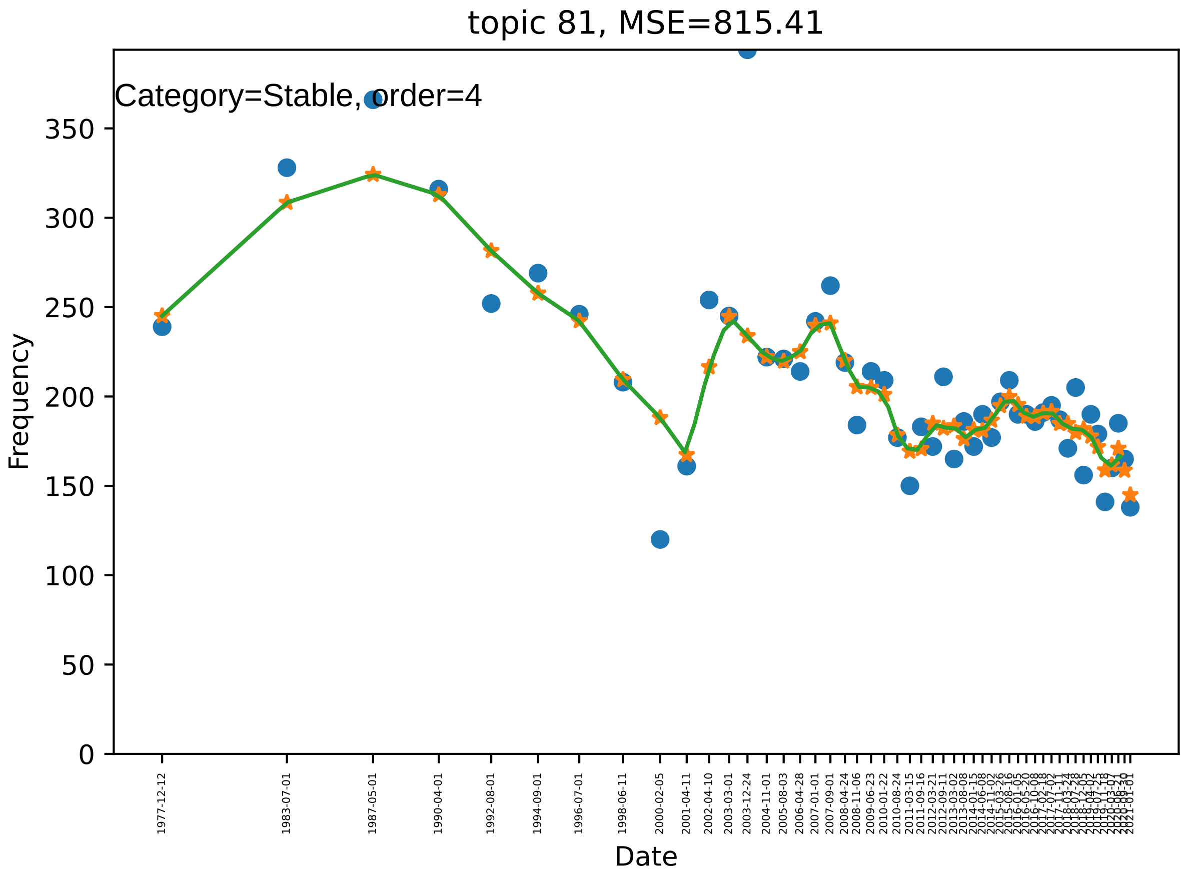
topic 81, MSE=815.41
Category=Stable, order=4
Frequency
Date
0
50
100
150
200
250
300
350
1977-12-12
1983-07-01
1987-05-01
1990-04-01
1992-08-01
1994-09-01
1996-07-01
1998-06-11
2000-02-05
2001-04-11
2002-04-10
2003-03-01
2003-12-24
2004-11-01
2005-08-03
2006-04-28
2007-01-01
2007-09-01
2008-04-24
2008-11-06
2009-06-23
2010-01-22
2010-08-24
2011-03-15
2011-09-16
2012-03-21
2012-09-11
2013-03-02
2013-08-08
2014-01-15
2014-06-08
2014-11-02
2015-03-26
2015-08-16
2016-01-05
2016-05-20
2016-10-08
2017-02-18
2017-07-02
2017-11-11

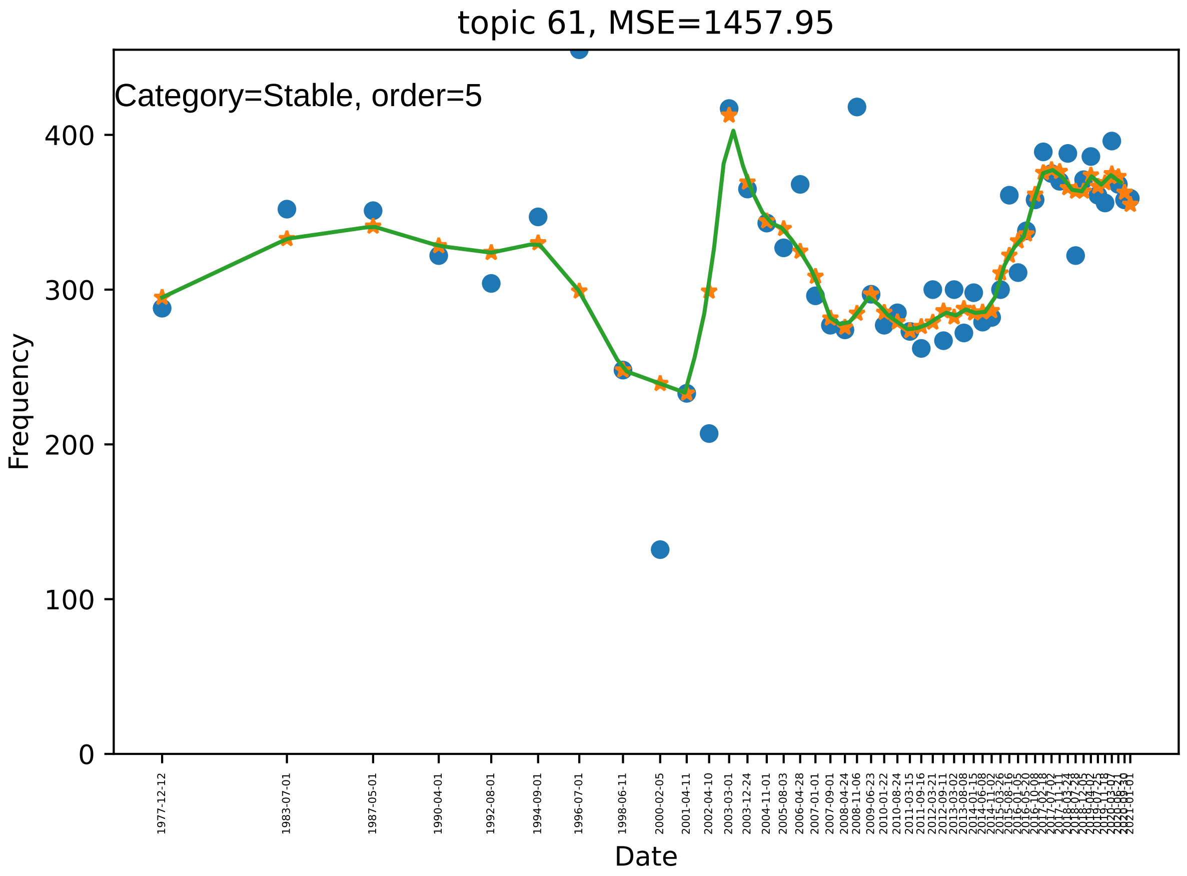
topic 61, MSE=1457.95
Category=Stable, order=5
Frequency
0
100
200
300
400
Date
1977-12-12
1983-07-01
1987-05-01
1990-04-01
1992-08-01
1994-09-01
1996-07-01
1998-06-11
2000-02-05
2001-04-11
2002-04-10
2003-03-01
2003-12-24
2004-11-01
2005-08-03
2006-04-28
2007-01-01
2007-09-01
2008-04-24
2008-11-06
2009-06-23
2010-01-22
2010-08-24
2011-03-15
2011-09-16
2012-03-21
2012-09-11
2013-03-02
2013-08-08
2014-01-15
2014-06-08
2014-11-02
2015-03-26
2015-08-16
2016-01-05
2016-05-20
2016-10-08

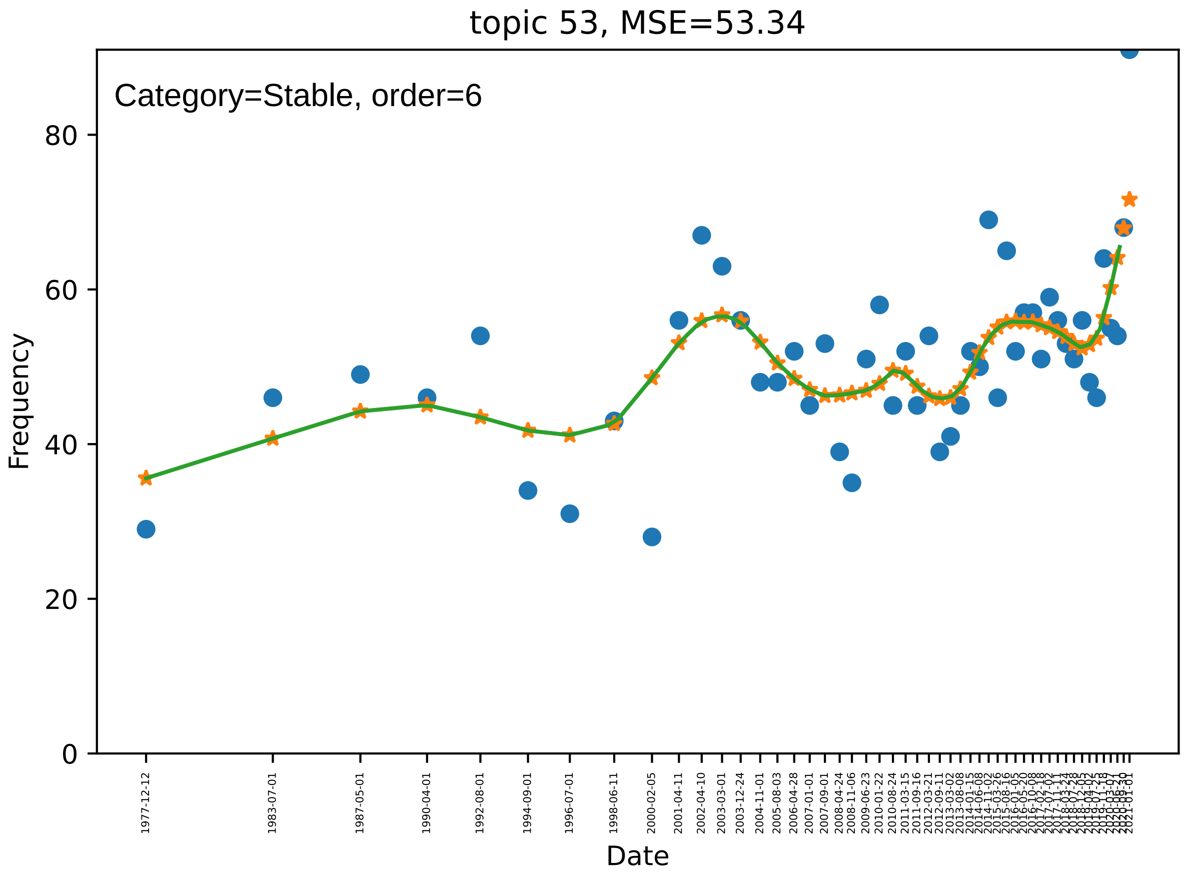
topic 53, MSE=53.34
Category=Stable, order=6
Frequency
Date
0
20
40
60
80
1977-12-12
1983-07-01
1987-05-01
1990-04-01
1992-08-01
1994-09-01
1996-07-01
1998-06-11
2000-02-05
2001-04-11
2002-04-10
2003-03-01
2003-12-24
2004-11-01
2005-08-03
2006-04-28
2007-01-01
2007-09-01
2008-04-24
2008-11-06
2009-06-23
2010-01-22
2010-08-24
2011-03-15
2011-09-16
2012-03-21
2012-09-11
2013-03-02
2013-08-08
2014-01-15
2014-06-08
2014-11-02

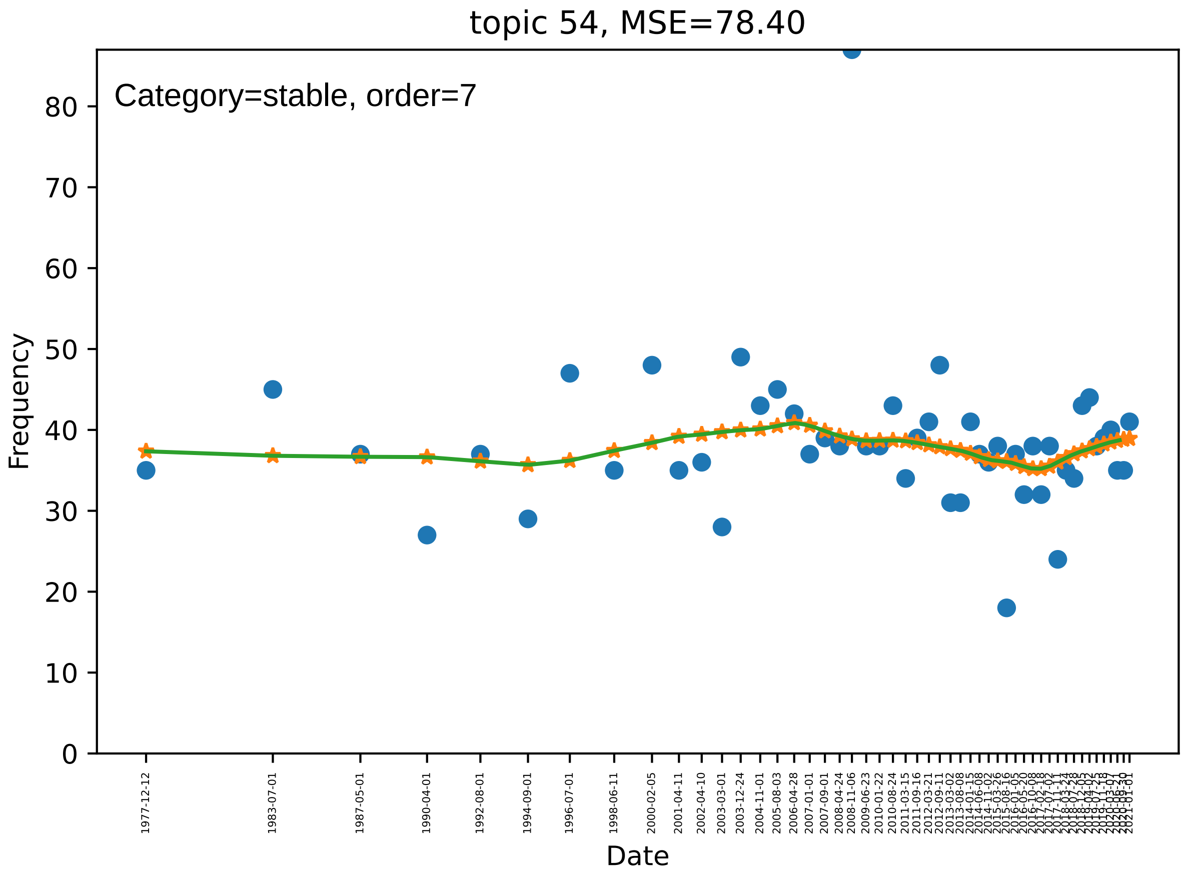
topic 54, MSE=78.40
Category=stable, order=7
Frequency
Date
0
10
20
30
40
50
60
70
80

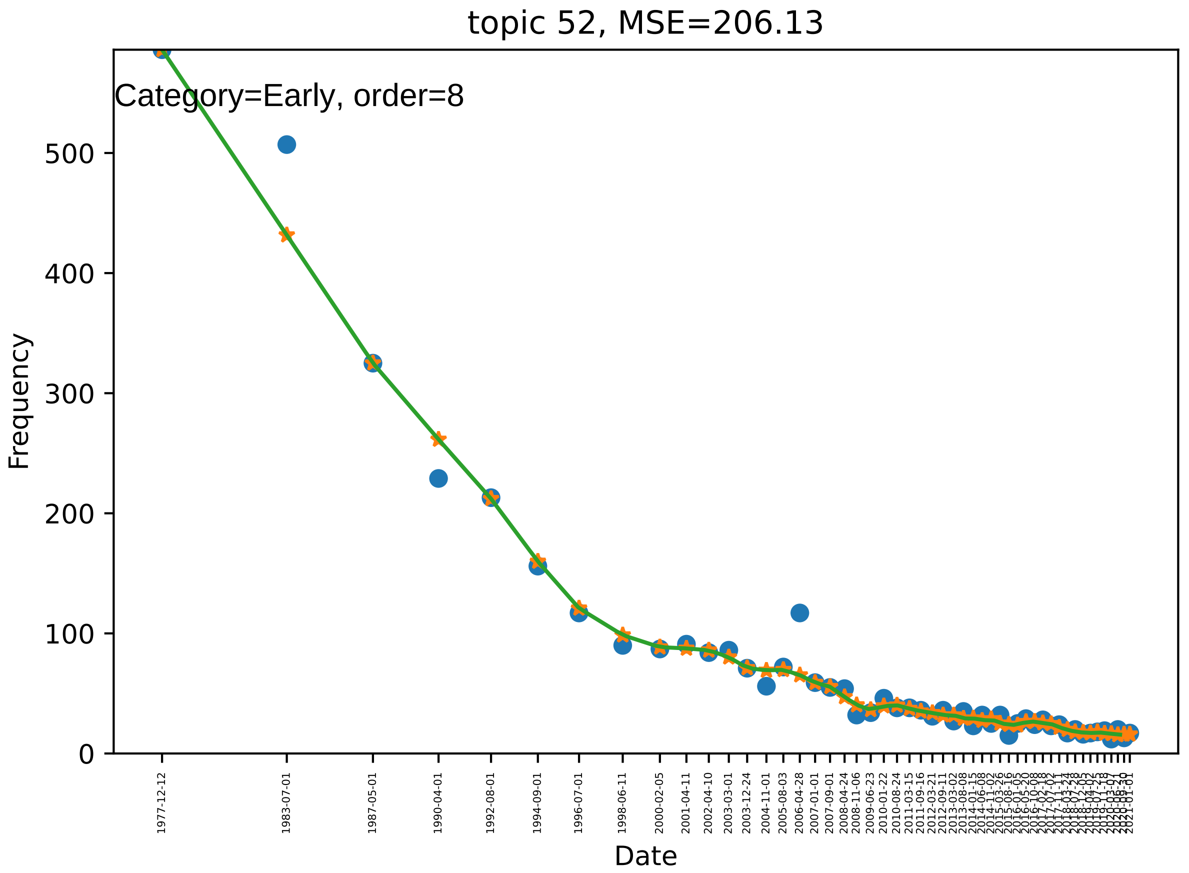
topic 52, MSE=206.13
Category=Early, order=8
Frequency
Date
0
100
200
300
400
500
1977-12-12
1983-07-01
1987-05-01
1990-04-01
1992-08-01
1994-09-01
1996-07-01
1998-06-11
2000-02-05
2001-04-11
2002-04-10
2003-03-01
2003-12-24
2004-11-01
2005-08-03
2006-04-28
2007-01-01
2007-09-01
2008-04-24
2008-11-06
2009-06-23
2010-01-22
2010-08-24
2011-03-15
2011-09-16
2012-03-21
2012-09-11
2013-03-02

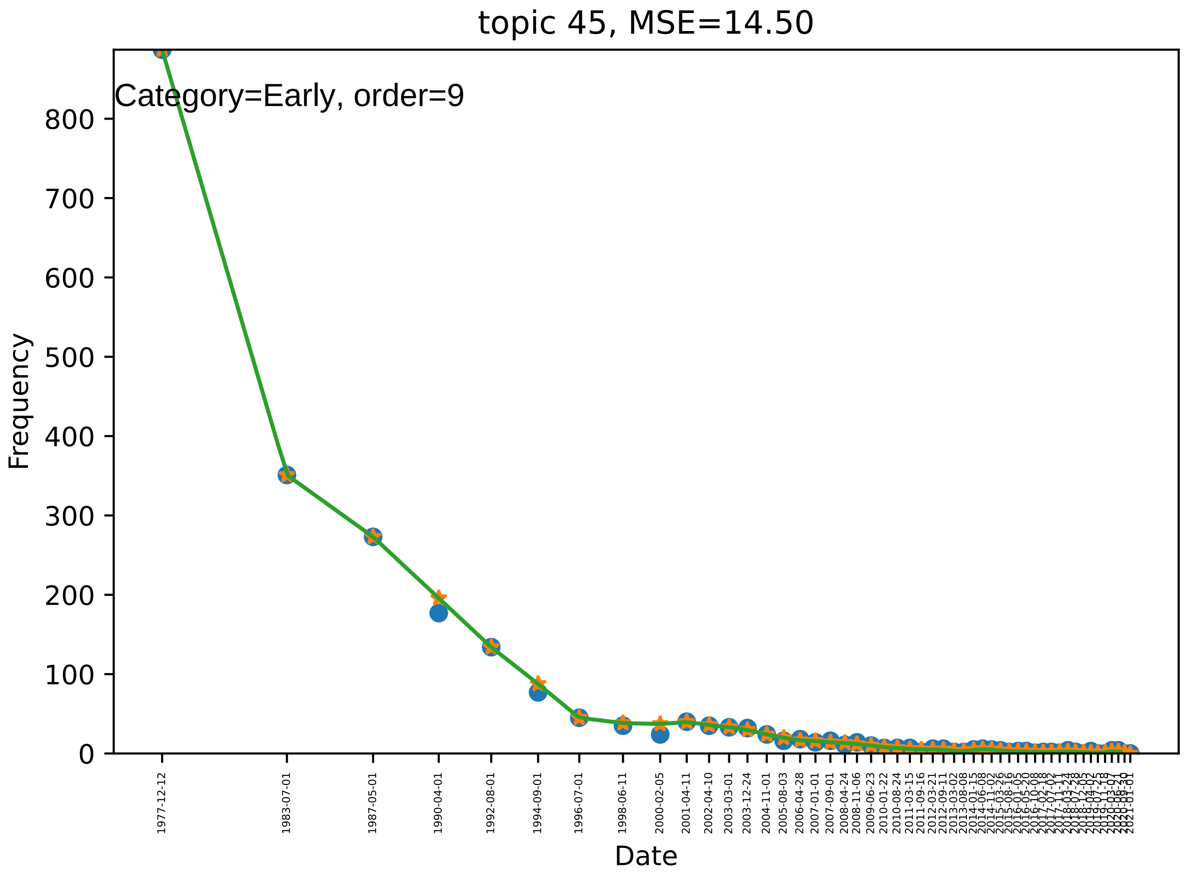

topic 45, MSE=14.50
Category=Early, order=9
Frequency
Date
0
100
200
300
400
500
600
700
800
1977-12-12
1983-07-01
1987-05-01
1990-04-01
1992-08-01
1994-09-01
1996-07-01
1998-06-11
2000-02-05
2001-04-11
2002-04-10
2003-03-01
2003-12-24
2004-11-01
2005-08-03
2006-04-28
2007-01-01
2007-09-01
2008-04-24
2008-11-06
2009-06-23
2010-01-22
2010-08-24
2011-03-15
2011-09-16
2012-03-21
2012-09-11
2013-03-02
2013-08-08
2014-01-15
2014-06-08
2014-11-02
2015-03-26
2015-08-16
2016-01-05
2016-05-20
2016-10-08
2017-02-18
2017-07-02
2017-11-11
2021-01-01

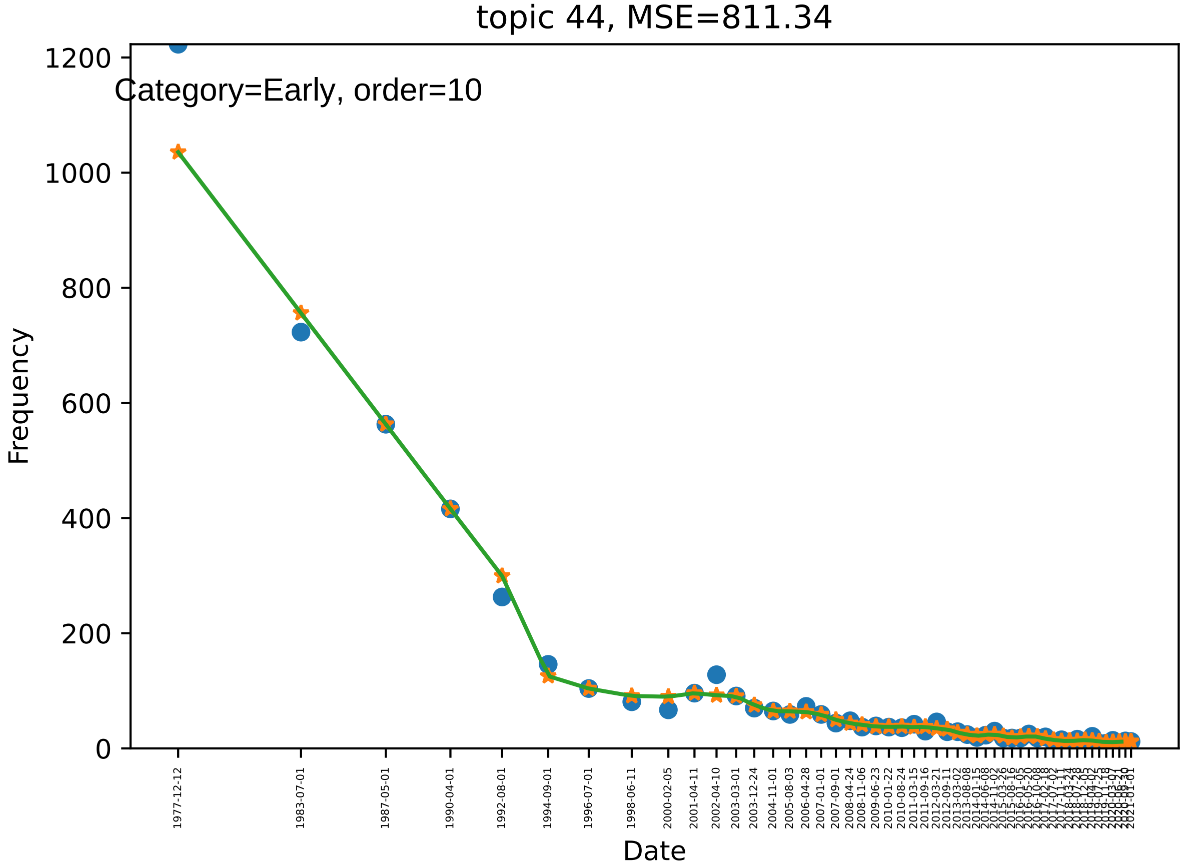
topic 44, MSE=811.34
Category=Early, order=10
Frequency
0
200
400
600
800
1000
1200
Date
1977-12-12
1983-07-01
1987-05-01
1990-04-01
1992-08-01
1994-09-01
1996-07-01
1998-06-11
2000-02-05
2001-04-11
2002-04-10
2003-03-01
2003-12-24
2004-11-01
2005-08-03
2006-04-28
2007-01-01
2007-09-01
2008-04-24
2008-11-06
2009-06-23
2010-01-22
2010-08-24
2011-03-15
2011-09-16
2012-03-21
2012-09-11
2013-03-02
2013-08-08
2014-01-15
2014-06-08
2014-11-02
2015-03-26
2015-08-16
2016-01-05
2016-05-20
2016-10-08
2017-02-18
2017-07-02
2017-11-11
2018-03-24
2018-07-28

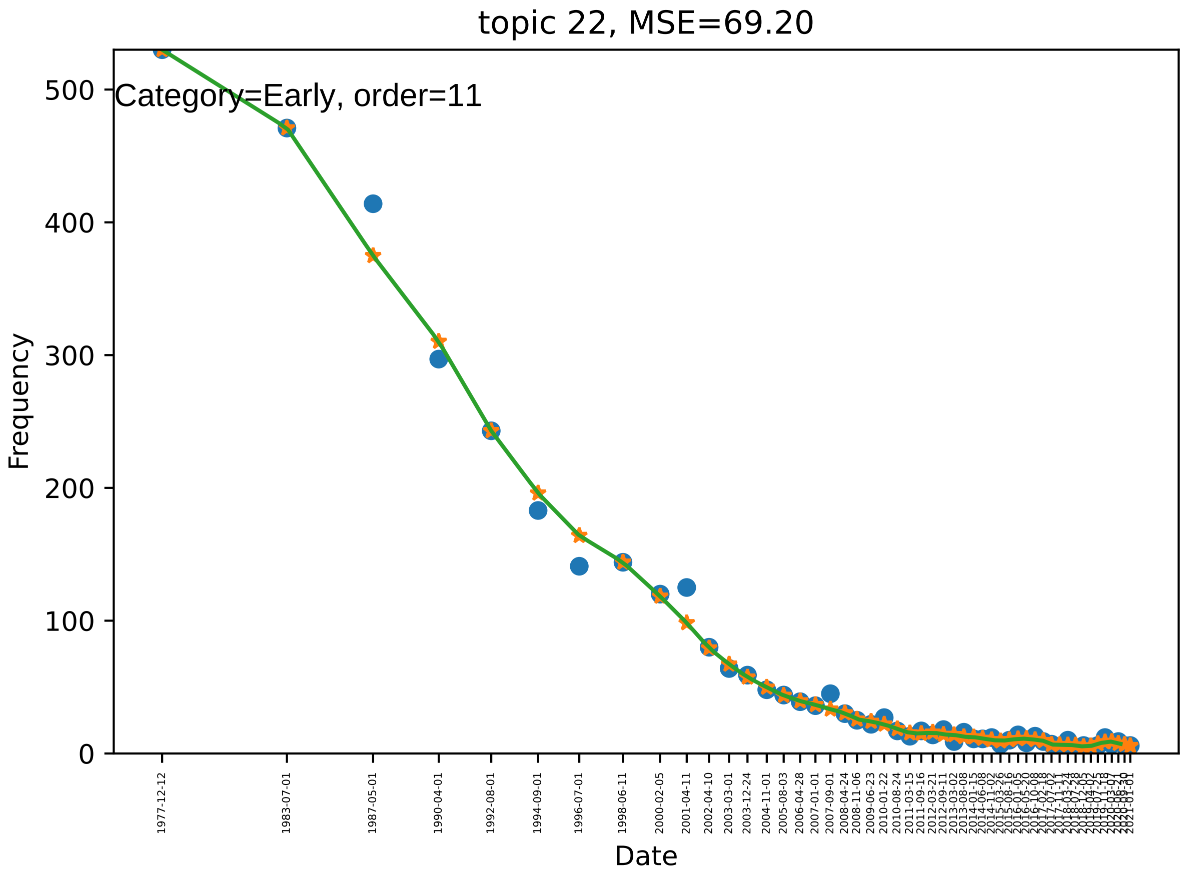
topic 22, MSE=69.20
Category=Early, order=11
Frequency
0
100
200
300
400
500
Date
1977-12-12
1983-07-01
1987-05-01
1990-04-01
1992-08-01
1994-09-01
1996-07-01
1998-06-11
2000-02-05
2001-04-11
2002-04-10
2003-03-01
2003-12-24
2004-11-01
2005-08-03
2006-04-28
2007-01-01
2007-09-01
2008-04-24
2008-11-06
2009-06-23
2010-01-22
2010-08-24
2011-03-15
2011-09-16
2012-03-21
2012-09-11
2013-03-02
2013-08-08
2014-01-15
2014-06-08
2014-11-02
2015-03-26
2015-08-16
2016-01-05
2016-05-20
2016-10-08
2017-02-18

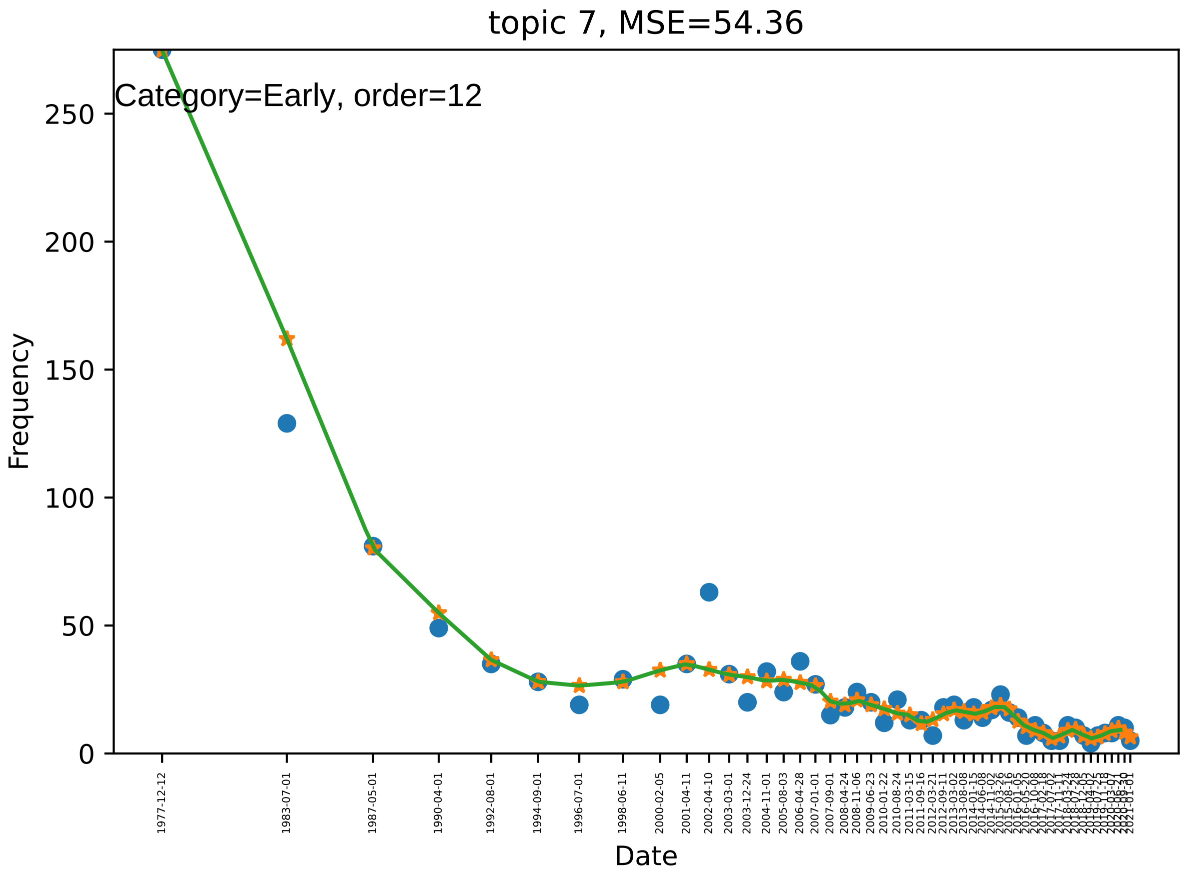

topic 7, MSE=54.36
Category=Early, order=12
Frequency
Date

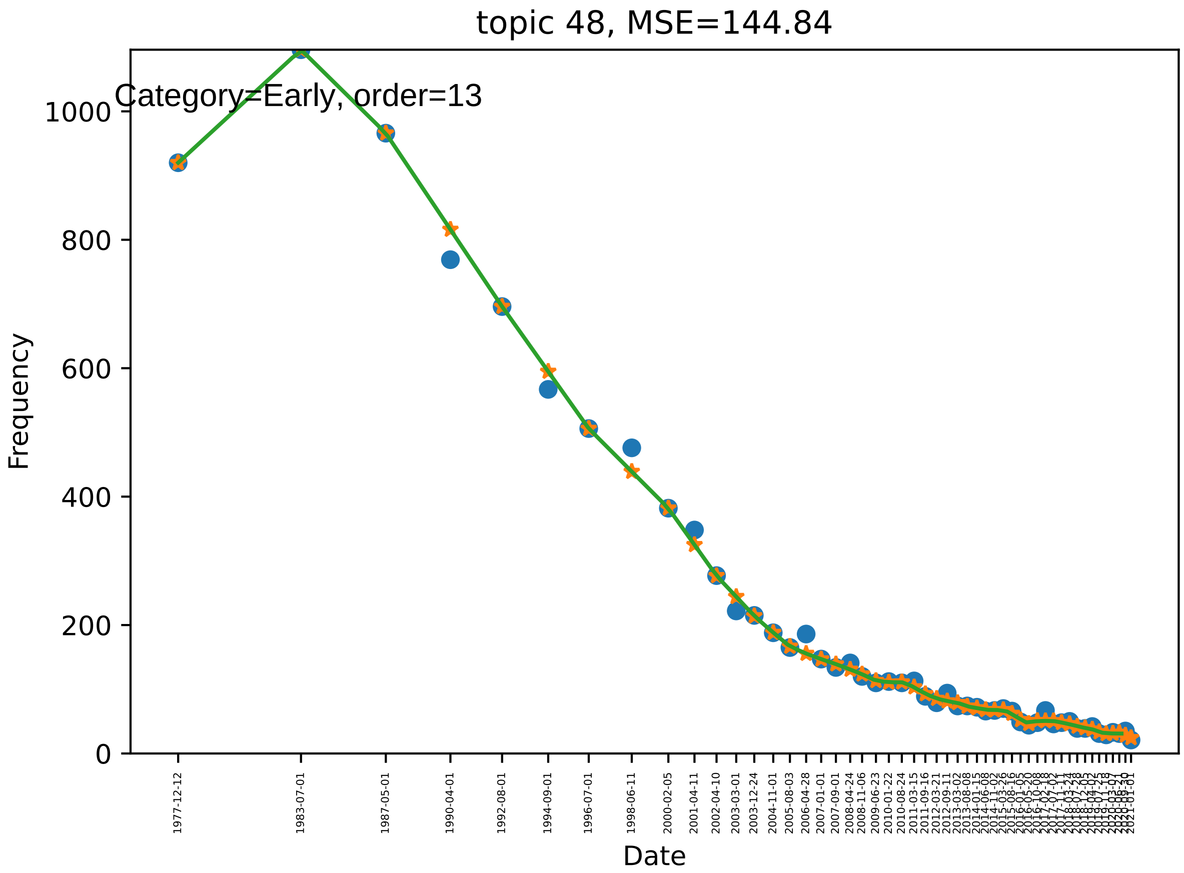
topic 48, MSE=144.84
Category=Early, order=13
Frequency
0
200
400
600
800
1000
Date
1977-12-12
1983-07-01
1987-05-01
1990-04-01
1992-08-01
1994-09-01
1996-07-01
1998-06-11
2000-02-05
2001-04-11
2002-04-10
2003-03-01
2003-12-24
2004-11-01
2005-08-03
2006-04-28
2007-01-01
2007-09-01
2008-04-24
2008-11-06
2009-06-23
2010-01-22
2010-08-24
2011-03-15
2011-09-16
2012-03-21
2012-09-11
2013-03-02
2013-08-08
2014-01-15
2014-06-08
2014-11-02
2015-03-26
2015-08-16
2016-01-05
2016-05-20
2016-10-08
2017-02-18
2017-07-02
2017-11-11
2018-03-24
2018-07-28
2018-12-05
2021-01-01

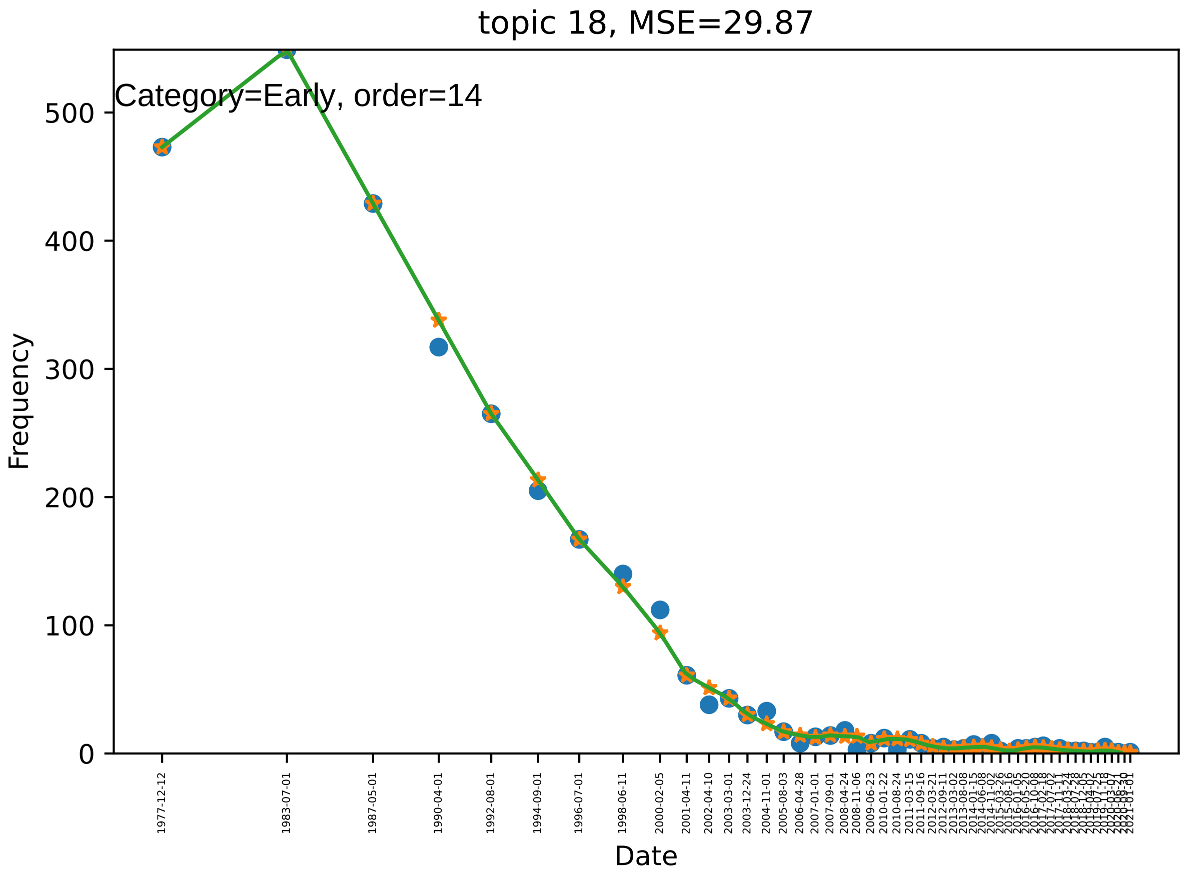

topic 18, MSE=29.87
Category=Early, order=14
Frequency
Date
0
100
200
300
400
500
1977-12-12
1983-07-01
1987-05-01
1990-04-01
1992-08-01
1994-09-01
1996-07-01
1998-06-11
2000-02-05
2001-04-11
2002-04-10
2003-03-01
2003-12-24
2004-11-01
2005-08-03
2006-04-28
2007-01-01
2007-09-01
2008-04-24
2008-11-06
2009-06-23
2010-01-22
2010-08-24
2011-03-15
2011-09-16
2012-03-21
2012-09-11
2013-03-02
2013-08-08
2014-01-15
2014-06-08
2014-11-02
2015-03-26
2015-08-16
2016-01-05
2016-05-20
2016-10-08
2017-02-18
2017-07-02
2017-11-11

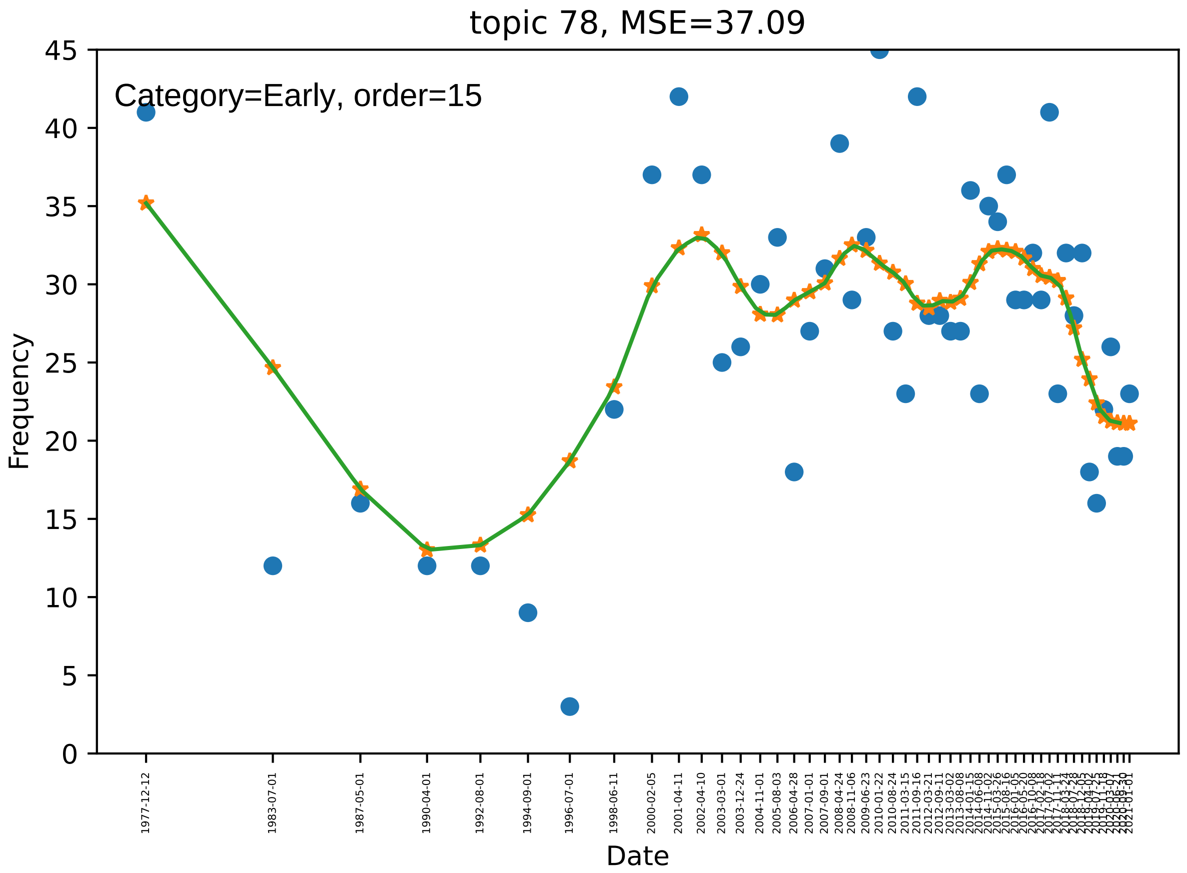
topic 78, MSE=37.09
Category=Early, order=15
Frequency
0
5
10
15
20
25
30
35
40
45
Date
1977-12-12
1983-07-01
1987-05-01
1990-04-01
1992-08-01
1994-09-01
1996-07-01
1998-06-11
2000-02-05
2001-04-11
2002-04-10
2003-03-01
2003-12-24
2004-11-01
2005-08-03
2006-04-28
2007-01-01
2007-09-01
2008-04-24
2008-11-06
2009-06-23
2010-01-22
2010-08-24
2011-03-15
2011-09-16
2012-03-21
2012-09-11
2013-03-02
2013-08-08
2014-01-15
2014-06-08
2014-11-02
2015-03-26

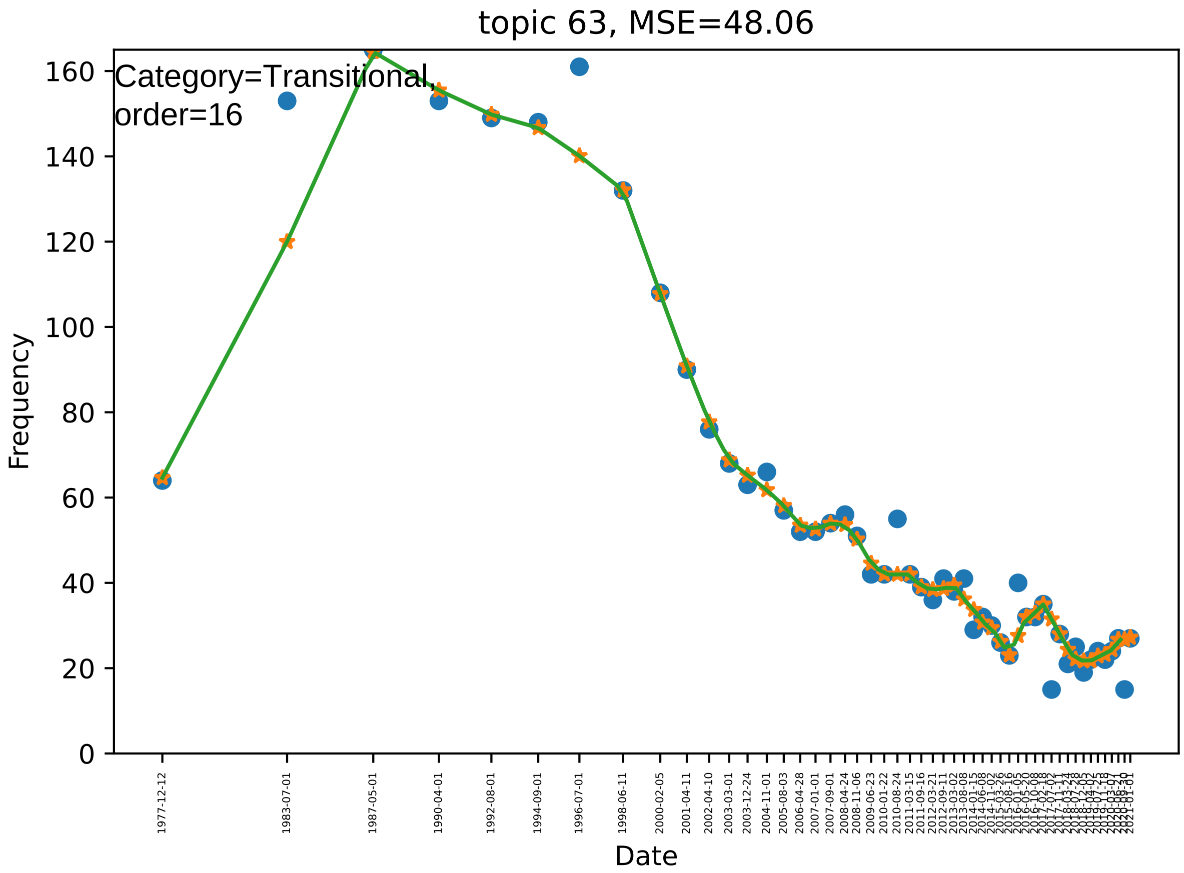

topic 63, MSE=48.06
Category=Transitional,
order=16
Frequency
Date
0
20
40
60
80
100
120
140
160

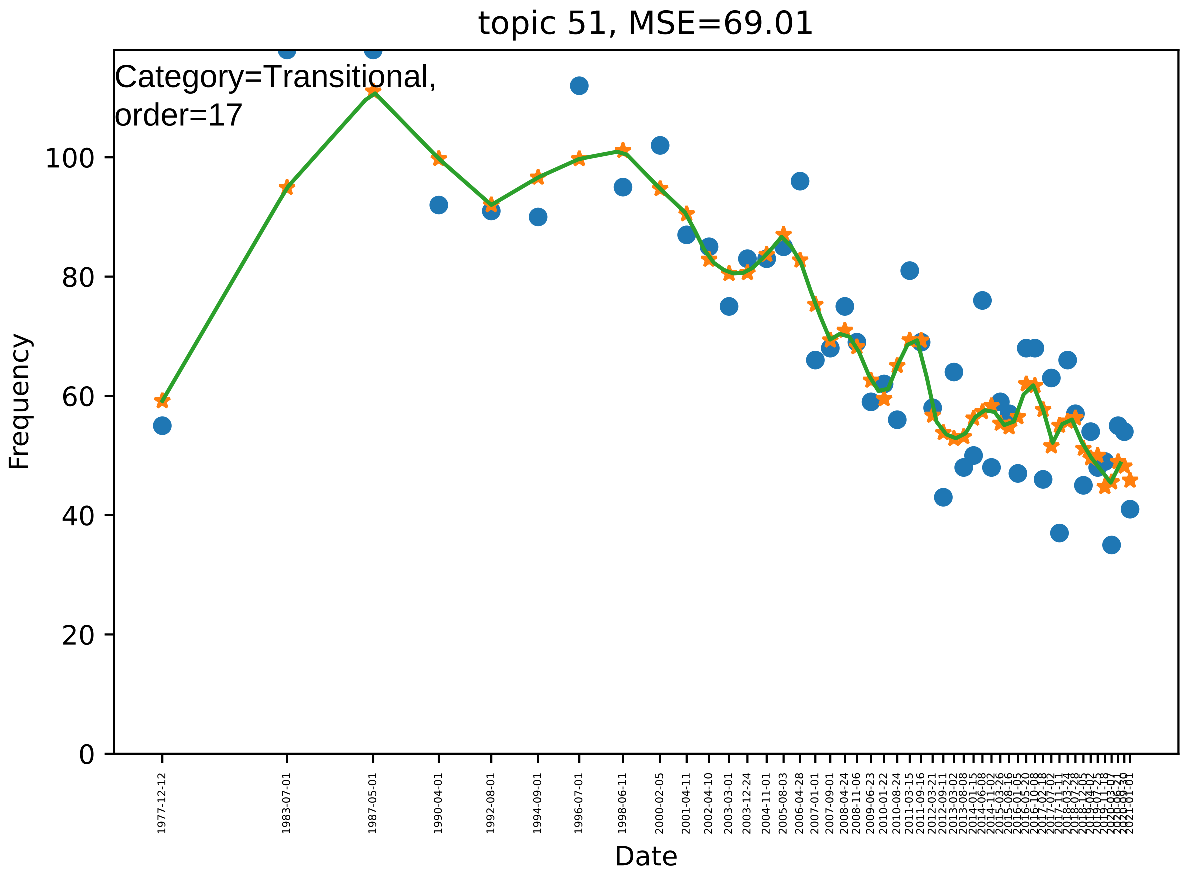
topic 51, MSE=69.01
Category=Transitional,
order=17
Frequency
Date
0
20
40
60
80
100
1977-12-12
1983-07-01
1987-05-01
1990-04-01
1992-08-01
1994-09-01
1996-07-01
1998-06-11
2000-02-05
2001-04-11
2002-04-10
2003-03-01
2003-12-24
2004-11-01
2005-08-03
2006-04-28
2007-01-01
2007-09-01
2008-04-24
2008-11-06
2009-06-23
2010-01-22
2010-08-24
2011-03-15
2011-09-16
2012-03-21
2012-09-11
2013-03-02
2013-08-08
2014-01-15
2014-06-08
2014-11-02
2015-03-26
2015-08-16
2016-01-05
2016-05-20
2016-10-08
2017-02-18
2017-07-02

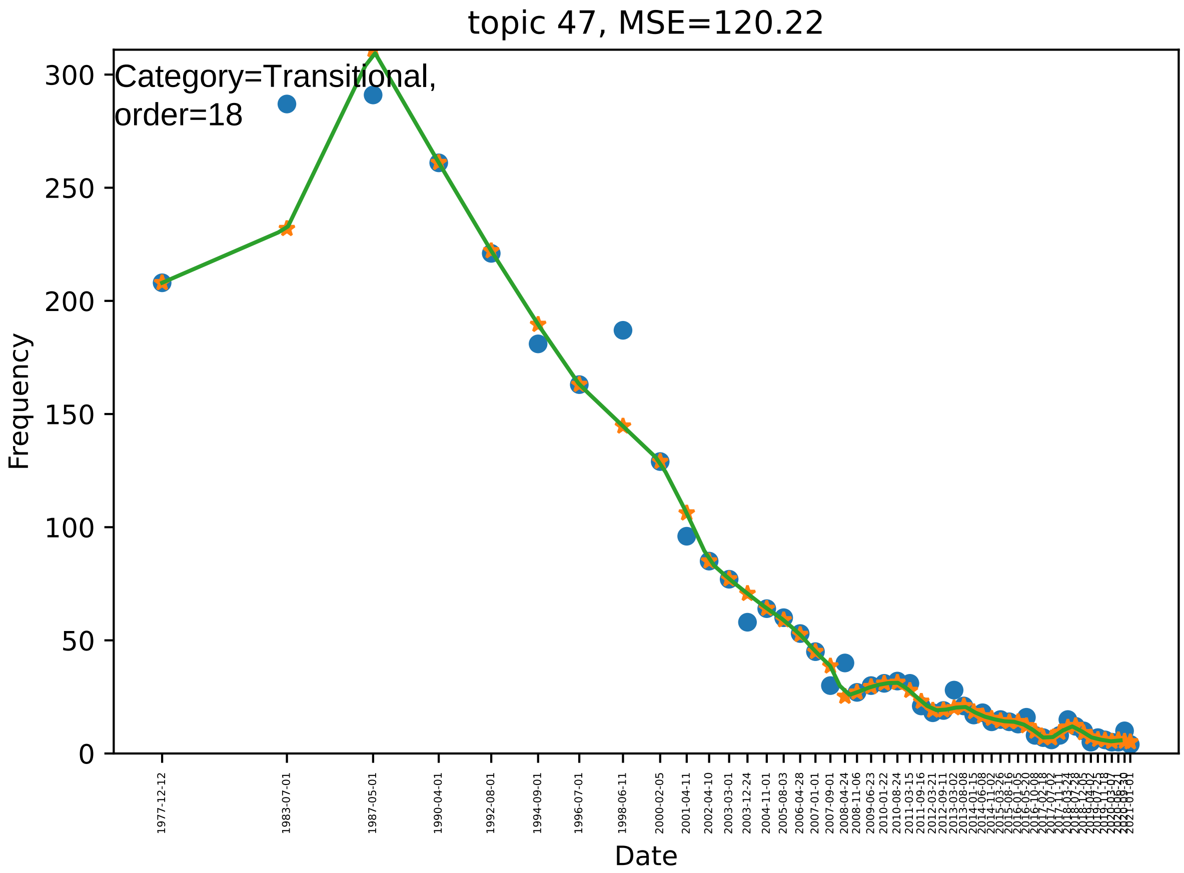

topic 47, MSE=120.22
Category=Transitional,
order=18
Frequency
Date
0
50
100
150
200
250
300
1977-12-12
1983-07-01
1987-05-01
1990-04-01
1992-08-01
1994-09-01
1996-07-01
1998-06-11
2000-02-05
2001-04-11
2002-04-10
2003-03-01
2003-12-24
2004-11-01
2005-08-03
2006-04-28
2007-01-01
2007-09-01
2008-04-24
2008-11-06
2009-06-23
2010-01-22
2010-08-24
2011-03-15
2011-09-16
2012-03-21
2012-09-11
2013-03-02

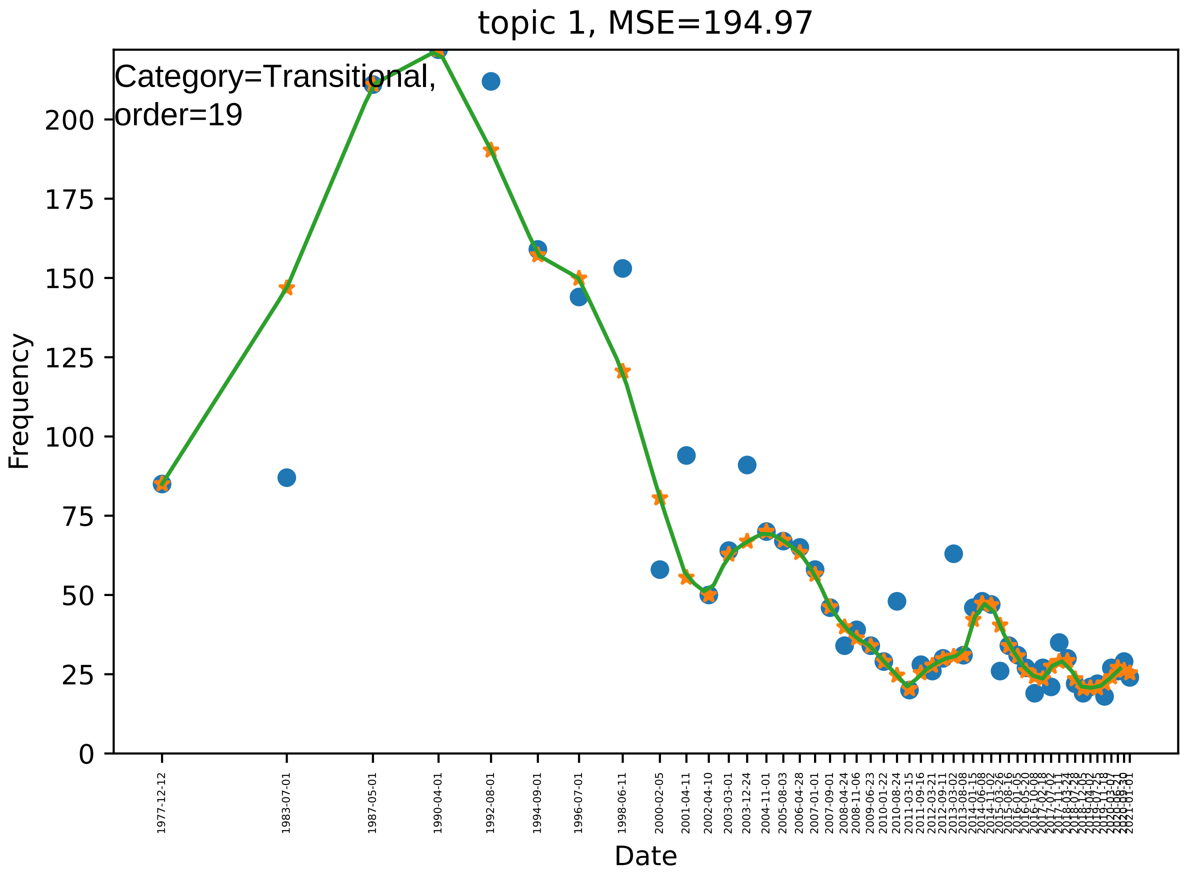
topic 1, MSE=194.97
Category=Transitional,
order=19
Frequency
0
25
50
75
100
125
150
175
200
Date
1977-12-12
1983-07-01
1987-05-01
1990-04-01
1992-08-01
1994-09-01
1996-07-01
1998-06-11
2000-02-05
2001-04-11
2002-04-10
2003-03-01
2003-12-24
2004-11-01
2005-08-03
2006-04-28
2007-01-01
2007-09-01
2008-04-24
2008-11-06
2009-06-23
2010-01-22
2010-08-24
2011-03-15
2011-09-16
2012-03-21
2012-09-11
2013-03-02
2013-08-08
2014-01-15

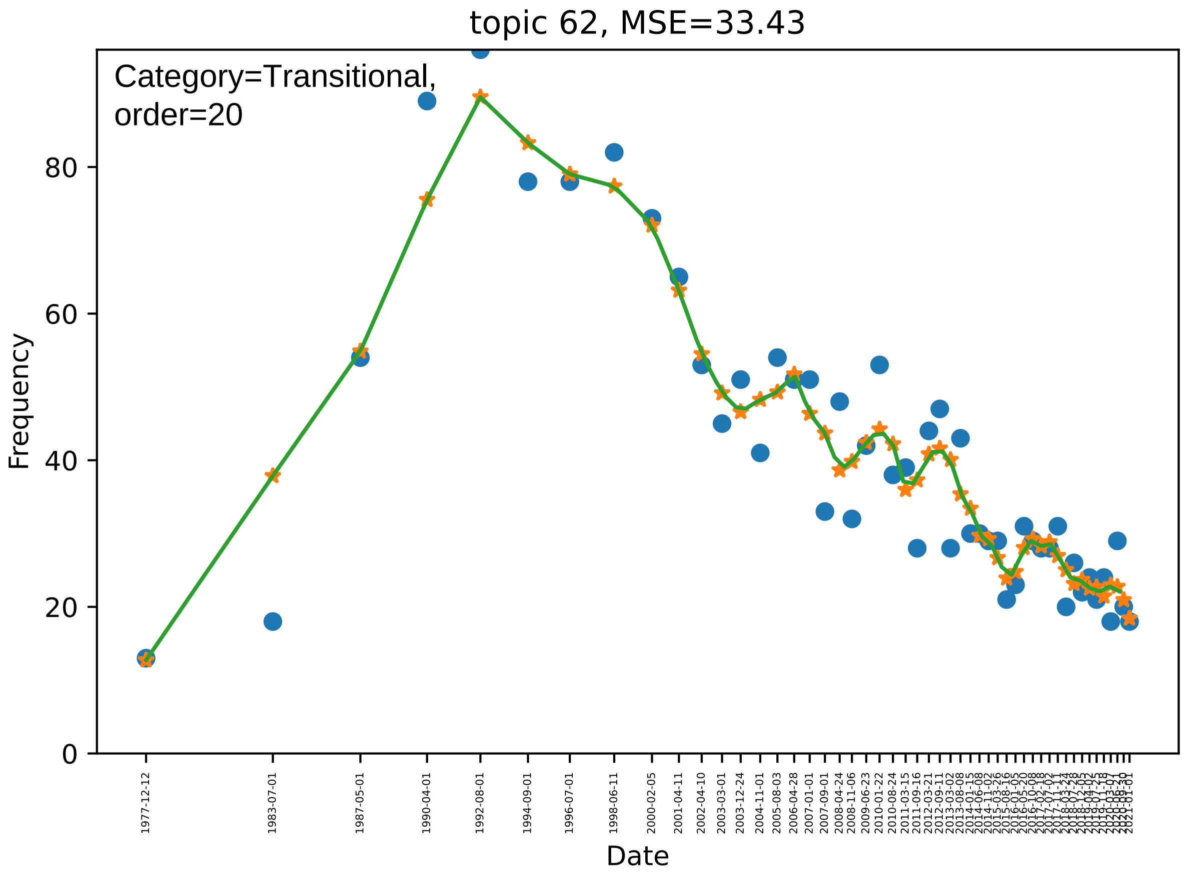

topic 62, MSE=33.43
Category=Transitional,
order=20
Frequency
0
20
40
60
80
Date
1977-12-12
1983-07-01
1987-05-01
1990-04-01
1992-08-01
1994-09-01
1996-07-01
1998-06-11
2000-02-05
2001-04-11
2002-04-10
2003-03-01
2003-12-24
2004-11-01
2005-08-03
2006-04-28
2007-01-01
2007-09-01
2008-04-24
2008-11-06
2009-06-23
2010-01-22
2010-08-24
2011-03-15
2011-09-16
2012-03-21
2012-09-11
2013-03-02
2013-08-08
2014-01-15
2014-06-08
2014-11-02
2015-03-26
2015-08-16
2016-01-05
2016-05-20
2016-10-08
2017-02-18
2017-07-02
2017-11-11
2018-03-24

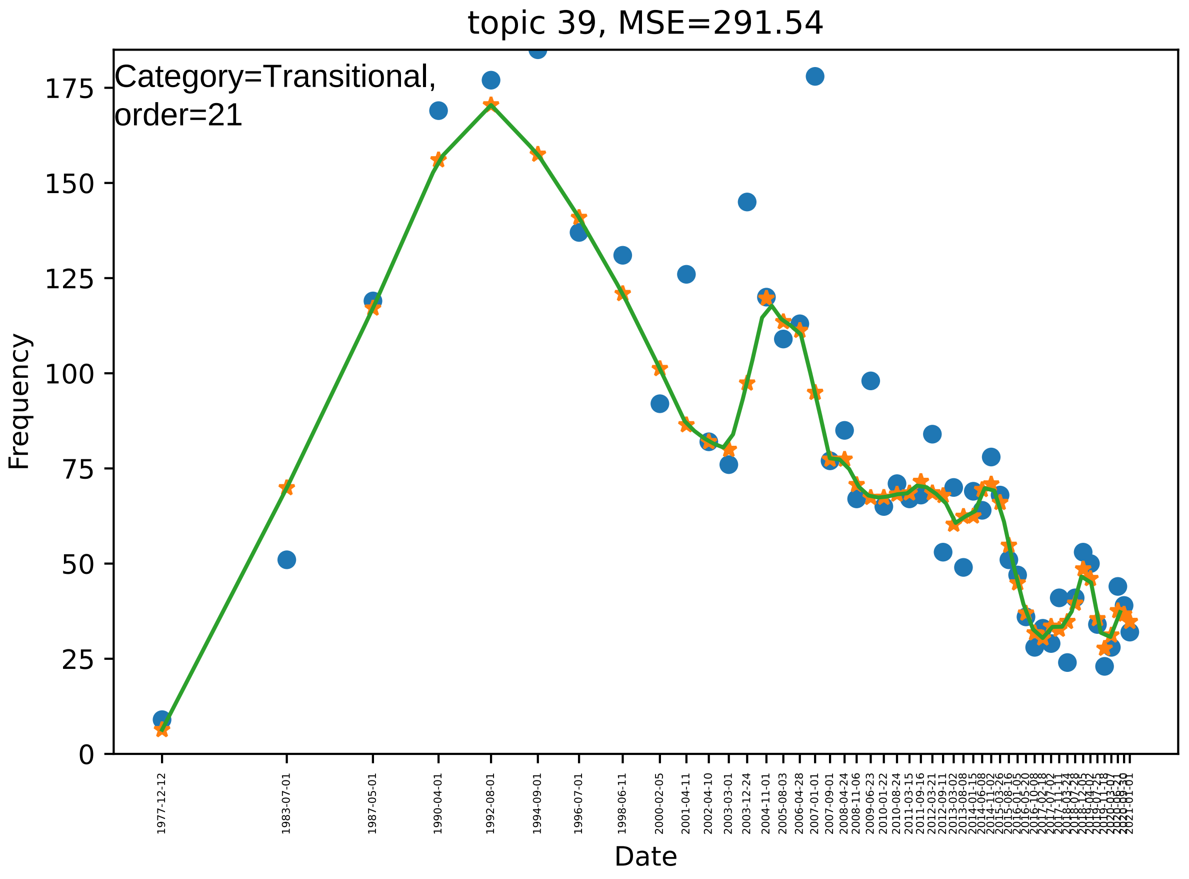

topic 39, MSE=291.54
Category=Transitional,
order=21
Frequency
0
25
50
75
100
125
150
175
Date
1977-12-12
1983-07-01
1987-05-01
1990-04-01
1992-08-01
1994-09-01
1996-07-01
1998-06-11
2000-02-05
2001-04-11
2002-04-10
2003-03-01
2003-12-24
2004-11-01
2005-08-03
2006-04-28
2007-01-01
2007-09-01
2008-04-24
2008-11-06
2009-06-23
2010-01-22
2010-08-24
2011-03-15
2011-09-16
2012-03-21
2012-09-11
2013-03-02
2013-08-08
2014-01-15
2014-06-08
2014-11-02
2015-03-26
2015-08-16
2016-01-05
2016-05-20
2016-10-08
2021-01-01

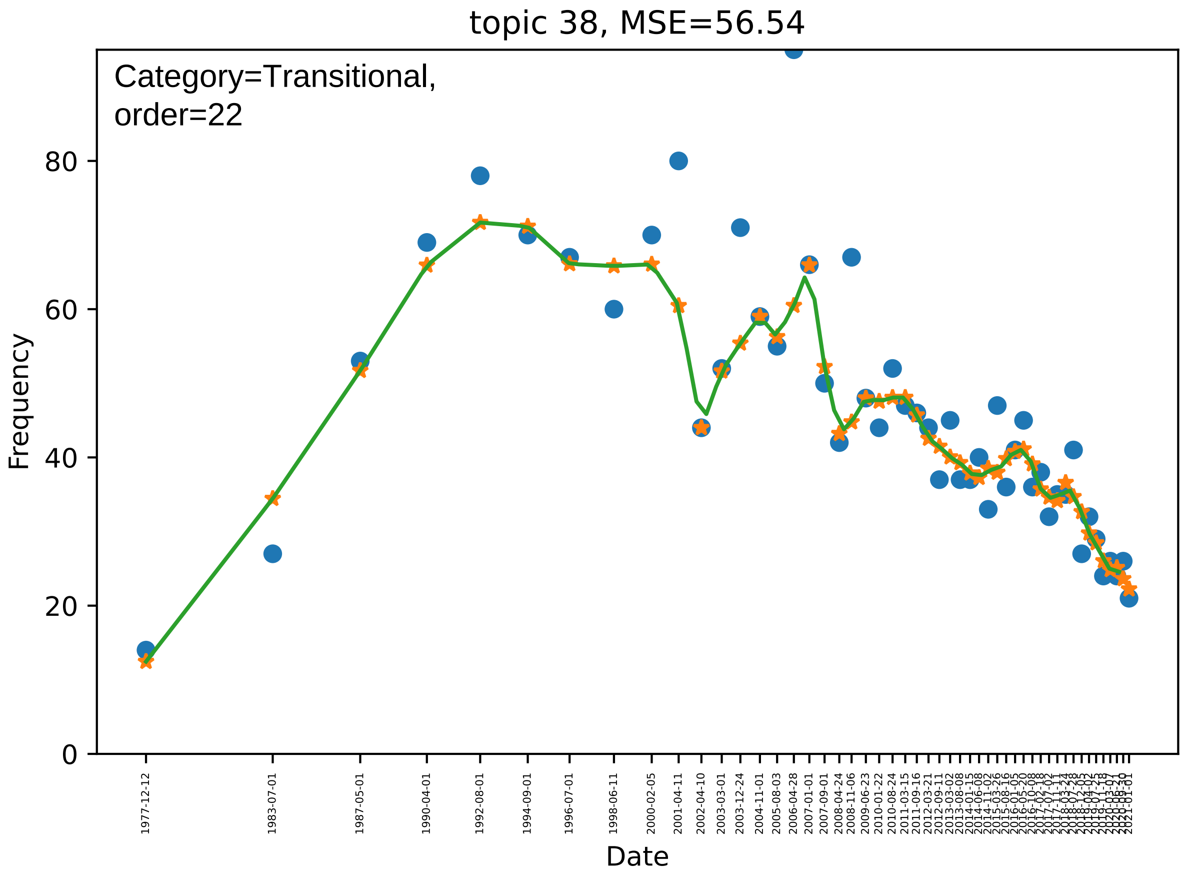
topic 38, MSE=56.54
Category=Transitional,
order=22
Frequency
0
20
40
60
80
Date
1977-12-12
1983-07-01
1987-05-01
1990-04-01
1992-08-01
1994-09-01
1996-07-01
1998-06-11
2000-02-05
2001-04-11
2002-04-10
2003-03-01
2003-12-24
2004-11-01
2005-08-03
2006-04-28
2007-01-01
2007-09-01
2008-04-24
2008-11-06
2009-06-23
2010-01-22
2010-08-24
2011-03-15
2011-09-16
2012-03-21
2012-09-11
2013-03-02
2013-08-08
2014-01-15
2014-06-08
2014-11-02
2015-03-26
2015-08-16
2016-01-05
2016-05-20
2016-10-08
2017-02-18
2017-07-02
2017-11-11
2018-03-24
2018-07-28
2018-12-05
2019-04-02
2019-07-25
2019-11-18
2020-03-07
2021-01-01

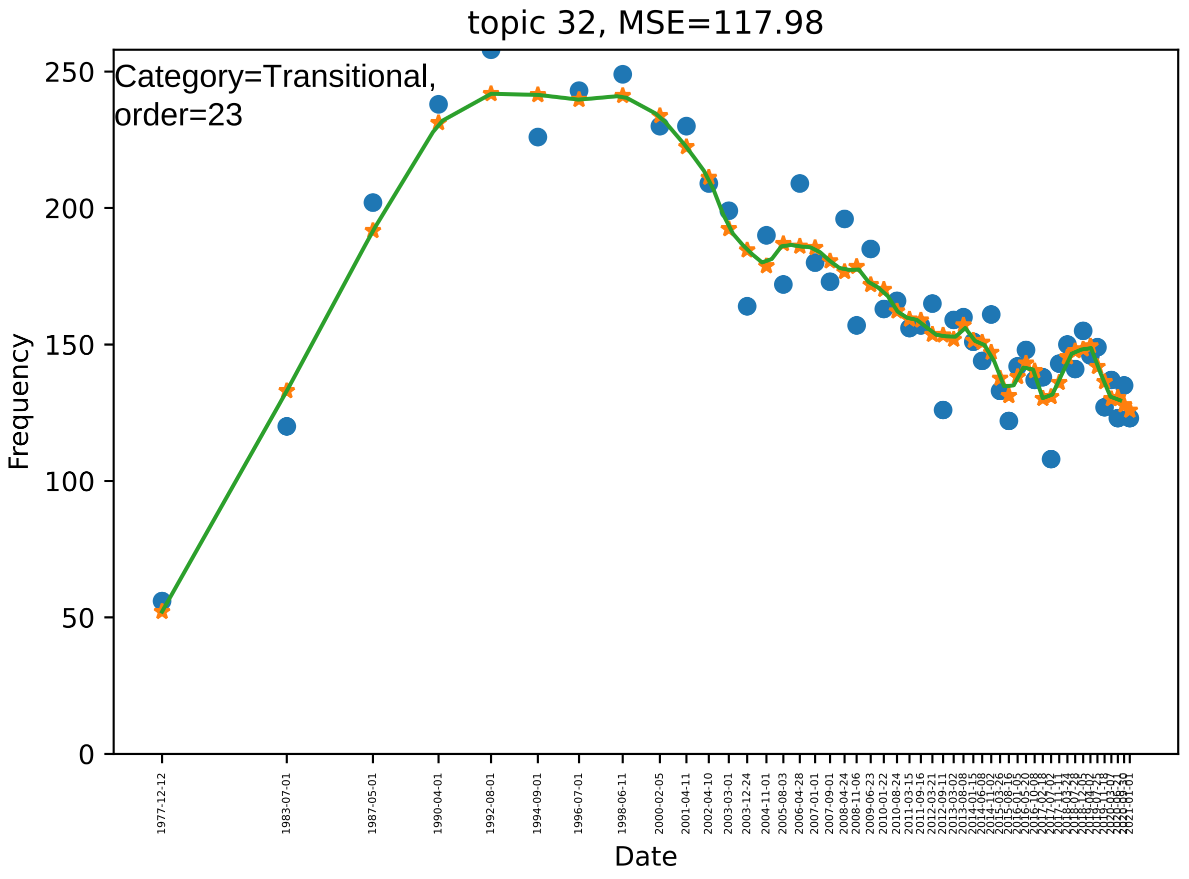

topic 32, MSE=117.98
Category=Transitional,
order=23
Frequency
250
200
150
100
50
0
Date
1977-12-12
1983-07-01
1987-05-01
1990-04-01
1992-08-01
1994-09-01
1996-07-01
1998-06-11
2000-02-05
2001-04-11
2002-04-10
2003-03-01
2003-12-24
2004-11-01
2005-08-03
2006-04-28
2007-01-01
2007-09-01
2008-04-24
2008-11-06
2009-06-23
2010-01-22
2010-08-24
2011-03-15
2011-09-16
2012-03-21
2012-09-11
2013-03-02
2013-08-08
2014-01-15
2014-06-08
2014-11-02
2015-03-26
2015-08-16
2016-01-05
2016-05-20
2016-10-08
2017-02-18
2017-07-02

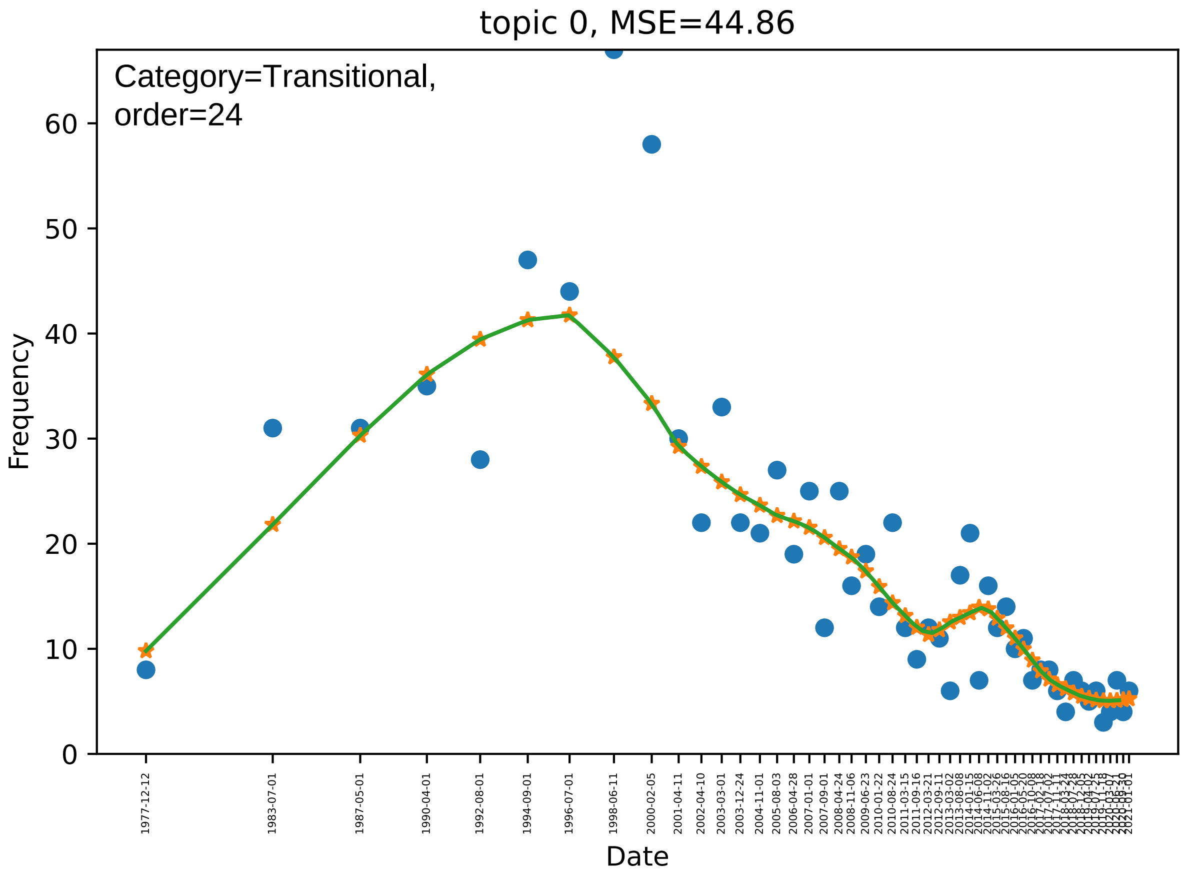
topic 0, MSE=44.86
Category=Transitional,
order=24
Frequency
0
10
20
30
40
50
60
Date
1977-12-12
1983-07-01
1987-05-01
1990-04-01
1992-08-01
1994-09-01
1996-07-01
1998-06-11
2000-02-05
2001-04-11
2002-04-10
2003-03-01
2003-12-24
2004-11-01
2005-08-03
2006-04-28
2007-01-01
2007-09-01
2008-04-24
2008-11-06
2009-06-23
2010-01-22
2010-08-24
2011-03-15
2011-09-16
2012-03-21
2012-09-11
2013-03-02

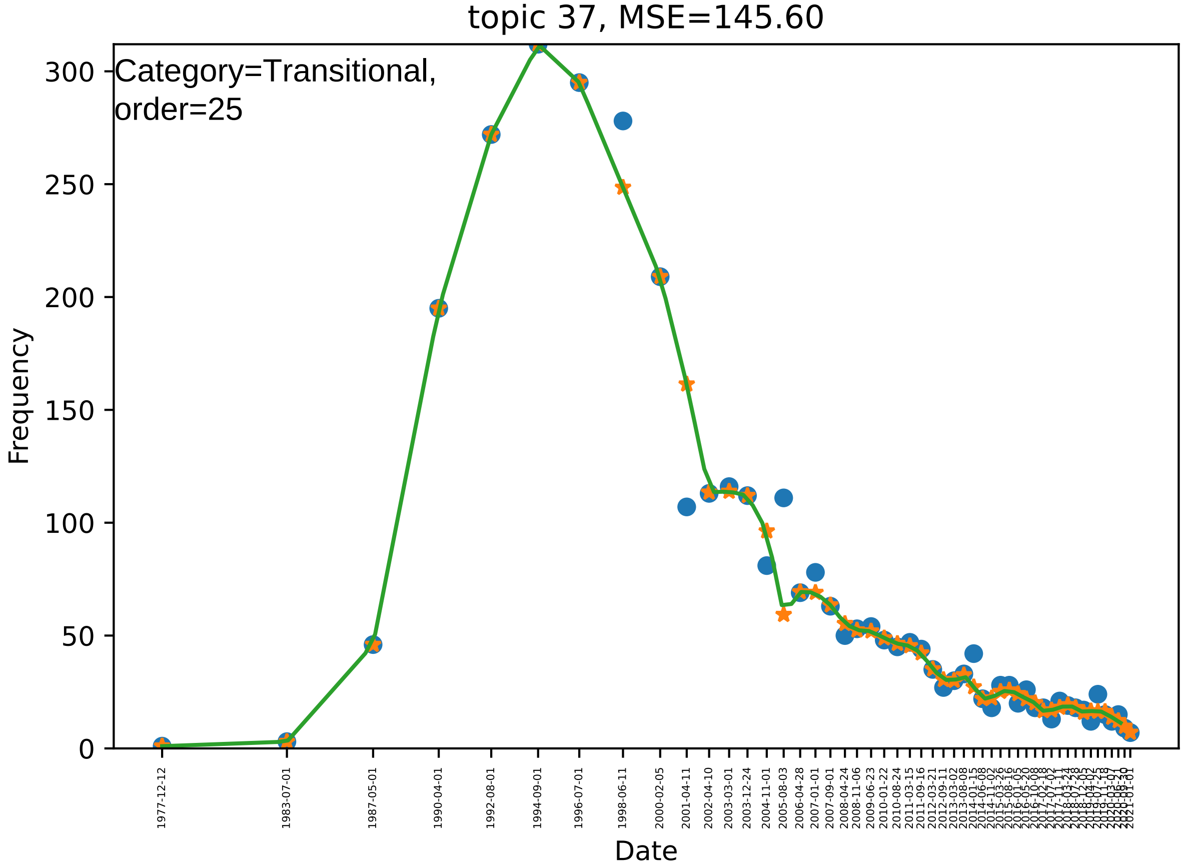

topic 37, MSE=145.60
Category=Transitional,
order=25
Frequency
Date
0
50
100
150
200
250
300
1977-12-12
1983-07-01
1987-05-01
1990-04-01
1992-08-01
1994-09-01
1996-07-01
1998-06-11
2000-02-05
2001-04-11
2002-04-10
2003-03-01
2003-12-24
2004-11-01
2005-08-03
2006-04-28
2007-01-01
2007-09-01
2008-04-24
2008-11-06
2009-06-23
2010-01-22
2010-08-24
2011-03-15
2011-09-16
2012-03-21
2012-09-11
2013-03-02
2013-08-08
2014-01-15
2014-06-08
2014-11-02
2015-03-26
2015-08-16
2016-01-05
2016-05-20
2016-10-08

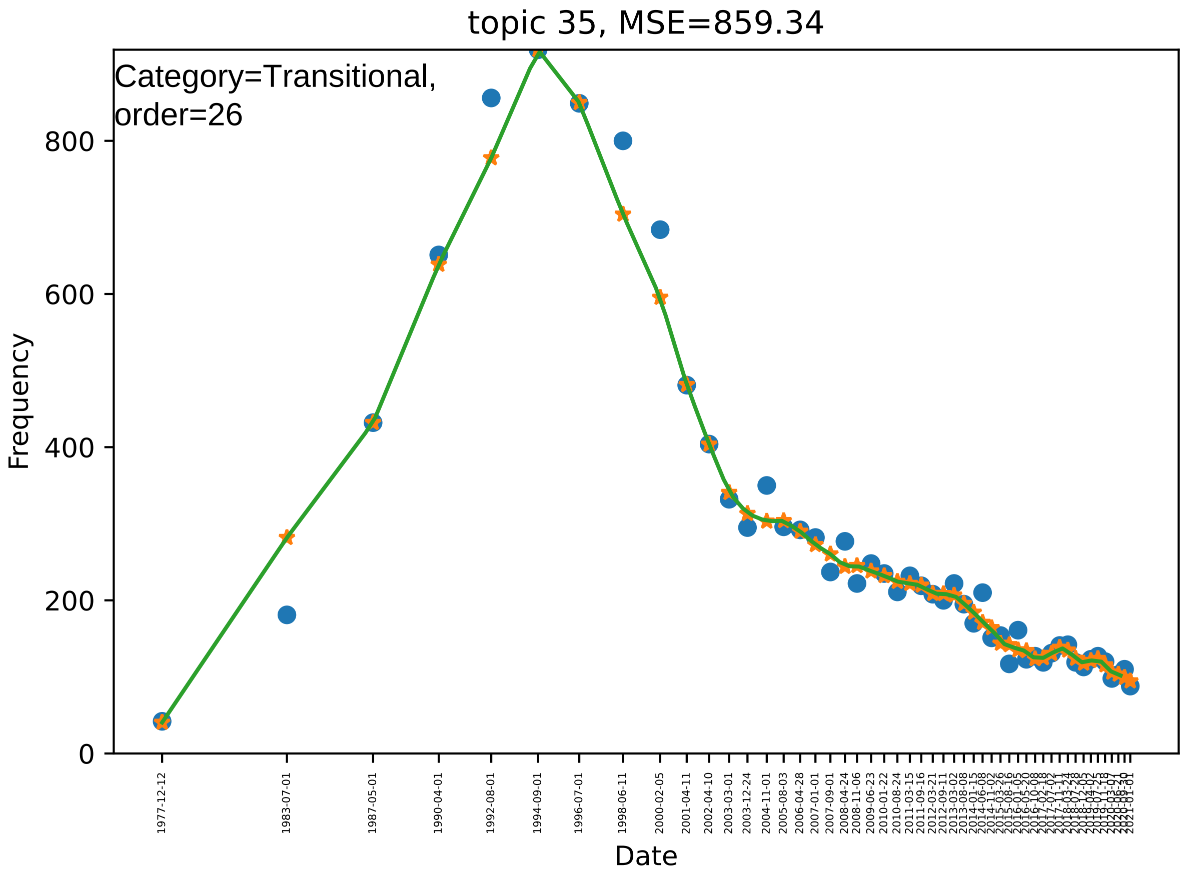
topic 35, MSE=859.34
Category=Transitional,
order=26
Frequency
0
200
400
600
800
Date
1977-12-12
1983-07-01
1987-05-01
1990-04-01
1992-08-01
1994-09-01
1996-07-01
1998-06-11
2000-02-05
2001-04-11
2002-04-10
2003-03-01
2003-12-24
2004-11-01
2005-08-03
2006-04-28
2007-01-01
2007-09-01
2008-04-24
2008-11-06
2009-06-23
2010-01-22
2010-08-24
2011-03-15
2011-09-16
2012-03-21
2012-09-11
2013-03-02
2021-01-01

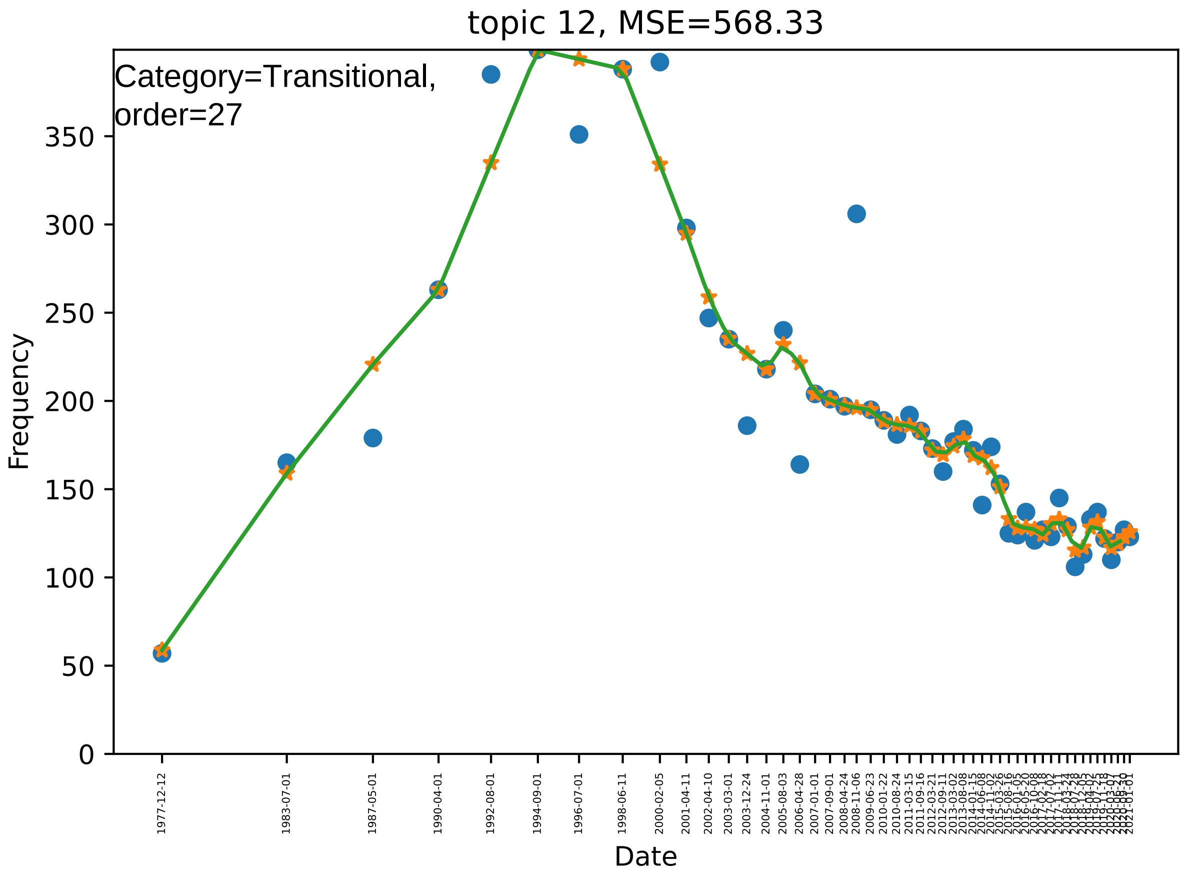
topic 12, MSE=568.33
Category=Transitional,
order=27
Frequency
0
50
100
150
200
250
300
350
Date
1977-12-12
1983-07-01
1987-05-01
1990-04-01
1992-08-01
1994-09-01
1996-07-01
1998-06-11
2000-02-05
2001-04-11
2002-04-10
2003-03-01
2003-12-24
2004-11-01
2005-08-03
2006-04-28
2007-01-01
2007-09-01
2008-04-24
2008-11-06
2009-06-23
2010-01-22
2010-08-24
2011-03-15
2011-09-16
2012-03-21
2012-09-11
2013-03-02
2013-08-08
2014-01-15
2014-06-08

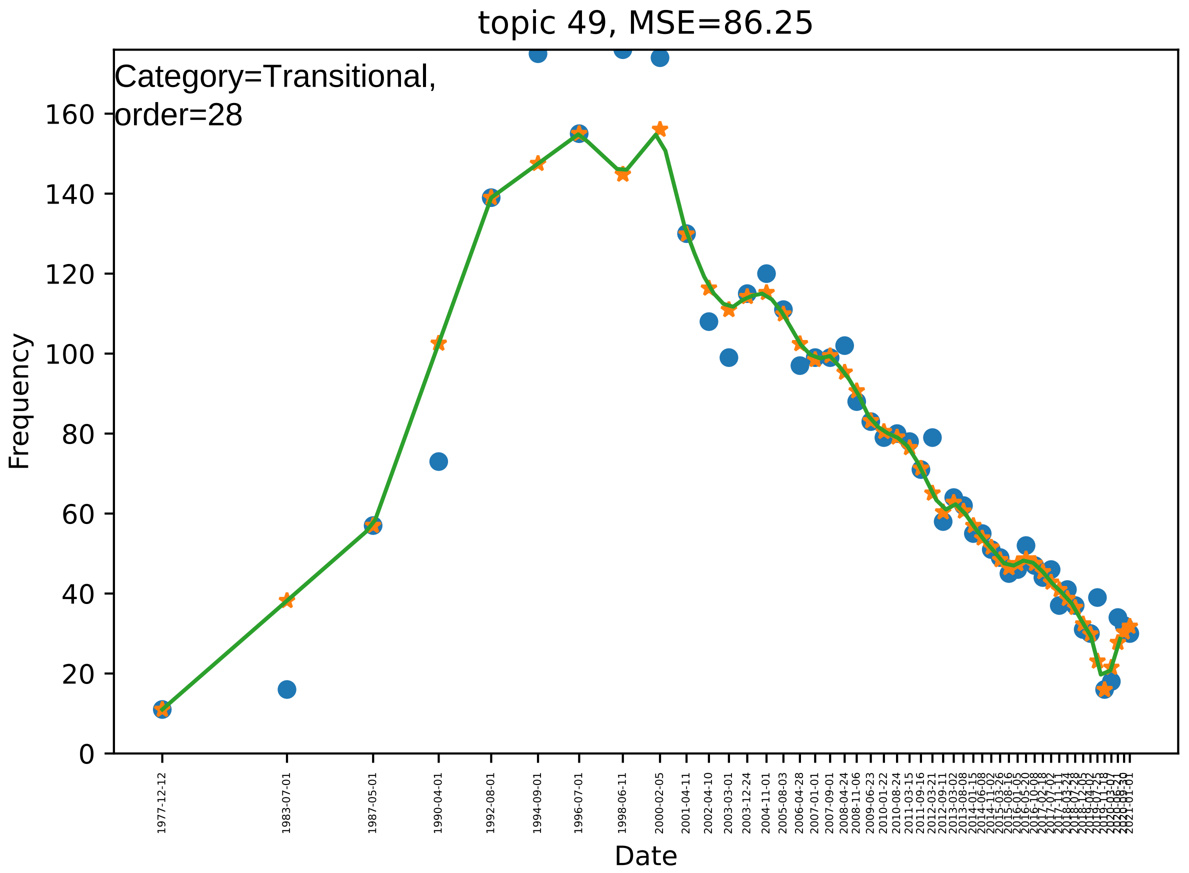
topic 49, MSE=86.25
Category=Transitional,
order=28
Frequency
0
20
40
60
80
100
120
140
160
Date
1977-12-12
1983-07-01
1987-05-01
1990-04-01
1992-08-01
1994-09-01
1996-07-01
1998-06-11
2000-02-05
2001-04-11
2002-04-10
2003-03-01
2003-12-24
2004-11-01
2005-08-03
2006-04-28
2007-01-01
2007-09-01
2008-04-24
2008-11-06
2009-06-23
2010-01-22
2010-08-24
2011-03-15
2011-09-16
2012-03-21
2012-09-11
2013-03-02
2013-08-08
2014-01-15
2014-06-08
2014-11-02
2015-03-26
2015-08-16
2016-01-05
2016-05-20
2016-10-08
2017-02-18
2017-07-02
2017-11-11

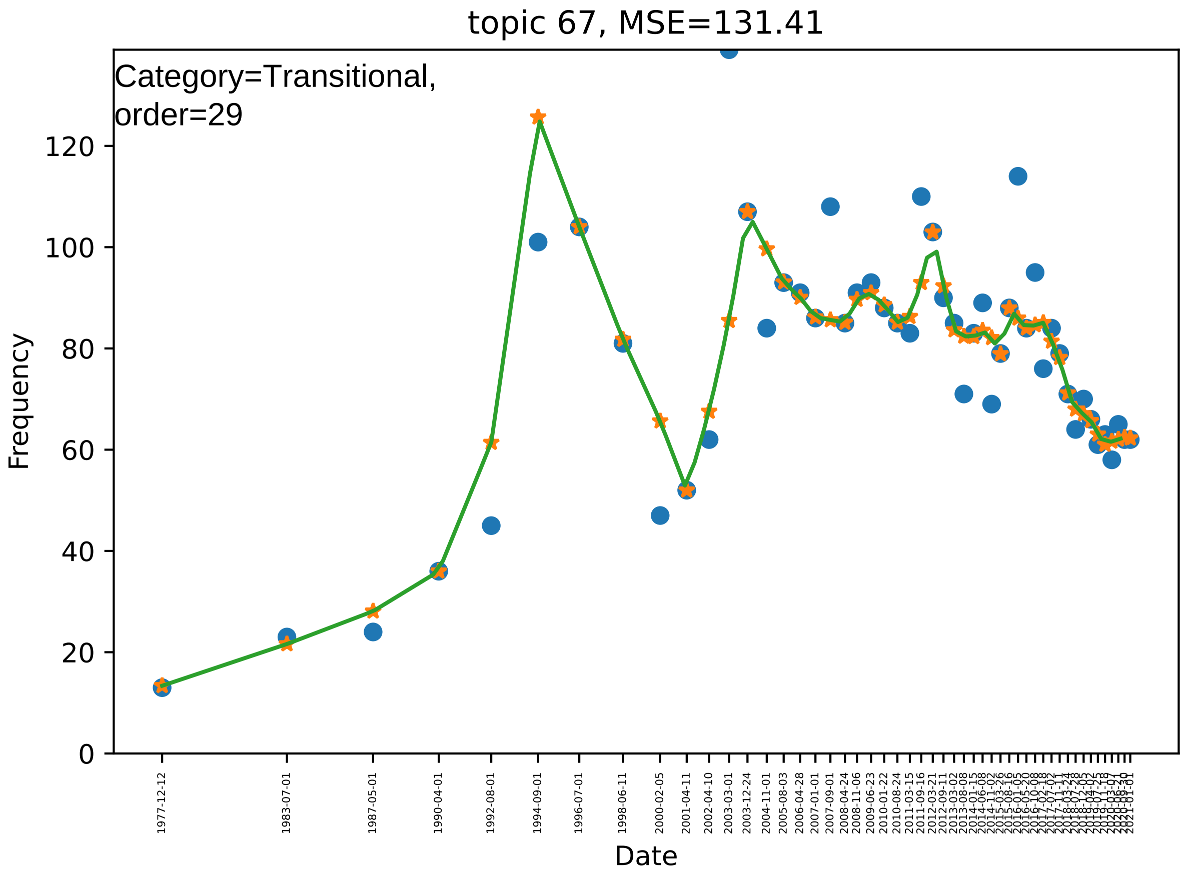
topic 67, MSE=131.41
Category=Transitional,
order=29
Frequency
0
20
40
60
80
100
120
Date
1977-12-12
1983-07-01
1987-05-01
1990-04-01
1992-08-01
1994-09-01
1996-07-01
1998-06-11
2000-02-05
2001-04-11
2002-04-10
2003-03-01
2003-12-24
2004-11-01
2005-08-03
2006-04-28
2007-01-01
2007-09-01
2008-04-24
2008-11-06
2009-06-23
2010-01-22
2010-08-24
2011-03-15
2011-09-16
2012-03-21
2012-09-11
2013-03-02
2013-08-08
2014-01-15
2014-06-08
2014-11-02
2015-03-26
2015-08-16
2016-01-05
2016-05-20
2016-10-08
2017-02-18
2017-07-02
2017-11-11

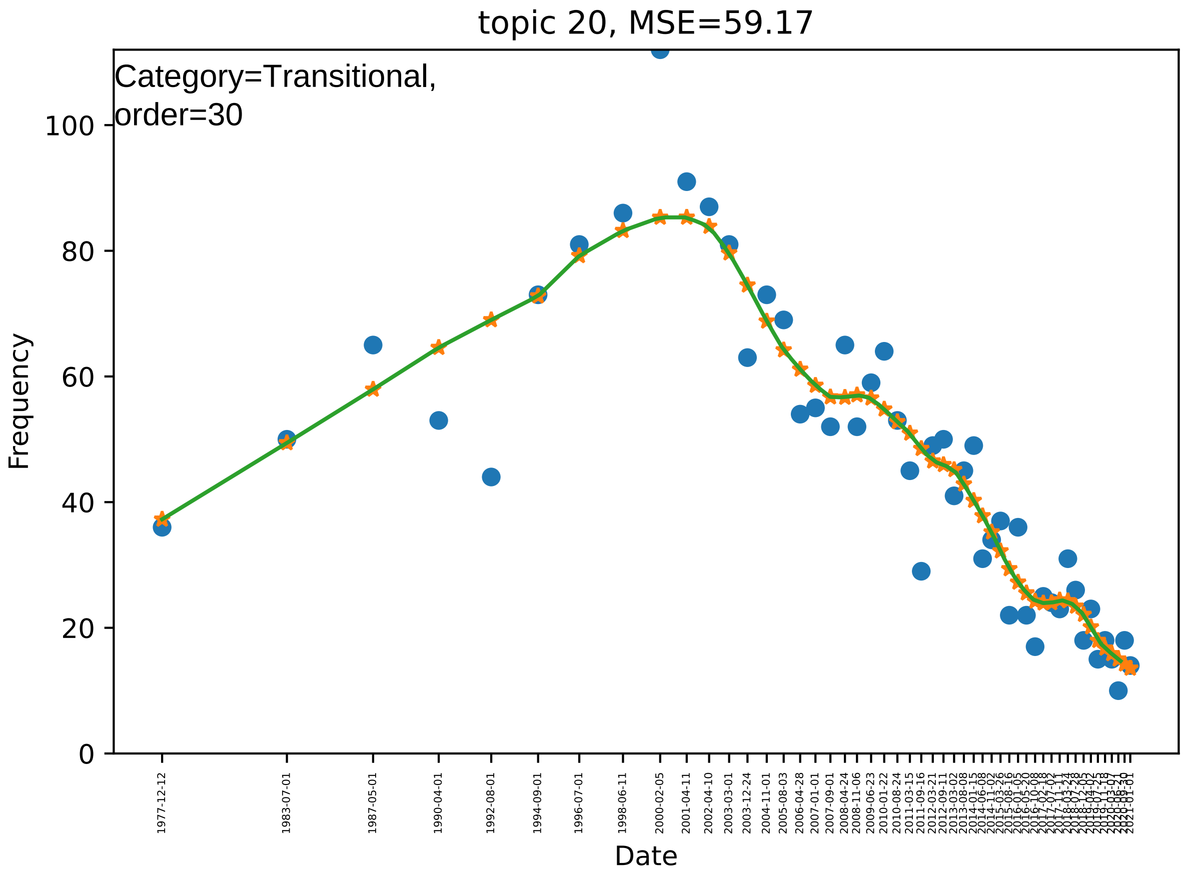
topic 20, MSE=59.17
Category=Transitional,
order=30
Frequency
0
20
40
60
80
100
Date
1977-12-12
1983-07-01
1987-05-01
1990-04-01
1992-08-01
1994-09-01
1996-07-01
1998-06-11
2000-02-05
2001-04-11
2002-04-10
2003-03-01
2003-12-24
2004-11-01
2005-08-03
2006-04-28
2007-01-01
2007-09-01
2008-04-24
2008-11-06
2009-06-23
2010-01-22
2010-08-24
2011-03-15
2011-09-16
2012-03-21
2012-09-11
2013-03-02
2013-08-08
2014-01-15
2014-06-08
2014-11-02
2015-03-26
2015-08-16
2016-01-05
2016-05-20
2016-10-08
2017-02-18
2017-07-02
2021-01-01

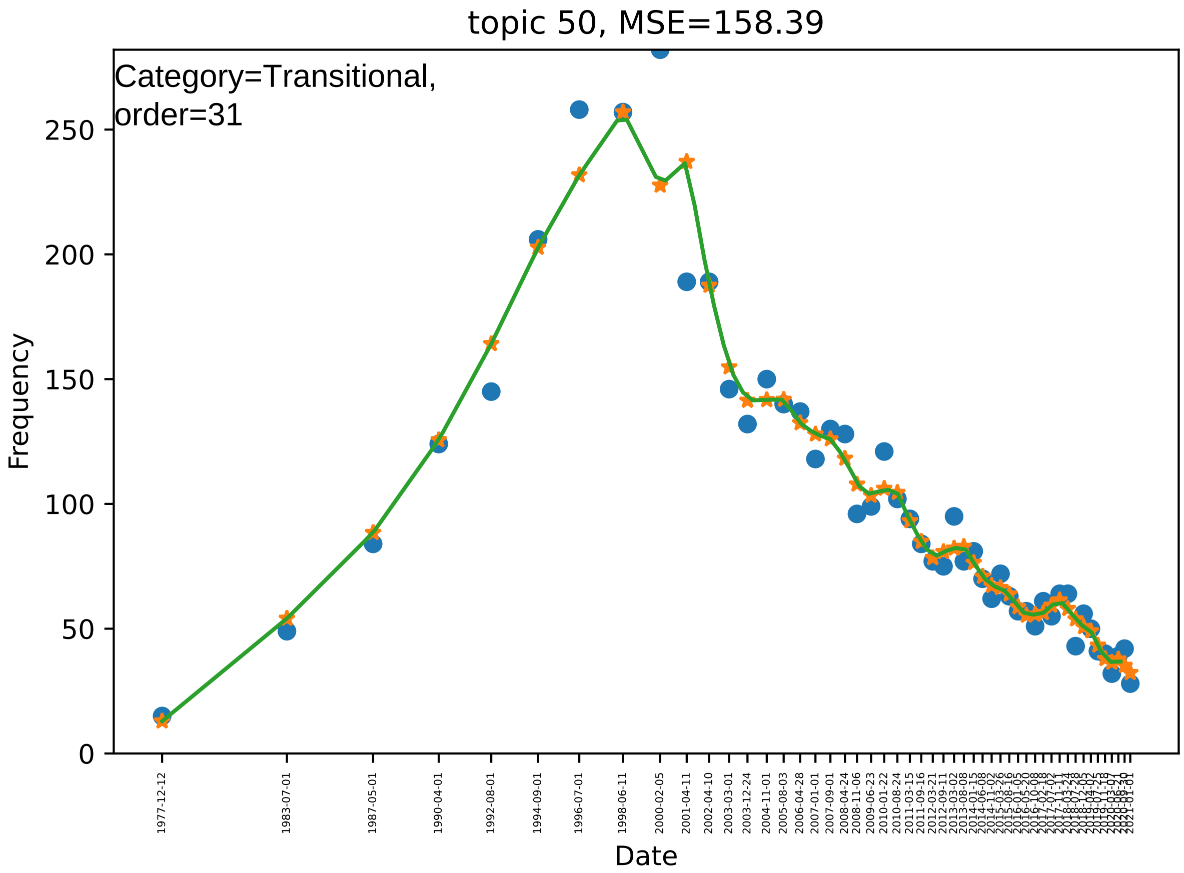

topic 50, MSE=158.39
Category=Transitional,
order=31
Frequency
Date
0
50
100
150
200
250
1977-12-12
1983-07-01
1987-05-01
1990-04-01
1992-08-01
1994-09-01
1996-07-01
1998-06-11
2000-02-05
2001-04-11
2002-04-10
2003-03-01
2003-12-24
2004-11-01
2005-08-03
2006-04-28
2007-01-01
2007-09-01
2008-04-24
2008-11-06
2009-06-23
2010-01-22
2010-08-24
2011-03-15
2011-09-16
2012-03-21
2012-09-11
2013-03-02
2013-08-08
2014-01-15
2014-06-08
2014-11-02
2015-03-26
2015-08-16
2016-01-05
2016-05-20
2016-10-08
2017-02-18
2017-07-02
2021-01-01

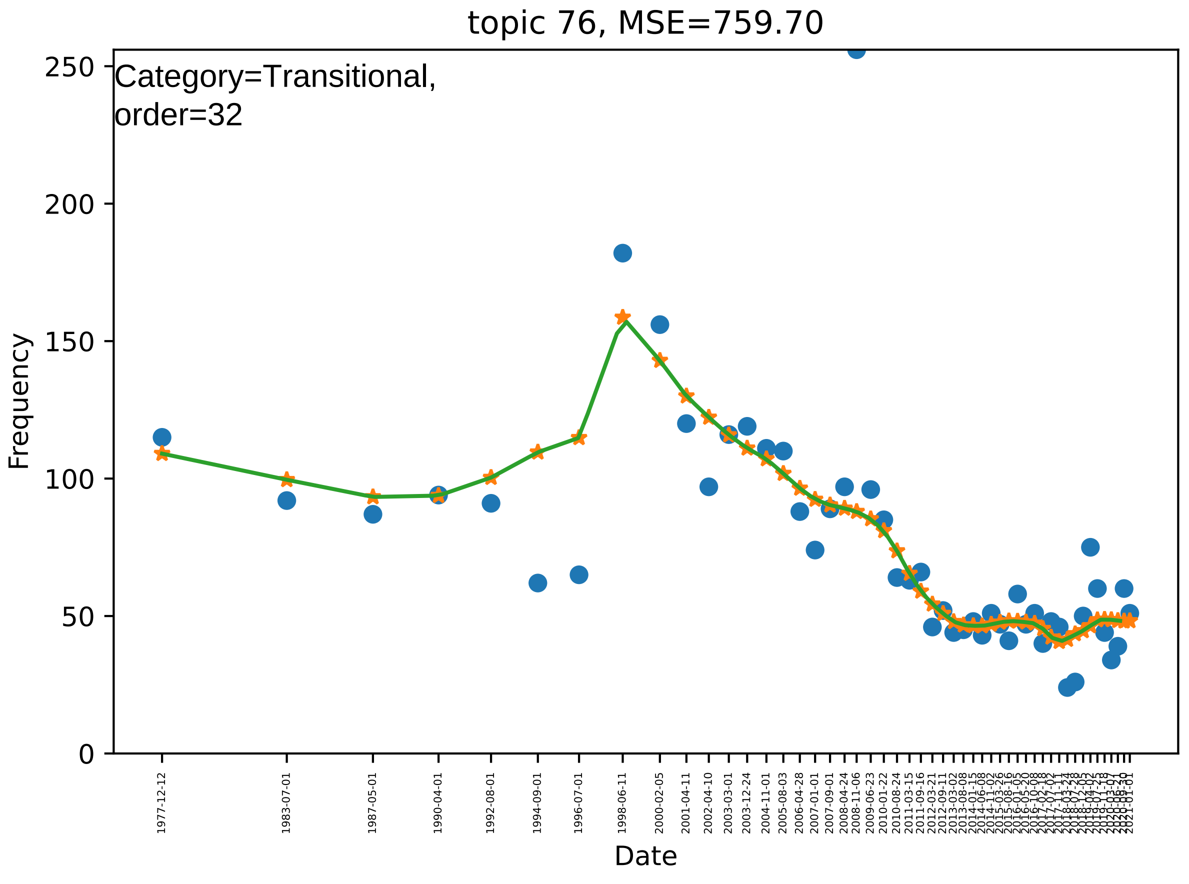

topic 76, MSE=759.70
Category=Transitional,
order=32
Frequency
Date
0
50
100
150
200
250

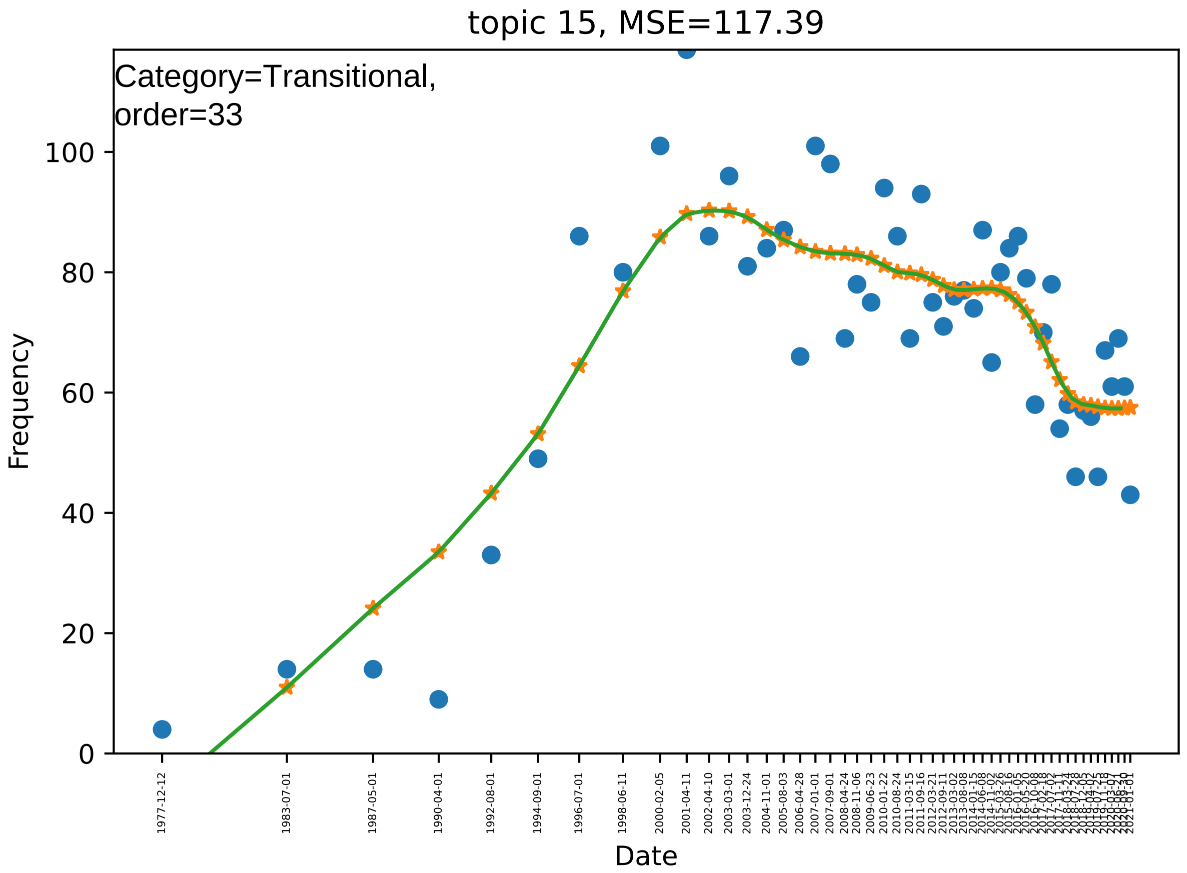

topic 15, MSE=117.39
Category=Transitional,
order=33
Frequency
0
20
40
60
80
100
Date
1977-12-12
1983-07-01
1987-05-01
1990-04-01
1992-08-01
1994-09-01
1996-07-01
1998-06-11
2000-02-05
2001-04-11
2002-04-10
2003-03-01
2003-12-24
2004-11-01
2005-08-03
2006-04-28
2007-01-01
2007-09-01
2008-04-24
2008-11-06
2009-06-23
2010-01-22
2010-08-24
2011-03-15
2011-09-16
2012-03-21
2012-09-11
2013-03-02
2013-08-08
2014-01-15
2014-06-08
2014-11-02
2015-03-26
2015-08-16
2016-01-05
2016-05-20
2016-10-08

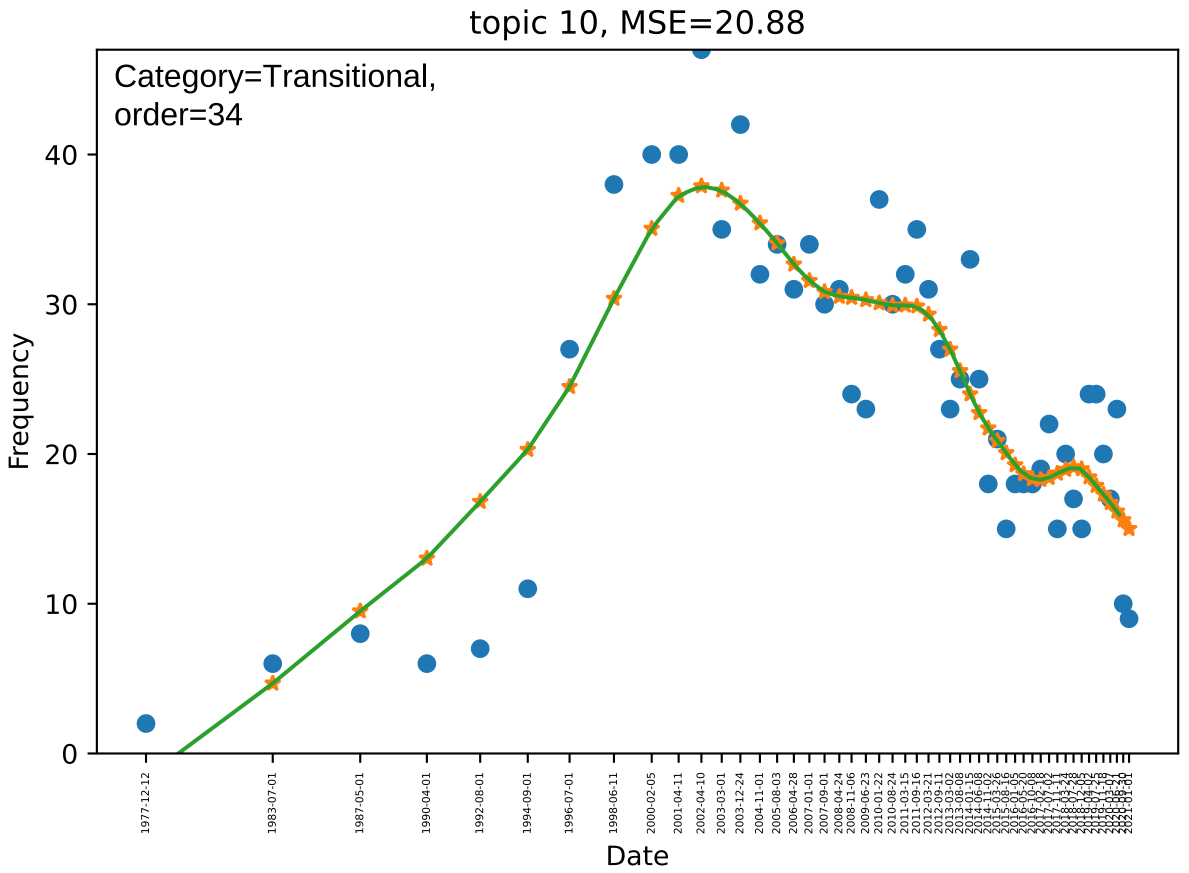
topic 10, MSE=20.88
Category=Transitional,
order=34
Frequency
Date
0
10
20
30
40
1977-12-12
1983-07-01
1987-05-01
1990-04-01
1992-08-01
1994-09-01
1996-07-01
1998-06-11
2000-02-05
2001-04-11
2002-04-10
2003-03-01
2003-12-24
2004-11-01
2005-08-03
2006-04-28
2007-01-01
2007-09-01
2008-04-24
2008-11-06
2009-06-23
2010-01-22
2010-08-24
2011-03-15
2011-09-16
2012-03-21
2012-09-11
2013-03-02
2013-08-08
2014-01-15
2014-06-08
2014-11-02

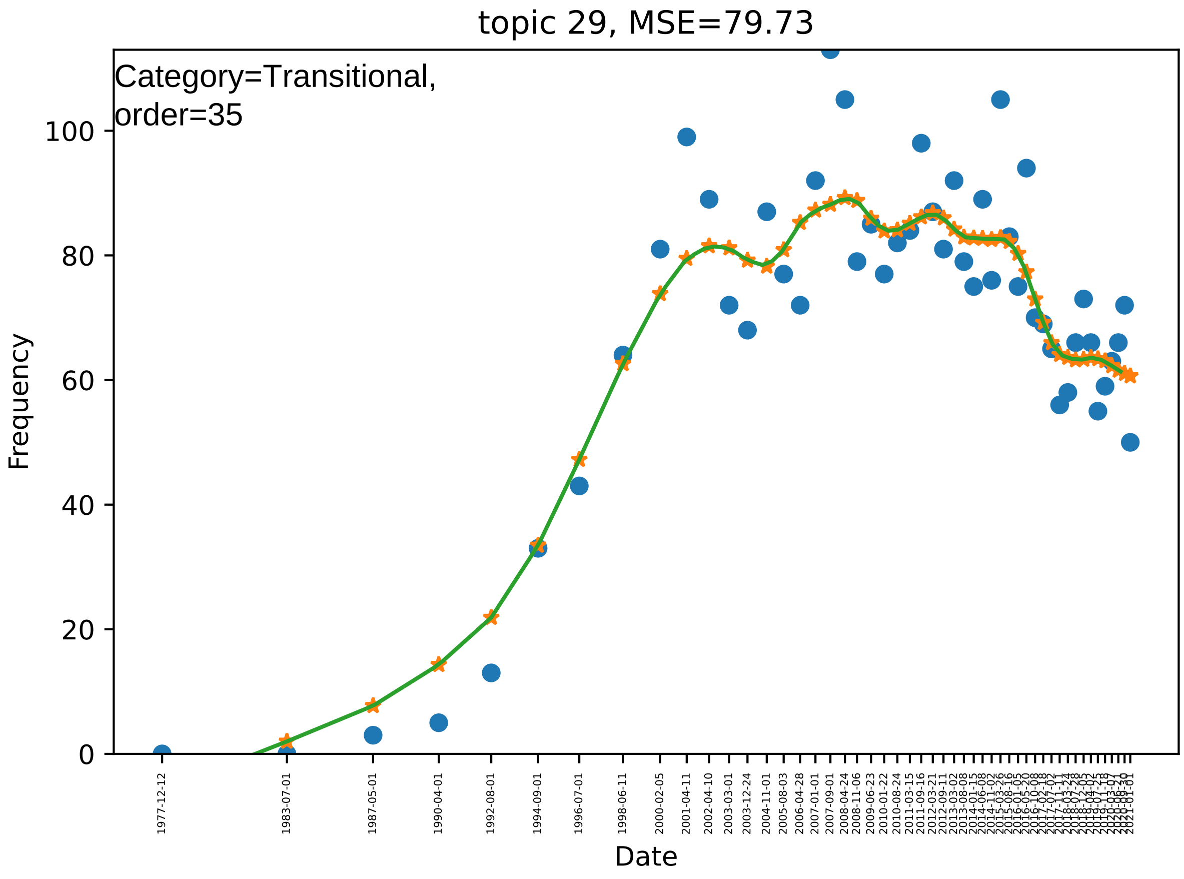

topic 29, MSE=79.73
Category=Transitional,
order=35
Frequency
Date
0
20
40
60
80
100
1977-12-12
1983-07-01
1987-05-01
1990-04-01
1992-08-01
1994-09-01
1996-07-01
1998-06-11
2000-02-05
2001-04-11
2002-04-10
2003-03-01
2003-12-24
2004-11-01
2005-08-03
2006-04-28
2007-01-01
2007-09-01
2008-04-24
2008-11-06
2009-06-23
2010-01-22
2010-08-24
2011-03-15
2011-09-16
2012-03-21
2012-09-11
2013-03-02
2013-08-08
2014-01-15
2014-06-08
2014-11-02
2015-03-26
2015-08-16
2016-01-05
2016-05-20
2016-10-08

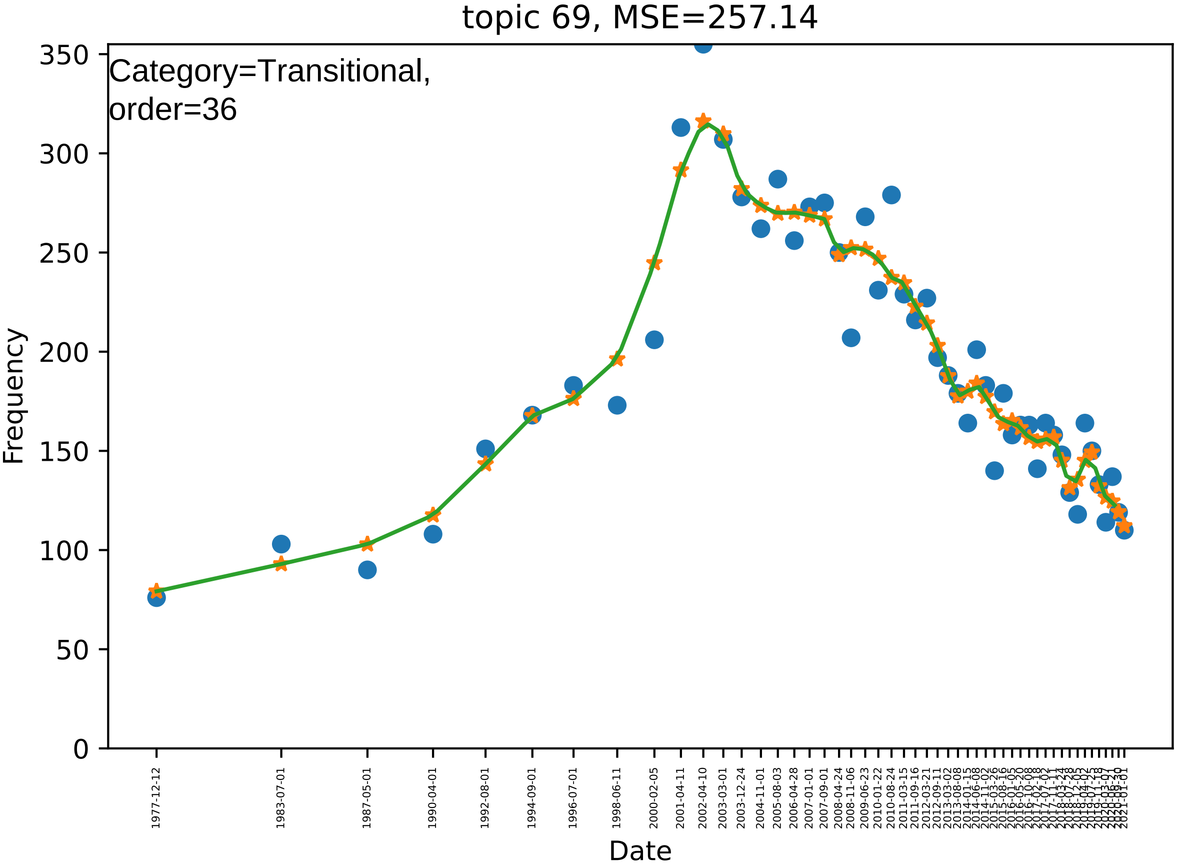

topic 69, MSE=257.14
Category=Transitional,
order=36
Frequency
0
50
100
150
200
250
300
350
Date
1977-12-12
1983-07-01
1987-05-01
1990-04-01
1992-08-01
1994-09-01
1996-07-01
1998-06-11
2000-02-05
2001-04-11
2002-04-10
2003-03-01
2003-12-24
2004-11-01
2005-08-03
2006-04-28
2007-01-01
2007-09-01
2008-04-24
2008-11-06
2009-06-23
2010-01-22
2010-08-24
2011-03-15
2011-09-16

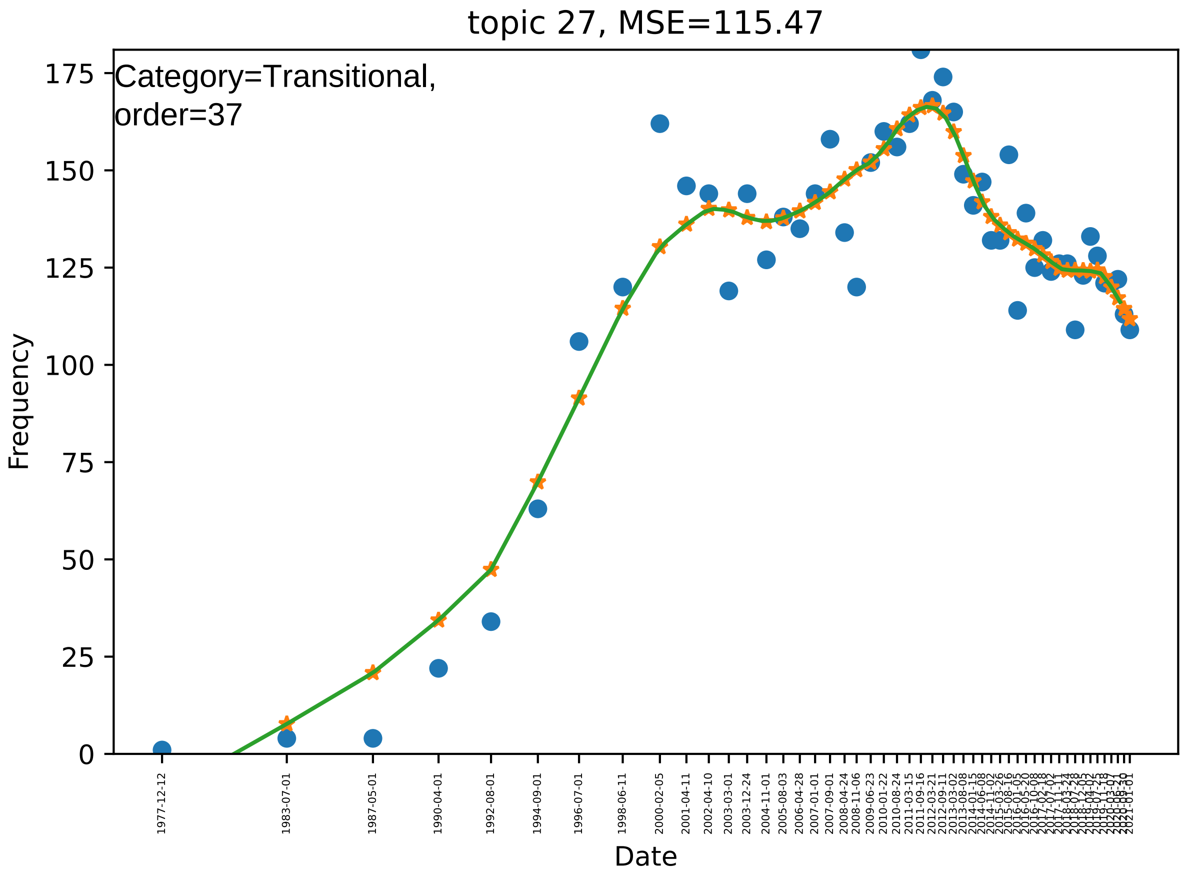
topic 27, MSE=115.47
Category=Transitional,
order=37
Frequency
0
25
50
75
100
125
150
175
Date
1977-12-12
1983-07-01
1987-05-01
1990-04-01
1992-08-01
1994-09-01
1996-07-01
1998-06-11
2000-02-05
2001-04-11
2002-04-10
2003-03-01
2003-12-24
2004-11-01
2005-08-03
2006-04-28
2007-01-01
2007-09-01
2008-04-24
2008-11-06
2009-06-23
2010-01-22
2010-08-24
2011-03-15
2011-09-16
2012-03-21
2012-09-11
2013-03-02
2013-08-08
2014-01-15
2014-06-08
2014-11-02
2015-03-26
2015-08-16
2016-01-05
2016-05-20
2016-10-08
2017-02-18
2017-07-02
2017-11-11
2018-03-24

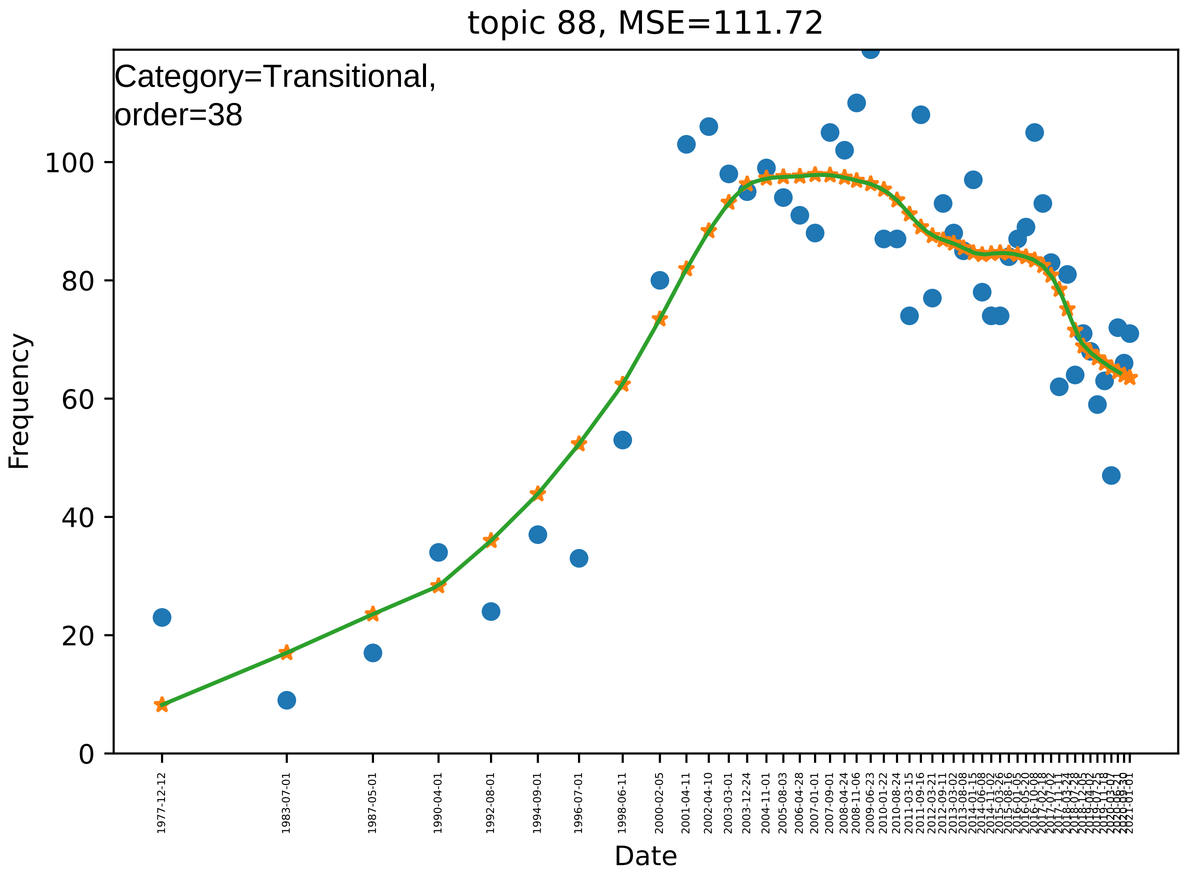
topic 88, MSE=111.72
Category=Transitional,
order=38
Frequency
0
20
40
60
80
100
Date
1977-12-12
1983-07-01
1987-05-01
1990-04-01
1992-08-01
1994-09-01
1996-07-01
1998-06-11
2000-02-05
2001-04-11
2002-04-10
2003-03-01
2003-12-24
2004-11-01
2005-08-03
2006-04-28
2007-01-01
2007-09-01
2008-04-24
2008-11-06
2009-06-23
2010-01-22
2010-08-24
2011-03-15
2011-09-16
2012-03-21
2012-09-11
2013-03-02
2013-08-08
2014-01-15
2014-06-08
2014-11-02
2015-03-26
2015-08-16
2016-01-05
2016-05-20
2016-10-08
2017-02-18
2017-07-02
2017-11-11

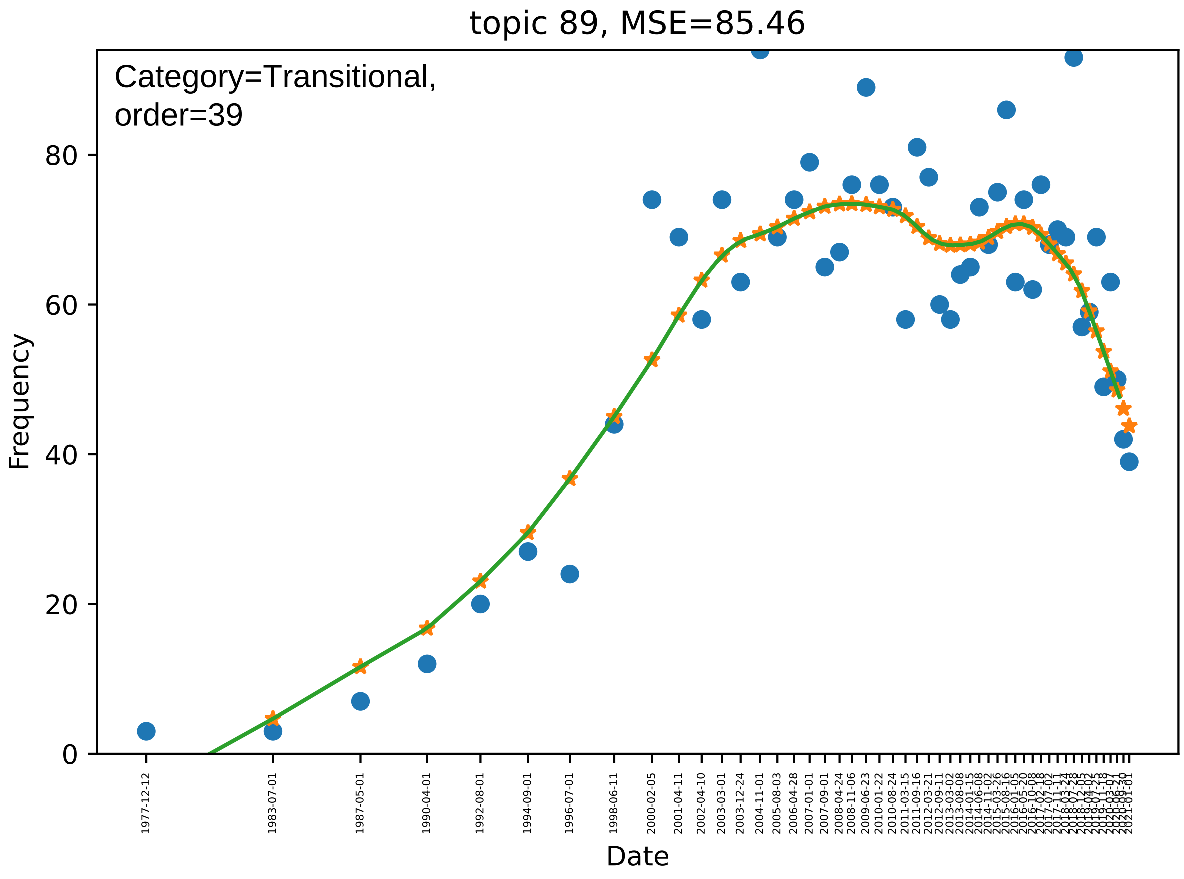

topic 89, MSE=85.46
Category=Transitional,
order=39
Frequency
0
20
40
60
80
Date
1977-12-12
1983-07-01
1987-05-01
1990-04-01
1992-08-01
1994-09-01
1996-07-01
1998-06-11
2000-02-05
2001-04-11
2002-04-10
2003-03-01
2003-12-24
2004-11-01
2005-08-03
2006-04-28
2007-01-01
2007-09-01
2008-04-24
2008-11-06
2009-06-23
2010-01-22
2010-08-24
2011-03-15
2011-09-16
2012-03-21
2012-09-11
2013-03-02
2013-08-08
2014-01-15
2014-06-08
2014-11-02

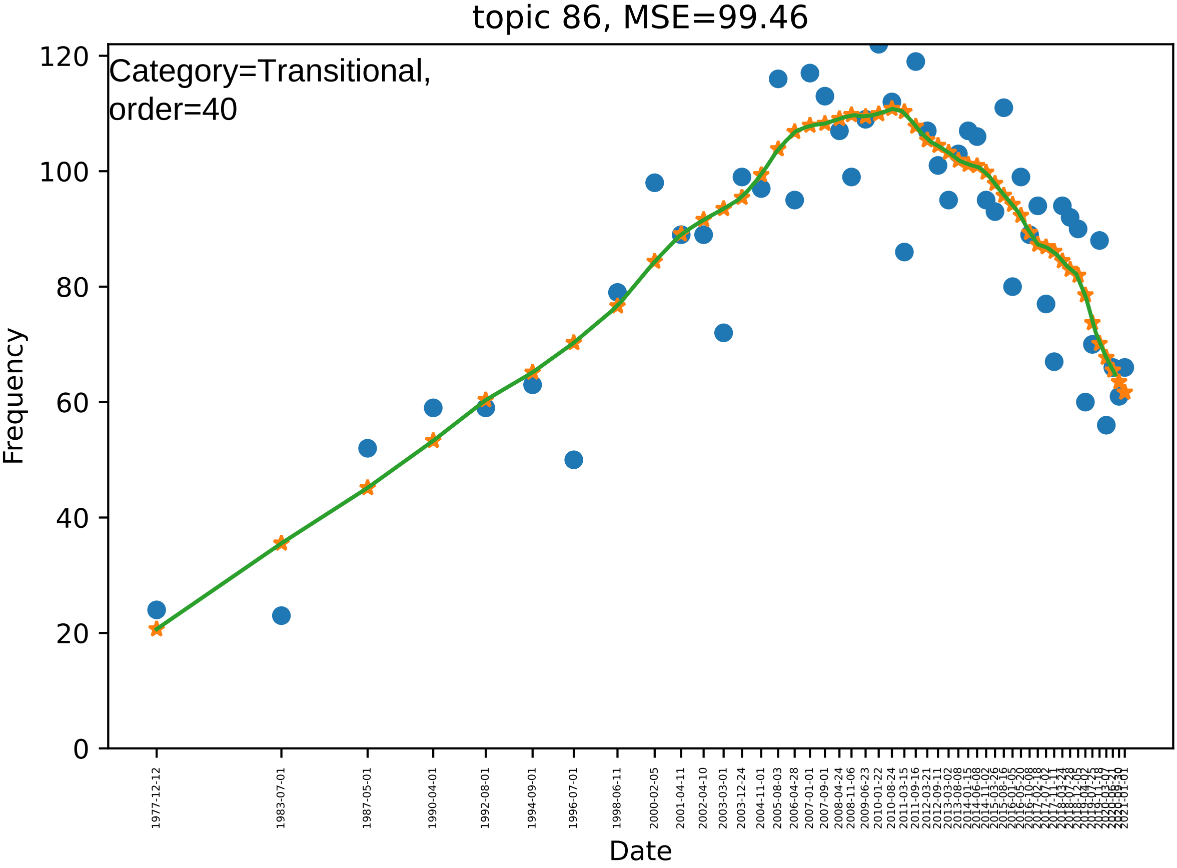

topic 86, MSE=99.46
Category=Transitional,
order=40
Frequency
Date
0
20
40
60
80
100
120
1977-12-12
1983-07-01
1987-05-01
1990-04-01
1992-08-01
1994-09-01
1996-07-01
1998-06-11
2000-02-05
2001-04-11
2002-04-10
2003-03-01
2003-12-24
2004-11-01
2005-08-03
2006-04-28
2007-01-01
2007-09-01
2008-04-24
2008-11-06
2009-06-23
2010-01-22
2010-08-24
2011-03-15
2011-09-16
2012-03-21
2012-09-11
2013-03-02
2013-08-08
2014-01-15
2014-06-08
2014-11-02
2015-03-26
2015-08-16
2016-01-05
2016-05-20
2016-10-08
2017-02-18
2017-07-02
2021-01-01

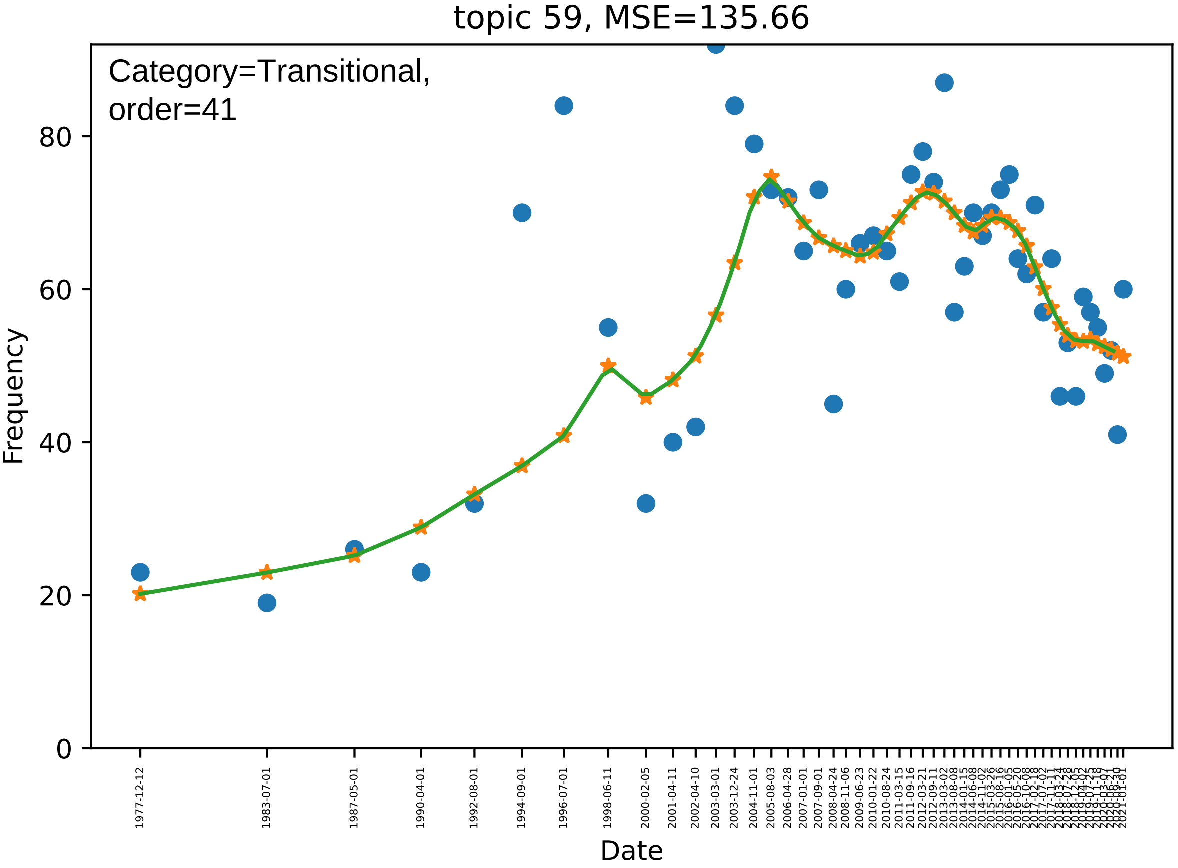
topic 59, MSE=135.66
Category=Transitional,
order=41
Frequency
Date
0
20
40
60
80
1977-12-12
1983-07-01
1987-05-01
1990-04-01
1992-08-01
1994-09-01
1996-07-01
1998-06-11
2000-02-05
2001-04-11
2002-04-10
2003-03-01
2003-12-24
2004-11-01
2005-08-03
2006-04-28
2007-01-01
2007-09-01
2008-04-24
2008-11-06
2009-06-23
2010-01-22
2010-08-24
2011-03-15
2011-09-16
2012-03-21
2012-09-11
2013-03-02
2013-08-08
2014-01-15
2014-06-08
2014-11-02
2015-03-26
2015-08-16
2016-01-05
2016-05-20
2016-10-08
2017-02-18
2017-07-02
2017-11-11
2018-03-24

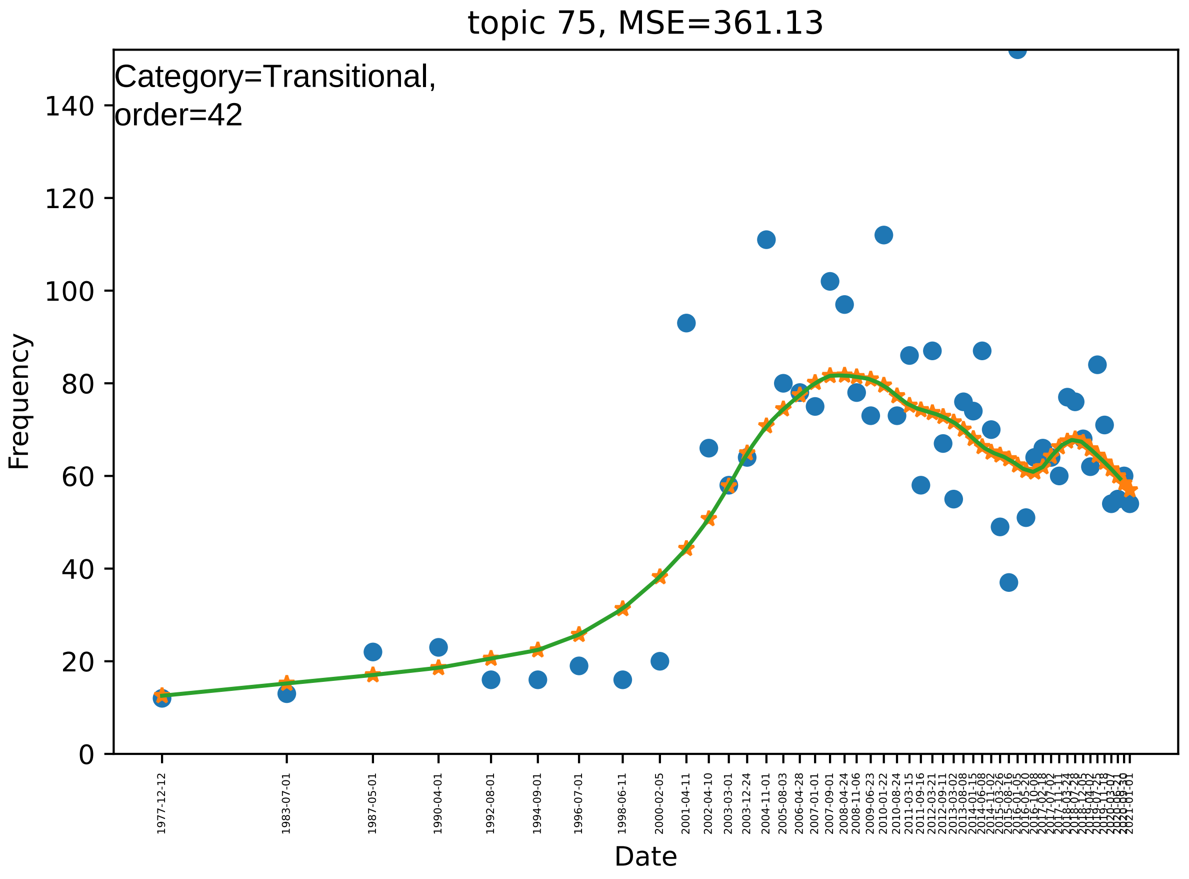

topic 75, MSE=361.13
Category=Transitional,
order=42
Frequency
Date
0
20
40
60
80
100
120
140
1977-12-12
1983-07-01
1987-05-01
1990-04-01
1992-08-01
1994-09-01
1996-07-01
1998-06-11
2000-02-05
2001-04-11
2002-04-10
2003-03-01
2003-12-24
2004-11-01
2005-08-03
2006-04-28
2007-01-01
2007-09-01
2008-04-24
2008-11-06
2009-06-23
2010-01-22
2010-08-24
2011-03-15
2011-09-16
2012-03-21
2012-09-11
2013-03-02
2013-08-08
2014-01-15
2014-06-08
2014-11-02
2015-03-26
2015-08-16
2016-01-05
2016-05-20
2016-10-08
2017-02-18
2017-07-02
2017-11-11
2021-01-01

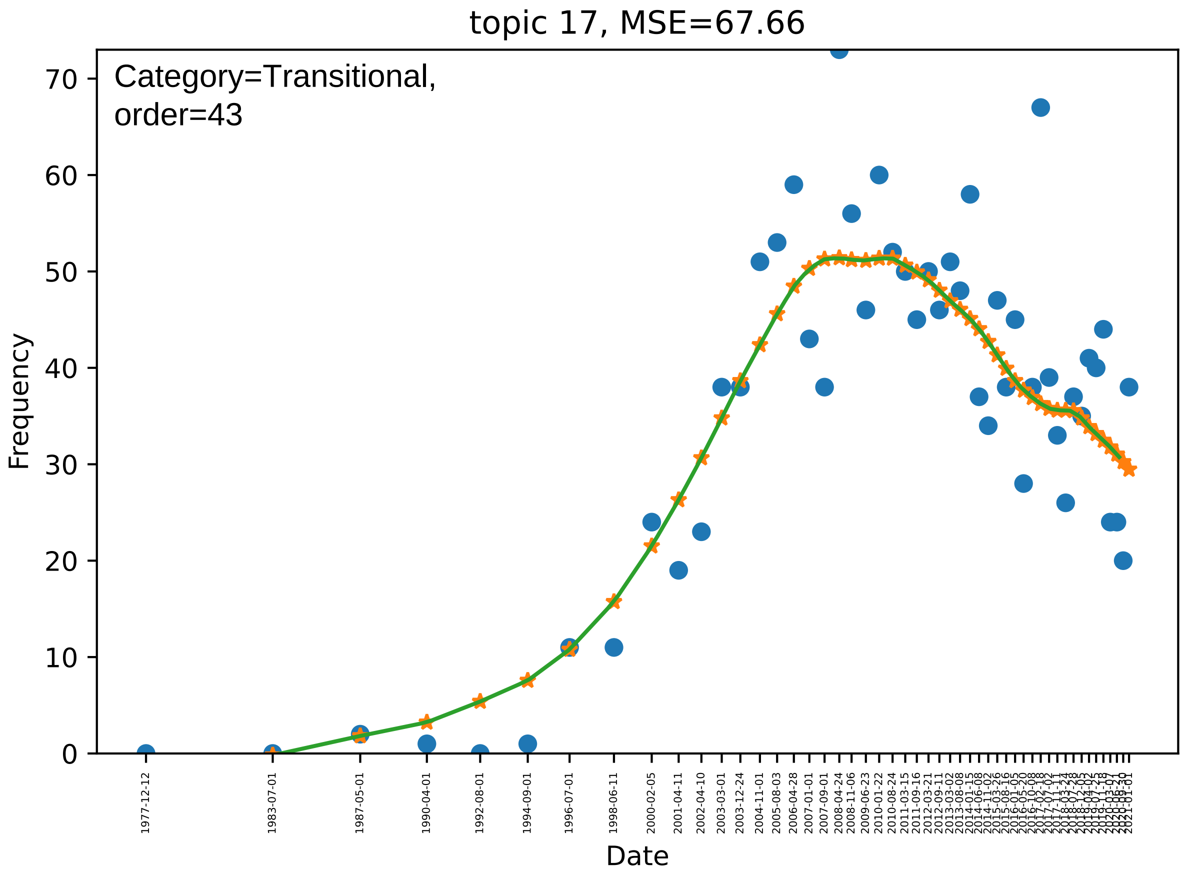
topic 17, MSE=67.66
Category=Transitional,
order=43
Frequency
70
60
50
40
30
20
10
0
1977-12-12
1983-07-01
1987-05-01
1990-04-01
1992-08-01
1994-09-01
1996-07-01
1998-06-11
2000-02-05
2001-04-11
2002-04-10
2003-03-01
2003-12-24
2004-11-01
2005-08-03
2006-04-28
2007-01-01
2007-09-01
2008-04-24
2008-11-06
2009-06-23
2010-01-22
2010-08-24
2011-03-15
2011-09-16
2012-03-21
2012-09-11
2013-03-02
2013-08-08
2014-01-15
2014-06-08
2014-11-02
2015-03-26
2015-08-16
2016-01-05
2016-05-20
2016-10-08
Date

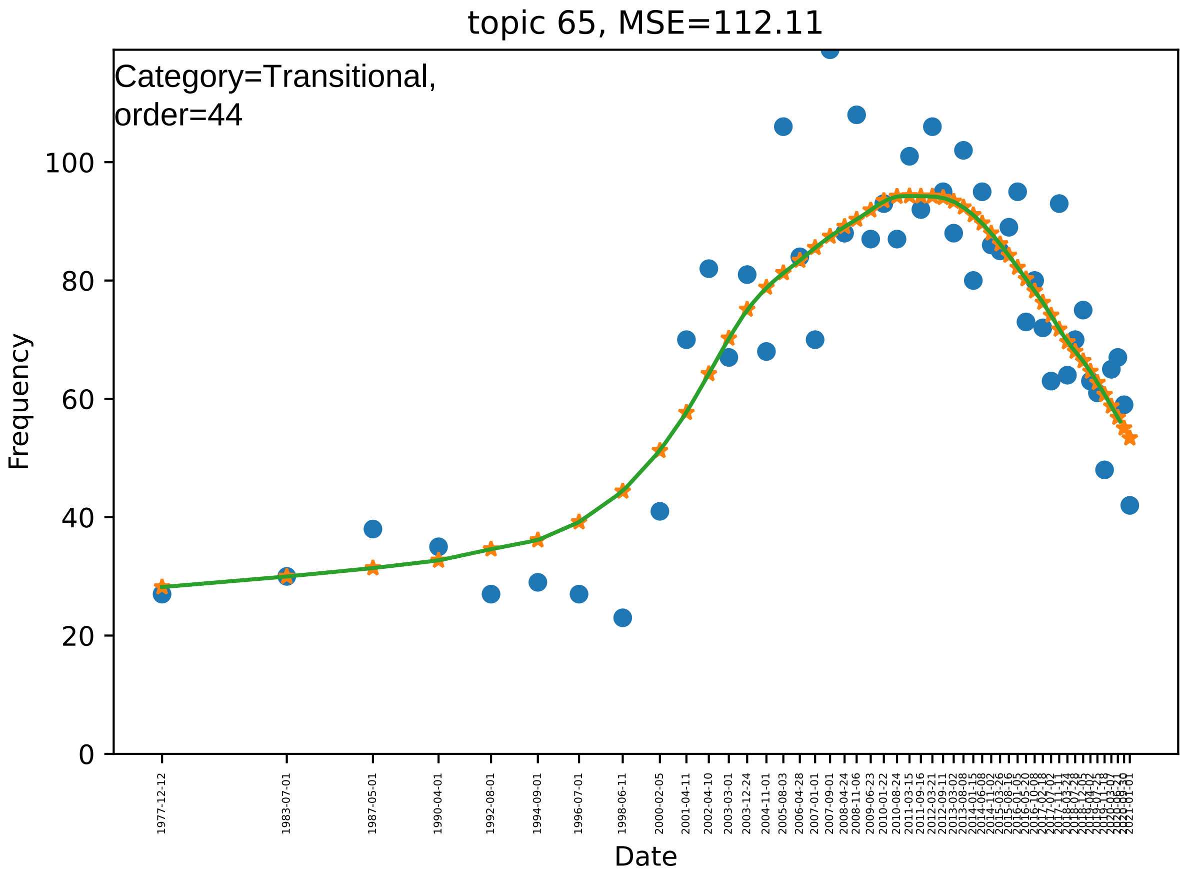

topic 65, MSE=112.11
Category=Transitional,
order=44
Frequency
0
20
40
60
80
100
Date
1977-12-12
1983-07-01
1987-05-01
1990-04-01
1992-08-01
1994-09-01
1996-07-01
1998-06-11
2000-02-05
2001-04-11
2002-04-10
2003-03-01
2003-12-24
2004-11-01
2005-08-03
2006-04-28
2007-01-01
2007-09-01
2008-04-24
2008-11-06
2009-06-23
2010-01-22
2010-08-24
2011-03-15
2011-09-16
2012-03-21
2012-09-11
2013-03-02
2013-08-08
2014-01-15
2014-06-08
2014-11-02
2015-03-26
2015-08-16
2016-01-05
2016-05-20
2016-10-08
2017-02-18
2017-07-02
2017-11-11
2021-01-01

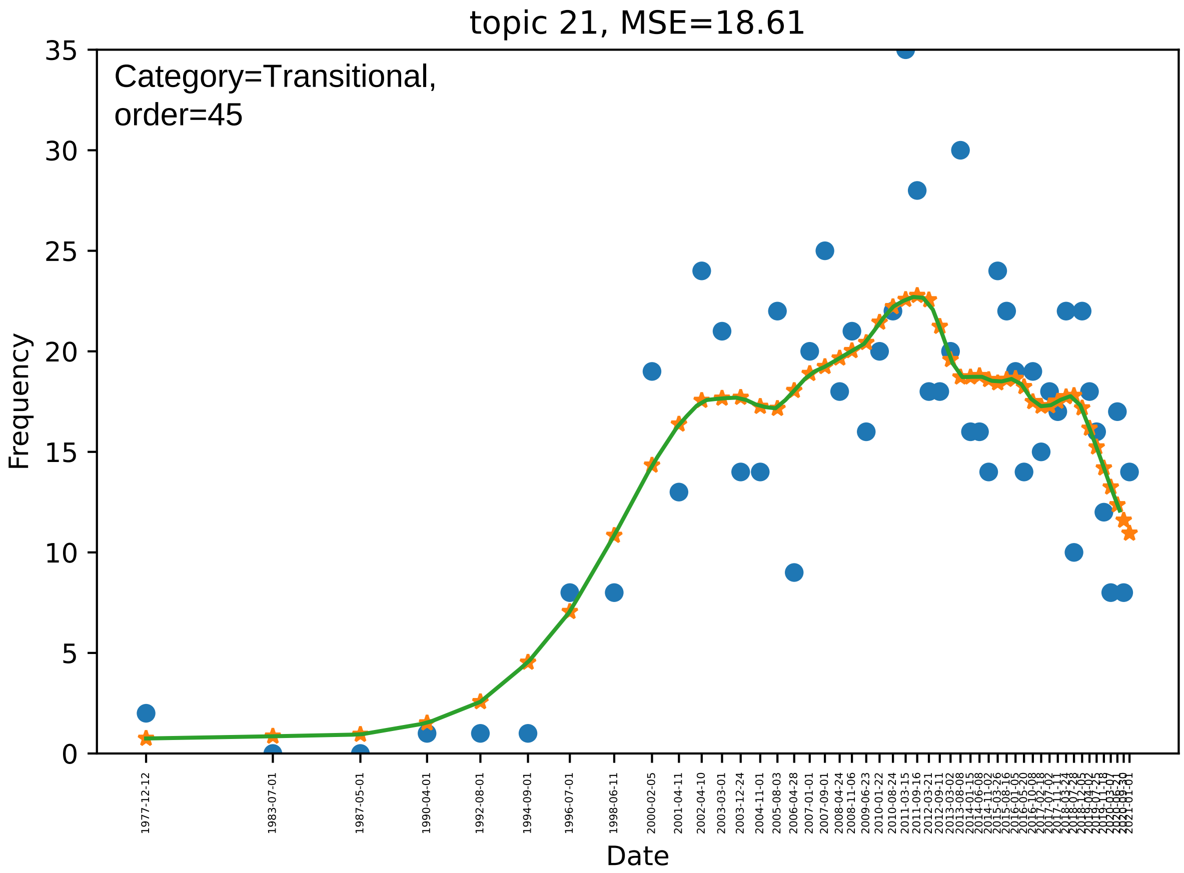
topic 21, MSE=18.61
Category=Transitional,
order=45
Frequency
Date
0
5
10
15
20
25
30
35
1977-12-12
1983-07-01
1987-05-01
1990-04-01
1992-08-01
1994-09-01
1996-07-01
1998-06-11
2000-02-05
2001-04-11
2002-04-10
2003-03-01
2003-12-24
2004-11-01
2005-08-03
2006-04-28
2007-01-01
2007-09-01
2008-04-24
2008-11-06
2009-06-23
2010-01-22
2010-08-24
2011-03-15
2011-09-16
2012-03-21
2012-09-11
2013-03-02
2013-08-08
2014-01-15
2014-06-08
2014-11-02

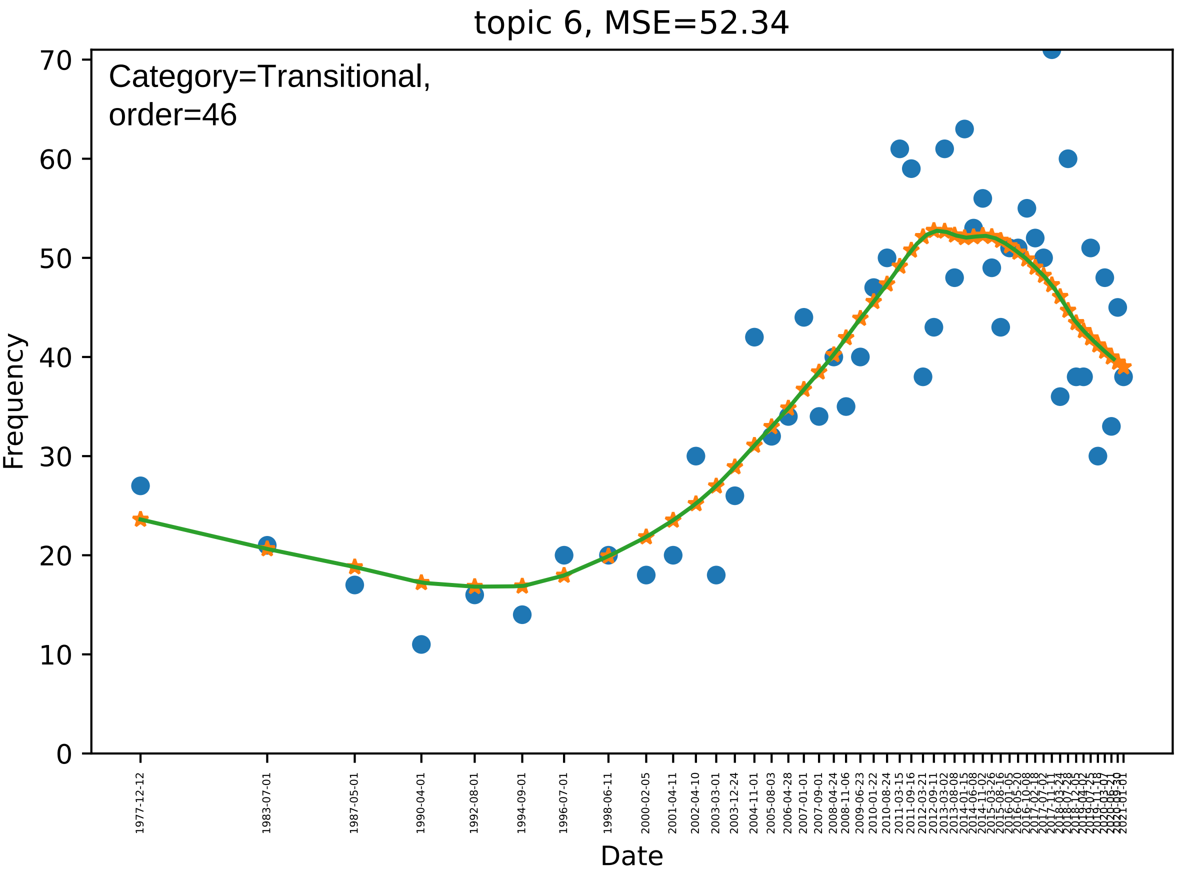
topic 6, MSE=52.34
Category=Transitional,
order=46
Frequency
Date
70
60
50
40
30
20
10
0
1977-12-12
1983-07-01
1987-05-01
1990-04-01
1992-08-01
1994-09-01
1996-07-01
1998-06-11
2000-02-05
2001-04-11
2002-04-10
2003-03-01
2003-12-24
2004-11-01
2005-08-03
2006-04-28
2007-01-01
2007-09-01
2008-04-24
2008-11-06
2009-06-23
2010-01-22
2010-08-24
2011-03-15
2011-09-16
2012-03-21
2012-09-11
2013-03-02
2013-08-08
2014-01-15
2014-06-08
2014-11-02

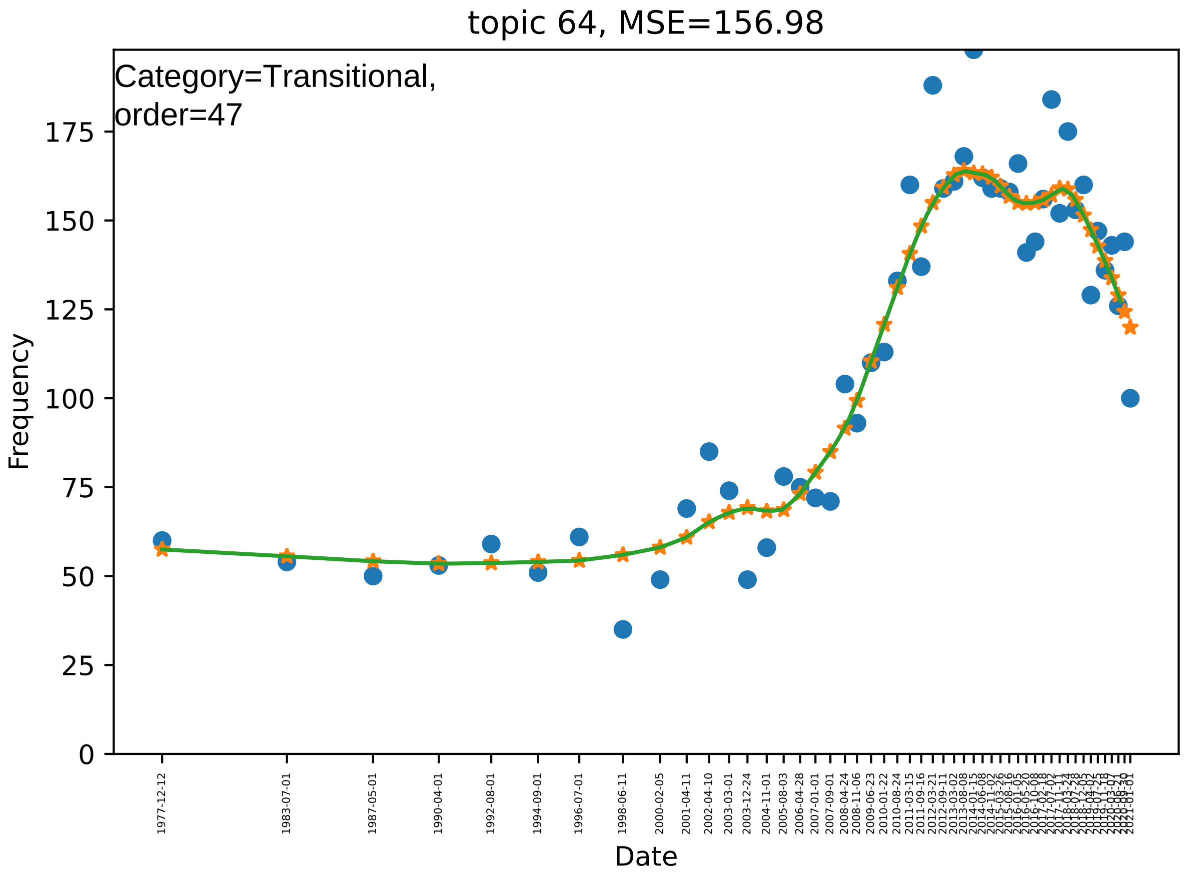

topic 64, MSE=156.98
Category=Transitional,
order=47
Frequency
0
25
50
75
100
125
150
175
Date
1977-12-12
1983-07-01
1987-05-01
1990-04-01
1992-08-01
1994-09-01
1996-07-01
1998-06-11
2000-02-05
2001-04-11
2002-04-10
2003-03-01
2003-12-24
2004-11-01
2005-08-03
2006-04-28
2007-01-01
2007-09-01
2008-04-24
2008-11-06
2009-06-23
2010-01-22
2010-08-24
2011-03-15
2011-09-16
2012-03-21
2012-09-11
2013-03-02
2013-08-08
2014-01-15
2014-06-08
2014-11-02
2015-03-26
2015-08-16
2016-01-05
2016-05-20
2016-10-08

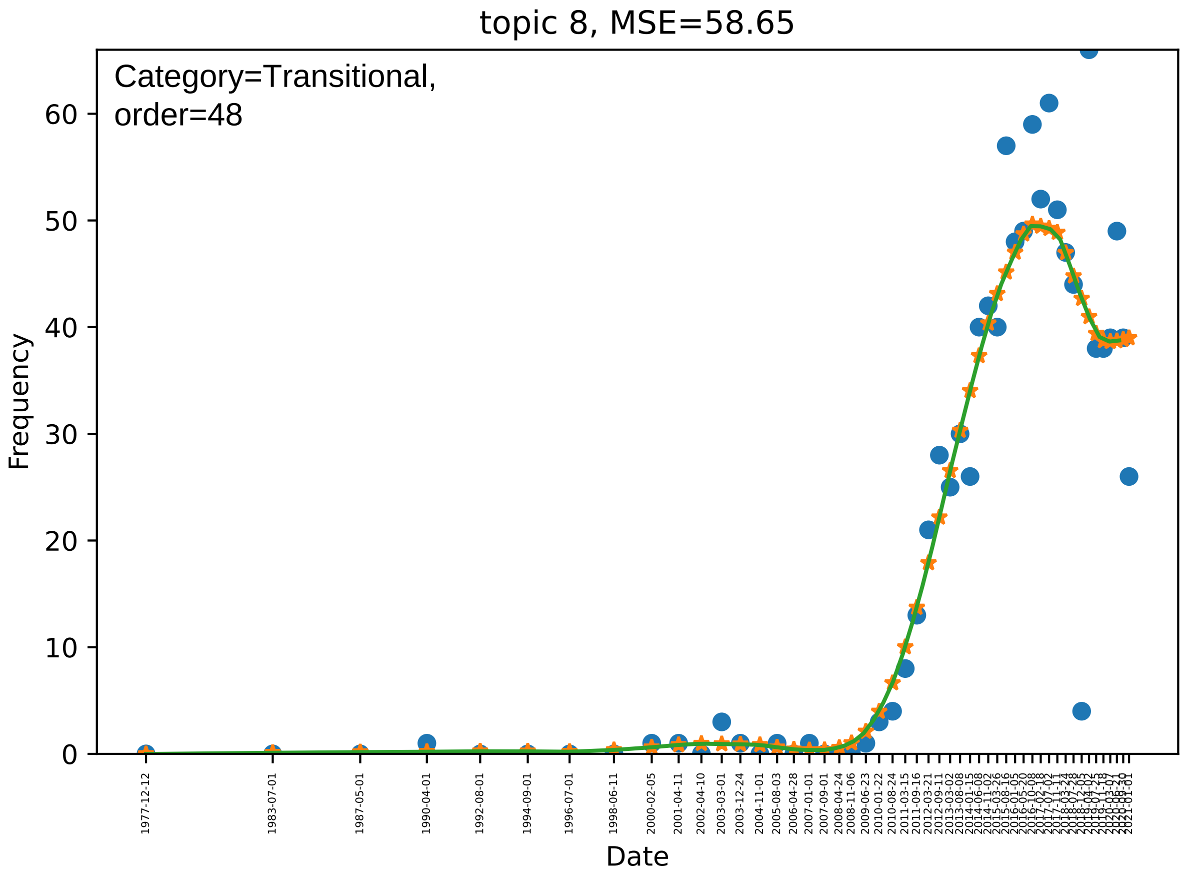

topic 8, MSE=58.65
Category=Transitional,
order=48
Frequency
0
10
20
30
40
50
60
Date
1977-12-12
1983-07-01
1987-05-01
1990-04-01
1992-08-01
1994-09-01
1996-07-01
1998-06-11
2000-02-05
2001-04-11
2002-04-10
2003-03-01
2003-12-24
2004-11-01
2005-08-03
2006-04-28
2007-01-01
2007-09-01
2008-04-24
2008-11-06
2009-06-23
2010-01-22
2010-08-24
2011-03-15
2011-09-16
2012-03-21
2012-09-11
2013-03-02
2013-08-08
2014-01-15
2014-06-08
2014-11-02
2015-03-26
2015-08-16
2016-01-05
2016-05-20
2016-10-08
2017-02-18
2017-07-02
2017-11-11
2018-03-24

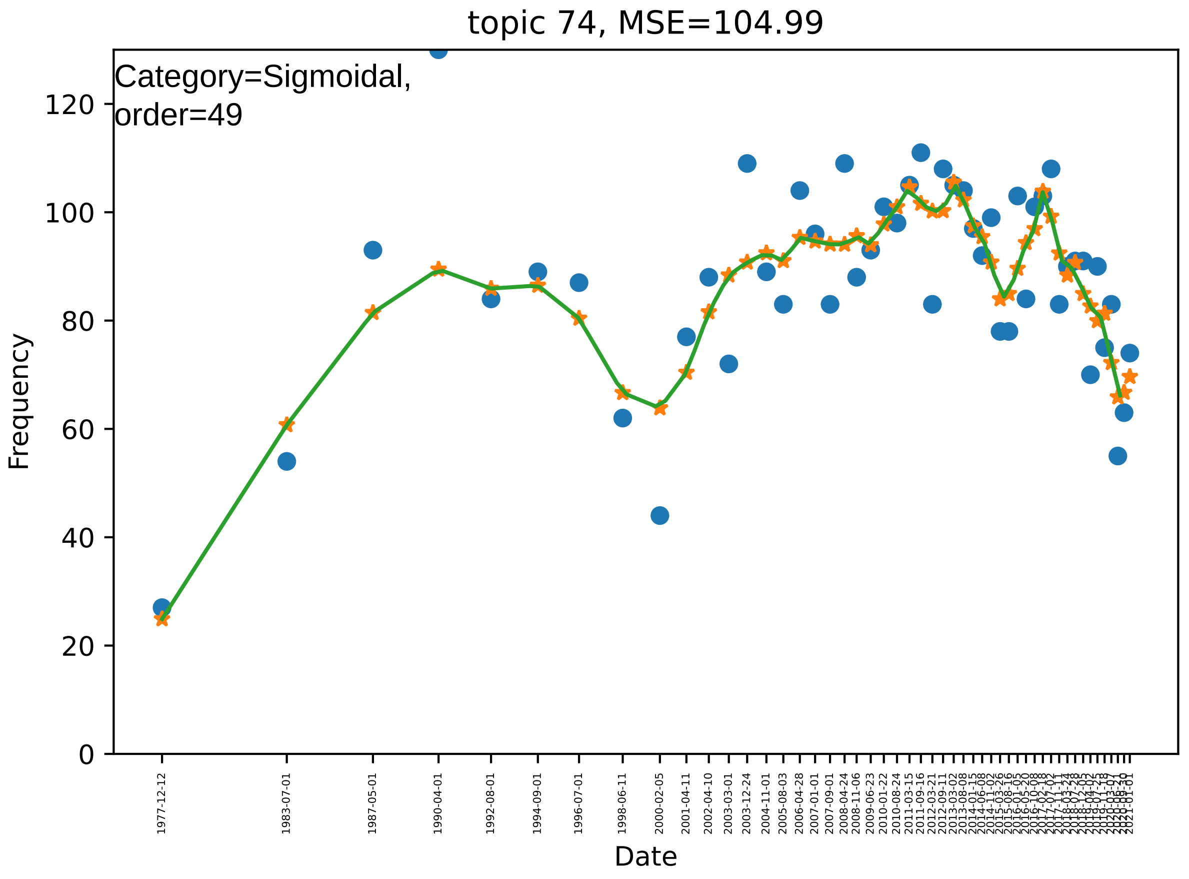

topic 74, MSE=104.99
Category=Sigmoidal,
order=49
Frequency
0
20
40
60
80
100
120
Date
1977-12-12
1983-07-01
1987-05-01
1990-04-01
1992-08-01
1994-09-01
1996-07-01
1998-06-11
2000-02-05
2001-04-11
2002-04-10
2003-03-01
2003-12-24
2004-11-01
2005-08-03
2006-04-28
2007-01-01
2007-09-01
2008-04-24
2008-11-06
2009-06-23
2010-01-22
2010-08-24
2011-03-15
2011-09-16
2012-03-21
2012-09-11
2013-03-02
2013-08-08
2014-01-15
2014-06-08
2014-11-02
2015-03-26
2015-08-16
2016-01-05
2016-05-20
2016-10-08
2017-02-18
2017-07-02
2021-01-31

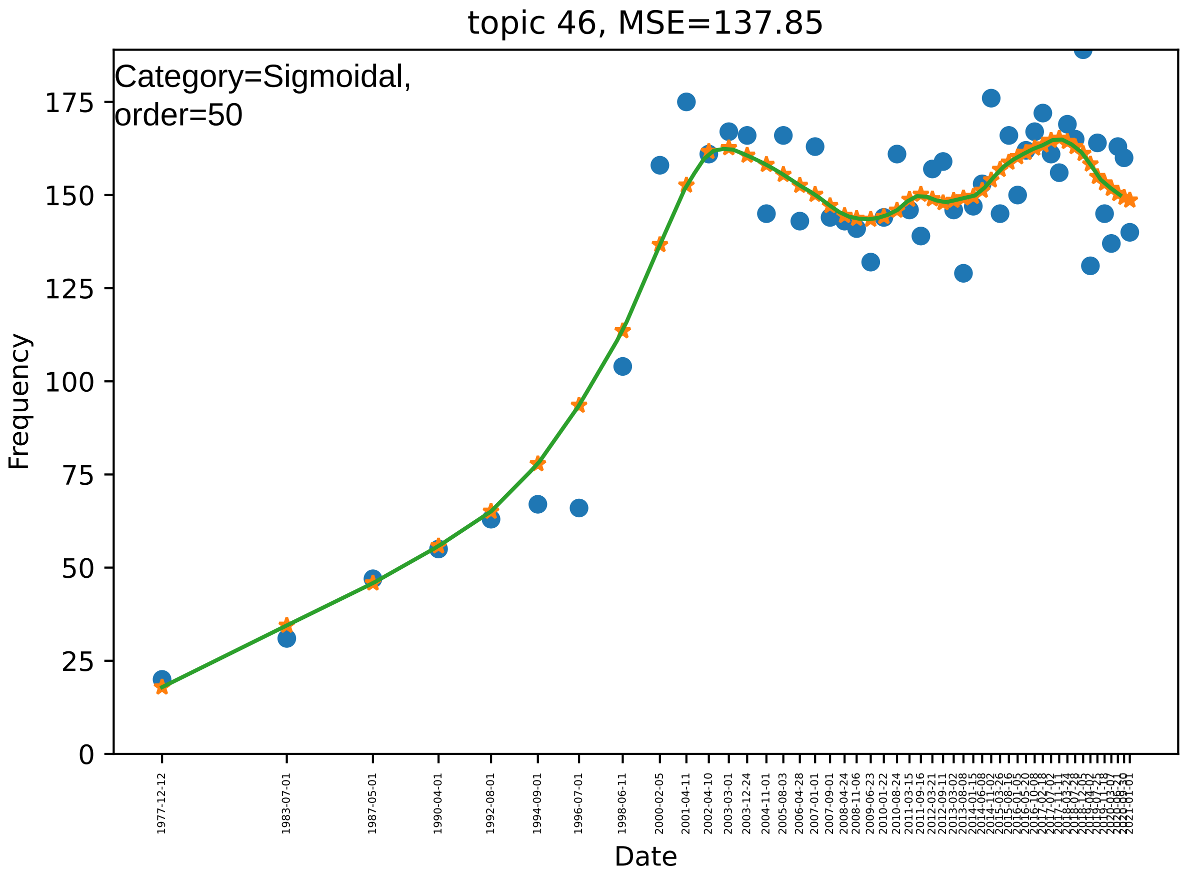
topic 46, MSE=137.85
Category=Sigmoidal,
order=50
Frequency
Date
0
25
50
75
100
125
150
175
1977-12-12
1983-07-01
1987-05-01
1990-04-01
1992-08-01
1994-09-01
1996-07-01
1998-06-11
2000-02-05
2001-04-11
2002-04-10
2003-03-01
2003-12-24
2004-11-01
2005-08-03
2006-04-28
2007-01-01
2007-09-01
2008-04-24
2008-11-06
2009-06-23
2010-01-22
2010-08-24
2011-03-15
2011-09-16
2012-03-21
2012-09-11
2013-03-02
2013-08-08
2014-01-15

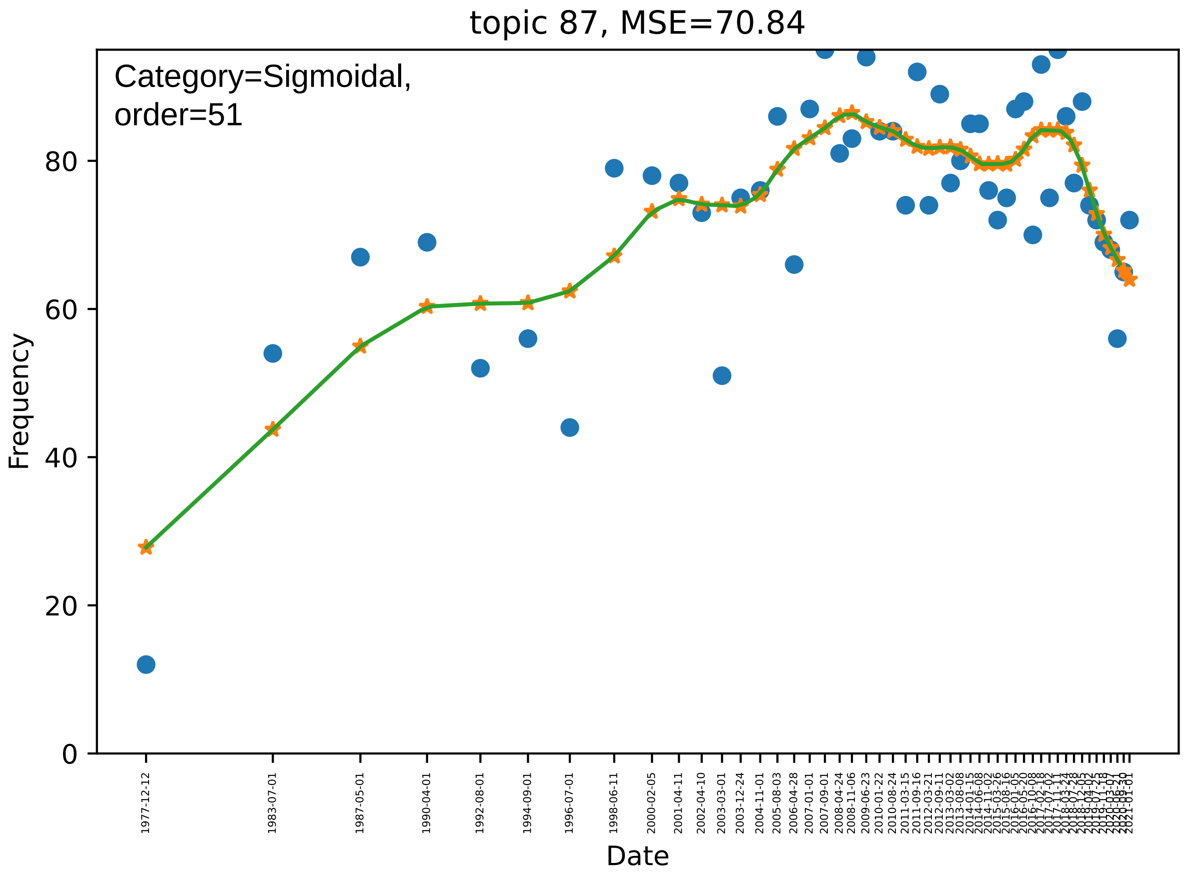
topic 87, MSE=70.84
Category=Sigmoidal,
order=51
Frequency
0
20
40
60
80
Date
1977-12-12
1983-07-01
1987-05-01
1990-04-01
1992-08-01
1994-09-01
1996-07-01
1998-06-11
2000-02-05
2001-04-11
2002-04-10
2003-03-01
2003-12-24
2004-11-01
2005-08-03
2006-04-28
2007-01-01
2007-09-01
2008-04-24
2008-11-06
2009-06-23
2010-01-22
2010-08-24
2011-03-15
2011-09-16
2012-03-21
2012-09-11
2013-03-02
2013-03-08

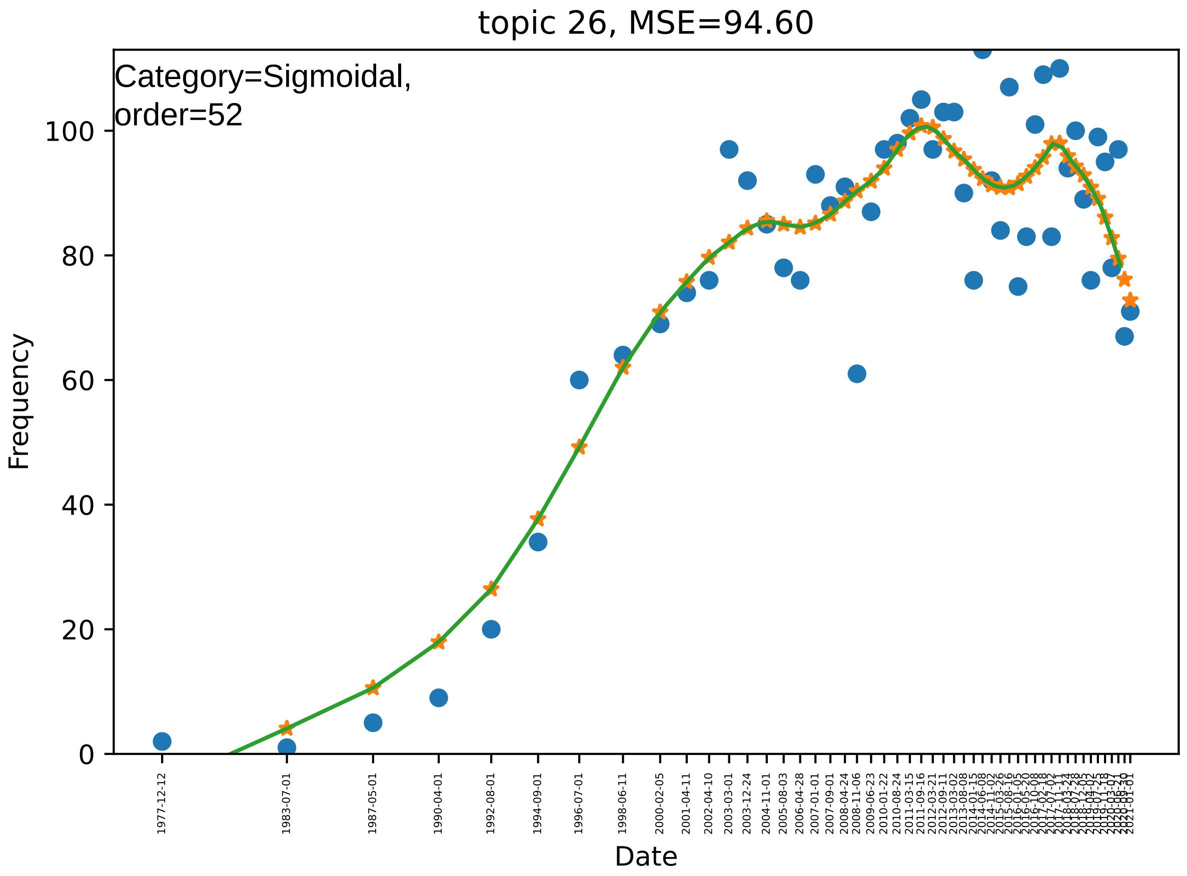

topic 26, MSE=94.60
Category=Sigmoidal,
order=52
Frequency
0
20
40
60
80
100
Date
1977-12-12
1983-07-01
1987-05-01
1990-04-01
1992-08-01
1994-09-01
1996-07-01
1998-06-11
2000-02-05
2001-04-11
2002-04-10
2003-03-01
2003-12-24
2004-11-01
2005-08-03
2006-04-28
2007-01-01
2007-09-01
2008-04-24
2008-11-06
2009-06-23
2010-01-22
2010-08-24
2011-03-15
2011-09-16
2012-03-21
2012-09-11
2013-03-02
2013-08-08
2014-01-15
2014-06-08
2014-11-02
2015-03-26
2015-08-16
2016-01-05
2016-05-20
2016-10-08
2017-02-18
2017-07-02
2021-01-01

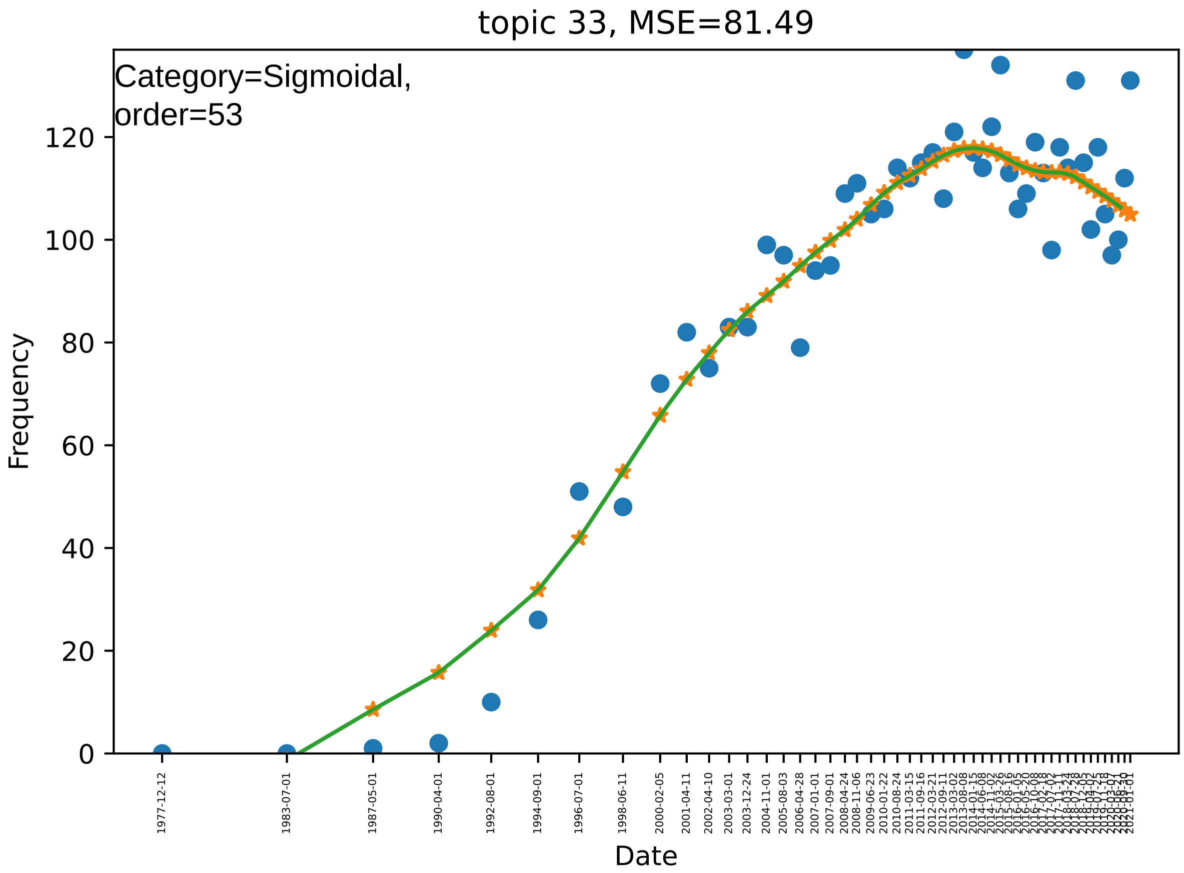

topic 33, MSE=81.49
Category=Sigmoidal,
order=53
Frequency
0
20
40
60
80
100
120
Date
1977-12-12
1983-07-01
1987-05-01
1990-04-01
1992-08-01
1994-09-01
1996-07-01
1998-06-11
2000-02-05
2001-04-11
2002-04-10
2003-03-01
2003-12-24
2004-11-01
2005-08-03
2006-04-28
2007-01-01
2007-09-01
2008-04-24
2008-11-06
2009-06-23
2010-01-22
2010-08-24
2011-03-15
2011-09-16
2012-03-21
2012-09-11
2013-03-02
2013-08-08
2014-01-15

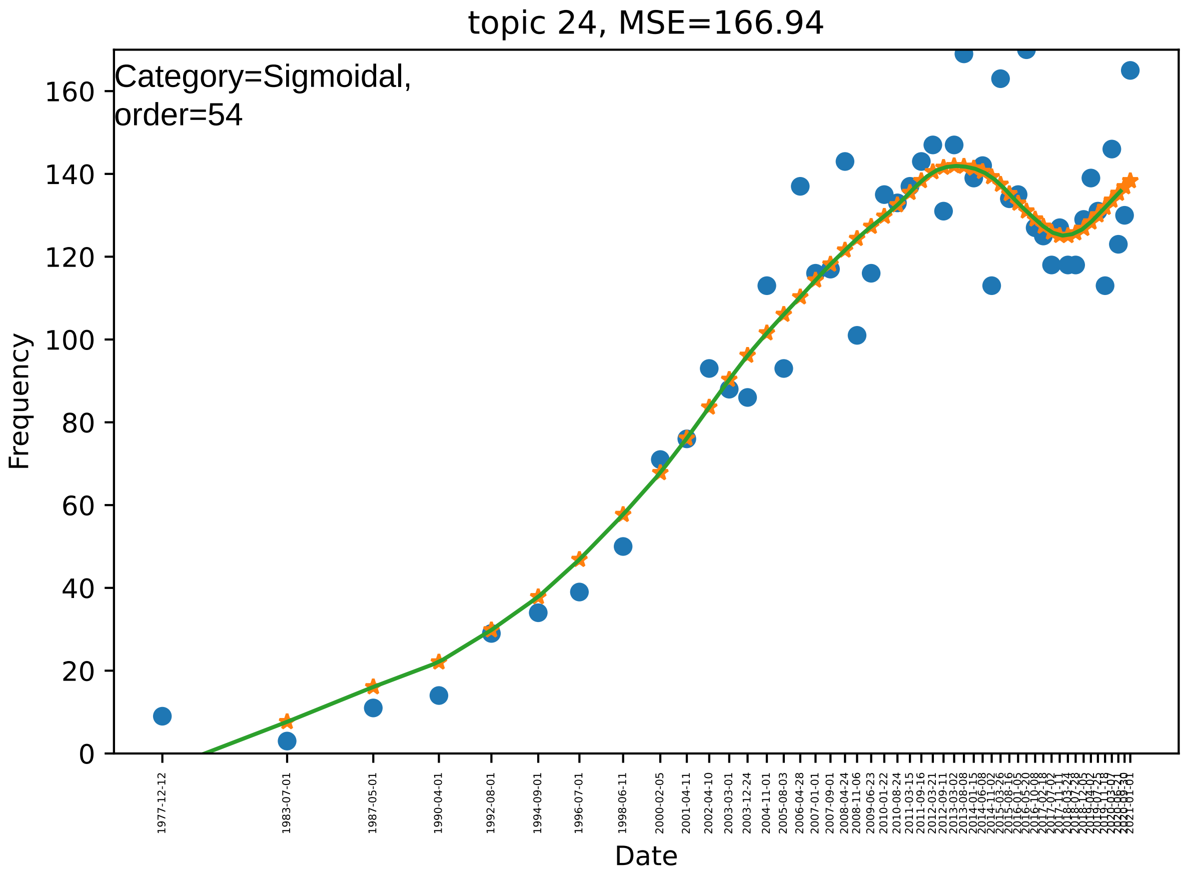
topic 24, MSE=166.94
Category=Sigmoidal,
order=54
Frequency
0
20
40
60
80
100
120
140
160
Date
1977-12-12
1983-07-01
1987-05-01
1990-04-01
1992-08-01
1994-09-01
1996-07-01
1998-06-11
2000-02-05
2001-04-11
2002-04-10
2003-03-01
2003-12-24
2004-11-01
2005-08-03
2006-04-28
2007-01-01
2007-09-01
2008-04-24
2008-11-06
2009-06-23
2010-01-22
2010-08-24
2011-03-15
2011-09-16
2012-03-21
2012-09-11

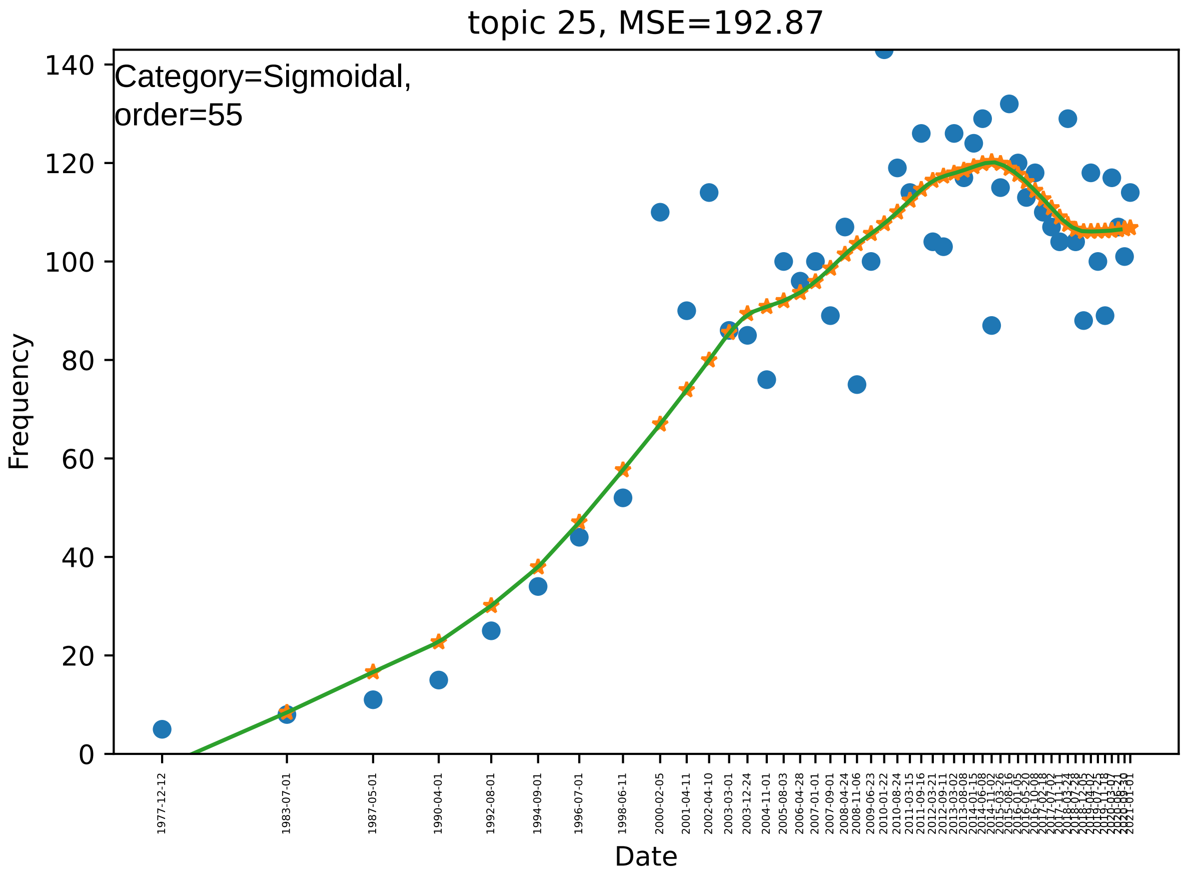

topic 25, MSE=192.87
Category=Sigmoidal,
order=55
Frequency
0
20
40
60
80
100
120
140
Date
1977-12-12
1983-07-01
1987-05-01
1990-04-01
1992-08-01
1994-09-01
1996-07-01
1998-06-11
2000-02-05
2001-04-11
2002-04-10
2003-03-01
2003-12-24
2004-11-01
2005-08-03
2006-04-28
2007-01-01
2007-09-01
2008-04-24
2008-11-06
2009-06-23
2010-01-22
2010-08-24
2011-03-15
2011-09-16
2012-03-21
2012-09-11
2013-03-02
2013-08-08
2014-01-15
2014-06-08
2014-11-02
2015-03-26
2015-08-16
2016-01-05
2016-05-20
2016-10-08
2017-02-18
2017-07-02
2017-11-11
2021-01-01

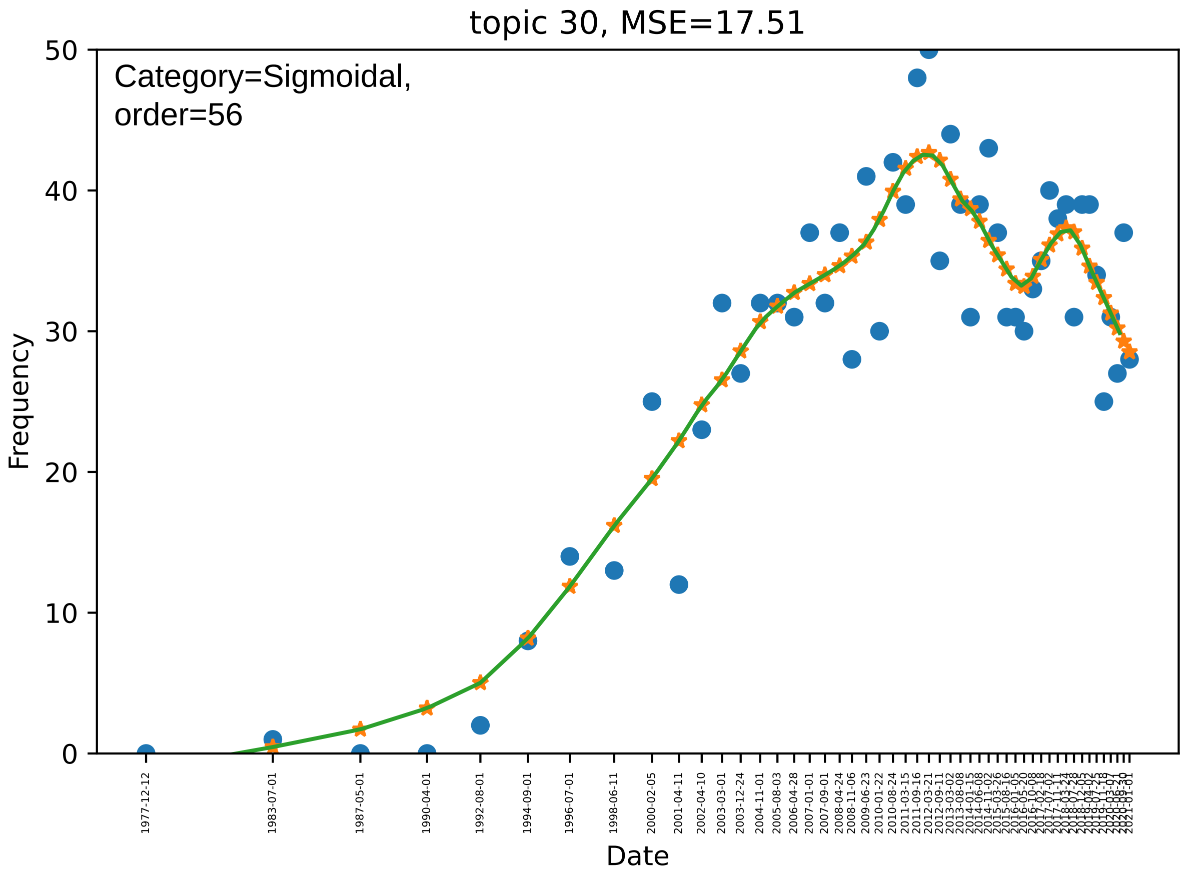
topic 30, MSE=17.51
Category=Sigmoidal,
order=56
Frequency
Date
0
10
20
30
40
50

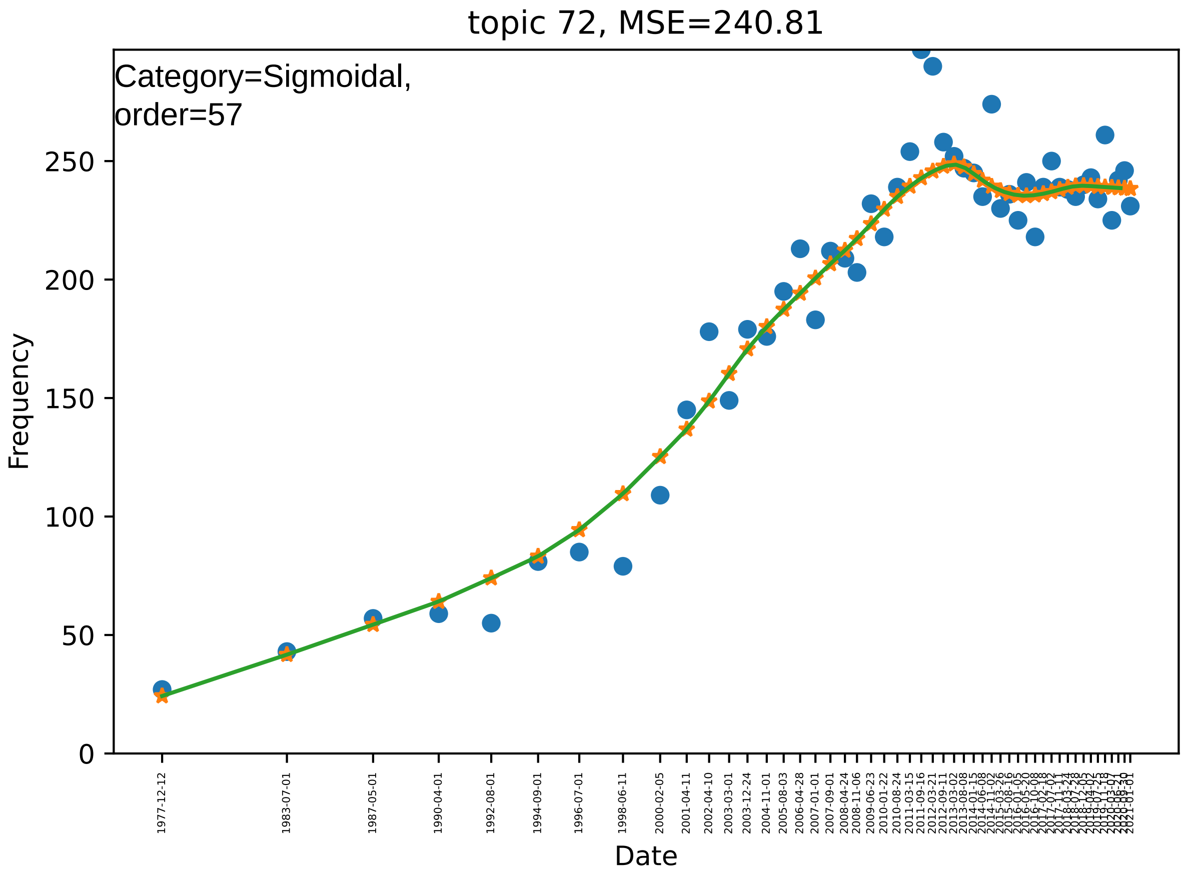
topic 72, MSE=240.81
Category=Sigmoidal,
order=57
Frequency
0
50
100
150
200
250
Date
1977-12-12
1983-07-01
1987-05-01
1990-04-01
1992-08-01
1994-09-01
1996-07-01
1998-06-11
2000-02-05
2001-04-11
2002-04-10
2003-03-01
2003-12-24
2004-11-01
2005-08-03
2006-04-28
2007-01-01
2007-09-01
2008-04-24
2008-11-06
2009-06-23
2010-01-22
2010-08-24
2011-03-15
2011-09-16
2012-03-21
2012-09-11
2013-03-02
2013-08-08

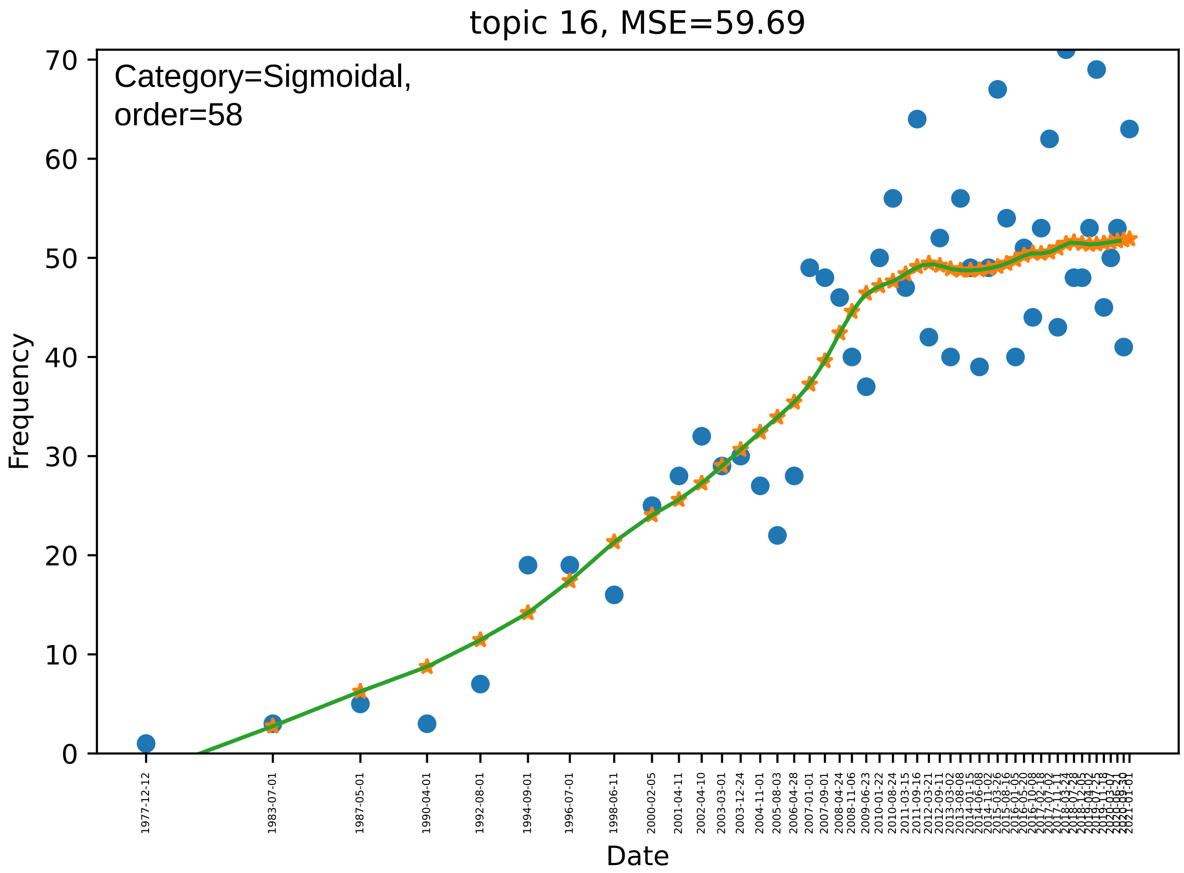

topic 16, MSE=59.69
Category=Sigmoidal,
order=58
Frequency
Date
0
10
20
30
40
50
60
70
1977-12-12
1983-07-01
1987-05-01
1990-04-01
1992-08-01
1994-09-01
1996-07-01
1998-06-11
2000-02-05
2001-04-11
2002-04-10
2003-03-01
2003-12-24
2004-11-01
2005-08-03
2006-04-28
2007-01-01
2007-09-01
2008-04-24
2008-11-06
2009-06-23
2010-01-22
2010-08-24
2011-03-15
2011-09-16
2012-03-21
2012-09-11
2013-03-02
2013-08-08
2014-01-15
2014-06-08
2014-11-02

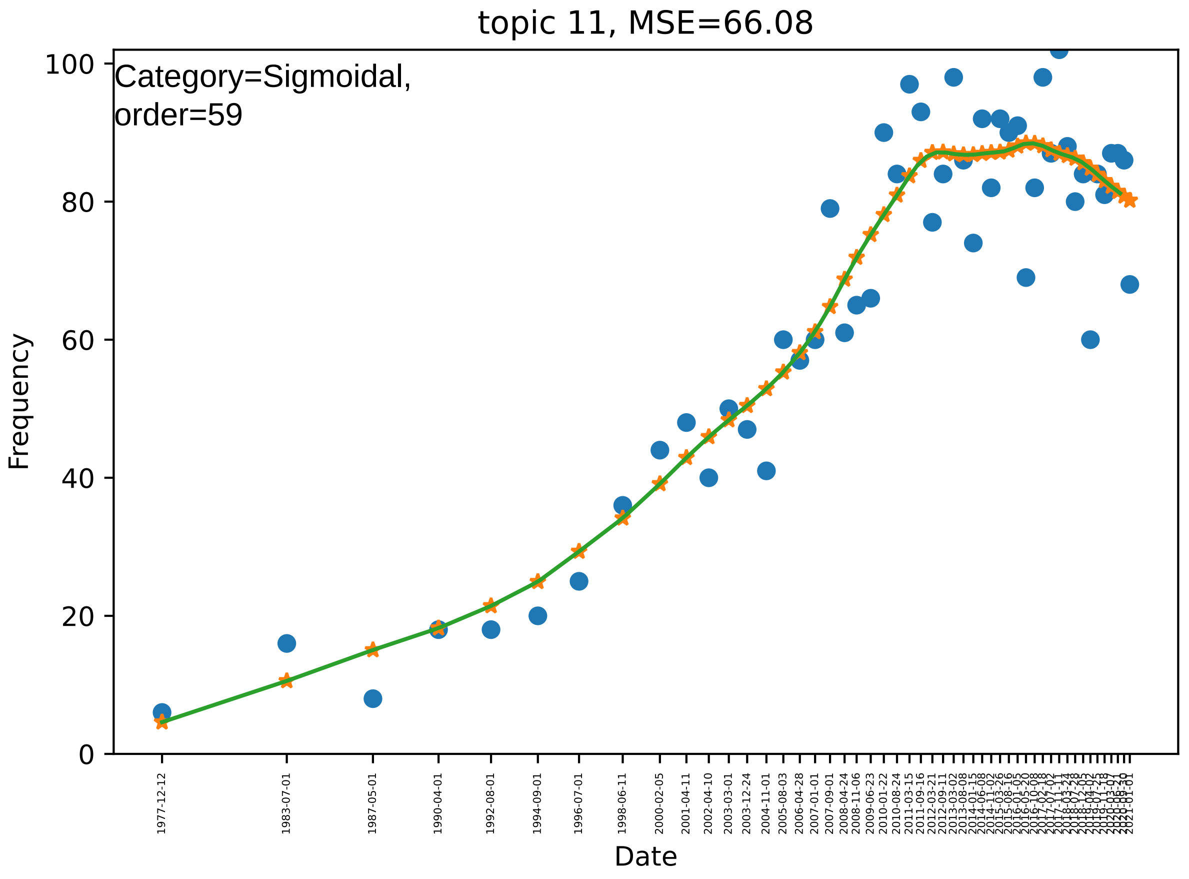
topic 11, MSE=66.08
Category=Sigmoidal,
order=59
Frequency
Date
0
20
40
60
80
100

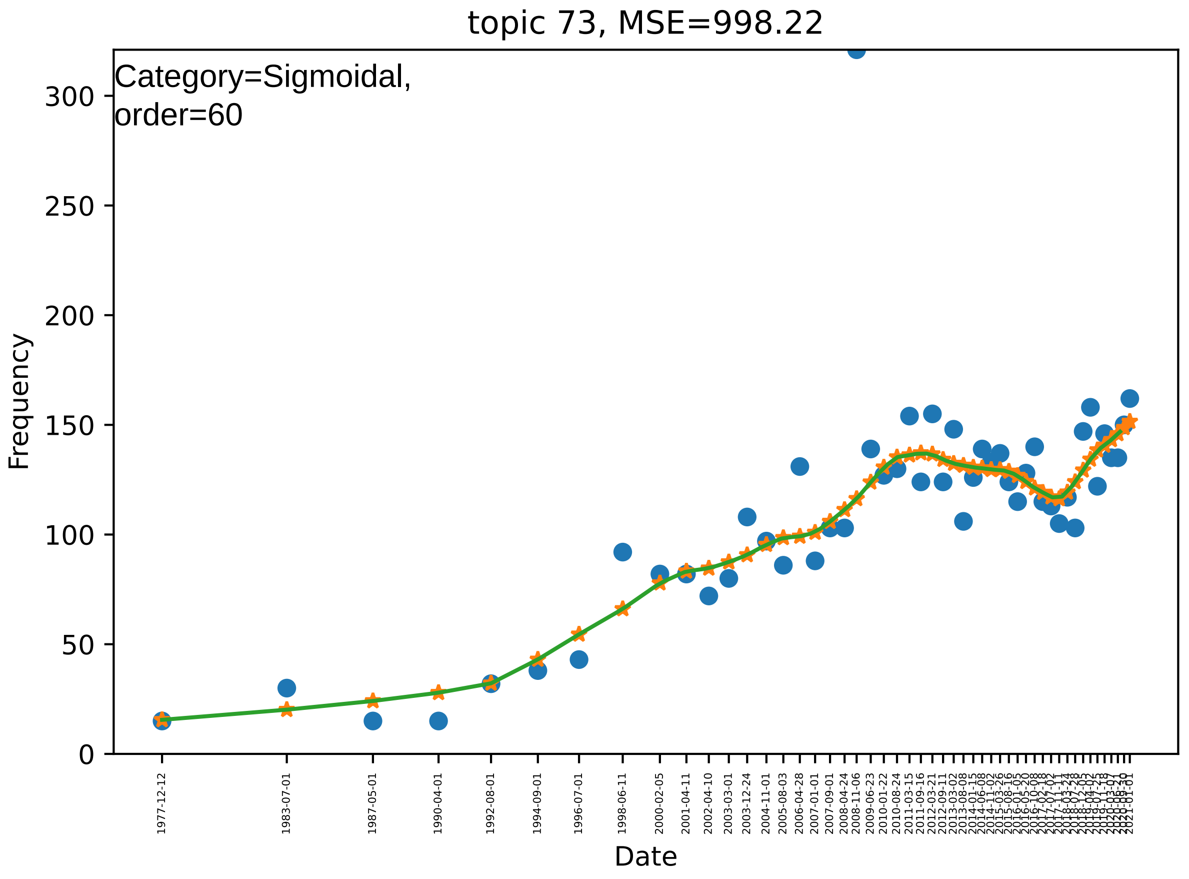
topic 73, MSE=998.22
Category=Sigmoidal,
order=60
Frequency
Date
0
50
100
150
200
250
300
1977-12-12
1983-07-01
1987-05-01
1990-04-01
1992-08-01
1994-09-01
1996-07-01
1998-06-11
2000-02-05
2001-04-11
2002-04-10
2003-03-01
2003-12-24
2004-11-01
2005-08-03
2006-04-28
2007-01-01
2007-09-01
2008-04-24
2008-11-06
2009-06-23
2010-01-22
2010-08-24
2011-03-15
2011-09-16
2012-03-21
2012-09-11
2013-03-02
2013-08-08
2014-01-15
2014-06-08
2014-11-02
2015-03-26
2015-08-16
2016-01-05
2016-05-20
2016-10-08

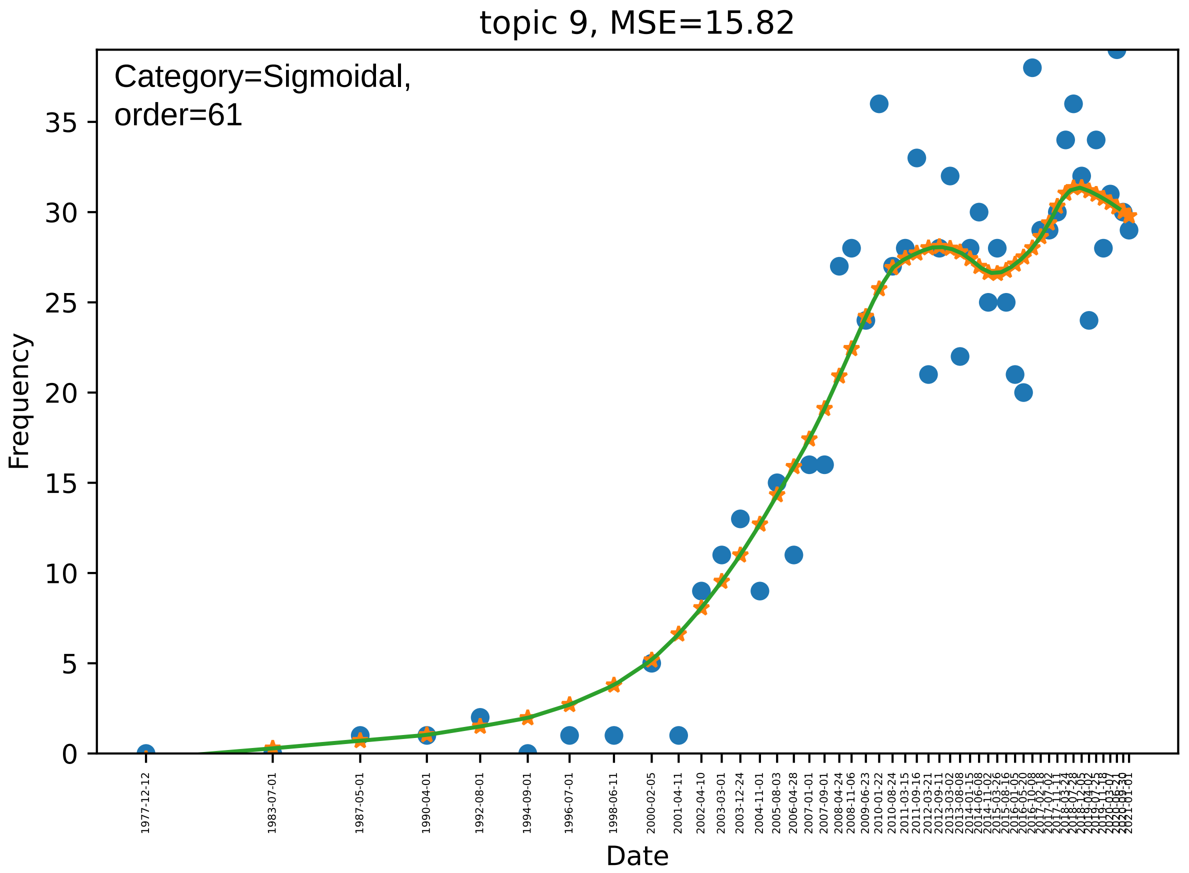

topic 9, MSE=15.82
Category=Sigmoidal,
order=61
Frequency
Date
0
5
10
15
20
25
30
35
1977-12-12
1983-07-01
1987-05-01
1990-04-01
1992-08-01
1994-09-01
1996-07-01
1998-06-11
2000-02-05
2001-04-11
2002-04-10
2003-03-01
2003-12-24
2004-11-01
2005-08-03
2006-04-28
2007-01-01
2007-09-01
2008-04-24
2008-11-06
2009-06-23
2010-01-22
2010-08-24
2011-03-15
2011-09-16
2012-03-21
2012-09-11
2013-03-02
2013-08-08
2014-01-15
2014-06-08
2014-11-02
2015-03-26
2015-08-16
2016-01-05
2016-05-20
2016-10-08
2017-02-18
2017-07-02
2017-11-11
2018-03-24

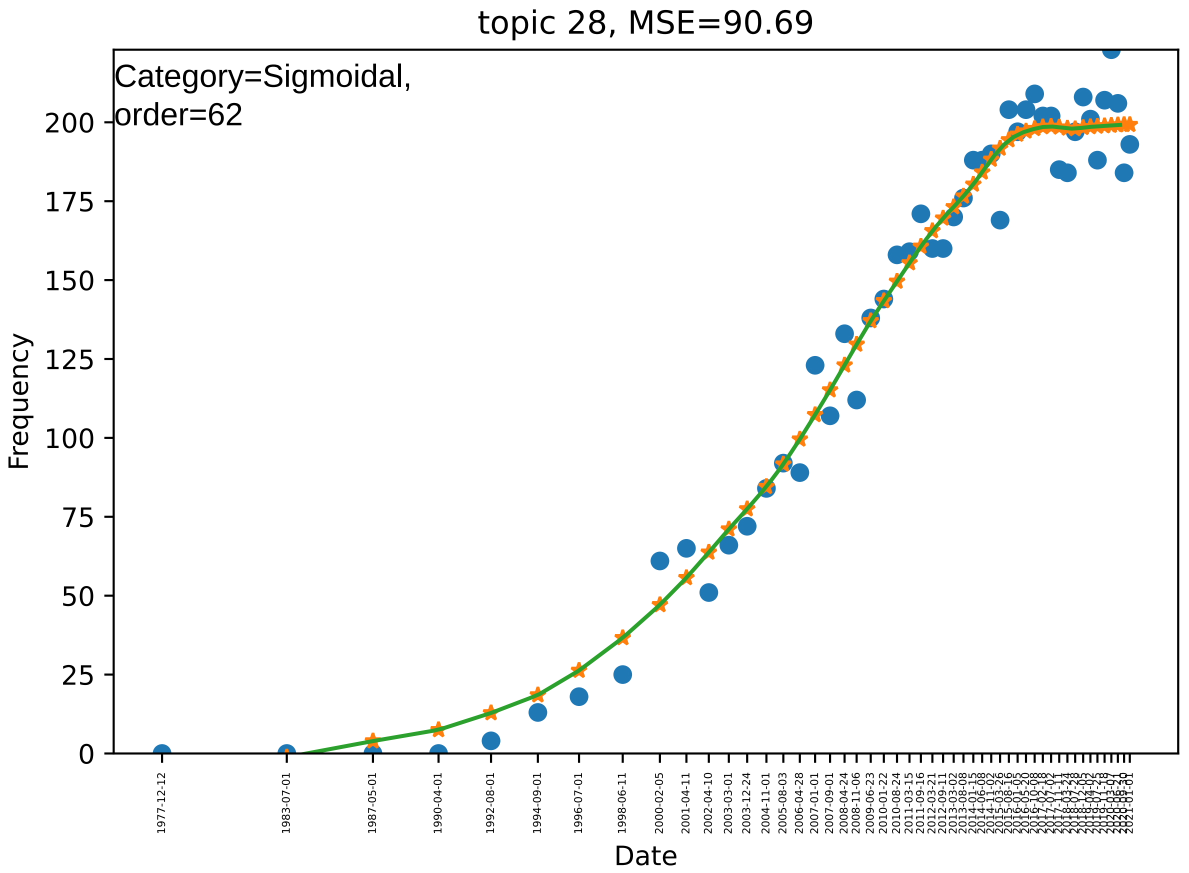
topic 28, MSE=90.69
Category=Sigmoidal,
order=62
Frequency
0
25
50
75
100
125
150
175
200
Date
1977-12-12
1983-07-01
1987-05-01
1990-04-01
1992-08-01
1994-09-01
1996-07-01
1998-06-11
2000-02-05
2001-04-11
2002-04-10
2003-03-01
2003-12-24
2004-11-01
2005-08-03
2006-04-28
2007-01-01
2007-09-01
2008-04-24
2008-11-06
2009-06-23
2010-01-22
2010-08-24
2011-03-15
2011-09-16
2012-03-21
2012-09-11
2013-03-02
2013-08-08
2014-01-15
2014-06-08
2014-11-02
2015-03-26
2015-08-16
2016-01-05
2016-05-20
2016-10-08
2017-02-18
2017-07-02
2021-01-01

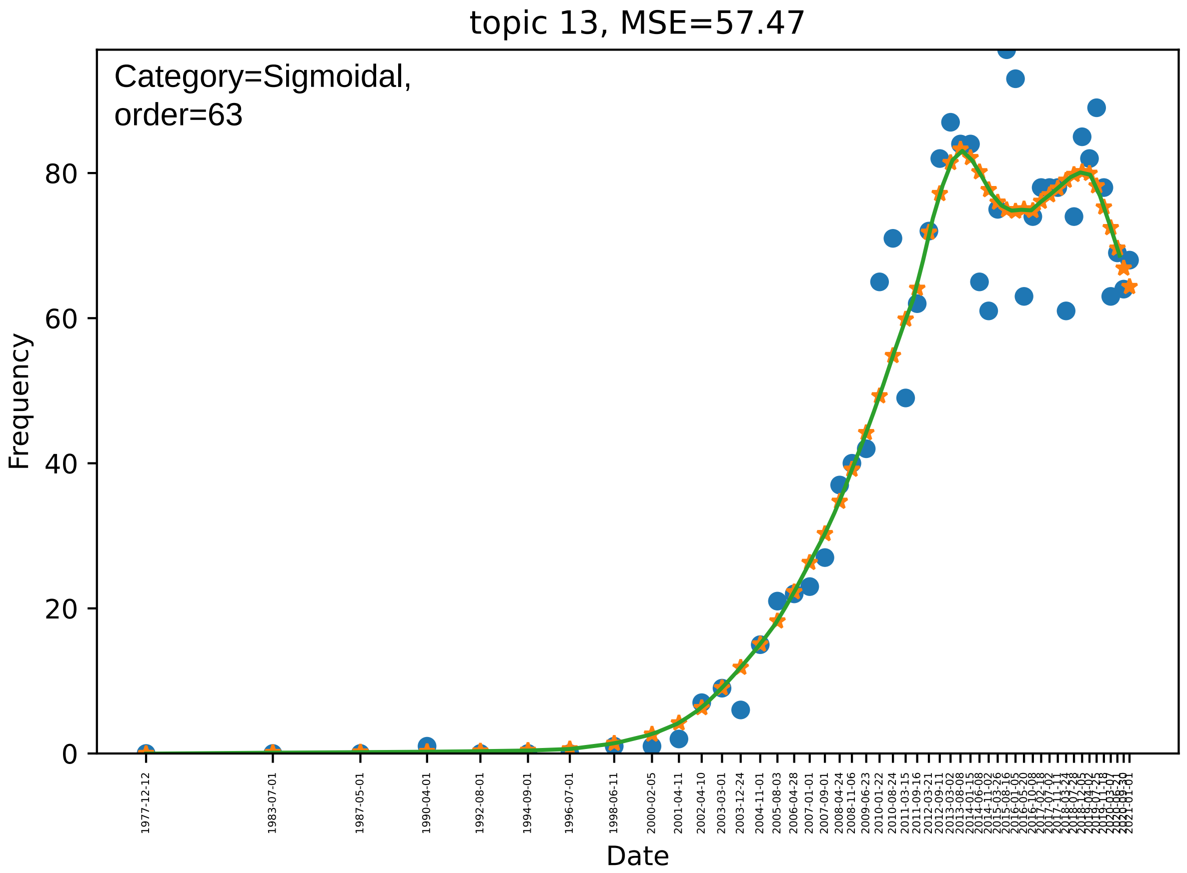

topic 13, MSE=57.47
Category=Sigmoidal,
order=63
Frequency
0
20
40
60
80
Date
1977-12-12
1983-07-01
1987-05-01
1990-04-01
1992-08-01
1994-09-01
1996-07-01
1998-06-11
2000-02-05
2001-04-11
2002-04-10
2003-03-01
2003-12-24
2004-11-01
2005-08-03
2006-04-28
2007-01-01
2007-09-01
2008-04-24
2008-11-06
2009-06-23
2010-01-22
2010-08-24
2011-03-15
2011-09-16
2012-03-21
2012-09-11
2013-03-02
2013-08-08
2014-01-15
2014-06-08
2014-11-02

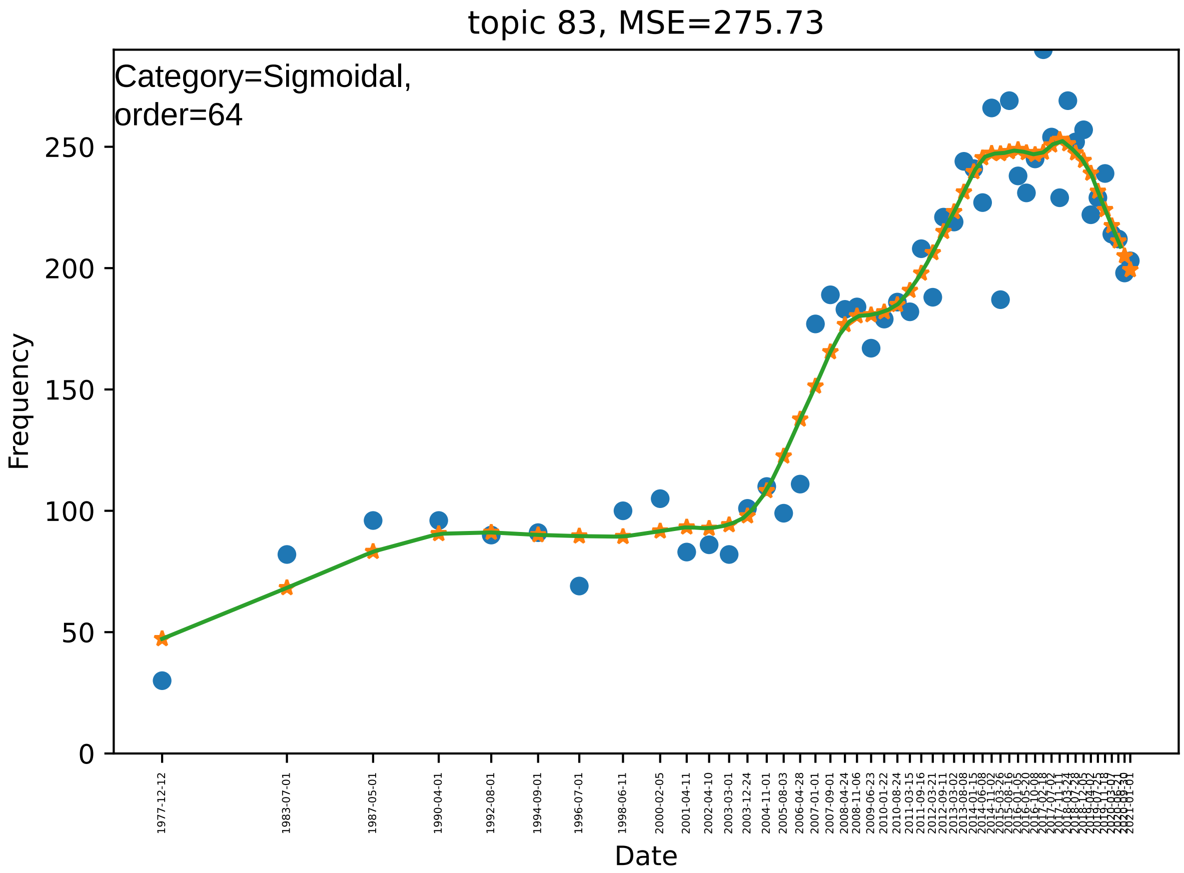

topic 83, MSE=275.73
Category=Sigmoidal,
order=64
Frequency
Date
0
50
100
150
200
250
1977-12-12
1983-07-01
1987-05-01
1990-04-01
1992-08-01
1994-09-01
1996-07-01
1998-06-11
2000-02-05
2001-04-11
2002-04-10
2003-03-01
2003-12-24
2004-11-01
2005-08-03
2006-04-28
2007-01-01
2007-09-01
2008-04-24
2008-11-06
2009-06-23
2010-01-22
2010-08-24
2011-03-15
2011-09-16
2012-03-21
2012-09-11
2013-03-02
2013-08-08
2014-01-15
2014-06-08
2014-11-02
2015-03-26
2015-08-16
2016-01-05
2016-05-20
2016-10-08
2017-02-18
2017-07-02
2017-11-11
2018-03-24
2018-07-28
2018-12-03
2019-04-05
2019-07-25
2019-11-18
2020-03-07
2021-01-01

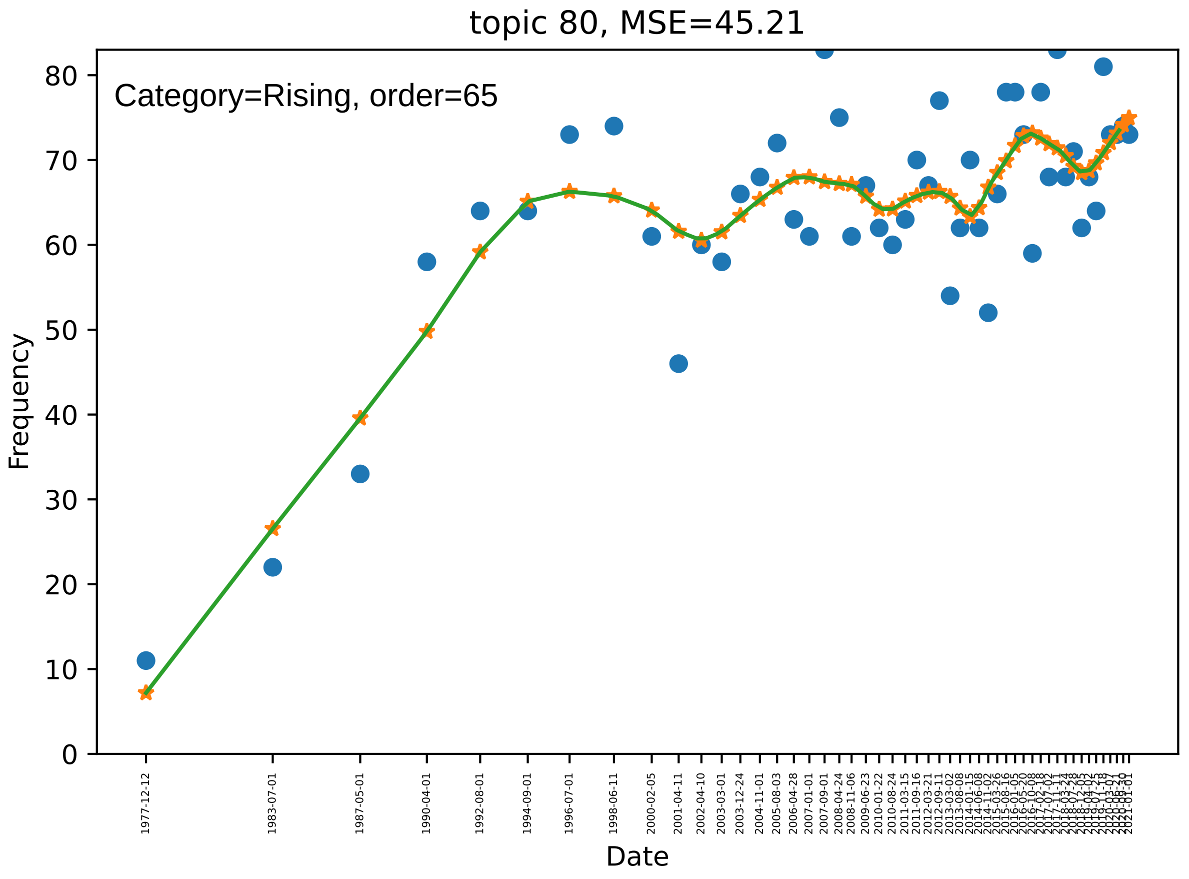
topic 80, MSE=45.21
Category=Rising, order=65
Frequency
Date
0
10
20
30
40
50
60
70
80
1977-12-12
1983-07-01
1987-05-01
1990-04-01
1992-08-01
1994-09-01
1996-07-01
1998-06-11
2000-02-05
2001-04-11
2002-04-10
2003-03-01
2003-12-24
2004-11-01
2005-08-03
2006-04-28
2007-01-01
2007-09-01
2008-04-24
2008-11-06
2009-06-23
2010-01-22
2010-08-24
2011-03-15
2011-09-16
2012-03-21
2012-09-11
2013-03-02
2013-08-08
2014-01-15
2014-06-08
2014-11-02

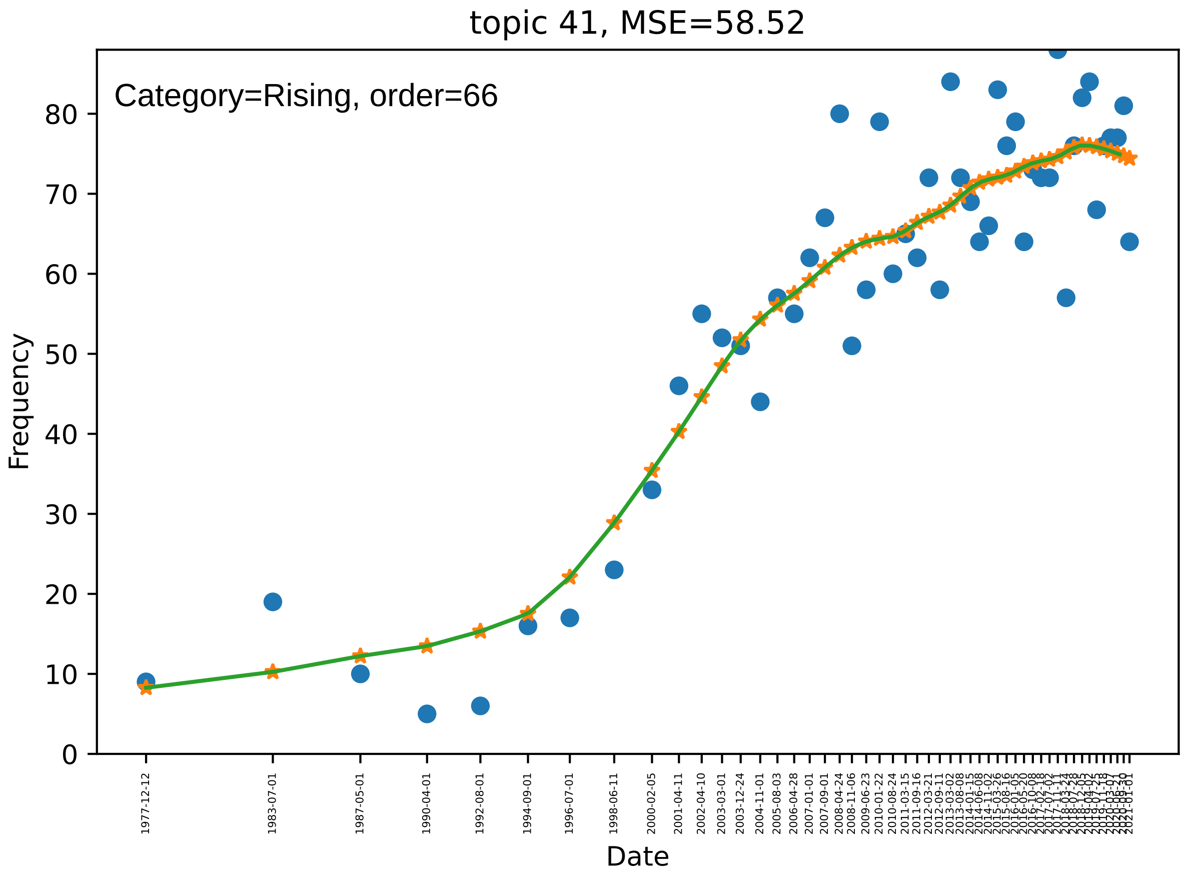

topic 41, MSE=58.52
Category=Rising, order=66
Frequency
Date
0
10
20
30
40
50
60
70
80
1977-12-12
1983-07-01
1987-05-01
1990-04-01
1992-08-01
1994-09-01
1996-07-01
1998-06-11
2000-02-05
2001-04-11
2002-04-10
2003-03-01
2003-12-24
2004-11-01
2005-08-03
2006-04-28
2007-01-01
2007-09-01
2008-04-24
2008-11-06
2009-06-23
2010-01-22
2010-08-24
2011-03-15
2011-09-16
2012-03-21
2012-09-11
2013-03-02
2013-08-08
2014-01-15
2014-06-08
2014-11-02

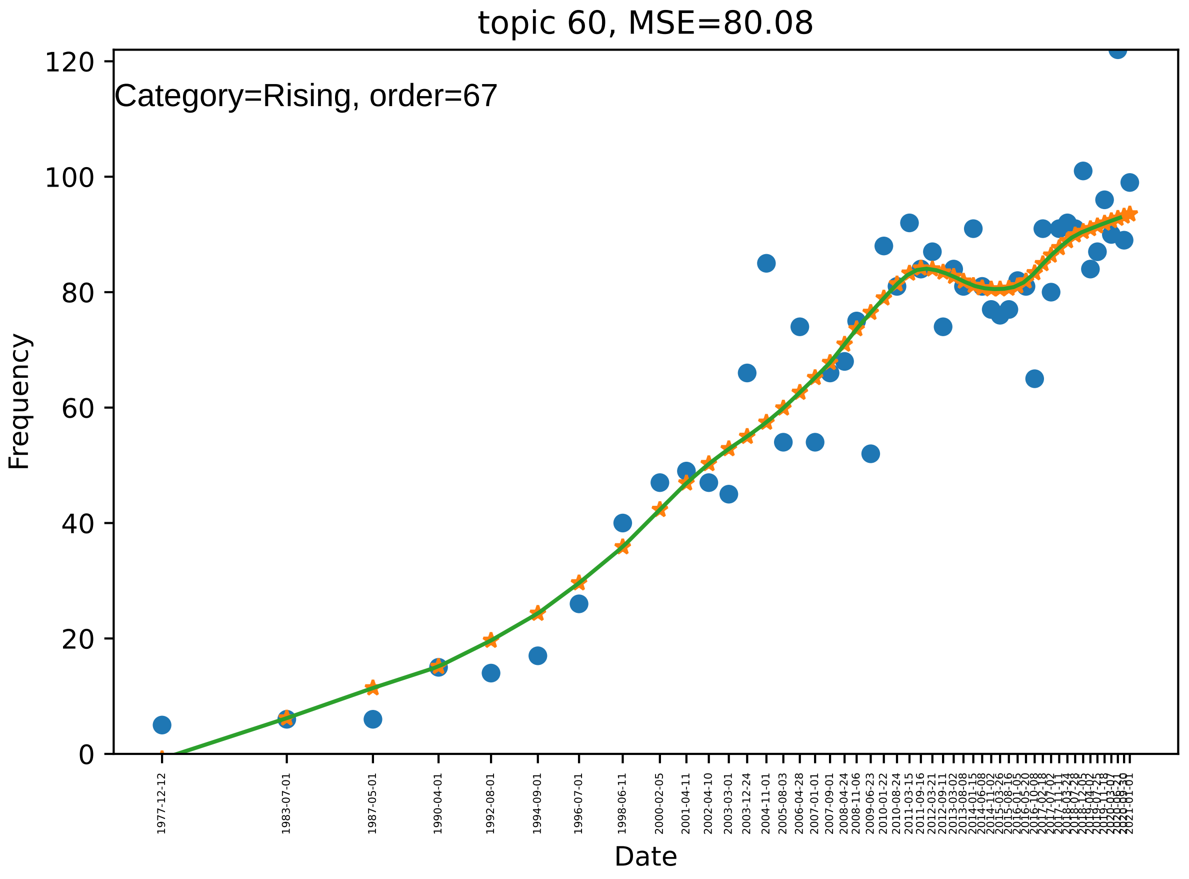

topic 60, MSE=80.08
Category=Rising, order=67
Frequency
Date
0
20
40
60
80
100
120
1977-12-12
1983-07-01
1987-05-01
1990-04-01
1992-08-01
1994-09-01
1996-07-01
1998-06-11
2000-02-05
2001-04-11
2002-04-10
2003-03-01
2003-12-24
2004-11-01
2005-08-03
2006-04-28
2007-01-01
2007-09-01
2008-04-24
2008-11-06
2009-06-23
2010-01-22
2010-08-24
2011-03-15
2011-09-16
2012-03-21
2012-09-11
2013-03-02
2013-08-08
2014-01-15

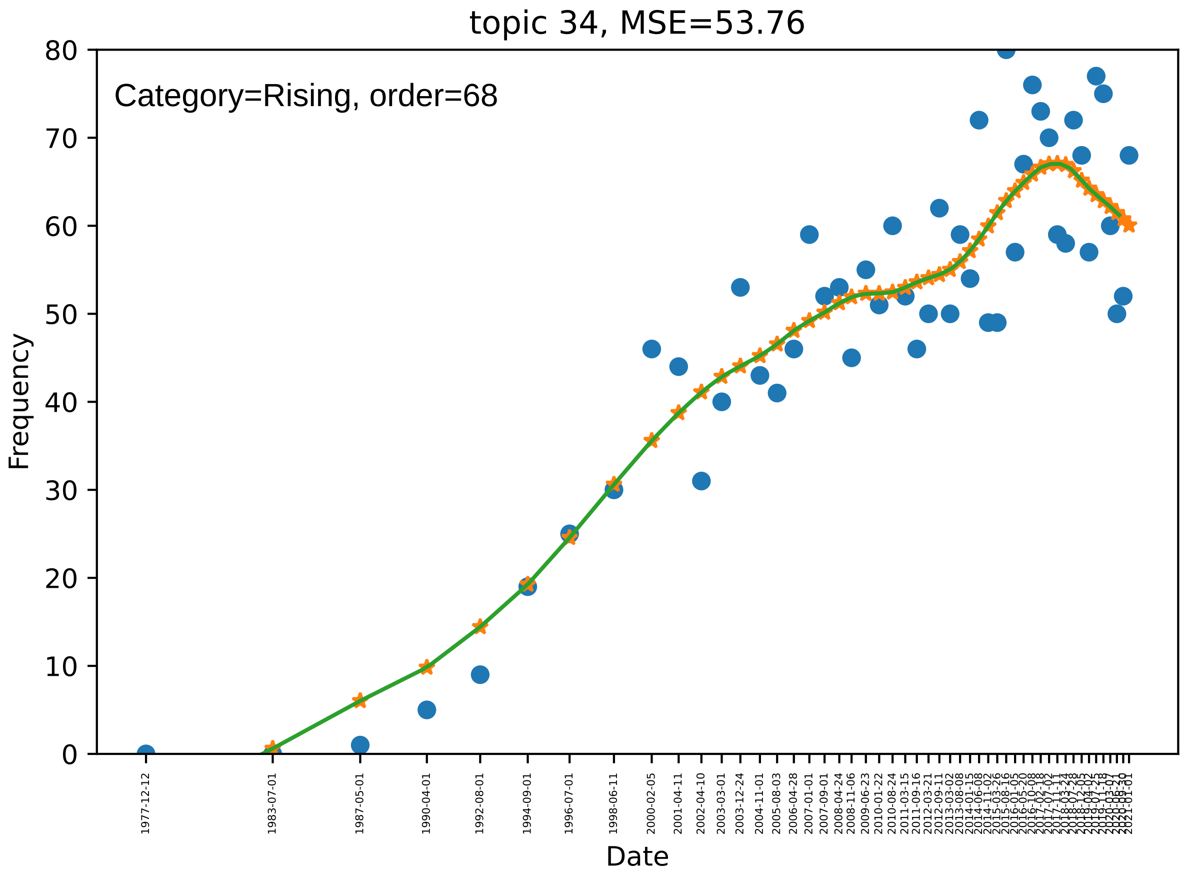
topic 34, MSE=53.76
Category=Rising, order=68
Frequency
Date
0
10
20
30
40
50
60
70
80

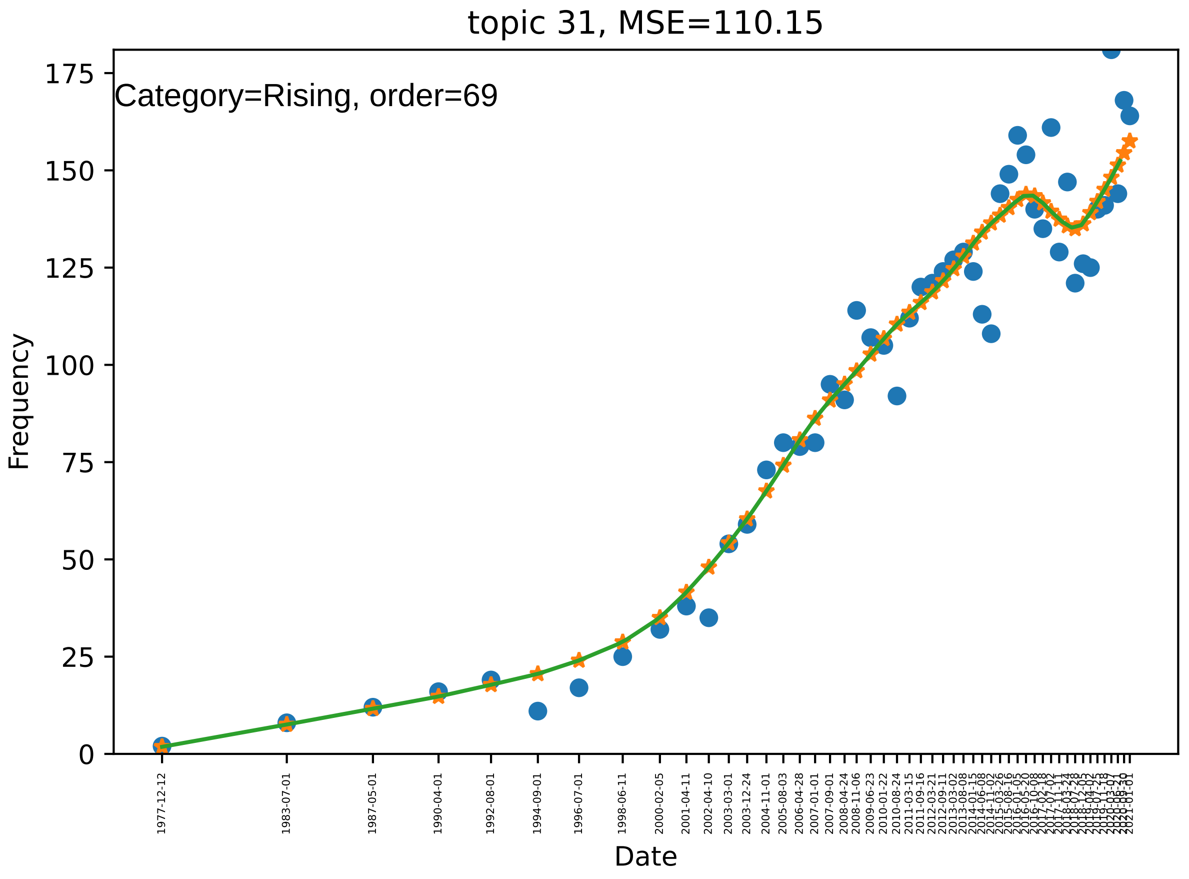
topic 31, MSE=110.15
Category=Rising, order=69
Frequency
Date
0
25
50
75
100
125
150
175

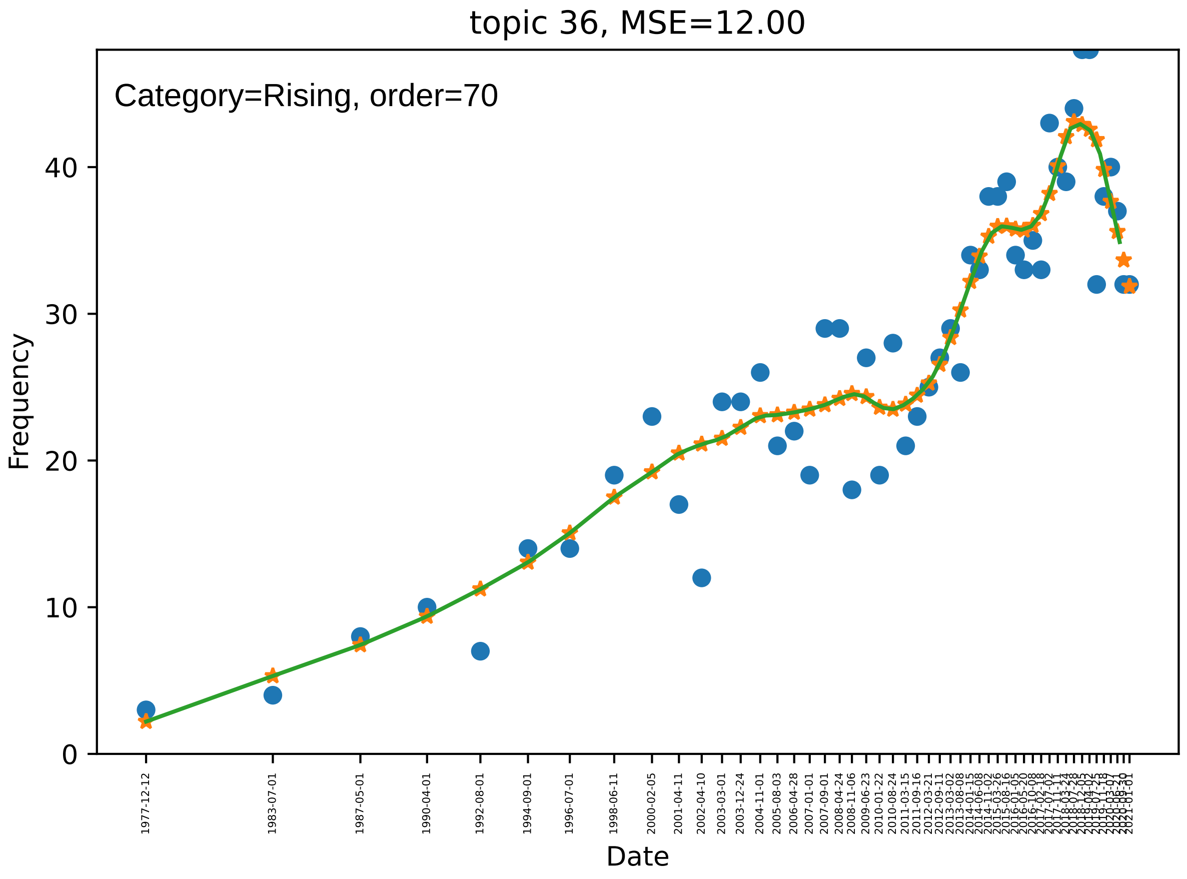
topic 36, MSE=12.00
Category=Rising, order=70
Frequency
0
10
20
30
40
Date
1977-12-12
1983-07-01
1987-05-01
1990-04-01
1992-08-01
1994-09-01
1996-07-01
1998-06-11
2000-02-05
2001-04-11
2002-04-10
2003-03-01
2003-12-24
2004-11-01
2005-08-03
2006-04-28
2007-01-01
2007-09-01
2008-04-24
2008-11-06
2009-06-23
2010-01-22
2010-08-24
2011-03-15
2011-09-16
2012-03-21
2012-09-11
2013-03-02
2013-08-08
2014-01-15
2014-06-08
2014-11-02
2015-03-26
2015-08-16
2016-01-05
2016-05-20
2016-10-08
2017-02-18
2017-07-02
2017-11-11
2018-03-24

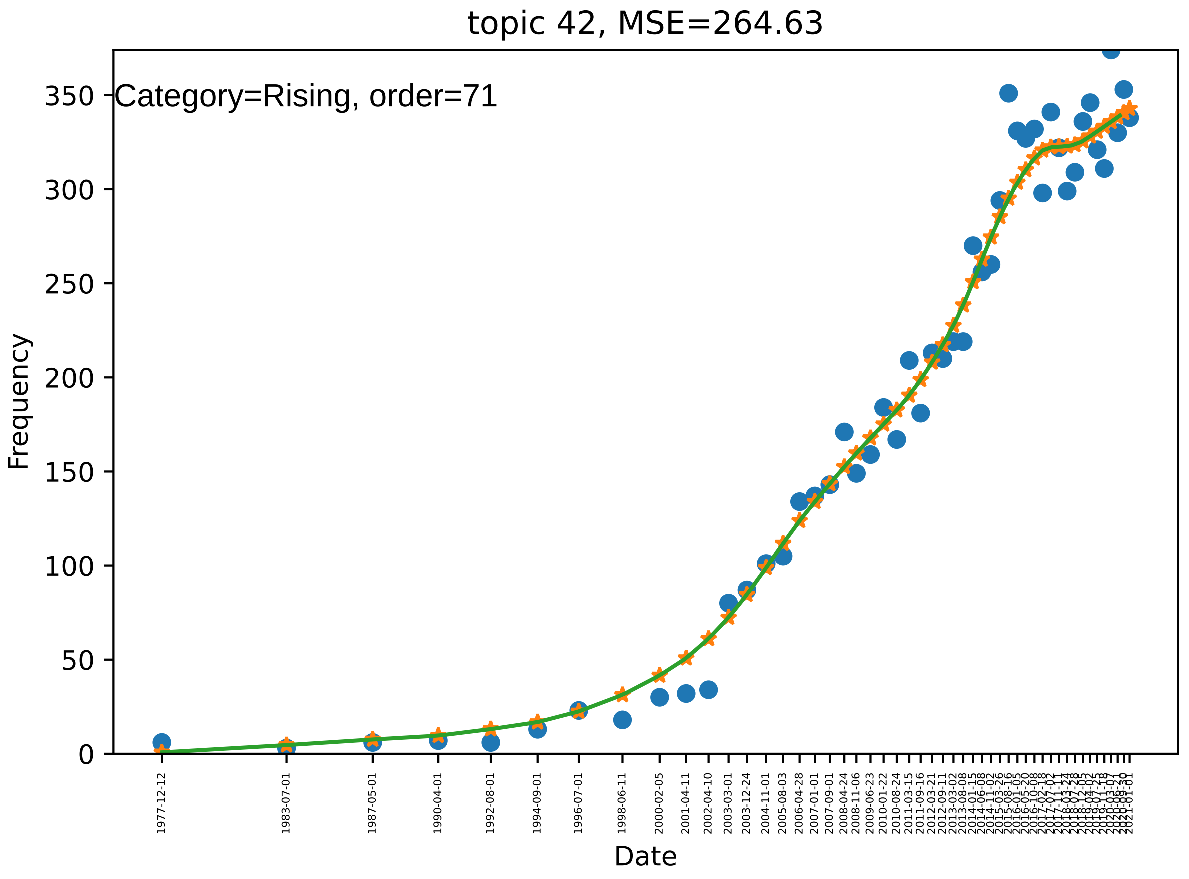

topic 42, MSE=264.63
Category=Rising, order=71
Frequency
Date
0
50
100
150
200
250
300
350
1977-12-12
1983-07-01
1987-05-01
1990-04-01
1992-08-01
1994-09-01
1996-07-01
1998-06-11
2000-02-05
2001-04-11
2002-04-10
2003-03-01
2003-12-24
2004-11-01
2005-08-03
2006-04-28
2007-01-01
2007-09-01
2008-04-24
2008-11-06
2009-06-23
2010-01-22
2010-08-24

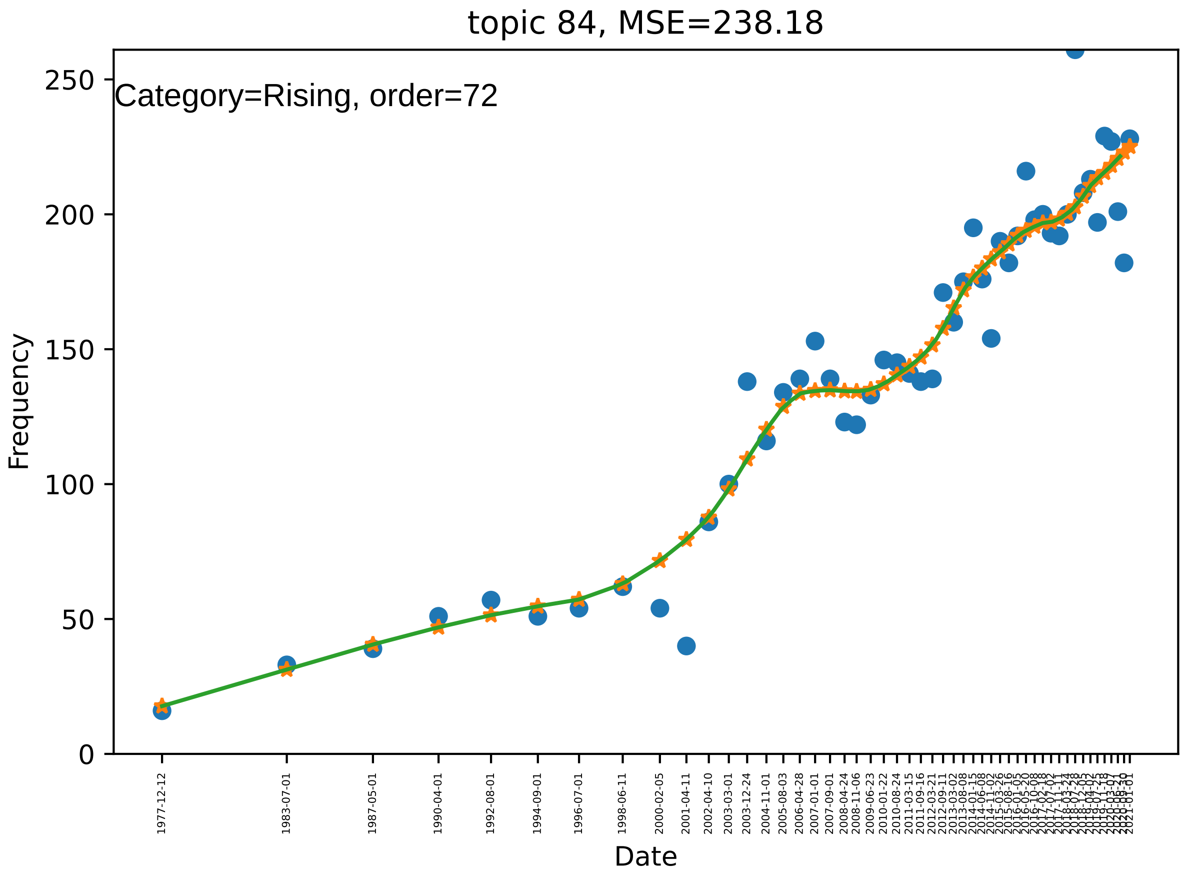

topic 84, MSE=238.18
Category=Rising, order=72
Frequency
Date
0
50
100
150
200
250

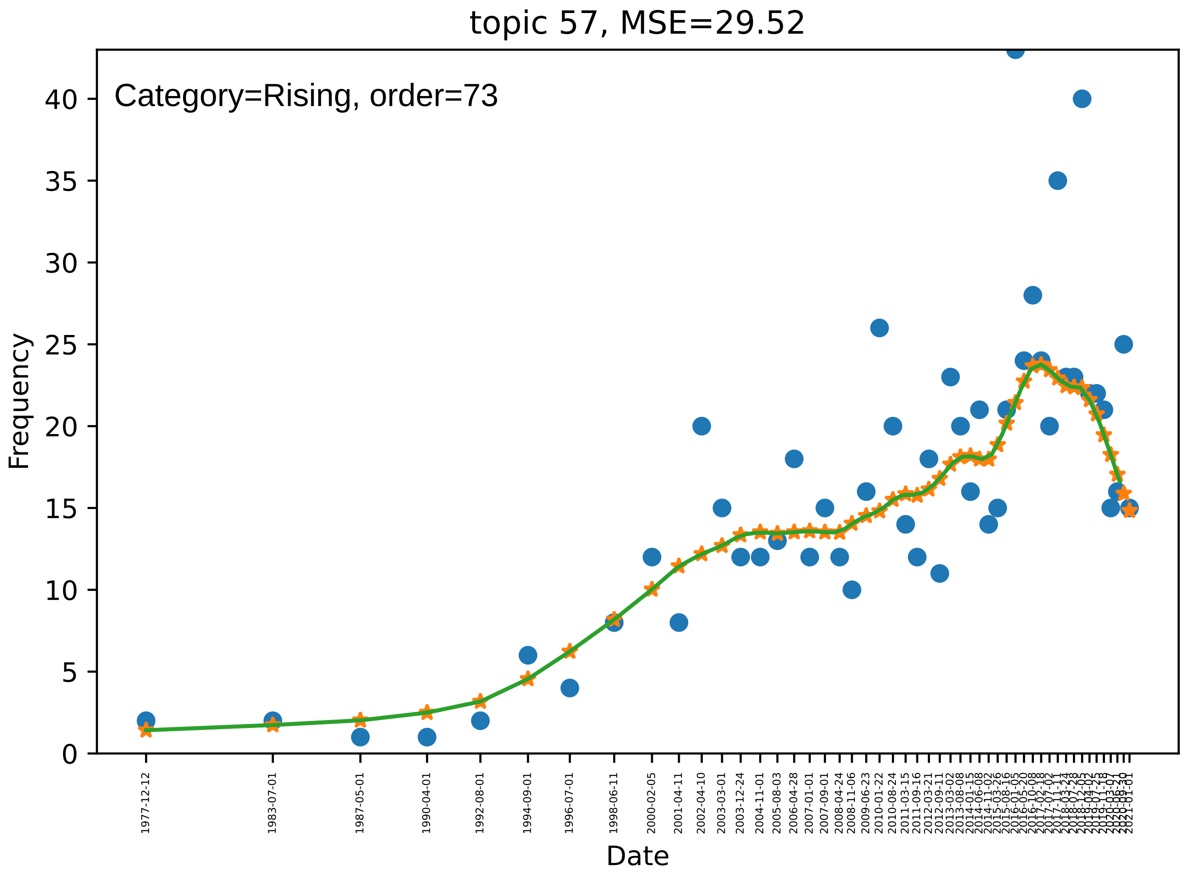
topic 57, MSE=29.52
Category=Rising, order=73
Frequency
Date
0
5
10
15
20
25
30
35
40
1977-12-12
1983-07-01
1987-05-01
1990-04-01
1992-08-01
1994-09-01
1996-07-01
1998-06-11
2000-02-05
2001-04-11
2002-04-10
2003-03-01
2003-12-24
2004-11-01
2005-08-03
2006-04-28
2007-01-01
2007-09-01
2008-04-24
2008-11-06
2009-06-23
2010-01-22
2010-08-24
2011-03-15
2011-09-16
2012-03-21
2012-09-11
2013-03-02
2013-08-08
2014-01-15
2014-06-08
2014-11-02
2015-03-26
2015-08-16
2016-01-05
2016-05-20
2016-10-08
2017-02-18
2017-07-02
2017-11-11
2018-03-24
2018-07-28
2018-12-05
2019-04-02
2019-07-25
2019-11-18
2020-03-07

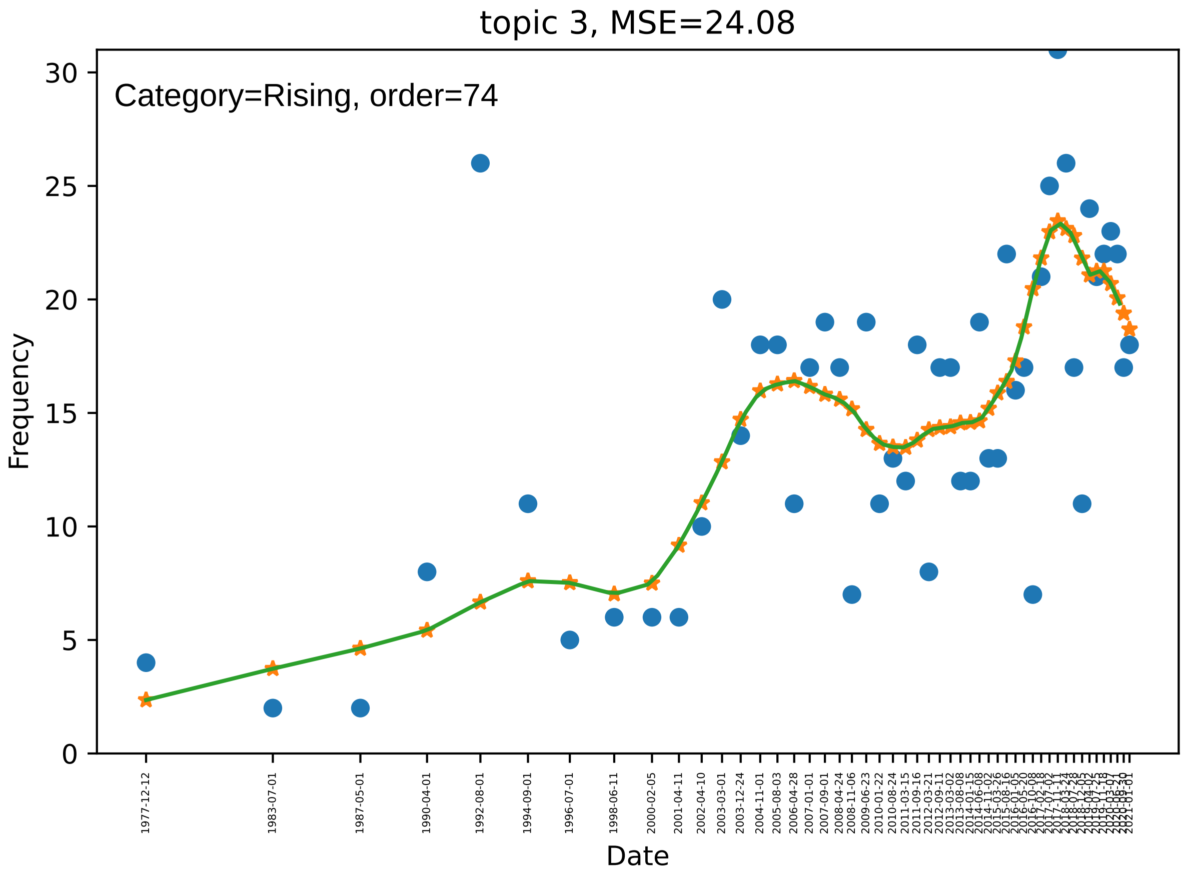

topic 3, MSE=24.08
Category=Rising, order=74
Frequency
0
5
10
15
20
25
30
Date
1977-12-12
1983-07-01
1987-05-01
1990-04-01
1992-08-01
1994-09-01
1996-07-01
1998-06-11
2000-02-05
2001-04-11
2002-04-10
2003-03-01
2003-12-24
2004-11-01
2005-08-03
2006-04-28
2007-01-01
2007-09-01
2008-04-24
2008-11-06
2009-06-23
2010-01-22
2010-08-24
2011-03-15
2011-09-16
2012-03-21
2012-09-11
2013-03-02
2013-08-08
2014-01-15
2014-06-08
2014-11-02
2015-03-26
2015-08-16
2016-01-05
2016-05-20
2016-10-08
2017-02-18
2017-07-02
2017-11-11
2018-03-24

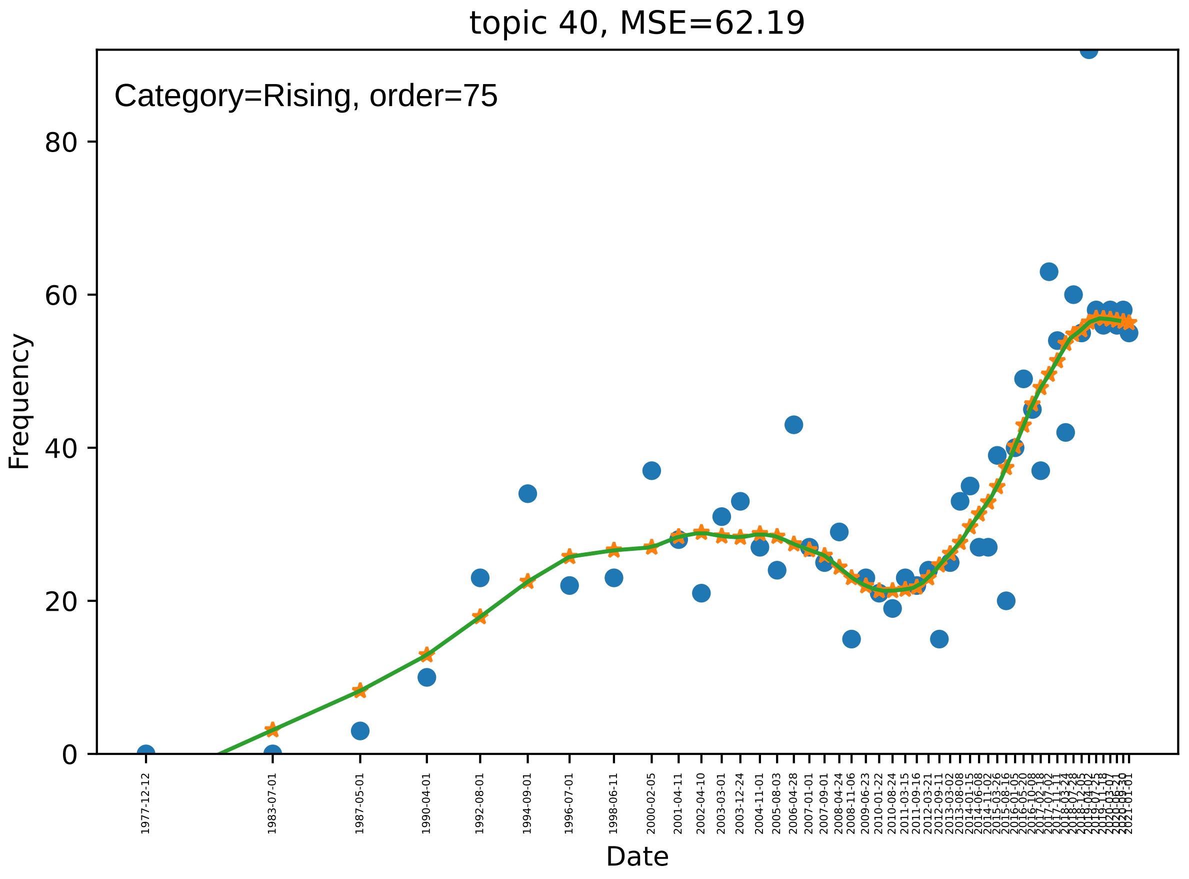

topic 40, MSE=62.19
Category=Rising, order=75
Frequency
0
20
40
60
80
Date
1977-12-12
1983-07-01
1987-05-01
1990-04-01
1992-08-01
1994-09-01
1996-07-01
1998-06-11
2000-02-05
2001-04-11
2002-04-10
2003-03-01
2003-12-24
2004-11-01
2005-08-03
2006-04-28
2007-01-01
2007-09-01
2008-04-24
2008-11-06
2009-06-23
2010-01-22
2010-08-24
2011-03-15
2011-09-16
2012-03-21
2012-09-11
2013-03-02
2013-03-08
2014-01-15
2014-06-08
2014-11-02

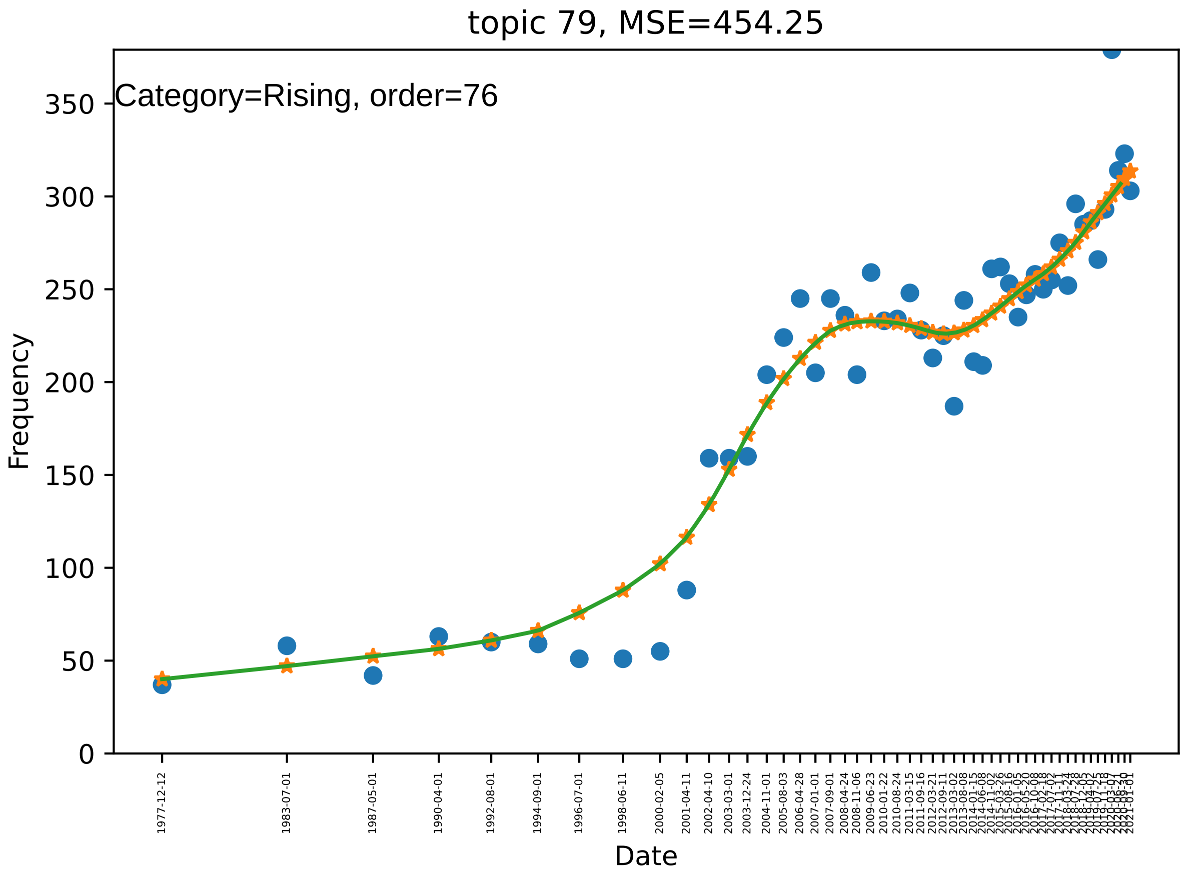

topic 79, MSE=454.25
Category=Rising, order=76
Frequency
0
50
100
150
200
250
300
350
Date
1977-12-12
1983-07-01
1987-05-01
1990-04-01
1992-08-01
1994-09-01
1996-07-01
1998-06-11
2000-02-05
2001-04-11
2002-04-10
2003-03-01
2003-12-24
2004-11-01
2005-08-03
2006-04-28
2007-01-01
2007-09-01
2008-04-24
2008-11-06
2009-06-23
2010-01-22
2010-08-24
2011-03-15
2011-09-16
2012-03-21
2012-09-11
2013-03-02
2013-08-08
2014-01-15
2014-06-08
2014-11-02
2015-03-26
2015-08-16
2016-01-05
2016-05-20
2016-10-08
2017-02-18
2017-07-02

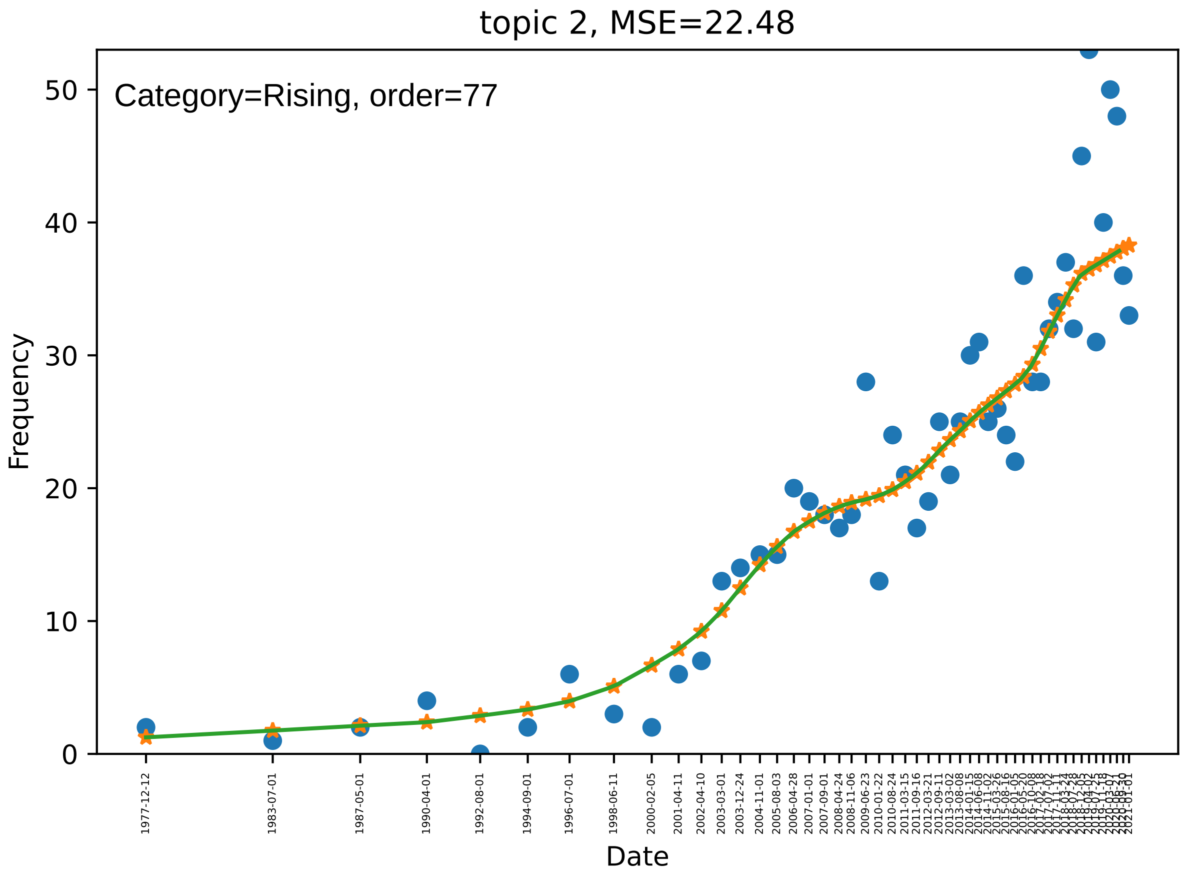
topic 2, MSE=22.48
Category=Rising, order=77
Frequency
0
10
20
30
40
50
Date
1977-12-12
1983-07-01
1987-05-01
1990-04-01
1992-08-01
1994-09-01
1996-07-01
1998-06-11
2000-02-05
2001-04-11
2002-04-10
2003-03-01
2003-12-24
2004-11-01
2005-08-03
2006-04-28
2007-01-01
2007-09-01
2008-04-24
2008-11-06
2009-06-23
2010-01-22
2010-08-24
2011-03-15
2011-09-16
2012-03-21
2012-09-11
2013-03-02
2013-08-08
2014-01-15

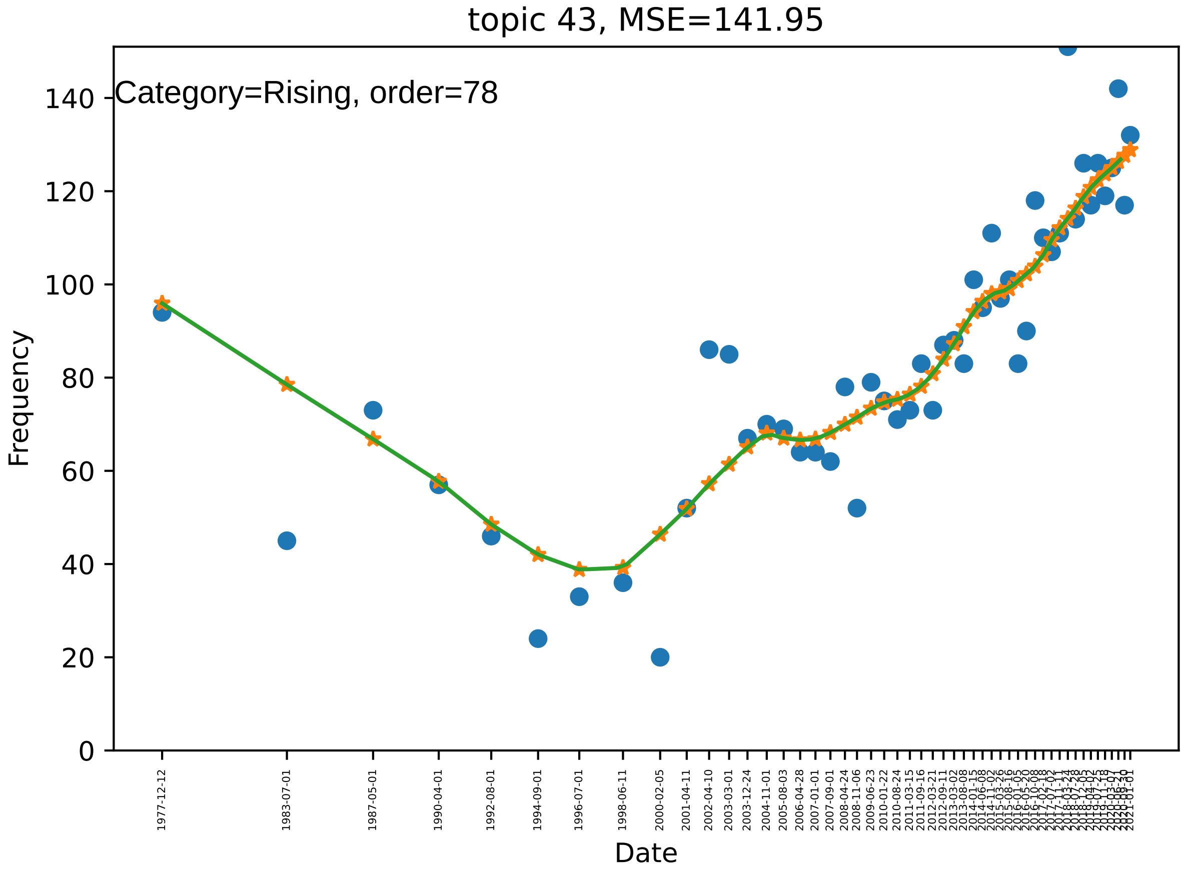
topic 43, MSE=141.95
Category=Rising, order=78
Frequency
Date
0
20
40
60
80
100
120
140
1977-12-12
1983-07-01
1987-05-01
1990-04-01
1992-08-01
1994-09-01
1996-07-01
1998-06-11
2000-02-05
2001-04-11
2002-04-10
2003-03-01
2003-12-24
2004-11-01
2005-08-03
2006-04-28
2007-01-01
2007-09-01
2008-04-24
2008-11-06
2009-06-23
2010-01-22
2010-08-24
2011-03-15
2011-09-16
2012-03-21
2012-09-11
2013-03-02
2013-08-08
2014-01-15
2014-06-08
2014-11-02
2015-03-26
2015-08-16
2016-01-05
2016-05-20
2016-10-08
2017-02-18
2017-07-02

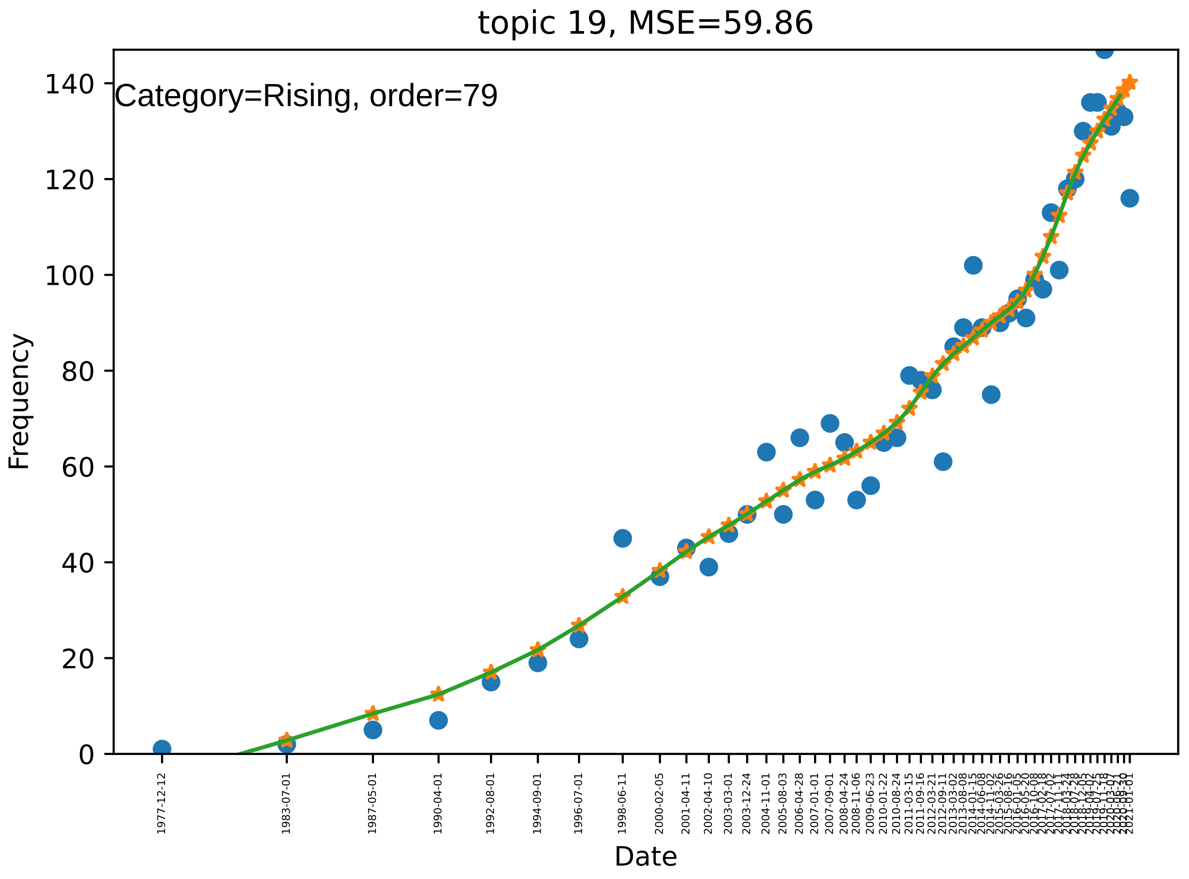

topic 19, MSE=59.86
Category=Rising, order=79
Frequency
0
20
40
60
80
100
120
140
Date
1977-12-12
1983-07-01
1987-05-01
1990-04-01
1992-08-01
1994-09-01
1996-07-01
1998-06-11
2000-02-05
2001-04-11
2002-04-10
2003-03-01
2003-12-24
2004-11-01
2005-08-03
2006-04-28
2007-01-01
2007-09-01
2008-04-24
2008-11-06
2009-06-23
2010-01-22
2010-08-24
2011-03-15
2011-09-16
2012-03-21
2012-09-11
2013-03-02
2013-08-08
2014-01-15
2014-06-08
2014-11-02
2015-03-26
2015-08-16
2016-01-05
2016-05-20
2016-10-08

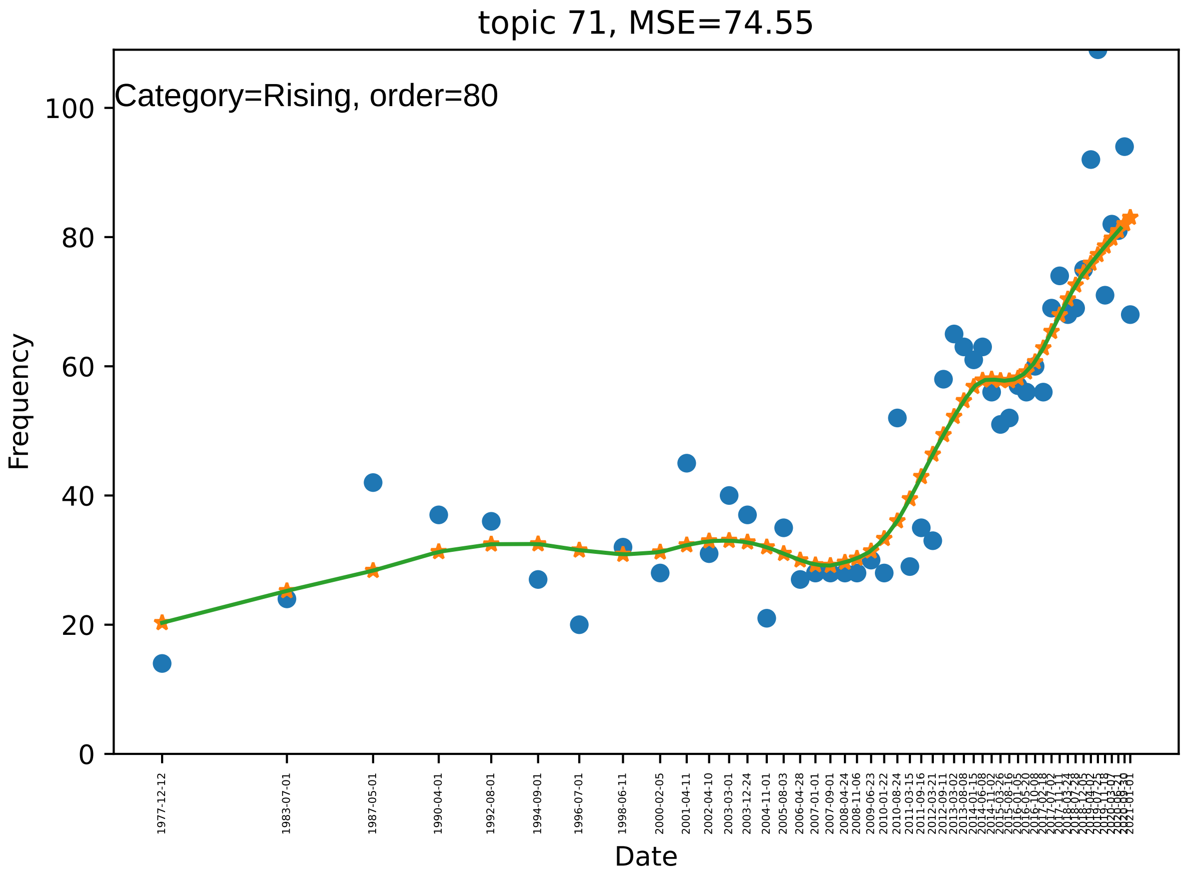
topic 71, MSE=74.55
Category=Rising, order=80
Frequency
Date
0
20
40
60
80
100
1977-12-12
1983-07-01
1987-05-01
1990-04-01
1992-08-01
1994-09-01
1996-07-01
1998-06-11
2000-02-05
2001-04-11
2002-04-10
2003-03-01
2003-12-24
2004-11-01
2005-08-03
2006-04-28
2007-01-01
2007-09-01
2008-04-24
2008-11-06
2009-06-23
2010-01-22
2010-08-24
2011-03-15
2011-09-16
2012-03-21
2012-09-11
2013-03-02
2013-08-08
2014-01-15
2014-06-08
2014-11-02
2015-03-26
2015-08-16
2016-01-05
2016-05-20
2016-10-08
2017-02-18
2017-07-02
2017-11-11

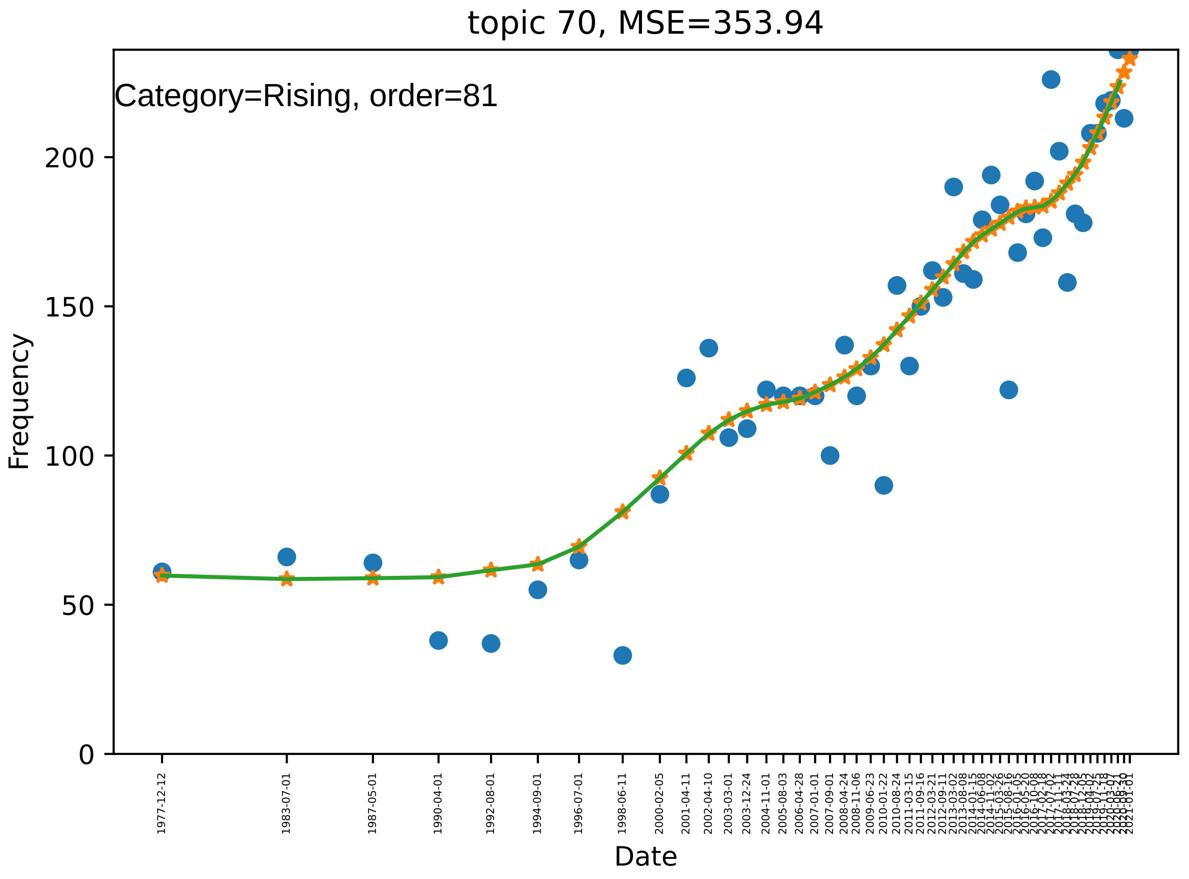
topic 70, MSE=353.94
Category=Rising, order=81
Frequency
0
50
100
150
200
Date

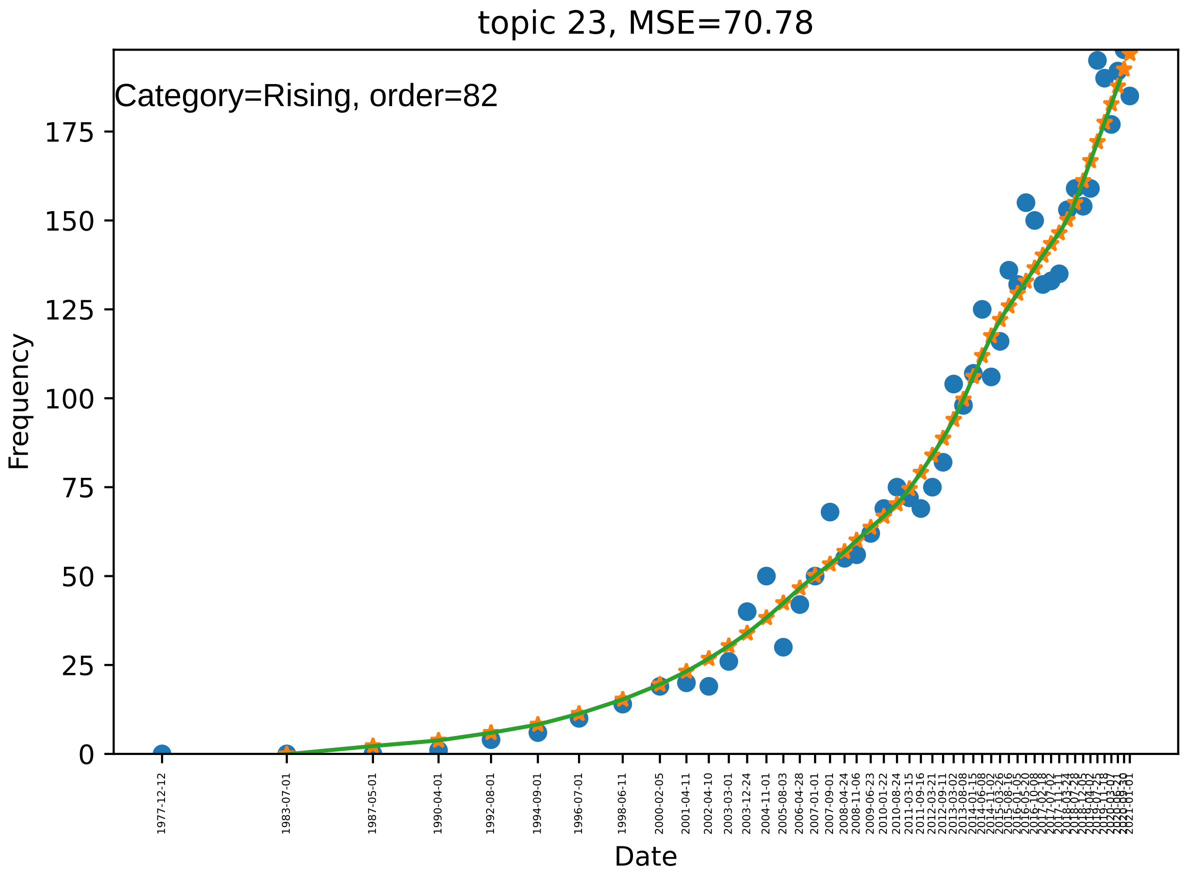
topic 23, MSE=70.78
Category=Rising, order=82
Frequency
0
25
50
75
100
125
150
175
Date
1977-12-12
1983-07-01
1987-05-01
1990-04-01
1992-08-01
1994-09-01
1996-07-01
1998-06-11
2000-02-05
2001-04-11
2002-04-10
2003-03-01
2003-12-24
2004-11-01
2005-08-03
2006-04-28
2007-01-01
2007-09-01
2008-04-24
2008-11-06
2009-06-23
2010-01-22
2010-08-24
2011-03-15
2011-09-16
2012-03-21
2012-09-11
2013-03-02
2013-08-08
2014-01-15
2014-06-08
2014-11-02
2015-03-26
2015-08-16
2016-01-05
2016-05-20
2016-10-08

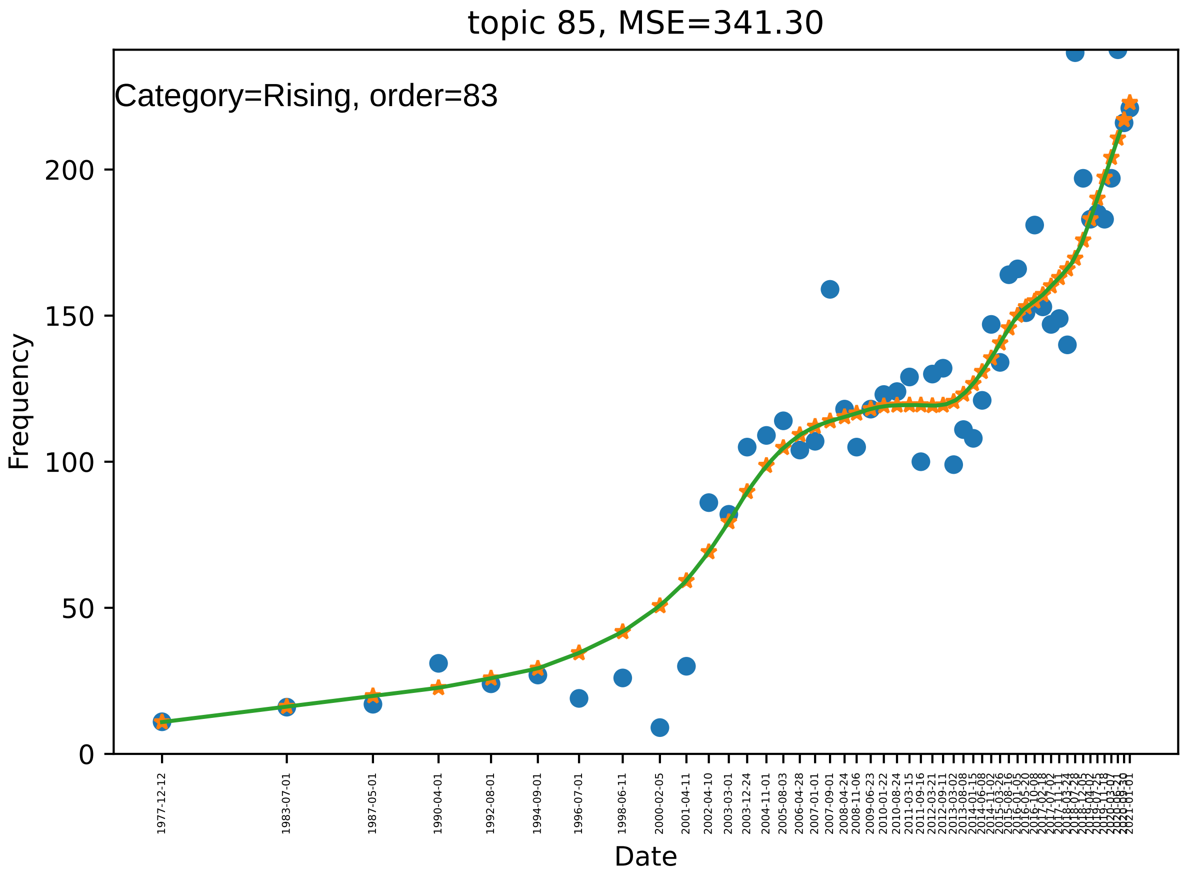
topic 85, MSE=341.30
Category=Rising, order=83
Frequency
0
50
100
150
200
Date
1977-12-12
1983-07-01
1987-05-01
1990-04-01
1992-08-01
1994-09-01
1996-07-01
1998-06-11
2000-02-05
2001-04-11
2002-04-10
2003-03-01
2003-12-24
2004-11-01
2005-08-03
2006-04-28
2007-01-01
2007-09-01
2008-04-24
2008-11-06
2009-06-23
2010-01-22
2010-08-24
2011-03-15
2011-09-16
2012-03-21
2012-09-11
2013-03-02
2013-08-08
2014-01-15
2014-06-08
2014-11-02
2015-03-26
2015-08-16
2016-01-05
2016-05-20
2016-10-08

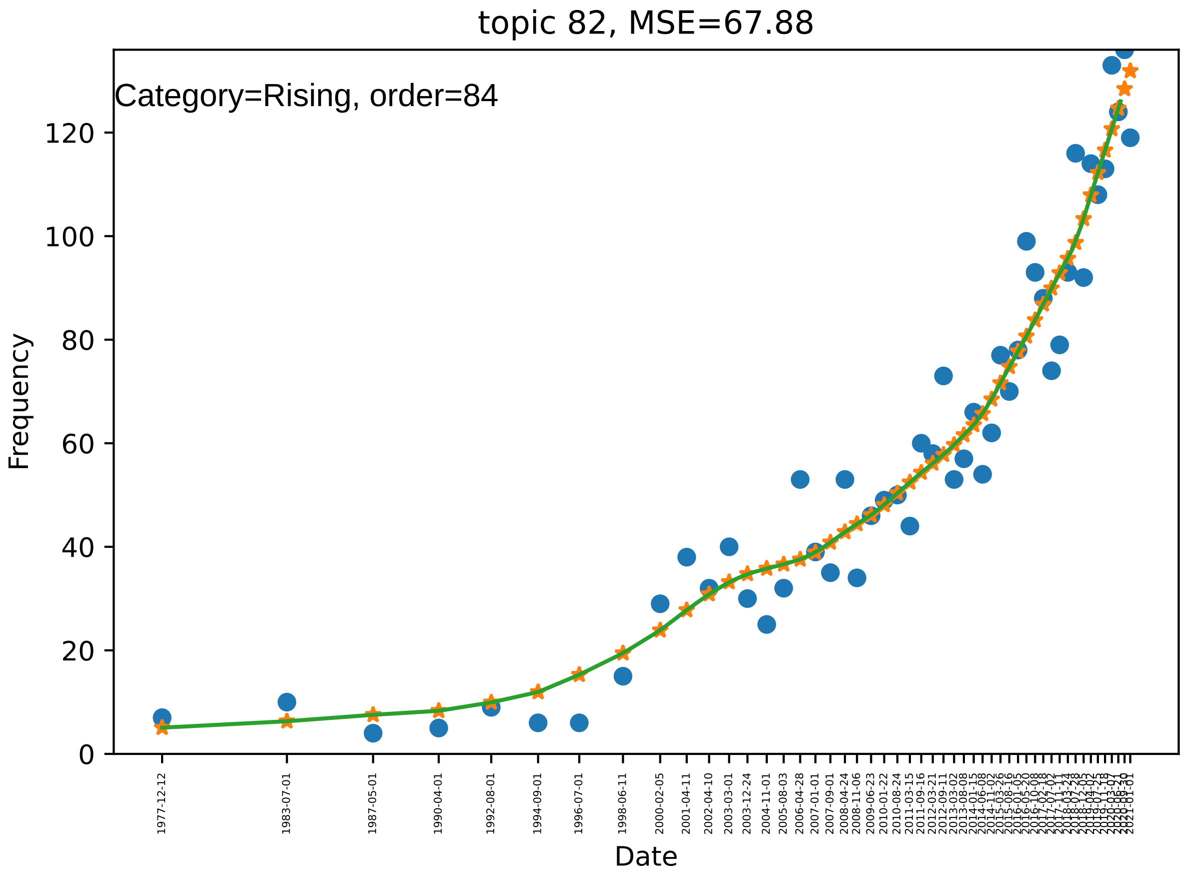

topic 82, MSE=67.88
Category=Rising, order=84
Frequency
0
20
40
60
80
100
120
Date
1977-12-12
1983-07-01
1987-05-01
1990-04-01
1992-08-01
1994-09-01
1996-07-01
1998-06-11
2000-02-05
2001-04-11
2002-04-10
2003-03-01
2003-12-24
2004-11-01
2005-08-03
2006-04-28
2007-01-01
2007-09-01
2008-04-24
2008-11-06
2009-06-23
2010-01-22
2010-08-24
2011-03-15
2011-09-16
2012-03-21
2012-09-11
2013-03-02
2013-08-08
2014-01-15
2014-06-08
2014-11-02
2015-03-26
2015-08-16
2016-01-05
2016-05-20
2016-10-08
2017-02-18
2017-07-02

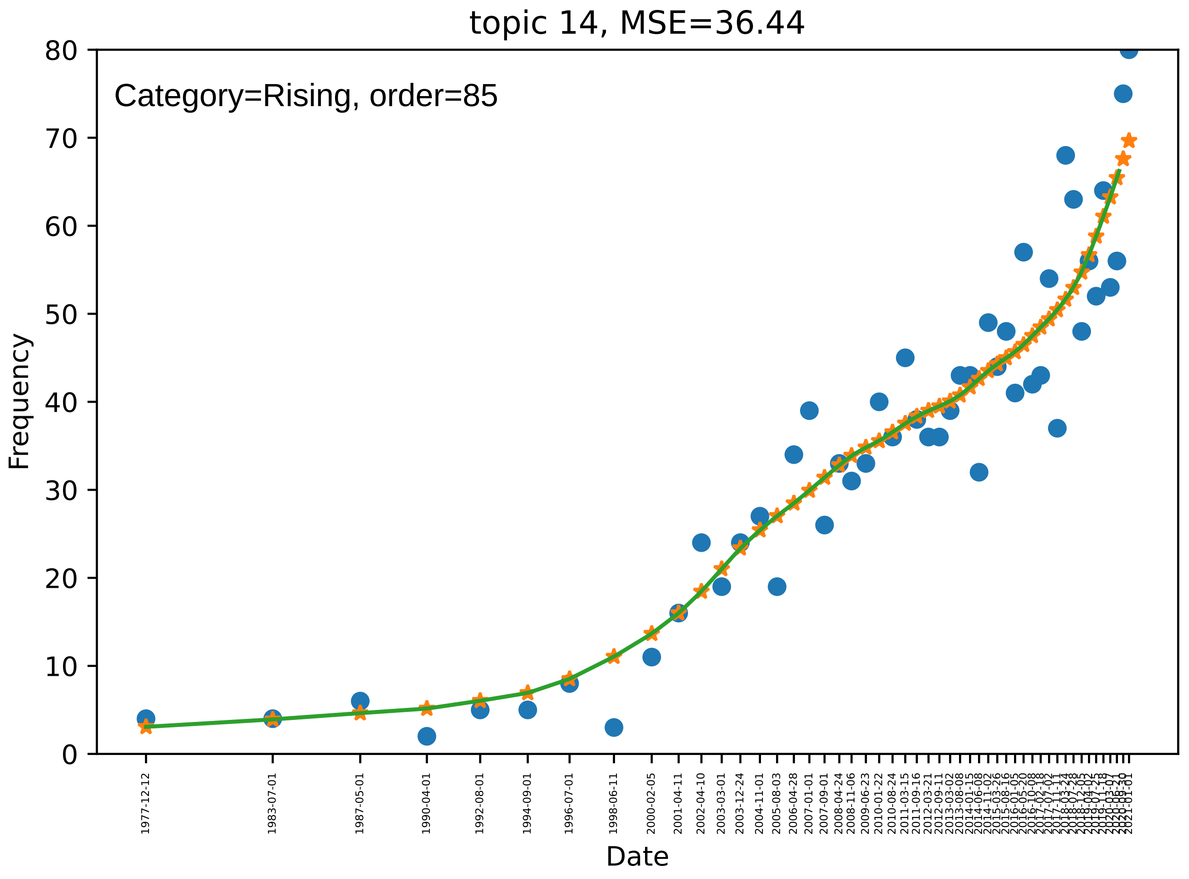

topic 14, MSE=36.44
Category=Rising, order=85
Frequency
Date

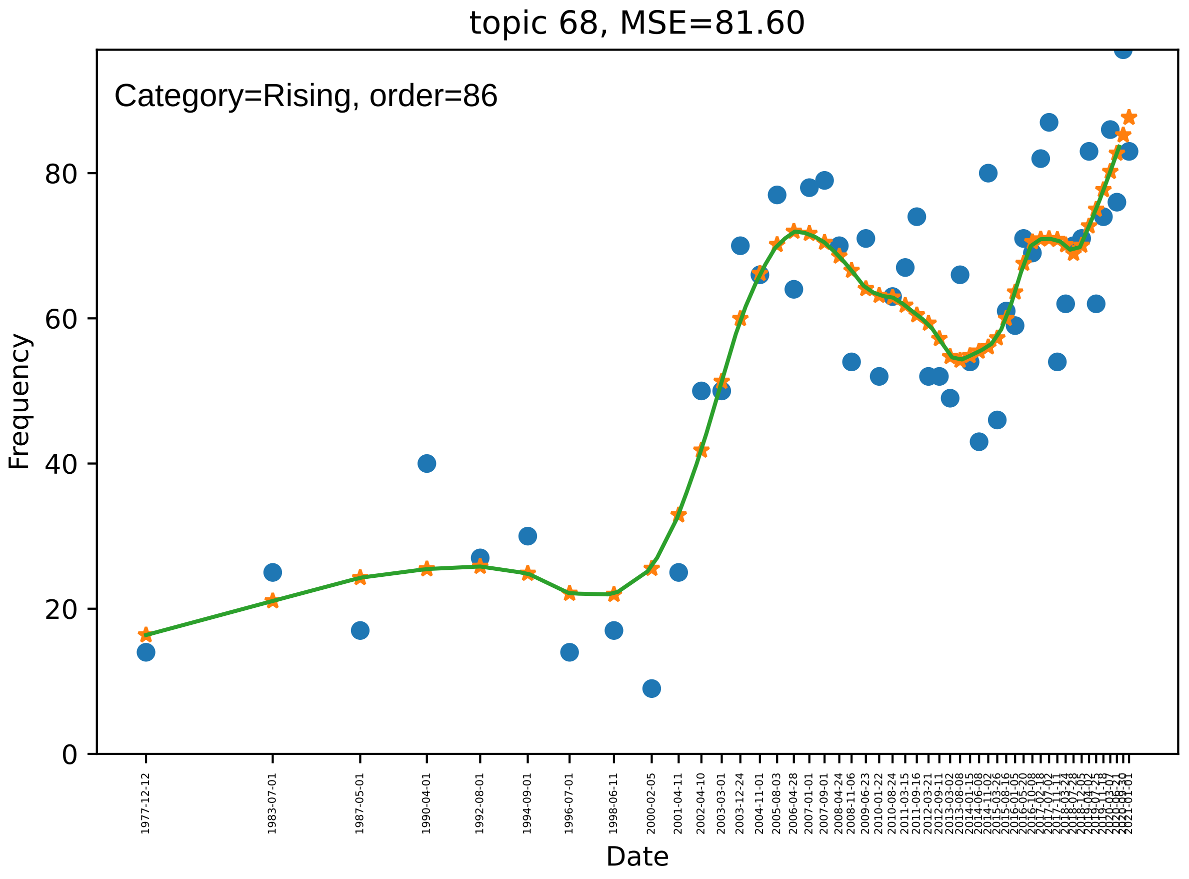

topic 68, MSE=81.60
Category=Rising, order=86
Frequency
Date
0
20
40
60
80

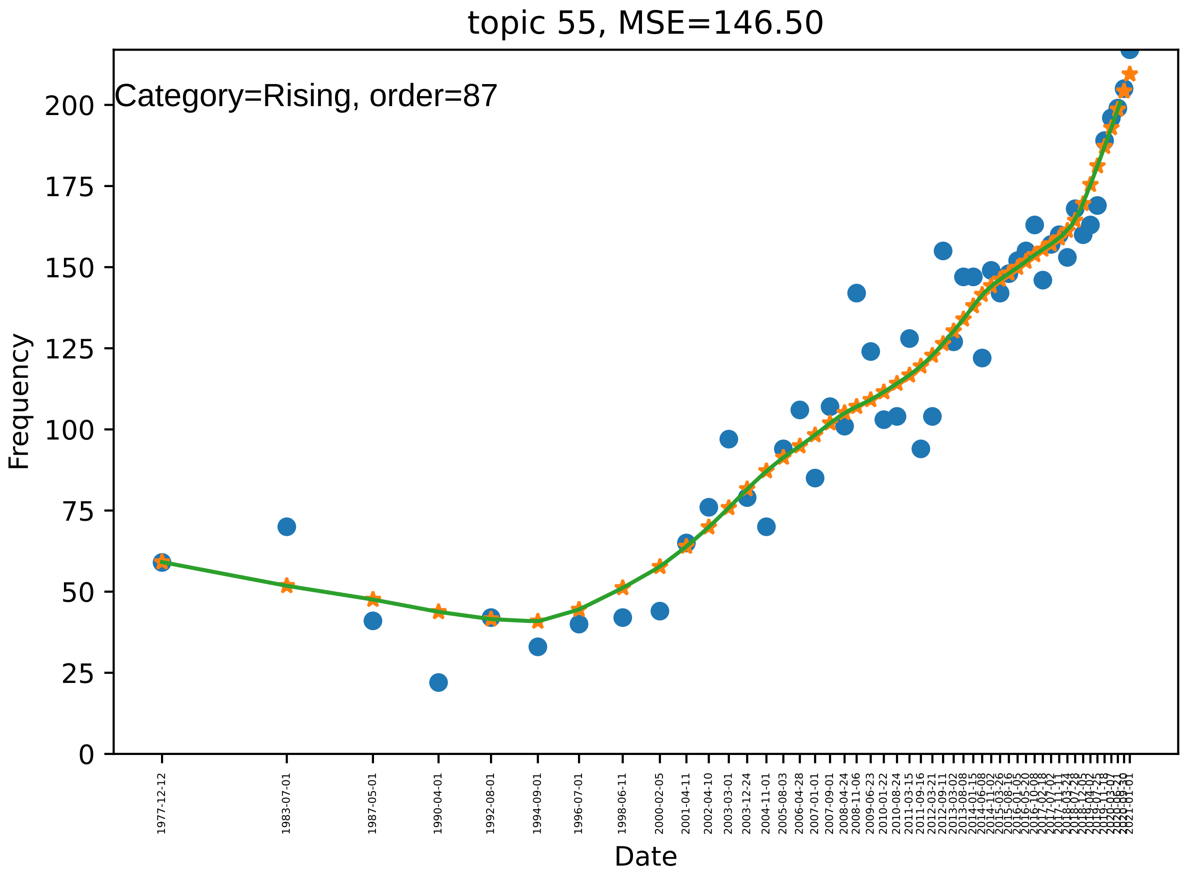
topic 55, MSE=146.50
Category=Rising, order=87
Frequency
0
25
50
75
100
125
150
175
200
Date
1977-12-12
1983-07-01
1987-05-01
1990-04-01
1992-08-01
1994-09-01
1996-07-01
1998-06-11
2000-02-05
2001-04-11
2002-04-10
2003-03-01
2003-12-24
2004-11-01
2005-08-03
2006-04-28
2007-01-01
2007-09-01
2008-04-24
2008-11-06
2009-06-23
2010-01-22
2010-08-24
2011-03-15
2011-09-16
2012-03-21
2012-09-11
2013-03-02
2013-08-08
2014-01-15
2014-06-08
2014-11-02
2015-03-26
2015-08-16
2016-01-05
2016-05-20
2016-10-08
