# Supplementary material for: Assessing the evolution of research topics in a biological field using plant science as an example
Source: PLoS Biol. 2024 May 23;22(5):e3002612. doi: 10.1371/journal.pbio.3002612 (PMC11115244; doi:10.1371/journal.pbio.3002612)
Supplement: Attachment — Submitted filename: responses_v4.docx [file pbio.3002612.s023.docx]

**Editor comments:**

Thank you for your patience while your manuscript "Evolution of research topics and paradigms in a biological field" was peer-reviewed at PLOS Biology. Your manuscript has been evaluated by the PLOS Biology editors, an Academic Editor with relevant expertise, and by two independent reviewers. Please accept our apologies for the additional delay incurred over the holiday period.

Response: This is indeed a strange but important topic. We appreciate the Editor for taking the time to evaluate this study and identify suitable reviewers.

You'll see that reviewer #1, who has expertise in scientometrics and "big data" methodology, is positive about your approach, which s/he thinks is valuable and original, but says s/he cannot assess the interest to the plant community.

Response: We appreciate reviewer 1’s feedback. The preprint for this manuscript is among the top 5% of all research output scored by Almetric, which includes published works. Thus, this demonstrates the interest of the scientific community. Social media posts for the preprint also convey the interest and excitement about our work.

Their major concern is that s/he finds their conclusion that there has been a decline in work with Arabidopsis (Fig 4C) very unlikely, which makes them worry about artefacts.

Response: This impression is due to insufficient explanation on our part. What we show is that, relative to all plant science publications, there is a reduced proportion of articles mentioning Arabidopsis. The number of Arabidopsis papers is still growing, just at a much slower rate compared to papers on other systems. During the review period, we conducted another analysis using US National Science Foundation-funded proposals. We observed the very same trend, which lends support to our findings from analysis of the literature. We have included this new analysis in the revision.

S/he also finds the code very inaccessible, and asks you to provide a jupyter notebook to help readers follow it.

Response: We understand where the reviewer is coming from and have included an additional tutorial notebook with example data to illustrate the main parts of the topic modeling process. The codes and associated documentation in the original repository faithfully document exactly what were done. We have provided an extensively revised README file for the repository to explain what the purpose of each of the subsections.

Their third major concern is that topic grouping should be done via an unbiased objective approach to avoid human bias. S/he has a range of more minor concerns too.

Response: We have addressed these concerns as detailed in our responses to reviewer below. The grouping was done with multiple approaches and criteria but in the last submission, we only provided the details through the code documentation in the repository. We have now summarized how the grouping is done in details in the method section.

Reviewer #2, who is a plant scientist, is rather more sceptical; s/he found the broad findings to be interesting, but thinks that the you may be mistaken in looking for technological explanations for shifts in the field, when conceptual advances (“paradigm shifts”) may be more likely. S/he thinks that while the approach allows one to study the influence of such factors, it may be less good at identifying them a priori.

Response: This is indeed a very interesting and important point, and we have addressed it in our response to the reviewer below. Specifically, we concur with the reviewer on the limitation of our approaches and remove any mention of paradigm shifts and use “trend” and “shift” instead. We also discuss the limitations more in our revised discussion section.

-----------------------------------------

**REVIEWS:**

***Reviewer #1:***

The article by Shiu et Lehti-Shiu performs a bibliometric analysis of trends of scientific topics in plant research over nearly one century. They further introduce methods and software that could already be applicable to other domains of biomedical research. Their work extends beyond static work in the biomedical space using BERT models (González-Márquez et al., bioRxiv, 2023) by providing temporal resolution and acting on a scale that, while large, still allows human inspection and interpretability of all topics. Their work extends beyond earlier time-resolved work on trends in the biomedical space (Telis et al., Genetics, 2016) by shifting away from individual keywords toward topics. Besides this unique combination of technology and interpretability, learning about the history of plant science felt enjoyable and valuable, albeit I lack the domain knowledge to confirm the specific insights about plant science.

Response: We appreciate the reviewer’s assessment and comparisons to two studies that are out there. We are hoping that this work can be an example of how to look at different biological disciplines or sub-disciplines beyond the plant sciences.

My primary comment relates to data presented in Figure 4C and its technical implications beyond Figure 4C. Specifically, the authors discuss a decline in Arabidopsis research, which I have personally never observed, and - more importantly - is not seen in PubMed's visualization of the time-resolved popularity of Arabidopsis (graph on left side when querying <https://pubmed.ncbi.nlm.nih.gov/?term=arabidopsis&sort=date__;!!HXCxUKc!y6BwIVDNXlq-84i-erYVflO402c7dXUv_giz_FRx0vGApkEPTeA6hi01sPRcf2oZPoB7LCwy6JwQSg$> ). A decline of Arabidopsis research also seems to stand in the opposition to observations of Telis et al., Genetics, 2016, which are based on only one journal (and thus may only provide a partial view).

Response: We regret that we did not describe this trend more clearly. When the preprint went out, we were questioned multiple times by colleagues who asked us to clarify the point the reviewer raised here. What we intended to show was that, in comparison to all plant science-related articles in our corpus, the proportion that mentioned Arabidopsis decreased, although the number of Arabidopsis papers continued to increase over time. We tried to emphasize this in the earlier version on p.9 L248 by saying “…the decline here did not mean that there were fewer Arabidopsis publications—in fact the number of Arabidopsis paper remain steady since 2011”. Regarding the curve shown in Figure 4C, one subtle point we should have also emphasized is that the time bin size was not the same (i.e., not year-by-year). Instead, it was sized to ensure that every bin had the same number of plant science articles. So, the y-axis label saying, “Total records” was misleading, and it should have read “Normalized total records”. We have changed the Figure 4C axis label and legends to make this clearer and revised the text on p. p. 10 to emphasize that the decrease is relative to studies of all the other species.

Tellis et al., 2016 Genetics discussed that Arabidopsis was at the forefront of genetic research. We should point out that the authors referred to the importance of Arabidopsis for the period between 1996 to 2006 specifically, which is consistent with what we found. Our analysis shows that the decrease in the proportion of Arabidopsis publications started after that period. Thus, there does not seem to be any contradiction.

While Figure 4C shows something unexpected to me, the underlying technical factor may also influence interpretation of other parts of the manuscript, particularly on dynamics.

Though I cannot evaluate from the distance where this difference arises, I suspect false negatives in the creation of the corpus, or a discrepancy between absolute and relative quantifications of occurrences, or a combination of both.

Response: Based on the explanation above, we hope the reviewer agrees that there is no underlying technical factor but instead an issue with clarity in our explanation. Considering this, we have revised the manuscript to highlight more clearly what the provided values mean and what the comparison groups are. For example, for Figure 3, we have added the following clarification (p.7):

“In Fig. 3, the relative decrease in document frequency does not mean that research output in a topic is dwindling. Because each row in the heatmap is normalized based on the minimum and maximum values within each topic, there still can be substantial research output in terms of numbers of publications even when the relative frequency is near 0. Thus, a reduced relative frequency of a topic reflects only a below-average growth rate compared with other topical areas.”

My second comment relates to the re-usability of the work of Shiu et Lehti-Shiu. The individual elements of their code on github look quite nice, and they already structure the code by figures. Given the scale of their code, I however could not find where to start, and in which order to call what script. I would find it very helpful if the authors created a brief jupyter notebook that walks others through their workflow to create data/analysis required for Figs. 1-3, and referred to this notebook in the README.md file in the top of the repository. This notebook could use plants but should convey to people interested in other domains (e.g.: invertebrate biology) how/where to modify code.

Response: We agree with the reviewer that a notebook illustrating the general approach will be helpful. As the reviewer recommended, we have created an “_example” folder that contains: (1) a notebook that makes use of a subset of the plant science records (i.e., those mentioning Arabidopsis) in the GitHub repository, (2) a standalone README file to instruct others in how the notebook can be run, and (3) a requirement file containing package version information. This code will allow readers to conduct topic modeling with a much smaller dataset using the same steps that we used: generate a topic model, obtain the top terms for topics, and determine the frequencies of topics over time. This should give the readers a good starting point.

We have also revised the main README file in the repo extensively to provide info on the content in each directory and subdirectory. The reviewer asked that we provide codes for Figure 1-3. Instead of providing them in the tutorial notebook, we created a detailed README file to point the readers to the location of the codes for generating the figures within the current repository:

<https://github.com/ShiuLab/plant_sci_hist>

Substantial efforts were made to customize the look-and-feel of these figures, particularly for Figure 3, and the details are provided in a new methods section “Topic categories and ordering” on p.20-21 (also see our response to the next point the reviewer raised).

In addition to what the reviewer requested, we have documented the codes (script_9_3a, b, c, d in section 9 of the repo) for the new analyses conducted in response to the reviewers’ comments.

My third comment is that it is presently unclear how topics were grouped in Figure 3, and to which extent this could be the result of human decisions. Preferentially, the grouping would be the result of some unbiased or formalized procedure.

Response: Thank you for pointing this out. We documented the criteria for the grouping in the associated notebooks but not in the manuscript, which was an oversight. We used multiple methods to group the topics, and human decisions were kept to a minimum. These methods are now described in detail in a new method section, “Topic categories and ordering” on p.20-21, and two new datasets, S7 (LOWESS fit of all topics) and S8 (criteria for sorting topics), have been created as supplements.

The new method section on p.19 reads as follows: “The topics were classified into five categories with contrasting trends: stable, early, transitional, sigmoidal, and rising. To define which category a topic belongs to, the frequency of documents over time bins for each topic was analyzed using three regression methods. We first tried two forecasting methods: recursive autoregressor (the ForcastAutoreg class in the skforecast package) and autoregressive integrated moving average (ARIMA implemented in the pmdarima package). In both cases, the forecasting results did not clearly follow the expected trend lines, likely due to the low numbers of data points (relative frequency values), which resulted in the need to extensively impute missing data. Thus, as a third approach, we sought to fit the trendlines with the data points using LOWESS (implemented in the statsmodels package) and applied additional criteria for assigning topics to categories. When fitting with LOWESS, three fraction parameters (frac, the fraction of the data used when estimating each y-value) were tested (0.1, 0.2, 0.3). While frac=0.3 had the smallest errors for most topics, in situations where there were peculiar outliers, frac=0.2 or 0.1 was chosen to minimize mean squared errors (new Dataset S7). The topics were classified into five categories based on the slopes of the fitted line over time: (1) stable: topics with near 0 slopes over time, (2) early: topics with negative (<-0.5) slopes throughout (with the exception of topic 78, which declined early on but bounced back by the late 90s), (3) transitional: early positive (>0.5) slopes followed by negative slopes at later time points, (4) sigmoidal: early positive slopes followed by zero slopes at later time points, (5) rising: continuously positive slopes. For each topic, the LOWESS fits were also used to determine when the relative document frequency reached its peak, first reaching a threshold of 0.6 (chosen after trial and error for a range of 0.3–0.9), and the overall trend. The topics were then ordered based on: (1) whether they belonged to the stable category or not, (2) whether the trends were decreasing, stable, or increasing, (3) the time the relative document frequency first reached 0.6, and (4) the time that the overall peak was reached (new Dataset S8).”

These are my peripheral comments:

Corpus is based on keywords, but usage of keywords has changed, and increased over decades - possibly contributing to growth of the corpus shown in Figure 1A. If not contributing to my primary comment (above), a half sentence and supplemental figure on share of PubMed with keywords could assist interpretation of Figure 1A.

Response: We did not use information from the keyword field in the PubMed records. Rather, we directly searched for the following terms (which we referred to as keywords): "plant," "plants," "botany," "botanical," “planta,” and “plantarum” within titles and abstracts. Therefore, the change in the usage of the keyword field in the PubMed records should not be an issue. We have clarified this in the methods section “Collection and pre-processing of a candidate plant science corpus” on p.14.

Some of the articles in PubMed do use languages other than English. It is unclear if they would form a separate topic, be dispersed outliers, or - given the usage of BERT - may even appear in topics irrespective of language.

Response: Because all positive examples were in English during the classification stage when the plant science corpus was defined, non-English records would be treated as non-plant science records and not subjected to further analysis. However, non-English journals with English titles and abstracts were still analyzed. Therefore, we double checked the PubMed language tags of records in the plant science corpus and found that non-English journals account for 2.9% of records in the Plant Science Corpus. Because of the low percentage of non-English records, their impact is expected to be minimal.

Similarly, it is unclear to which extent the growth reported for China and India is truly exceptional growth of plant research in these countries, or increased publishing in English, or efforts of NCBI to include literature written in languages other than English. While such a distinction could be obtained by analyzing plant-related publications contained in OpenAlex, a few extra words would seem to suffice.

Response: To address the reviewer’s concern here, we conducted additional analyses of the top 17 plant science journals, which are historically the major plant science publishers and are used as positive examples in our plant science classification model. In the new Fig. S7D, the general trends we observed in Fig. 5B remain the same: the publication volume from China and India was higher than that of the other top publishing countries such as the US and Great Britain. There are some differences between Fig. 5B and Fig. S7D. One is that the proportions of papers published by the US and Great Britain were slightly lower in Fig. S7D (17 journals) than in Fig. 5B (entire corpus). India published more in the 17 plant science journals till 2015, then the number of publications dropped to a level more consistent with the overall trend observed in Figure 5B. In both figures, China’s high rates of growth were the same. We hope that this additional analysis conveys the truly exceptional growth in plant research in these countries.

The results section opens with a statement on comparing different models, but the methods section and referred supplemental figure did not allow me to understand, which specific approaches were evaluated and whether differences in performance were significant. Aside from selling them short, inability to follow this initial scientific statement may deter some readers from proceeding to the rest of the publication, which is quite accessible.

Response: We apologize for being unclear. The original intent was to dive into the more interesting results sooner, and we ended up leaving important details out or moving them to the methods. As the reviewer suggested, we have modified the beginning of the results section to explain more clearly what the text classification models are, define the important terms for the models, and discuss the model performance in classifying the positive and negative examples. These revisions are found on p.4 of the revised manuscript.

Independent of above paragraph, it would be good to see an evaluation of false positive/false negatives in the creation of the corpus. Comparing against a corpus formed by NCBI's MeSH term D010944 (Plants) would seem reasonable as they are an authority and follows good practices. The authors could, for instance, obtain MeSH by running [https://urldefense.com/v3/__https://github.com/titipata/pubmed_parser__;!!HXCxUKc!y6BwIVDNXlq-84i-erYVflO402c7dXUv_giz_FRx0vGApkEPTeA6hi01sPRcf2oZPoB7LCzVFtnjMA$](https://urldefense.com/v3/__https:/github.com/titipata/pubmed_parser__;!!HXCxUKc!y6BwIVDNXlq-84i-erYVflO402c7dXUv_giz_FRx0vGApkEPTeA6hi01sPRcf2oZPoB7LCzVFtnjMA$) on the files that they already downloaded, and obtain the children terms of D010944 via the definition of MeSH terms downloadable on NCBI/'MeSH's ftp server.

Response: Thanks for the suggestion. In our original submission, we evaluated the corpus in terms of false positive rate (5.2%) and false negative rate (2.6%) using hold-out testing positive and negative examples. This was reported in the “Plant science record classification” section of the Methods, and the results were summarized in Figure S1.

We have also conducted the analysis as suggested. Records with the MeSH term “Plants” also include pharmaceutical studies of plants and plant metabolites or immunological studies of plants as allergens in journals that are not generally considered plant science journals (e.g., *Acta astronautica*, *International journal for parasitology*, *Journal of chromatography*) or journals from local scientific societies (e.g., *Acta pharmaceutica Hungarica*, *Huan jing ke xue*, *Izvestiia Akademii nauk*. *Seriia biologicheskaia*). Because we explicitly label papers from such journals as negative examples, we focused on 4,004 records with the “Plants” MeSH term published in the 17 plant science journals that used as positive instances and found that 88.3% were predicted as the positive class. Thus, based on the MeSH term, there is a 11.7% false prediction rate. The prediction probability distribution of positive instances with the “Plants” MeSH term has an expected left-skew to lower values (new **Figure S1C**) compared to the distributions of all positive instances (**Figure S1A**). Thus, this subset with the MeSH term is a biased representation of articles from these 17 major plant science journals. We speculate that the higher false prediction rate compared to that observed using all positive instances may be because the “Plants” MeSH term was assigned by annotators with a biomedical perspective rather than a plant science one which limits its utility as a benchmark. We have included this point when discussing false classification rate in p.4 and the approach for using the MeSH term is explained in p. 17.

As Figure 2 and 3 are very information rich, more direct references from text toward figure could assist reading (e.g.: noting that topics 27, 26, 25 are in bottom left corner; or that evolution of topics is seen for topic cluster F shown in blue).

Response: We have added more direct in-text references to the information shown in Fig. 2 and 3 as the reviewer suggested (p.5 to p.8).

The manuscript would to me seem equally strong if substituting "paradigm shift" by "shift". Similarly, if title was shortened to remove paradigms. Though the authors follow a common-language use of "paradigm shift" this usage does not capture Kuhn's original work, where paradigm shifts are preceded by long periods of stagnation and very rare. This is not in line with the usage of "paradigm shift" as part of Figure 3. Further, Kuhn compared paradigms to declinations in grammar, whereas the present manuscript uses words as a surrogate of paradigms.

Response: We agree with the reviewer that the use of paradigm shift does not fit with Kuhn’s original definition and have removed the word “paradigm” throughout. In addition, we emphasize in the discussion that, while we were interested in identifying paradigm shifts, the ability of our approach to identify these shifts is limited (p.13).

Figure 5D is framed as being "interesting", but would seem to align well with the phenomenon of "regression to the mean".

Response: We appreciate the reviewer’s point here. We now include a discussion (on p.12) of our hypothesis that the pattern in Figure 5D may be a consequence of the ease in scientific communication among geographically isolated research groups. It could also be because of the prevalence of open access online journals, which makes scientific information more readily accessible. Or it can be due to increasing international collaboration. In any case, the causes for the regression toward the mean are not immediately clear and should be addressed in future studies.

For Figure 5F it is not clear which of the changes are statistically significant, particularly if controlling for testing of multiple hypotheses.

Response: We previously reported the log likelihood ratios to show effect sizes and conducted statistical tests on these changes but did not perform multi-testing correction. Enrichment values with significant corrected p-values are now indicated in Figure 5F. In addition, we created a new file, Data S9, with the log likelihood ratios and corrected p-values.

We note that Figure 4D shows a similar analysis but for species. We have also provided multi-testing corrected p-values in the revised Data S10 and modified Figure 4D to indicate cells with significant corrected p-values.

The focus on topics rather than individual terms promises enhanced robustness of conclusions. However, this is not directly tested. One quick analysis would be to take the top term of each topic in Figure 3, and use it as a surrogate of the topic. Would single terms yield similar observations or very different ones? Either outcome would help others to better grasp the practical consequences of the methodological advances contained in this manuscript.

Response: It would be challenging to represent the topics using only the top term. We intended Figure 2B,C to illustrate this point, but we were not sufficiently explicit. For example, auxin is the top term in topic 25 (auxin | signaling | cell | development | growth) and topic 26 (auxin | light | signaling | phytochrome | mutant). Using auxin as a surrogate would lead to the merging of two topics, which have very different foci—the former focuses on signaling mechanisms at the cellular and developmental levels and the latter connects auxin with light signaling using loss-of-function mutants. We have now revised the discussion of Fig. 2B, C to use these examples to highlight the importance of using multiple top terms to represent the topics (p.6).

The discussion section suggests that technological innovation has driven research in plant sciences as key terms frequently are technologies. However, among the 80+ topics, only around 4-6 top terms relate to technologies.

Response: We should have been clearer on this point. What we meant to indicate is that the key terms defining the topics frequently describe various technologies (e.g., topic 38/39: transformation, 40: genome editing, 59: genetic markers, 65: mass spectrometry, 69: nuclear magnetic resonance) or are indicative of studies enabled by molecular genetics and omics technologies (e.g., topic 8/60: genome, 11: epigenetic modifications, 18: molecular biological studies of macromolecules, 13: small RNAs, 61: quantitative genetics, 82/84: metagenomics). Thus, our analysis highlights how technological innovation, particularly in the realm of omics has contributed to the proliferation of a substantial number of research topics in the plant sciences. We have incorporated these points in the discussion section on p.13.

For time resolved binning, find it very clever to bin multiple years prior mid-1990ies to increase signal, but would find it technically safer and more directly human interpretable to use individual years as the resolution of analysis afterwards. This would also dampen technical noise associated with different publication frequencies (Jan 1st often attracts journals/sub-field on annual publication schedule, while other journals/sub-fields are spread out over the year when following monthly schedules).

Response: We appreciate that the reviewer finds our approach useful. Regarding the reviewer’s suggestions on using individual years for analysis, we have done as the reviewer suggested for the analyses leading to Figure 1 and 5. This approach is reasonable for these two figures because the rate (number of pubs/year) is an important point we want to make (Fig. 1, Fig. 5B), and because the factors we examined were already normalized by the total number of records in each time bin (e.g., citation score in Fig. 5C).

For the results reported in other figures, we did start by analyzing the data year-by-year but realized that it was challenging to compare trends across topics because all appeared to be increasing. Although we considered using the differences in slopes as the basis of comparison, we found that they were difficult to explain and not easy to interpret. We also found it challenging to distinguish the five topical categories using individual years because, again, nearly all of them had increased numbers of publications each year. For these reasons, we chose to bin the documents in Fig. 3 and 4 so that there were the same numbers of documents per bin.

Regarding the reviewer’s point about technical noise, we concur that noise could impact the most recent time bins because they represent much narrower time periods. However, when we examined the relative frequencies across all topics (see new Dataset S7), we found that recent time bins have similar variances in relative frequencies as other time bins. We also moderated the impact of variation using LOWESS (10% to 30% of the datapoints were used for fitting the trend lines) to determine topical trends for Fig. 3. Thus, the influence of the variation is expected to be minimized. We have incorporated the above discussion in the “Dynamic topic modeling” method section on p.20.

The projection of publications in a 2D space in Figure 1C is great but made me curious about seeing publication years superimposed. This would help to see how science shifted over the space of the corpus.

Response: We were also curious to find out after the reviewer raised this interesting point. We have now generated similar UMAP plots by year and summarized the result as a new figure (Figure 2D) and animation (Data S5). As shown in Fig. 2D, there is substantial fluctuation in the positions of records in the 2D space over time. For example, topic 44 (light, leave, co, synthesis, photosynthesis) existed in 1975 but the numbers of articles gradually decreased thereafter. In 1985, topic 39 (Agrobacterium-based transformation) became dense enough to be visualized. Additional examples include topic 79 (soil heavy metals), 42 (differential expression), and 82 (bacterial community metagenomics), which became prominent in ~2005, 2010, and 2020, respectively (Fig. 2D). In addition, animating the document occupancy in the 2D space over time revealed a broad change in pattern over time: some initially dense areas became sparse over time and a large number of topics in areas previously only loosely occupied at the turn of the century increased over time (Data S5). While the 2D representations reveal substantial details on the evolution of topics, comparison over time is challenging because the number of plant science records has grown exponentially. This also reinforces the need for binning, where bins consist of similar numbers of documents to facilitate comparison. The above discussion is now incorporated on p.7 of the manuscript.

***Reviewer #2:***

This paper presents a comprehensive analysis of plant science topics over the last 50 years with a view to identifying key conceptual or technological innovations (paradigm shifts). After arriving at 90 topics, each defined by a combination of about 5 terms, the authors analyse topic frequency over time. They show that some topics have declined, others have had a transitory popularity, and others are on the rise. The most interesting finding is that they identify three major transitions, each involving multiple topics, which they attribute to paradigm shifts at around 1980, 1990 and 2000 (why they happen to be spaced out by 10 years is not discussed).

Response: The major transitions were determined based on timepoints where the net changes in slopes (combining positive and negative trends) were the largest over the time course. The goal was to show where major decreases and increases in topical prevalence were happening. Analysis of the data revealed that the shifts occurred every 10 years, which is curious to us also.

However, when it comes to explaining these shifts, the authors look for largely technical causes, such as adoption of recombinant DNA technologies, better approaches to creating transgenic plants, more efficient mutagenesis etc. Key conceptual changes, which to my mind is what paradigm shifts are all about, are not identified. I was left wondering whether this is a fundamental limitation of the approach, which can identify changes in popularity and scientific fashions but not paradigm shifts which relate to concepts and ideas.

Response: In retrospect, we completely agree with the reviewer that it is misleading to use “paradigm shift” to describe the changes and trends. We started the analysis with the hope of visualizing paradigm shifts but as the results came in, it became clear that the trends mostly reflected technological advances that contributed to the radiation of topics. Nonetheless, we do not intend to claim that such trends reflect paradigm shifts. Considering this, we have removed mentions of paradigm shifts in the revised manuscript to avoid implying that the shifts reflect changes in concepts and ideas. Our inability to detect true paradigm shifts is a limitation of our approach as the reviewer points out, and it is fair to say that what we uncovered reflects the popularity of topics instead. We have revised the discussion to incorporate the above point and further emphasize the inability of this approach to pinpoint conceptual advances.

To illustrate the limitation of the numerical approach, consider the topic of transposons (topic 62) which gained popularity in the 1980s. This is not considered a paradigm shift by the authors because only one topic is involved. Yet it could be considered as a paradigm shift (anticipated by Barbara McClintock in the 1950s) because it changed the way genomes were viewed from static to dynamic entities. This is not to say that the results presented in the paper are without value, but they provide trends that may be taken into account in a study of paradigm shifts rather than providing a way of identifying such shifts, which is what the paper claims.

Response: In our original manuscript, we did use transposon research as an example to show that we were only able to detect the signal of McClintock’s work when she received a Nobel Prize for it. In the revision, we have placed more emphasis on this to illustrate the limitations of our approach in the results and discussion and the conclusion. We have also removed mention of paradigm shifts. We do have some ideas in mind about how our analyses can be improved to identify true paradigm shifts, and we are optimistic that we will get there in the next few years.
