# Supplementary material for: Assessing the evolution of research topics in a biological field using plant science as an example
Source: PLoS Biol. 2024 May 23;22(5):e3002612. doi: 10.1371/journal.pbio.3002612 (PMC11115244; doi:10.1371/journal.pbio.3002612)
Supplement: Attachment — Submitted filename: responses_ml_shs.docx [file pbio.3002612.s024.docx]

**Editor comments:**

a) Please change the Title to make it more explicit. We do understand that you wish to present your study as a generalisable approach, with plant science as a case study, but we think that its specific interest to plant scientists would nonetheless be substantial, so we suggest something like: "Using machine learning and language models to assess the evolution of research topics within specific fields: the case of plant science"

Response: In light of the suggestion, we changed the title to: “Assessing the evolution of research topics in a biological field using plant science as an example”. We did not include machine learning and language model because they are already mentioned in the manuscript and their use in the title distracts the readers from the scientific focus on topical evolution.

b) Please address the remaining concerns raised by the reviewers. Reviewer #1 mentions a few issues where some biases may have crept in. S/he suggests a further small experiment to compare human and machine classifiers and requests some clarifications. Reviewer #2 would like you to discuss an apparent ~10-year periodicity in the data, which s/he wonders might be linked to career cycles.

Response: We have responded to both reviewers’ comments in the following sections and have performed the analysis suggested by Reviewer 1.

c) Please address my Data Policy requests below; specifically, we need you to supply the numerical values underlying Figs 1AB, 2B, 3ABCDE, 4ABC, 5ABCDEF, S1ABCD, S5ABCD, S6ABCD, S7ABCD, S8ABCD, S9, either as a supplementary data file or as a permanent DOI’d deposition. I note that you already have a zipped data supplement that contains 12 supp data files (plus other material), but it's unclear how this relates to the Figures. Please clarify the relationship to the Figure panels and/or supply the underlying numerical values.

Response: All numerical values supporting the figures and supplemental figures are now included as supplemental data: Fig 1A (Data S1), 1B/2BC (Data S3), 3 (Data S5), 4 (Data S9), 5ABCD (Data S11), 5EF (Data S12). S1 (Data S13), S5 (Data S5,S6), S6 (Data S9), S7ABC (Data S9), S7D/S8 (Data S11), and S9 (Data S12).

d) Please cite the location of the data clearly in all relevant main and supplementary Figure legends, e.g. “The data underlying this Figure can be found in S1 Data” or “The data underlying this Figure can be found in…”.

Response: We have included references to data in the relevant figures and supplemental figures.

e) Thank you for making your code available. However, because Github depositions can be readily changed or deleted, please make a permanent DOI’d copy (e.g. in Zenodo) and provide this URL in the manuscript and Data Availability Statement (see below).

Response: We have submitted the repo as a compressed archive to Zenodo with DOI 10.5281/zenodo.10894387, which is now referenced in the manuscript.

-----------------------------------------

**REVIEWS:**

***Reviewer #1:***

The authors have largely addressed my comments. This is an improvement of a strong manuscript, and I especially wish to thank the authors for the added _examples in their code repository.

Response: We also thank Reviewer 1 for the critical, constructive, and helpful comments that allowed us to further improve our work.

My prior main critique was the reported drop in Arabidopsis thaliana research. The language of the revised manuscript now is more precise to convey that this is a relative statement, relative to all other plant research. Further, I can confirm in an own reanalysis of PubMed, that there is a drop relative to other plant publications, when latter are assessed in the reference journals provided by the authors.
As the manuscript singles out Arabidopsis thaliana, a possible reading is that there is something special going on with Arabidopsis thaliana. However, it is unclear to me if these observations are specific to Arabidopsis thaliana, or a (relative) decrease of model system research, or a (relative) decrease of specific plant species being mentioned in abstracts. I suggest a word of caution or one control analysis.

Response: We focused on Arabidopsis because of its dominance in plant science research in terms of publications and funding. On a personal level, both authors have been working on Arabidopsis since the 90s and have witnessed its rapid rise and the “feeling” of its relative decline in the last 10-15 years. The main point we’d like to impress on the reader is that genomic technology has in some way significantly widened our ability to work with traditionally non-model species. This is emphasized on p.11.

A preceding peripheral point of mine is the estimation of the false-positive and false-negative rates. The authors now include an analysis. Based on the methods section it appears that these rates are estimated by testing consistency with the authors' respective training / testing sets, and/or cross-validation on held-out samples. While this strategy suggests consistency, it however does not tell if the authors' assumptions on plant and not-plant are correct. Further, these numbers could be biased when applied to the corpus of reference journals of which most publications should be about plants.
I suggest that the authors additionally randomly 50 papers labelled as "Plant" by their classifier, 50 random papers labelled as "not-plant", mix both groups of papers, only keep title and abstract, and have human curator blinded toward "Plant" and "not-plant" (and journal names of these papers etc.) label as "Plant" and "Not-Plant". Then compare human and computational labelling in a confusion matrix, and estimate overall number of false positives through Bayes' theorem, to account for plant science only being a minority of publications on PubMed.

Response: We concur with the reviewer that the issue of bias remains important and have enlisted five colleagues in the plant sciences (three graduate students in plant science and genetic graduate programs, one postdoctoral quantitative geneticist, and one postdoctoral molecular geneticist) to annotate 100 randomly selected abstracts as the reviewer suggested. Each record was annotated by two colleagues. Among 85 entries where the annotations are consistent between annotators, 48 were annotated as negative but with 7 predicted as positive (false positive rate=14.6%) and 37 were annotated as positive but with 4 predicted as negative (false negative rate=10.8%). We have incorporated the above results in p.4 and p.18.

We have also conducted an additional analysis asking how well we can recover records from plant science journals not included as positive examples during training because they did not meet the threshold of 2,000 records. We found that the false negative rate was 9.9%, which is similar to that obtained when we evaluated the results of the top 17 journals with the MeSH term “Plants”. The results of this analysis have been incorporated in the revised manuscript (p.4).

Another peripheral point is the usage of "keywords" to define the corpus. As stated by the authors in their reply they used the title or abstract to define concepts, not PubMed keywords. Along this point, I suggest further editing for clarity line 74 which speaks about keywords: "To this end, we first collected over 30 million PubMed records and narrowed down candidate plant science records with plant-related terms and taxon names as keywords"

Response: We have now replaced mention of keyword either by defining the ideas behind or use alternative wording, such as “search term”.

***Reviewer #2:***

The authors have addressed my concerns and the paper is now a more measured and better qualified description of their findings.  I wonder if the authors could comment on the 10 year cycle which they also find curious.  Could it be that this relates not only to the scientific advances but also to the fashion-driven aspect of science.  That is, for research to sound current and novel (important both for grants and publications), it needs to latch on to new buzz words.  As these new words become prevalent, yet new buzz words are needed.  So the ten year cycle could reflect, at least in part, the time it takes for buzz-word to peak and saturate.  Given that a  PhD takes around 4-5 years, a grant is typically 3-5 years, and a scientific career lasts around 30-40 years, a 10 year cycle would correspond to two PhDs, 2-3 grants, or one third or quarter of a scientific career.  According to this hypothesis, similar cycle durations would be predicted for other scientific disciplines, which could be tested through further studies.

Response: This is something we have not thought about. We appreciate the idea but are concerned that the sample size of three (major shifts) is too small for us to derive a general trend and to speculate on the cause. We have mentioned this briefly in the discussion.
